# A concise access to bridged [2,2,1] bicyclic lactones with a quaternary stereocenter via stereospecific hydroformylation

**DOI:** 10.1038/s41467-021-25569-5

**Published:** 2021-09-06

**Authors:** Shuailong Li, Zhuangxing Li, Mingzheng Li, Lin He, Xumu Zhang, Hui Lv

**Affiliations:** 1 Key Laboratory of Biomedical Polymers of Ministry of Education & College of Chemistry and Molecular Sciences, Engineering Research Center of Organosilicon Compounds & Materials, Ministry of Education, Sauvage Center for Molecular Sciences, Wuhan University, Wuhan, China; 2 Key Laboratory for Green Processing of Chemical Engineering of Xinjiang Bingtuan, School of Chemistry and Chemical Engineering, Shihezi University, Xinjiang Uygur Autonomous Region, Xinjiang, China; 3 Grubbs Institute and Department of Chemistry, Southern University of Science and Technology, Shenzhen, Guangdong China

**Keywords:** Homogeneous catalysis, Asymmetric synthesis

## Abstract

Chiral bridged [2,2,1] bicyclic lactones are privileged structural units in pharmaceutics and bioactive nature products. However, the synthetic methods for these compounds are rare. Here we report an efficient method for enantioselective construction of bridged [2,2,1] bicyclic lactones bearing a quaternary stereocenter via Rh-catalyzed asymmetric hydroformylation/intramolecular cyclization/pyridium chlorochromate (PCC) oxidation. By employing a hybrid phosphine-phosphite chiral ligand, a series of cyclopent-3-en-1-ols are transformed into corresponding γ-hydroxyl aldehydes with specific syn-selectivity. Then, hemiacetals form in situ and oxidation with PCC in one-pot affords bridged [2,2,1] bicyclic lactones in high yields and excellent enantiomeric excess. Replacing the hydroxyl group by an ester group, cyclopentanecarbaldehydes with a chiral all-carbon quaternary stereocenter in the γ-position can be generated efficiently.

## Abstract (web summary)

Enantiomeric bridged [2,2,1] bicyclic lactone skeletons and their ring-opening products are important scaffolds widely occurring in both pharmaceutics and biology active compounds. Here the authors show an efficient method for enantioselective construction of these compounds bearing a quaternary stereocenter via Rh-catalyzed asymmetric hydroformylation/intramolecular cyclization/ pyridinium chlorochromate oxidation.

## Introduction

**Fig. 1:**
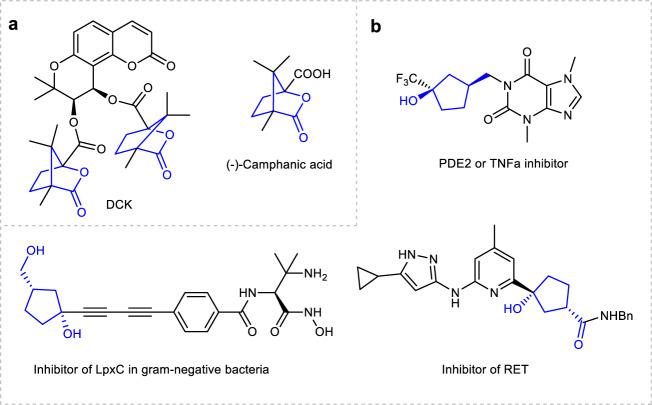
Pharmaceutics and bioactive compounds containing bridged [2,2,1] bicyclic lactones and their ring-opening derivatives. **a** Bioactive molecules containing bridged [2,2,1] bicyclic lactones. **b** Ring-opening derivatives of bridged [2,2,1] bicyclic lactones.

**Fig. 2:**
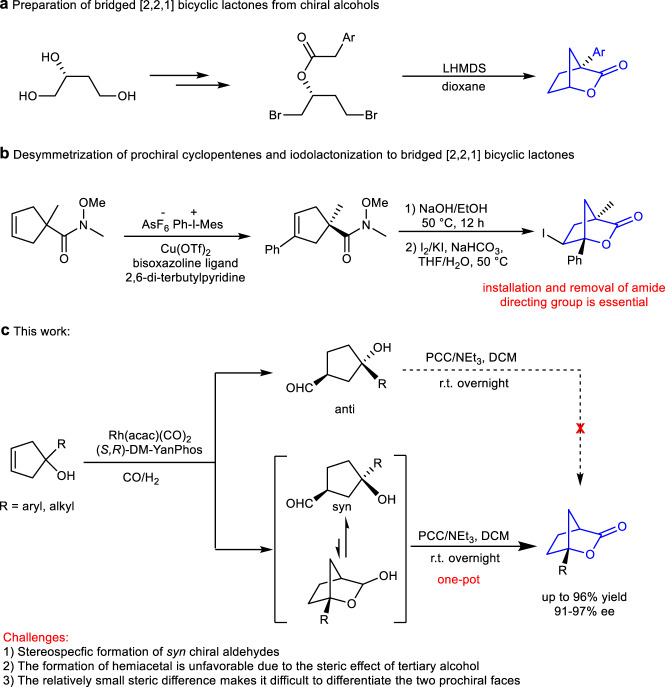
Methods for synthesis of chiral bridged [2,2,1] bicyclic lactones. **a** Synthesis of bridged [2,2,1] bicyclic lactones by using chiral alcohol as starting material. **b** Preparation of bridged [2,2,1] bicyclic lactones by amide-directed Heck reaction and iodolactonization. **c** Stereopecific hydroformylation and lactonization to form bridged [2,2,1] bicyclic lactones.

Enantiomeric bridged [2,2,1] bicyclic lactone skeletons and their ring-opening products, cyclopentanols bearing two chiral centers, are important scaffolds widely occurring in both pharmaceutics and biology active compounds (Fig. 1)^1,2^. For example, chiral molecular DCK is a kind of anti-HIV agent^3–5^. Consequently, the synthesis of bridged [2,2,1] bicyclic lactones received wide attentions and several approaches have been developed. The typical methods include Baeyer–Villiger oxidation^6^, esterification^7,8^, halolactonization^9,10^, electrocatalytic reaction^11^, and others^12–14^. However, most of these approaches focused on the synthesis of racemic bridged [2,2,1] bicyclic lactones and multistep synthesis was necessary to achieve these transformations. To date, there are only two examples on the construction of chiral bridged [2,2,1] bicyclic lactones in an enantioselective manner. In 2015, Dominguez developed a synthetic route to chiral bridged [2,2,1] bicyclic lactones by using chiral alcohol as starting material (Fig. 2a)^15^. In 2018, Zhu et al. developed a copper-catalyzed enantioselective arylative desymmetrization reaction of prochiral cyclopentenes, and then followed by hydrolysis and intramolecular iodolactonlization to generate bridged [2,2,1] bicyclic lactones. However, the installation and removal of an amide directing group were essential to this synthetic route, which resulted in relatively low atom economy (Fig. 2b)^16^. Therefore, the development of a concise and efficient method to produce bridged [2,2,1] bicyclic lactones is highly desirable.

Asymmetric hydroformylation (AHF) represents an efficient approach for asymmetric formation of C–C bond in an atomic economic manner^17–25^, and the aldehyde products can be easily converted to versatile functional compounds, such as chiral alcohols, acids, amines and esters^26–34^, thus AHF has been widely investigated and some significant progresses have been made^35–45^. However, most of the work focused on the AHF of monosubstituted olefins and disubstituted alkenes, and only one chiral center was introduced into the product. As a result, the construction of chiral aldehydes containing a quaternary center and a tertiary center simultaneously by AHF is rarely exploited. To the best of our knowledge, there was only one report achieved this transformation by using desymmetric hydroformylation strategy, but the substrate scope was limited to cyclopropenes with high ring strain. Furthermore, only moderate to good enantioselectivities were obtained (≤83% ee)^46^. Consequently, highly efficient synthesis of multichiral aldehydes bearing a quaternary stereocenter is still a problematic issue in this field.

Recently, our group developed a Rh-catalyzed AHF of 1,1-disubstituted allyl alcohols to generate γ-butyrolactones^47^. We envisioned that the similar transformation might occur if 1-substituted cyclopent-3-en-1-ols were used as starting material, providing efficient access to bridged [2,2,1] bicyclic lactones with a quaternary stereocenter. However, this transformation faces several challenges (Fig. 2c). First, it is very difficult to generate chiral aldehydes with exclusive *syn*-selectivity through AHF of 1-substituted cyclopent-3-en-1-ols, but it’s an essential factor to form bridged [2,2,1] bicyclic lactones in high yield. Moreover, the isomerization of alkene is difficult to be inhibited in AHF of cyclic olefines^48–53^, which further increases the difficulty of producing *syn* γ-hydroxyaldehydes. Second, the generation of the hemiacetals is unfavorable in this transformation because the large steric hindrance of tertiary alcohols greatly decreased the nucleophilic ability of hydroxy group to aldehydes. In addition, the relatively small steric difference between the two prochiral faces makes it difficult to obtain high enantioselectivity. Thus, the development of a highly efficient method for asymmetric synthesis of bridged [2,2,1] bicyclic lactones containing a quaternary stereocenter is still a challenge.

Herein, we report one-pot synthesis of chiral bridged [2,2,1] bicyclic lactones from readily available cyclopent-3-en-1-ols, delivering target products with good yields and high enantioselectivities, which provides efficient access to chiral bridged [2,2,1] bicyclic lactones containing a quaternary stereocenter.

## Results and discussion

**Fig. 3:**
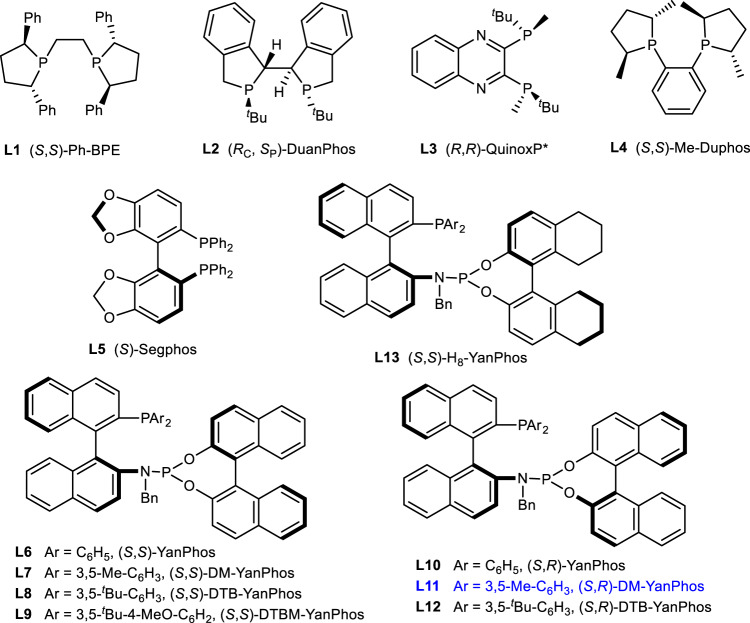
Ligands evaluation for asymmetric hydroformylation of 1a. **L1-L5** are commercially available diphosphine ligands, **L6-L13** are Yanphos-type ligands developed in our lab.

Initially, considering only *syn* oxo-products can be transfered to corresponding bridged [2,2,1] bicyclic lactones, AHF of **1a** was investigated to obtain **2a** stereospecifically. When (*S*,*S*)-Ph-BPE, the representative ligand in AHF^54^, was employed, **1a** was transformed into oxo-product with high conversion and excellent ee, along with good diastereoselectivity (Table 1, entry 1). (*Rc*,*Sp*)-Duanphos showed low activity in this transformaiton albeit with good stereocontrol (Table 1, entry 2). (*R*,*R*)-Quinoxp, which performed well in asymmetric hydrogenation reactions^55–59^, afforded target product in low yield with moderate enantioselectivity (Table 1, entry 3). The reaction was totally inhibitted when (*S*,*S*)-Me-Duphos and (*S*)-Segphos were employed (Table 1, entries 4–5). In order to obtain higher enantio- and diastereoselectivity, a series of YanPhos-type ligands with different axial chirality (Fig. 3), which were developed by our group, were evaluated^60–63^. The results showed that all YanPhos-type ligands had good catalytic activity for this transformation, but there were big differences in the control of enantioselectivity and diastereoselectivity. Generally, YanPhos containing (*S*,*R*) axial chirality had better perfomance than that of YanPhos with (*S*,*S*) axial chirality (Table 1, entries 6–13). When (*S*,*R*)-DM-YanPhos was employed (Table 1, entry 11), the target product was obtained with the best diastereo- and enenatioselectivity.

**Table 1:** Ligand screening in the asymmetric hydroformylation of 1a^a^.


Entry	Ligand	Conv. (%)^b^	Ee (%) of 3a^c^	(2a + 2a′)/2a″^b^
1	**L1**	90	94	12.5
2	**L2**	43	90	5.3
3	**L3**	37	−73	2.9
4	**L4**	Trace	ND	ND
5	**L5**	Trace	ND	ND
6	**L6**	>99	45	4.2
7	**L7**	>99	50	5.3
8	**L8**	>99	79	>20
9	**L9**	>99	70	7.7
10	**L10**	>99	96	11.6
11	**L11**	>99(61)^d^	94	>20
12	**L12**	>99	88	11.1
13	**L13**	>99	39	2.4

*ND* not detected.

^a^The reaction of **1a** (0.2 mmol) was performed in the presence of Rh(acac)(CO)_2_ (2 mol%), **L** (4 mol%), H_2_/CO = 5/5 bar in toluene (1 mL) at 70 °C for 24 h, then PCC (0.5 mmol) in DCM (4 mL) 25 °C for 12 h. After the completion of AHF, partial of reaction solution was took out for ^1^H NMR to detect the conversion of AHF and the ratio of (**2a** + **2a′**)/**2a″**, the rest of solution was treated with PCC to give target product **3a**.

^b^Determined by 1H NMR spectroscopy.

^c^Determined by HPLC analysis on a chiral stationary phase.

^d^Isolated yield.

Having established the optimized reaction condition for AHF of **1a**, we attempt to synthesize bridged [2,2,1] bicyclic lactone **3a** in one pot by sequential AHF/intramolecular cyclization/dehydrogenation oxidation (Table 2). Based on our previous work^64^, PCC (pyridinium chlorochromate) was selected as oxidant and delivered target product **3a** with moderate yield (entry 1). Increasing reaction temperature could not improve the yield (entry 2). Considering the bulky steric hindrance greatly decreased the nucleophilicity of tertiary alcohol^65^, several additives were screened to promote the cyclization of **2a**. Acetic acid lead to a significant drop in yield, NaOAc resulted in the decrease of yield to some extent. To our delight, K_2_CO_3_, Cs_2_CO_3_ and NEt_3_ can promote this reaction, affording target product in high yield without compromising the enantioselectivity (entries 5–7). However, a racemization occurred when NaOH was used, resulting in the decrease of ee and dr values (entry 8, 40% yield, 80% ee). Thus, one practical method for synthesis of bridged [2,2,1] bicyclic lactones was most effective with (*S*,*R*)-DM-YanPhos as the ligand in AHF and NEt_3_ as additive in PCC oxidation.

**Table 2:** Additive screening in the PCC oxidation^a^.


Entry	Additive	Yield (%)	Ee (%) ^[b]^
1	–	61	94
2^c^	–	56	94
3	AcOH	26	94
4	NaOAc•3H_2_O	49	94
5	K_2_CO_3_	82	94
6	Cs_2_CO_3_	85	94
7	NEt_3_	90	94
8	NaOH	40	80

^a^The reaction of **1a** (0.2 mmol) was performed in the presence of Rh(acac)(CO)_2_ (2 mol%), **L11** (4 mol%), H_2_/CO = 5/5 bar in toluene (1 mL) at 70 °C for 24 h, The reaction was cooled to room temperature and the pressure was carefully released in a well-ventilated hood, then the mixture was treated with PCC (0.5 mmol), additive (0.1 mmol) in DCM (4 mL) 25 °C for 12 h in one pot.

^b^Determined by HPLC analysis on a chiral stationary phase.

^c^Performed at 40 °C.

**Fig. 4:**
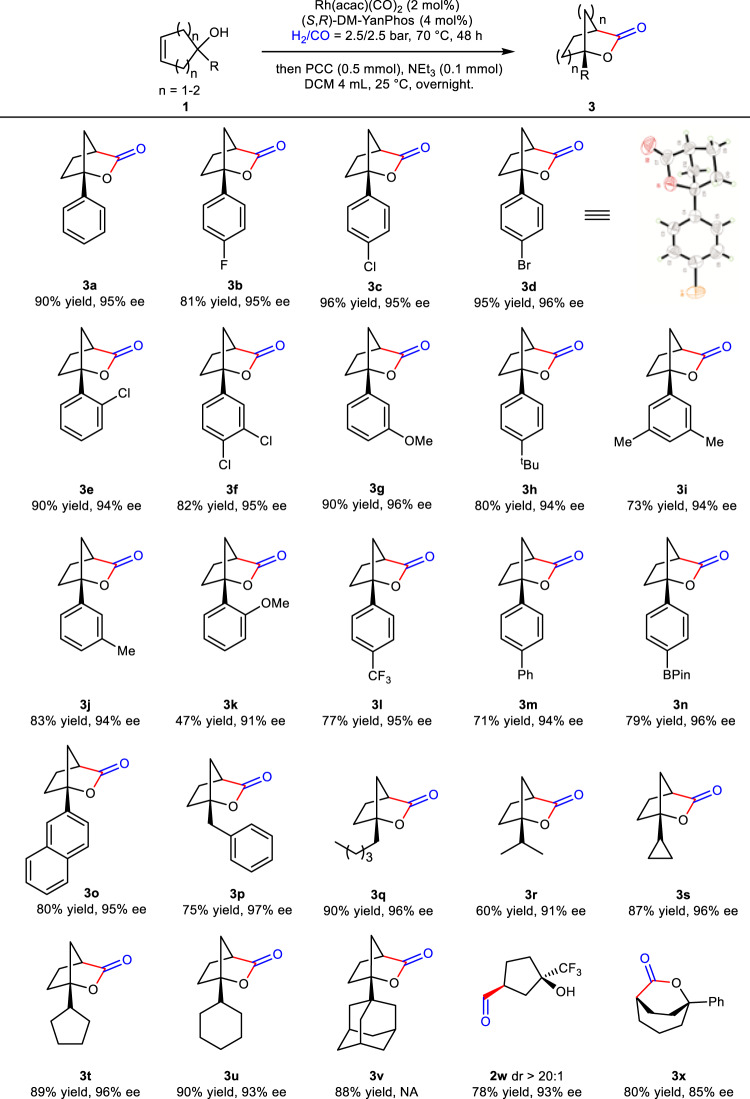
Scope of 1-substituted cyclopent-3-en-1-ols and 1-phenylcyclohept-4-en-1-ol. Reaction conditions: The reaction of **1a** (0.2 mmol) was performed in the presence of Rh(acac)(CO)_2_ (2 mol%), **L11** (4 mol%), H_2_/CO = 2.5/2.5 bar in toluene (1 mL) at 70 °C for 48 h, The reaction was cooled to room temperature and the pressure was carefully released in a well-ventilated hood, then the mixture was treated with PCC (0.5 mmol), Et_3_N (0.1 mmol) in DCM (4 mL) at 25 °C, overnight. NA = not available.

Under the optimal conditions, we investigated the substrate scope. All the bridged [2,2,1] bicyclic lactones were prepared in good yields with excellent enantioselectivities (Fig. 4). Substrates bearing halides on the phenyl ring performed well in this transformation, giving target products with high yields and excellent ee’s (**3b**–**3f**). The absolute configuration of **3d** was confirmed by X-ray crystallographic analysis. Electron-donating and electron-withdrawing substituted groups on the phenyl ring were also tolerated, furnishing **3f**, **3g**, **3h**, **3i**, **3j** with high yields and excellent enantioselectivities, respectively. The yield of **3k** was dropped sharply due to the ortho effect of methoxy group, but the high enantioselectivity was remained. In addition, functional groups, such as trifloromethyl, phenyl and borate (**3l**–**3n**) on the *para*-position of the benzene ring were compatible, and the corresponding products were afforded with moderate to good yields and high ee’s. Replacing phenyl by a naphthyl group (**3o**), the reaction also proceeded smoothly, providing the desired compound with high yield and excellent ee. Notably, alkyl substituents, such as benzyl, *n*-hexyl, isopropyl, cyclopropyl, cyclopentyl and cyclohexyl were also well tolerated in this transformation, delivering bridged [2,2,1] bicyclic lactones with excellent ee’s and high yields (**3p**–**3u**). Cyclopent-3-en-1-ol bearing a bulky sterically hindered damantyl group also proceeded effectively, affording target product with high yield (**3v**). Moreover, the oxo-product **2w** was produced with high diastereoselectivity and excellent enantioselectivity^66^. Interestingly, 1-phenylcyclohept-4-en-1-ol, a challenge substrate for AHF because of the substituent far away from reaction site, which made it difficult to control the stereoselectivity, also worked very well in this transformation, delivering 6-oxabicyclo[3.2.2]nonan-7-one **3x** with high yield and good enantioselectivity.

**Fig. 5:**
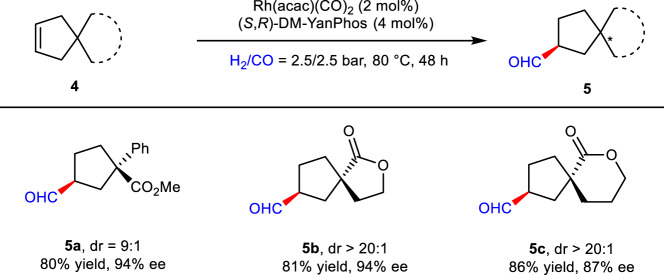
Substrates for synthesis of chiral aldehydes with an all-carbon quaternary stereocenter. Reaction conditions: **4** (0.2 mmol), Rh(acac)(CO)_2_ (2 mol%), **L11** (4 mol%), H_2_/CO = 2.5/2.5 bar, toluene (1 mL), 80 °C, 48 h. The dr value of **5a**–**5c** were determined by ^1^H NMR spectroscopy.

Encouraged by the success of desymmetric strategy for construction of chiral bridged [2,2,1] bicyclic lactones with a O-subsitituted quaternary center, primary exploration on efficient synthesis of cyclopentanecarbaldehyde with an all-carbon quaternary stereocenter was conducted. As shown in Fig. 5, when symmetric cyclopentene with phenyl and ester substituents was employed, the desired chiral aldehyde **5a** was generated in good yield with high diastereo- and enantioselectivity. Moreover, all-carbon substituted chiral spiro-lactones could also be efficiently synthesized by this strategy, delivering target products with good yields and high enantioselectivities (**5b**, **5c**).

**Fig. 6:**
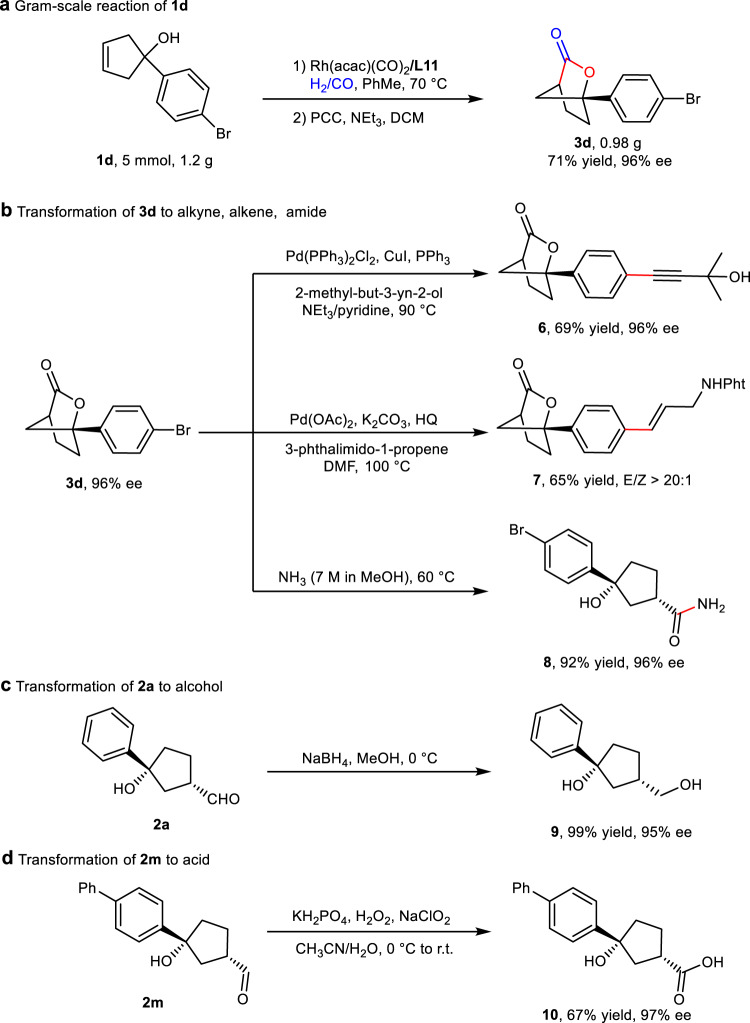
Gram-scale reaction and transformations of oxo-products and ring-opening reaction of bridged [2,2,1] bicyclic lactones. **a** Gram-scale reaction. **b** Transformation of **3d** to corresponding alkyne, alkene and amide. **c** Reduction of **2a** to alcohol. **d** Oxidation of **2m** to acid.

To further demonstrate the practical utility of this methodology, a gram-scale reaction of **1d** was conducted in the presence of 0.2 mol% catalyst, then treated with NEt_3_ and PCC, **3d** was obtained with high yield and without any loss in enantioselectivity (Fig. 6a). Under the classical conditions of Sonogashira reaction and Heck reaction, alkyne and alkene groups were effectively incorporated into **3d**, and the chiral bridged [2,2,1] bicyclic lactone skeleton was not affected (Fig. 6b). In order to obtain the ring-opening derivatives of bridged [2,2,1] bicyclic lactones, **3d** was treated with methanol solution of ammonia, furnishing chiral amide **8** with high yield and excellent ee (Fig. 6b). The hydroformylation product **2a** can be efficiently reducted by NaBH_4_, affording chiral dual alcohol **9** in high yield (Fig. 6c). Under a mild condition, the bioactive chiral acid **10** was readily prepared by oxidation of **2m** with H_2_O_2_ and NaClO_2_ (Fig. 6d)^67^.

**Fig. 7:**
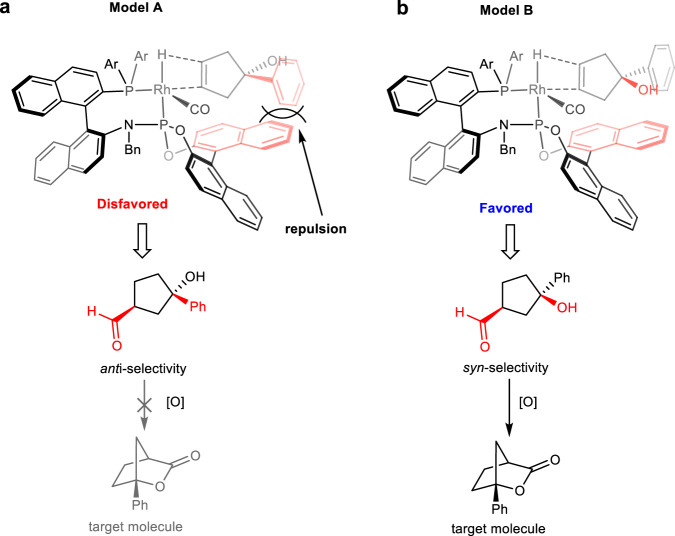
Proposed stereochemical model. **a** Disfavored stereochemical model. **b** Favored stereochemical model.

Based on the experimental results and the related mechanisum studies on Rh/Yanphos catalyzed AHF in literature^27,61^, we proposed the possible stereochemical model to explain the reason for high stereoselectivity in this transformation. According to the orientation of substrate closed to the catalyst, there are two possible coordination models (Fig. 7). In model A, there is a large repulsion between the benzene ring of the substrate and the naphthalene ring of ligand, hence this model is disfavored. By comparison, the repusion betweeen substrate and catalyst is much smaller in model B due to the small streric hindrance of hydroxy group, thus the asymmetric hydrofomylation reaction occurred smoothly, delivering chiral γ-hydroxyaldehyde with excellent *syn*-selectivity, which can be efficiently transfer to the target bridged [2,2,1] bicyclic lactone in the presence of PCC.

In this work, we have developed an efficient and concise method for preparing bridged [2,2,1] bicyclic lactones bearing a quaternary stereocenter from readily available starting material by one-pot process. This methodology shows excellent substrate compatibility and excellent stereocontrol, giving target products with high yields and excellent enantioselectivities. In addtion, this protocol also provides a useful strategy for construction of chiral aldehydes with an all-carbon quaternary stereocenter. Gram-scale reaction and diverse transformations of the oxo-products and bridged [2,2,1] bicyclic lactones demonstrate the utility of this method in synthetic chemistry. Further exploration on the construction of quaternary chiral center by AHF is ongoing in our laboratory.

## Methods

In a glovebox filled with argon, to a 5 mL vial equipped with a magnetic bar was added (*S*,*R*)-DM-YanPhos (0.004 mmol) and Rh(acac)(CO)_2_ (0.002 mmol in 1 mL toluene). After stirring for 10 min, the mixture was charged to substrate (0.2 mmol). The vial was transferred into an autoclave and taken out of the glovebox. The argon gas was replacement with hydrogen gas for three times, and then hydrogen (2.5 bar) and carbon monoxide (2.5 bar) were charged in sequence. The reaction mixture was stirred at 70 °C (oil bath) for 48 h. The reaction was cooled to room temperature and the pressure was carefully released in a well-ventilated hood. The solution was transferred into a solution of PCC (0.5 mmol) and triethylamine (0.1 mmol) in 4 mL dichloromethane, the reaction mixture was stirred at 25 °C (oil bath) overnight. The solution was concentrated and the product was isolated by column chromatography using petrol ether/EtOAc (30:1–10:1) as eluent to give the desired product.

## Supplementary information

Supplementary Information

## Data Availability

The data supporting the findings of this study are available in the paper and its Supplementary Information, further data are available from the corresponding author on request. The X-ray crystallographic coordinates for structures reported in this study have been deposited at the Cambridge Crystallographic Data Centre (CCDC), under deposition numbers CCDC 2034549 (**3d**). These data can be obtained free of charge from the Cambridge Crystallographic Data Centre via www.ccdc.cam.ac.uk/data_request/cif.
