# Author Correction: A concise access to bridged [2,2,1] bicyclic lactones with a quaternary stereocenter via stereospecific hydroformylation

**DOI:** 10.1038/s41467-021-26074-5

**Published:** 2021-09-27

**Authors:** Shuailong Li, Zhuangxing Li, Mingzheng Li, Lin He, Xumu Zhang, Hui Lv

**Affiliations:** 1 Key Laboratory of Biomedical Polymers of Ministry of Education & College of Chemistry and Molecular Sciences, Engineering Research Center of Organosilicon Compounds & Materials, Ministry of Education, Sauvage Center for Molecular Sciences, Wuhan University, Wuhan, China; 2 Key Laboratory for Green Processing of Chemical Engineering of Xinjiang Bingtuan, School of Chemistry and Chemical Engineering, Shihezi University, Xinjiang Uygur Autonomous Region, Xinjiang, China; 3 Grubbs Institute and Department of Chemistry, Southern University of Science and Technology, Shenzhen, Guangdong China

**Keywords:** Homogeneous catalysis, Asymmetric synthesis

Correction to: *Nature Communications* 10.1038/s41467-021-25569-5, published online 06 September 2021.

The original version of this Article contained an error in Fig. 7, in which P atoms in the catalyst structures were labelled as PH not P. This has been corrected in both the PDF and HTML versions of the Article.

