# Supplementary material for: A concise access to bridged [2,2,1] bicyclic lactones with a quaternary stereocenter via stereospecific hydroformylation
Source: Nat Commun. 2021 Sep 6;12:5279. doi: 10.1038/s41467-021-25569-5 (PMC8421442; doi:10.1038/s41467-021-25569-5)
Supplement: Supplementary file 1 — Supplementary Information [file 41467_2021_25569_MOESM1_ESM.pdf]

A Concise Access to Bridged [2,2,1] Bicyclic Lactones with A Quaternary Stereocenter *via* Stereospecific Hydroformylation

Li et al.

Supplementary Methods

All reactions and manipulations that were sensitive to moisture or air were performed in an argon-filled glovebox or using standard schlenk techniques, unless otherwise noted. Solvents were dried with standard procedures, degassed with N_2 and transferred by syringe. NMR spectra were recorded on Bruker ADVANCE III (400 MHz) spectrometers for ^1H NMR and ^{13}C NMR. $CDCl_3$ was the solvent used for the NMR analysis, with tetramethylsilane as the internal standard. Chemical shifts were reported upfield to TMS (0.00 ppm) for ^1H NMR and relative to $CDCl_3$ (77.0 ppm) for ^{13}C NMR. Optical rotation was determined using a Perkin Elmer 343 polarimeter. GC analysis was carried out on gas chromatography using chiral capillary columns. HPLC analysis was conducted on an Agilent 1260 Series instrument. Thin layer chromatography (TLC) was performed on EM reagents 0.25 mm silica 60-F plates. All new products were further characterized by HRMS. A positive ion mass spectrum of sample was acquired on a Thermo LTQ-FT mass spectrometer with an electrospray ionization source. The substrates were synthesized according to the literature reference [1-4].

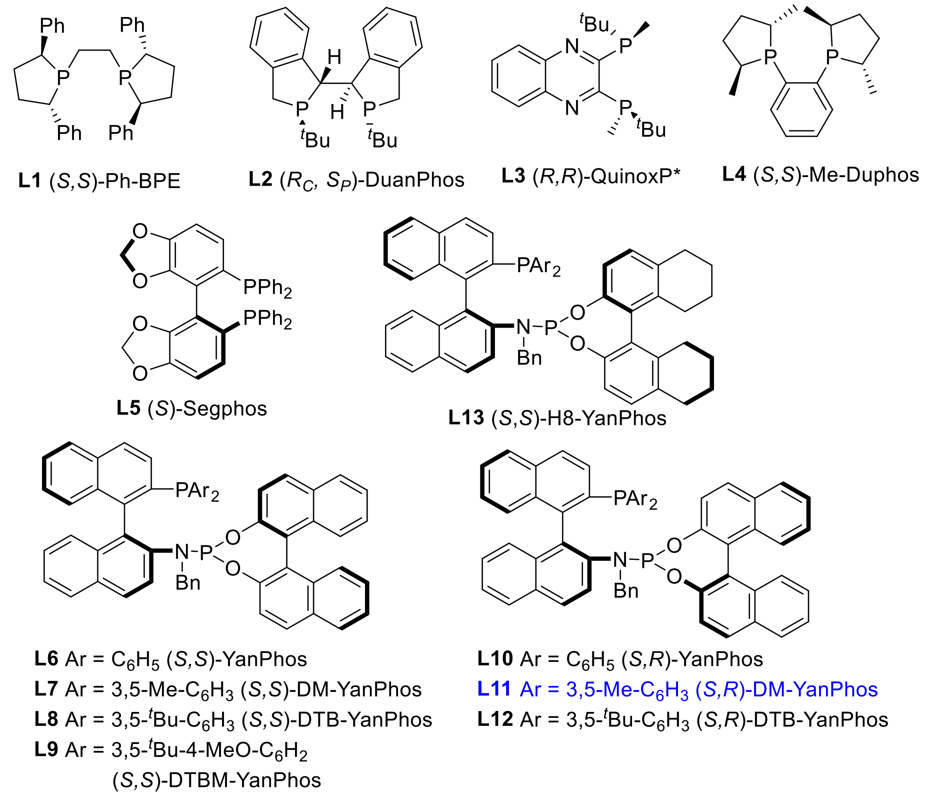

Supplementary Figure 1. Ligands evaluated for asymmetric hydroformylation of 1a.

Supplementary Table 1. Ligand screening for the asymmetric hydroformylation and PCC oxidation of 1a[a]

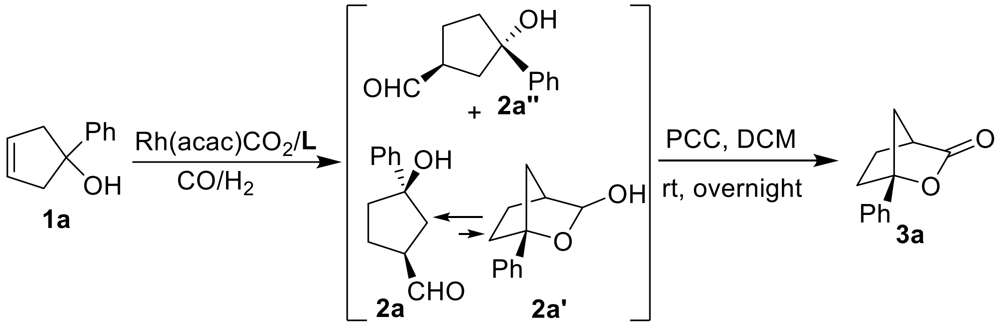

Entry	Ligand	Conv. (%)[b]	Ee (%) of **3a**[c]	**2a+2a'/2a''**[b]
1	**L1**	90	94	12.5
2	**L2**	43	90	5.3
3	**L3**	37	-73	2.9
4	**L4**	Trace	ND	ND
5	**L5**	Trace	ND	ND
6	**L6**	>99	45	4.2
7	**L7**	>99	50	5.3
8	**L8**	>99	79	>20
9	**L9**	>99	70	7.7
10	**L10**	>99	96	11.6
11	**L11**	>99	94(61)[d]	>20
12	**L12**	>99	88	11.1
13	**L13**	>99	39	2.4

[a]The reaction of **1a** (0.2 mmol) was performed in the presence of Rh(acac)CO_2 (2 mol%), L (4 mol%), H_2/CO = 5/5 bar in toluene (1 mL) at 70 °C for 24 h, then PCC (0.5 mmol) in DCM (4 mL) 25 °C for 12 h. [b]Determined by ^{1}H NMR spectroscopy. [c]Determined by HPLC analysis on a chiral stationary phase. [d]Isolated yield. ND: not detected.

Supplementary Table 2. Additive screening for the lactonization[a]

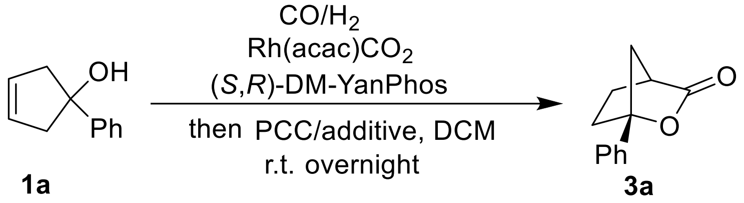

Entry	Additive	Iso. yield (%)	Ee (%) of **3a**[b]
1	-	61	94
2[c]	-	56	94
3	AcOH	26	94
4	$NaOAc{\bullet}3H_2O$	49	94
5	K_2CO_3	82	94
6	Cs_2CO_3	85	94
7	NEt_3	90	94
8	NaOH	40	80

[a]The oxidation reaction, after hydroformylation, was performed in the presence of PCC (0.5 mmol), additive (0.1 mmol) in DCM (4 mL) 25 °C for 12 h. [b]Determined by HPLC analysis on a chiral stationary phase. [c]Performed at 40 °C.

General procedure for one-pot synthesis of chiral bridged[2,2,1] bicyclic lactones

In a glovebox filled with argon, to a 5 mL vial equipped with a magnetic bar was added (*S*,*R*)-DM-YanPhos (0.004 mmol) and Rh(acac)$(CO)_2$ (0.002 mmol in 1 mL toluene). After stirring for 10 minutes, the mixture was charged to substrate (0.2 mmol). The vial was transferred into an autoclave and taken out of the glovebox. The argon gas was replacement with hydrogen gas for three times, and then hydrogen (2.5 bar) and carbon monoxide (2.5 bar) were charged in sequence. The reaction mixture was stirred at 70 °C (oil bath) for 48 h. The reaction was cooled to room temperature and the pressure was carefully released in a well-ventilated hood. The solution was transferred into a solution of pyridinium chlorochromate (PCC) (0.5 mmol) and triethylamine (0.1 mmol) in 4 mL dichloromethane, the reaction mixture was stirred at 25 °C (oil bath) overnight. The solution was concentrated and the product was isolated by column chromatography. The enantiomeric excesses were determined by GC analysis or by HPLC analysis.

NMR, optical rotation and HRMS Data of 3 and 2w

(1*R*,4*S*)-1-phenyl-2-oxabicyclo[2.2.1]heptan-3-one (3a)

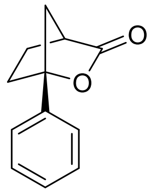

Colorless oil. 33.9 mg, 90% yield, 95% ee, $[\alpha]^{20}_{D}$ = +42.3 (*c* = 1.0, $CHCl_3$). ^1H NMR (400 MHz, $CDCl_3$) δ 7.47 - 7.34 (m, 5 H), 3.08 - 3.07 (m, 1 H), 2.32 - 2.29 (m, 1 H), 2.21 - 2.13 (m, 3 H), 2.12 - 2.09 (m, 1 H), 2.00 - 1.91(m, 1 H) ppm. ^{13}C NMR (100 MHz, $CDCl_3$) δ 177.6, 136.8, 128.5, 128.5, 125.4, 92.4, 44.6, 44.0, 34.6, 24.7 ppm. The enantio-meric excess was determined by HPLC on Chiralcel OD-H column, hexane/isopropanol = 97/3; flow rate = 1.0 mL/min; UV detection at 220 nm; t_R = 27.7 min (major), t_R = 32.5 min (minor). **HRMS** calculated $[M+Na]^+$ for $C_{12}H_{12}NaO_2$ = 211.0730, found: 211.0727.

(1*R*,4*S*)-1-(4-fluorophenyl)-2-oxabicyclo[2.2.1]heptan-3-one (3b)

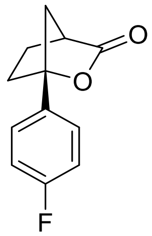

Colorless oil. 33.4 mg, 81% yield, 95% ee, $[\alpha]^{20}_{D}$ = +37.3 (*c* = 1.0, $CHCl_3$). ^1H NMR (400 MHz, $CDCl_3$) δ 7.46 - 7.41 (m, 2 H), 7.12 - 7.06 (m, 2 H), 3.08 (s, 1 H), 2.32 - 2.28 (m, 1 H), 2.20 - 2.12 (m, 3 H), 2.08 (dd, *J* = 10.3, 1.1 Hz, 1 H), 2.01 - 1.93 (m, 1 H) ppm. ^{13}C NMR (100 MHz, CDCl3) δ 177.4, 162.7 (d, *J* = 247.6 Hz), 132.7 (d, *J* = 3.2 Hz), 127.4 (d, *J* = 8.3 Hz), 115.5 (d, *J* = 21.6 Hz), 91.8, 44.6, 44.0, 34.6, 24.7 ppm. The enantiomeric excess was determined by HPLC on Chiralcel OJ-H column, hexane/isopropanol = 95/5; flow rate = 1.0 mL/min; UV detection at 210 nm; t_R = 37.7 min (major), t_R = 43.4 min (minor). **HRMS** calculated $[M+Na]^+$ for $C_{12}H_{11}FNaO_2$ = 229.0635, found: 229.0635.

(1*R*,4*S*)-1-(4-chlorophenyl)-2-oxabicyclo[2.2.1]heptan-3-one (3c)

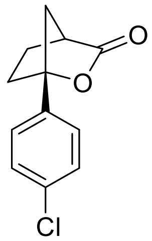

White solid. 42.8 mg, 96% yield, 95% ee, mp = 92 - 94 °C. $[\alpha]^{20}_{D}$ = +34.3 (*c* = 1.0, $CHCl_3$). ^1H NMR (400 MHz, $CDCl_3$) δ 7.41 - 7.36 (m, 4 H), 3.09 - 3.08 (m, 1 H), 2.29 - 2.26 (m, 1 H), 2.19 - 2.12 (m, 3 H), 2.09 (dd, *J* = 10.3, 1.0 Hz, 1 H), 2.00 - 1.92 (m, 1 H) ppm. ^{13}C NMR (100 MHz, $CDCl_3$) δ 177.2, 135.4, 134.4, 128.8, 126.9, 91.7, 44.6, 44.0, 34.7, 24.7 ppm. The enantiomeric excess was determined by HPLC on Chiralcel OJ-H column, hexane/isopropanol = 95/5; flow rate = 1.0 mL/min; UV detection at 220 nm; t_R = 33.8 min (major), t_R = 40.0 min (minor). **HRMS** calculated $[M+Na]^+$ for $C_{12}H_{11}ClNaO_2$ = 245.0340, found: 245.0339.

(1*R*,4*S*)-1-(4-bromophenyl)-2-oxabicyclo[2.2.1]heptan-3-one (3d)

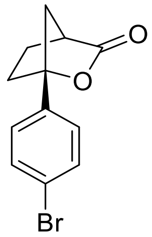

White solid. 50.7 mg, 95% yield, 96% ee, mp = 100 - 102 °C. $[\alpha]^{20}_{D}$ = +27.6 (*c* = 1.0, $CHCl_3$). ^1H NMR (400 MHz, $CDCl_3$) δ 7.55 - 7.52 (m, 2 H), 7.35 - 7.31 (m, 2 H), 3.09 - 3.08 (m, 1 H), 2.27 (dd, *J* = 10.4, 1.5 Hz, 1 H), 2.19 - 2.13 (m, 3 H), 2.09 (dd, *J* = 10.3, 1.0 Hz, 1 H), 2.00 - 1.92 (m, 1 H) ppm. ^{13}C NMR (100 MHz, $CDCl_3$) δ 177.2, 135.9, 131.7, 127.1, 122.5, 91.7, 44.6, 44.0, 34.7, 24.7 ppm. The enantiomeric excess was determined by HPLC on Chiralcel OJ-H column, hexane/isopropanol = 95/5; flow rate = 1.0 mL/min; UV detection at 220 nm; t_R = 37.6 min (major), t_R = 44.8 min (minor). **HRMS** calculated $[M+Na]^+$ for $C_{12}H_{11}BrNaO_2$ = 267.0015, found: 267.0017.

(1*R*,4*S*)-1-(2-chlorophenyl)-2-oxabicyclo[2.2.1]heptan-3-one (3e)

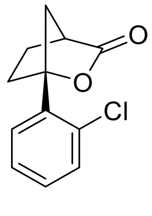

Colorless oil. 40.1 mg, 90% yield, 94% ee, $[\alpha]^{20}_{D}$ = +31.8 (c = 1.0, $CHCl_3$). ^1H NMR (400 MHz, $CDCl_3$) δ 7.72 - 7.70 (m, 1 H), 7.40 - 7.38 (m, 1 H), 7.33 - 7.26 (m, 2 H), 3.05 - 3.04 (m, 1 H), 2.71 (dd, J = 10.5, 1.1 Hz, 1 H), 2.64 (ddd, J = 13.1, 10.7, 3.7 Hz, 1 H), 2.26 (ddd, J = 10.5, 3.9, 2.2 Hz, 1 H), 2.21 - 2.05 (m, 2 H), 2.00 - 1.93 (m, 1 H) ppm. ^{13}C NMR (100 MHz, $CDCl_3$) δ 176.7, 134.3, 131.0, 130.9, 129.4, 128.4, 127.1, 91.9, 43.5, 43.4, 32.8, 24.8 ppm. The enantiomeric excess was determined by HPLC on Chiralcel OD-H column, hexane/isopropanol = 97/3; flow rate = 1.0 mL/min; UV detection at 220 nm; t_R = 13.8 min (major), t_R = 20.8 min (minor). **HRMS** calculated $[M+Na]^+$ for $C_{12}H_{11}ClNaO_2$ = 245.0340, found: 245.0336.

(1*R*,4*S*)-1-(3,4-dichlorophenyl)-2-oxabicyclo[2.2.1]heptan-3-one (3f)

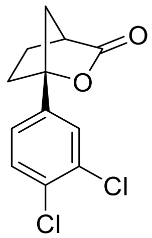

White solid. 42.2 mg, 82% yield, 95% ee, mp = 79 - 82 °C. $[\alpha]^{25}_{D}$ = +35.8 (c = 1.0, $CHCl_3$). ^1H NMR (400 MHz, $CDCl_3$) δ 7.55 (d, J = 2.1 Hz, 1 H), 7.48 (d, J = 8.3 Hz, 1 H), 7.29 (dd, J = 8.4, 2.2 Hz, 1 H), 3.11 - 3.09 (m, 1 H), 2.28 - 2.24 (m, 1 H), 2.20 - 2.14 (m, 3 H), 2.10 (dd, J = 10.3, 1.3 Hz, 1 H), 1.98 - 1.93 (m, 1 H) ppm. ^{13}C NMR (100 MHz, $CDCl_3$) δ 176.8, 137.2, 132.9, 132.6, 130.7, 127.7, 124.8, 90.9, 44.8, 44.0, 34.8, 24.6 ppm. The enantiomeric excess was determined by HPLC on Chiralcel OJ-H column, hexane/isopropanol = 95/5; flow rate = 1.0 mL/min; UV detection at 210 nm; t_R = 29.0 min (major), t_R = 35.1 min (minor). **HRMS** calculated $[M+Na]^+$ for $C_{12}H_{10}Cl_2NaO_2$ = 279.9950, found: 279.9960.

(1*R*,4*S*)-1-(3-methoxyphenyl)-2-oxabicyclo[2.2.1]heptan-3-one (3g)

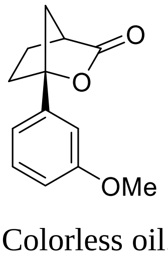

Colorless oil. 39.3 mg, 90% yield, 96% ee, $[\alpha]^{20}_{D}$ = +33.3 (c = 1.0, $CHCl_3$). ^1H NMR (400 MHz, $CDCl_3$) δ 7.33 - 7.26 (m, 1 H), 7.02 - 7.00 (m, 2 H), 6.91 - 6.88 (m, 1 H), 3.83 (s, 3 H), 3.08 - 3.07 (m, 1 H), 2.30 - 2.27 (m, 1 H), 2.20 - 2.11 (m, 3 H), 2.11 -

2.08 (m, 1 H), 1.99 - 1.91 (m, 1 H) ppm. ^{13}C NMR (100 MHz, $CDCl_3$) δ 177.5, 159.7, 138.4, 129.6, 117.4, 113.9, 111.2, 92.3, 55.3, 44.7, 44.0, 34.8, 24.7 ppm. The enantiomeric excess was determined by HPLC on Chiralcel OD-H column, hexane/isopropanol = 97/3; flow rate = 1.0 mL/min; UV detection at 220 nm; t_R = 56.3 min (minor), t_R = 59.1 min (major). **HRMS** calculated $[M+Na]^+$ for $C_{13}H_{14}NaO_3$ = 241.0835, found: 241.0834.

(1*R*,4*S*)-1-(4-(tert-butyl)phenyl)-2-oxabicyclo[2.2.1]heptan-3-one (3h)

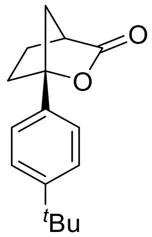

White solid. 39.1 mg, 80% yield, 94% ee, mp = 106 - 107 °C. $[\alpha]^{25}_D$ = +31.5 (*c* = 1.0, $CHCl_3$). ^{1}H NMR (400 MHz, $CDCl_3$) δ 7.44 - 7.38 (m, 4 H), 3.07 - 3.06 (m, 1 H), 2.32 -2.29 (m, 1 H), 2.21 - 2.11 (m, 3 H), 2.07 (dd, *J* = 10.4, 0.9 Hz, 1 H), 1.97 - 1.91 (m, 1 H), 1.33 (s, 9 H) ppm. ^{13}C NMR (100 MHz, $CDCl_3$) δ 177.7, 151.6, 133.8, 125.4, 125.2, 92.4, 44.6, 44.0, 34.6, 34.4, 31.2, 24.7 ppm. The enantiomeric excess was determined by HPLC on Chiralcel OD-H column, hexane/isopropanol = 97/3; flow rate = 1.0 mL/min; UV detection at 220 nm; t_R = 12.5 min (minor), t_R = 13.1 min (major). **HRMS** calculated $[M+H]^+$ for $C_{16}H_{21}O_2$ = 245.1536, found: 245.1535.

(1*R*,4*S*)-1-(3,5-dimethylphenyl)-2-oxabicyclo[2.2.1]heptan-3-one (3i)

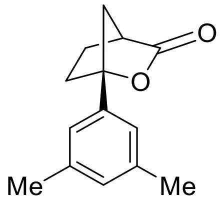

White solid. 31.6 mg, 73% yield, 94% ee, mp = 114 - 116 °C. $[\alpha]^{25}_D$ = +34.3 (*c* = 1.0, $CHCl_3$). ^{1}H NMR (400 MHz, $CDCl_3$) δ 7.07 (s, 2 H), 6.99 (s, 1 H), 3.06 - 3.05 (m, 1 H), 2.34 (s, 6 H), 2.29 - 2.26 (m, 1 H), 2.19 - 2.11 (m, 3 H), 2.09 - 2.06 (m, 1 H), 1.99 - 1.90 (m, 1 H) ppm. ^{13}C NMR (100 MHz, $CDCl_3$) δ 177.7, 138.2, 136.7, 130.1, 123.1, 92.5, 44.6, 44.0, 34.7, 24.8, 21.3 ppm. The enantiomeric excess was determined by HPLC on Chiralpak AD-H column, hexane/isopropanol = 97/3; flow rate = 1.0 mL/min;

UV detection at 210 nm; t_R = 11.0 min (major), t_R = 13.1 min (minor). **HRMS** calculated $[M+Na]^+$ for $C_{14}H_{16}NaO_2$ = 239.1043, found: 239.1039.

(1*R*,4*S*)-1-(m-tolyl)-2-oxabicyclo[2.2.1]heptan-3-one (3j)

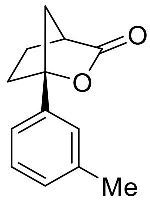

White solid. 33.6 mg, 83% yield, 94% ee, mp = 59 - 61 °C. $[\alpha]^{25}_D$ = +33.6 (*c* = 1.0, $CHCl_3$). ^1H NMR (400 MHz, $CDCl_3$) δ 7.31 - 7.27 (m, 2 H), 7.25 - 7.22 (m, 1 H), 7.18 - 7.16 (m, 1 H), 3.08 - 3.06 (m, 1 H), 2.38 (s, 3 H), 2.31 - 2.28 (m, 1 H), 2.20 - 2.12 (m, 3 H), 2.10 - 2.07 (m, 1 H), 1.97 - 1.91 (m, 1 H) ppm. ^{13}C NMR (100 MHz, $CDCl_3$) δ 177.6, 138.3, 136.8, 129.2, 128.5, 126.1, 122.4, 92.5, 44.6, 44.0, 34.7, 24.8, 21.4 ppm. The enantiomeric excess was determined by HPLC on Chiralcel OD-H column, hexane/isopropanol = 95/5; flow rate = 1.0 mL/min; UV detection at 210 nm; t_R = 14.4 min (major), t_R = 16.5 min (minor). **HRMS** calculated $[M+Na]^+$ for $C_{13}H_{14}NaO_2$ = 225.0886, found: 225.0882.

(1*R*,4*S*)-1-(2-methoxyphenyl)-2-oxabicyclo[2.2.1]heptan-3-one (3k)

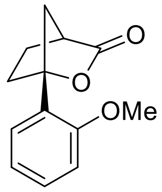

Light yellow oil. 20.5 mg, 47% yield, 91% ee. $[\alpha]^{25}_D$ = +27.9 (*c* = 1.0, $CHCl_3$). ^1H NMR (400 MHz, $CDCl_3$) δ 7.59 - 7.56 (m, 1 H), 7.34 - 7.30 (m, 1 H), 7.01 - 6.97 (m, 1 H), 6.93 - 6.91 (m, 1 H), 3.84 (s, 3 H), 3.00 - 2.99 (m, 1 H), 2.50 - 2.43 (m, 2 H), 2.24 - 2.19 (m, 1 H), 2.15 - 2.07 (m, 1 H), 2.05 - 1.98 (m, 1 H), 1.95 - 1.88 (m, 1 H) ppm. ^{13}C NMR (100 MHz, $CDCl_3$) δ 177.7, 156.4, 129.3, 127.4, 125.1, 120.7, 110.9, 91.7, 55.3, 43.9, 43.6, 33.1, 24.9 ppm. The enantiomeric excess was determined by HPLC on Chiralcel OD-H column, hexane/isopropanol = 95/5; flow rate = 1.0 mL/min; UV detection at 210 nm; t_R = 14.3 min (major), t_R = 21.3 min (minor). **HRMS** calculated $[M+Na]^+$ for $C_{13}H_{14}NaO_3$ = 241.0835, found: 241.0833.

(1*R*,4*S*)-1-(4-(trifluoromethyl)phenyl)-2-oxabicyclo[2.2.1]heptan-3-one (3l)

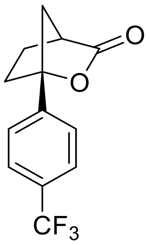

White solid. 39.5 mg, 77% yield, 95% ee, mp = 82 - 84 °C. $[\alpha]^{25}_{D}$ = +33.1 (*c* = 1.0, $CHCl_3$). ^{1}H NMR (400 MHz, $CDCl_3$) δ 7.67 (d, *J* = 8.2 Hz, 2 H), 7.58 (d, *J* = 8.2 Hz, 2 H), 3.13 - 3.12 (m, 1 H), 2.31 - 2.28 (m, 1 H), 2.23 - 2.14 (m, 4 H), 2.03 - 1.94 (m, 1 H) ppm. ^{13}C NMR (100 MHz, $CDCl_3$) δ 176.9, 140.9, 130.61 (q, *J* = 32.6 Hz), 125.7, 125.6 (q, *J* = 3.8 Hz), 123.9 (q, *J* = 272.2 Hz), 91.5, 44.8, 44.0, 35.0, 24.7 ppm. The enantiomeric excess was determined by HPLC on Chiralcel OD-H column, hexane/isopropanol = 98/2; flow rate = 0.5 mL/min; UV detection at 220 nm; t_R = 46.7 min (minor), t_R = 48.4 min (major). **HRMS** calculated $[M+Na]^+$ for $C_{13}H_{11}F_3NaO_2$ = 279.0603, found: 279.0606.

(1*R*,4*S*)-1-([1,1'-biphenyl]-4-yl)-2-oxabicyclo[2.2.1]heptan-3-one (3m)

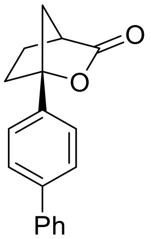

White solid. 37.5 mg, 71% yield, 94% ee, mp = 128 - 130 °C. $[\alpha]^{25}_{D}$ = +28.5 (*c* = 1.0, $CHCl_3$). ^{1}H NMR (400 MHz, $CDCl_3$) δ 7.64 - 7.34 (m, 9 H), 3.10 - 3.09 (m, 1 H), 2.35 - 2.33 (m, 1 H), 2.25 - 2.11 (m, 4 H), 2.00 - 1.94 (m, 1 H) ppm. ^{13}C NMR (100 MHz, $CDCl_3$) δ 177.6, 141.5, 140.4, 135.8, 128.8, 127.5, 127.3, 127.1, 125.9, 92.3, 44.7, 44.0, 34.6, 24.8 ppm. The enantiomeric excess was determined by HPLC on Chiralcel OD-H column, hexane/isopropanol = 95/5; flow rate = 1.0 mL/min; UV detection at 254 nm; t_R = 29.4 min (minor), t_R = 35.3 min (major). **HRMS** calculated $[M+Na]^+$ for $C_{18}H_{16}NaO_2$ = 287.1043, found: 287.1038.

(1*R*,4*S*)-1-(4-(4,4,5,5-tetramethyl-1,3,2-dioxaborolan-2-yl)phenyl)-2-oxabicyclo[2.2.1]heptan-3-one (3n)

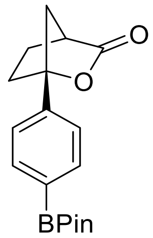

White solid. 49.6 mg, 79% yield, 96% ee, mp = 198 - 202 °C. $[\alpha]^{25}_{D}$ = +26.1 (*c* = 1.0, $CHCl_3$). ^{1}H NMR (400 MHz, $CDCl_3$) δ 7.86 - 7.84 (m, 2 H), 7.47 - 7.45 (m, 2 H), 3.09 - 3.07 (m, 1 H), 2.30 - 2.26 (m, 1 H), 2.21 - 2.10 (m, 4 H), 1.97 - 1.91 (m, 1 H), 1.35 (s, 12 H) ppm. ^{13}C NMR (100 MHz, $CDCl_3$) δ 177.5, 139.9, 135.0, 124.6, 92.4, 84.0, 44.8, 44.1, 34.9, 24.9, 24.9, 24.8 ppm. The enantiomeric excess was determined by HPLC on Chiralcel OD-H column, hexane/isopropanol = 99/1; flow rate = 0.5 mL/min; UV detection at 220 nm; t_R = 26.7 min (minor), t_R = 27.7 min (major). **HRMS** calculated $[M+Na]^+$ for $C_{18}H_{23}BNaO_4$ = 337.1582, found: 337.1573.

(1*R*,4*S*)-1-(naphthalen-2-yl)-2-oxabicyclo[2.2.1]heptan-3-one (3o)

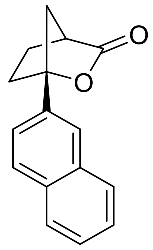

White solid. 42.9 mg, 90% yield, 93% ee, mp = 109 - 111 °C. $[\alpha]^{25}_{D}$ = +19.6 (*c* = 1.0, $CHCl_3$). ^{1}H NMR (400 MHz, $CDCl_3$) δ 7.94 - 7.83 (m, 4 H), 7.56 - 7.49 (m, 3 H), 3.13 - 3.12 (m, 1 H), 2.40 - 2.37 (m, 1 H), 2.31 - 2.16 (m, 4 H), 2.04 - 1.97 (m, 1 H) ppm. ^{13}C NMR (100 MHz, $CDCl_3$) δ 177.6, 134.2, 133.1, 133.0, 128.4, 128.1, 127.7, 126.5, 126.5, 124.5, 123.1, 92.5, 44.8, 44.1, 34.7, 24.8 ppm. The enantiomeric excess was determined by HPLC on Chiralpak AD-H column, hexane/isopropanol = 95/5; flow rate = 0.5 mL/min; UV detection at 220 nm; t_R = 35.9 min (major), t_R = 37.9 min (minor). **HRMS** calculated $[M+Na]^+$ for $C_{16}H_{14}NaO_2$ = 261.0886, found: 261.0880.

(1*R*,4*S*)-1-benzyl-2-oxabicyclo[2.2.1]heptan-3-one (3p)

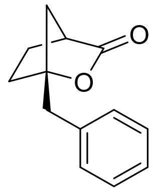

White solid. 30.3 mg, 75% yield, 97% ee, mp = 71- 74 °C. $[\alpha]^{25}_{D}$ = +44.4 (*c* = 1.0, $CHCl_3$). ^{1}H NMR (400 MHz, $CDCl_3$) δ 7.33 - 7.23 (m, 5 H), 3.23 - 3.15 (m, 2 H), 2.85 - 2.84 (m, 1 H), 1.96 - 1.71 (m, 5 H), 1.59 - 1.56 (m, 1 H) ppm. ^{13}C NMR (100 MHz, $CDCl_3$) δ 178.1, 135.8, 129.9, 128.4, 126.9, 92.5, 43.2, 42.8, 39.4, 31.4, 24.4 ppm. The enantiomeric excess was determined by HPLC on Chiralcel OD-H column, hexane/isopropanol = 97/3; flow rate = 1.0 mL/min; UV detection at 210 nm; t_R = 23.7 min (major), t_R = 27.0 min (minor). **HRMS** calculated $[M+Na]^+$ for $C_{13}H_{14}NaO_2$ = 225.0886, found: 225.0883.

(1*R*,4*S*)-1-hexyl-2-oxabicyclo[2.2.1]heptan-3-one (3q)

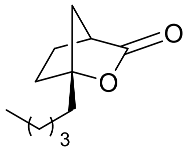

Colorless oil. 27.8 mg, 80% yield, 95% ee, $[\alpha]^{20}_{D}$ = +44.5 (*c* = 1.0, $CHCl_3$). ^{1}H NMR (400 MHz, $CDCl_3$) δ 2.88 - 2.87 (m, 1 H), 1.99 - 1.91 (m, 2 H), 1.88 - 1.78 (m, 5 H), 1.61 - 1.58 (m, 1 H), 1.50 - 1.41 (m, 2 H), 1.36 - 1.31 (m, 4 H), 0.92 - 0.88 (m, 3 H) ppm. ^{13}C NMR (100 MHz, $CDCl_3$) δ 178.5, 93.2, 43.5, 42.9, 32.9, 32.0, 31.6, 24.4, 24.1, 22.4, 13.9 ppm. The enantiomeric excess was determined by GC on *β*-dex225 130 ºC isotherm 10 min, 0.5 ºC/min to 160 ºC, t_R = 46.7 min (minor), t_R = 49.7 min (major). **HRMS** calculated $[M+Na]^+$ for $C_{11}H_{18}NaO_2$ = 205.1199, found: 205.1196.

(1*R*,4*S*)-1-isopropyl-2-oxabicyclo[2.2.1]heptan-3-one (3r)

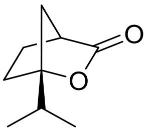

Colorless oil. 27.8 mg, 90% yield, 96% ee, $[\alpha]^{20}_{D}$ = +38.1 (*c* = 1.0, $CHCl_3$). ^{1}H NMR (400 MHz, $CDCl_3$) δ 2.88 - 2.87 (m, 1 H), 2.18 - 2.11 (m, 1 H), 1.99 - 1.91 (m, 2 H), 1.87 - 1.75 (m, 3 H), 1.60 - 1.58 (m, 1 H), 1.04 (dd, *J* = 9.3, 6.9 Hz, 6 H) ppm. ^{13}C NMR (100 MHz, $CDCl_3$) δ 178.5, 96.5, 43.4, 41.0, 30.6, 28.5, 24.3, 17.8, 17.5 ppm. The enantiomeric excess was determined by GC on *β*-dex225 130 ºC isotherm 10 min, 0.5 ºC/min to 160 ºC, t_R = 19.6 min (minor), t_R = 20.8 min (major). **HRMS** calculated $[M+Na]^+$ for $C_9H_{14}NaO_2$ = 177.0886, found: 177.0882.

(1*R*,4*S*)-1-cyclopropyl-2-oxabicyclo[2.2.1]heptan-3-one (3s)

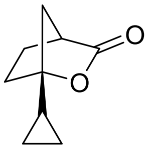

Colorless oil. 18.3 mg, 60% yield, 91% ee, $[\alpha]^{20}_{D}$ = +36.1 (*c* = 1.0, $CHCl_3$). ^{1}H NMR (400 MHz, $CDCl_3$) δ 2.88 - 2.87 (m, 1 H), 1.99 - 1.90 (m, 1 H), 1.87 - 1.78 (m, 4 H), 1.54 - 1.51 (m, 1 H), 1.33 - 1.24 (m, 2 H), 0.67 - 0.59 (m, 2 H), 0.53 - 0.45 (m, 2 H) ppm. ^{13}C NMR (100 MHz, $CDCl_3$) δ 178.1, 93.9, 43.3, 41.8, 31.3, 24.2, 12.5, 2.3, 1.9 ppm. The enantiomeric excess was determined by GC on *β*-dex225 130 ºC isotherm 10 min, 0.5 ºC/min to 160 ºC, t_R = 31.9 min (minor), t_R = 33.8 min (major). **HRMS** calculated $[M+Na]^+$ for $C_9H_{12}NaO_2$ = 175.0730, found: 175.0724.

(1*R*,4*S*)-1-cyclopentyl-2-oxabicyclo[2.2.1]heptan-3-one (3t)

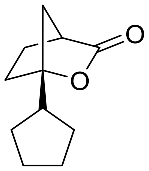

Colorless oil. 31.4 mg, 87% yield, 96% ee, $[\alpha]^{20}_{D}$ = +33.6 (*c* = 1.0, $CHCl_3$). ^{1}H NMR (400 MHz, $CDCl_3$) δ 2.87 - 2.86 (m, 1 H), 2.31 (p, *J* = 8.6 Hz, 1 H), 2.00 - 1.91 (m, 2 H), 1.85 - 1.74 (m, 5 H), 1.69 - 1.55 (m, 5 H), 1.52 - 1.43 (m, 2 H) ppm. ^{13}C NMR (100 MHz, $CDCl_3$) δ 178.7, 95.2, 43.4, 42.1, 41.7, 30.6, 27.8, 27.8, 25.7, 25.7, 24.6 ppm. The enantiomeric excess was determined by GC on *β*-dex225 130 ºC isotherm 10 min, 0.5 ºC/min to 160 ºC, t_R = 53.4 min (minor), t_R = 55.4 min (major). **HRMS** calculated $[M+Na]^+$ for $C_{11}H_{16}NaO_2$ = 203.1043, found: 203.1039.

(1*R*,4*S*)-1-cyclohexyl-2-oxabicyclo[2.2.1]heptan-3-one (3u)

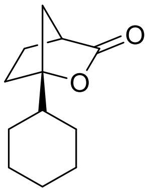

Colorless oil. 34.6 mg, 89% yield, 96% ee, $[\alpha]^{20}_{D}$ = +34.8 (*c* = 1.0, $CHCl_3$). ^{1}H NMR (400 MHz, $CDCl_3$) δ 2.87 - 2.86 (m, 1 H), 1.97 - 1.69 (m, 11 H), 1.58 - 1.55 (m, 1 H), 1.32 - 1.08 (m, 5 H) ppm. ^{13}C NMR (100 MHz, $CDCl_3$) δ 178.5, 96.2, 43.3, 41.0, 40.4, 28.8, 28.1, 27.6, 26.1, 26.1, 26.0, 24.2 ppm. The enantiomeric excess was determined

by GC on β-dex225 130 ºC isotherm 10 min, 0.5 ºC/min to 160 ºC, t_R = 71.3 min (minor), t_R = 73.0 min (major). **HRMS** calculated $[M+Na]^+$ for $C_{12}H_{18}NaO_2$ = 217.1199, found: 217.1198.

(1*R*,4*S*)-1-((3R,5R,7R)-adamantan-1-yl)-2-oxabicyclo[2.2.1]heptan-3-one (3v)

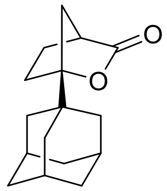

White solid. 43.4 mg, 88% yield, mp = 158 - 160 ℃. $[\alpha]^{25}_D$ = +25.1 (*c* = 1.0, $CHCl_3$). ^1H NMR (400 MHz, $CDCl_3$) δ 2.87 - 2.86 (m, 1 H), 2.05 - 2.01 (m, 4 H), 1.98 - 1.83 (m, 2 H), 1.81 - 1.66 (m, 13 H), 1.58 (s, 1 H), 1.49 (m, 1 H) ppm. ^{13}C NMR (100 MHz, $CDCl_3$) δ 178.6, 99.1, 43.3, 37.9, 37.3, 36.9, 34.5, 28.1, 26.5, 24.2 ppm. **HRMS** calculated $[M+Na]^+$ for $C_{16}H_{22}NaO_2$ = 269.1512, found: 269.1509.

(1*S*,3*R*)-3-hydroxy-3-(trifluoromethyl)cyclopentane-1-carbaldehyde (2w)

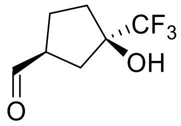

Light yellow oil. 28.4 mg, 78% yield, 93% ee. $[\alpha]^{25}_D$ = - 4.9 (*c* = 1.0, $CHCl_3$). ^1H NMR (400 MHz, $CDCl_3$) δ 9.71 (d, *J* = 1.1 Hz, 1 H),3.03 - 2.96 (m, 1 H), 2.68 (brs, 1 H), 2.28 - 2.18 (m, 3 H), 2.17 - 2.03 (m, 2 H), 1.91 - 1.83 (m, 1 H) ppm. ^{13}C NMR (100 MHz, $CDCl_3$) δ 203.0, 125.7 (q, *J* = 282.2 Hz), 81.3 (q, *J* = 29.8 Hz), 49.5, 34.7, 33.9, 24.0 ppm. The enantiomeric excess was determined by GC on β-dex225 120 ºC isotherm 10 min, 0.5 ºC/min to 140 ºC, t_R = 37.5 min (major), t_R = 39.8 min (minor).

(1*S*,5*R*)-5-phenyl-6-oxabicyclo[3.2.2]nonan-7-one (3x)

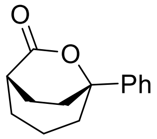

Colorless oil. 34.6 mg, 80% yield, 85% ee, $[\alpha]^{25}_D$ = -56.2 (*c* = 1.0, $CHCl_3$). ^1H NMR (400 MHz, $CDCl_3$) δ 7.47 - 7.45 (m, 2 H), 7.36 - 7.32 (m, 2 H), 7.27 - 7.23 (m, 1 H), 2.94 - 2.90 (m, 1 H), 2.51 - 2.41 (m, 1 H), 2.20 - 2.14 (m, 1 H), 2.12 - 2.05 (m, 3 H), 2.04 - 1.77 (m, 5 H) ppm. ^{13}C NMR (100 MHz, $CDCl_3$) δ 176.3, 146.4, 128.4, 127.1,

123.9, 84.5, 41.0, 38.2, 30.9, 28.1, 21.4, 21.2 ppm. The enantiomeric excess was determined by HPLC on Chiralpak AD-H column, hexane/isopropanol = 97/3; flow rate = 1.0 mL/min; UV detection at 210 nm; t_R = 26.3 min (minor), t_R = 29.2 min (major). **HRMS** calculated $[M+Na]^+$ for $C_{14}H_{16}NaO_2$ = 239.1043, found: 239.1045.

General procedure for asymmetric hydroformylation of 4

In a glovebox filled with argon, to a 5 mL vial equipped with a magnetic bar was added (*S*,*R*)-DM-YanPhos (0.004 mmol) and $Rh(acac)(CO)_2$ (0.002 mmol in 1 mL toluene). After stirring for 10 minutes, the mixture was charged to substrate (0.2 mmol). The vial was transferred into an autoclave and taken out of the glovebox. The argon gas was replacement with hydrogen gas for three times, and then hydrogen (2.5 bar) and carbon monoxide (2.5 bar) were charged in sequence. The reaction mixture was stirred at 80 °C (oil bath) for 48 h. The reaction was cooled to room temperature and the pressure was carefully released in a well-ventilated hood. The solution was concentrated and the product was isolated by column chromatography. The enantiomeric excesses were determined by GC analysis or by HPLC analysis.

NMR, optical rotation and HRMS Data of 5

The enantiomeric excesses of **5** were determined HPLC after Wittig reductions with methyl (triphenylphosphoranylidene)acetate. Compounds **5** were isolated by column chromatography (AcOEt/hexane 1:30 to 1:10).

methyl (3*S*)-3-formyl-1-phenylcyclopentane-1-carboxylate (5a)

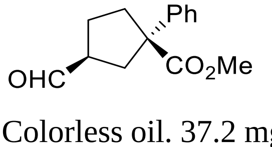

Colorless oil. 37.2 mg, 80% yield, 94% ee, $[\alpha]^{25}_D$ = +16.4 (*c* = 0.5, CH_3OH). ^1H NMR (400 MHz, $CDCl_3$) δ 9.74 (d, *J* = 1.2 Hz, 1 H), 4.33 - 4.22 (m, 2 H), 3.22 - 3.14 (m, 1 H), 2.27 - 2.18 (m, 3 H), 2.15 - 2.06 (m, 3 H), 2.05 - 1.95 (m, 1 H), 1.75 - 1.69 (m, 1 H) ppm. ^{13}C NMR (100 MHz, $CDCl_3$) δ 202.1, 181.6, 65.6, 51.0, 48.6, 36.0, 35.6, 35.0, 25.8 ppm. The enantiomeric excess was determined by HPLC on Chiralpak AS-H column, hexane/isopropanol = 99/1; flow rate = 1.0 mL/min; UV detection at 220 nm; t_R = 15.0 min (minor), t_R = 16.2 min (major). **HRMS** calculated $[M+Na]^+$ for $C_{14}H_{16}NaO_3$ = 255.0992, found: 255.0993.

(7*S*)-1-oxo-2-oxaspiro[4.4]nonane-7-carbaldehyde (5b)

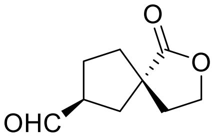

Colorless oil. 27.2 mg, 81% yield, 94% ee, $[\alpha]^{25}_D$ = +13.4 (*c* = 1.0, CH_3OH). ^{1} H NMR (400 MHz, $CDCl_3$) δ 9.74 (d, *J* = 1.2 Hz, 1 H), 4.33 - 4.22 (m, 2 H), 3.22 - 3.14 (m, 1 H), 2.27 - 2.18 (m, 3 H), 2.15 - 2.06 (m, 3 H), 2.05 - 1.95 (m, 1 H), 1.75 - 1.69 (m, 1 H) ppm. ^{13}C NMR (100 MHz, $CDCl_3$) δ 202.1, 181.6, 65.6, 51.0, 48.6, 36.0, 35.6, 35.0, 25.8 ppm. The enantiomeric excess was determined by HPLC on Chiralcel OJ-H column, hexane/isopropanol = 85/15; flow rate = 1.0 mL/min; UV detection at 220 nm; t_R = 29.0 min (major), t_R = 33.7 min (minor). **HRMS** calculated $[M+Na]^+$ for $C_9H_{12}NaO_3$ = 191.0679, found: 191.0679.

(2*S*)-6-oxo-7-oxaspiro[4.5]decane-2-carbaldehyde (5c)

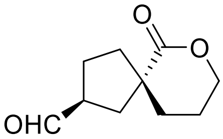

Colorless oil. 31.3 mg, 86% yield, 87% ee, $[\alpha]^{25}_D$ = +7.6 (*c* = 1.0, CH_3OH). ^{1}H NMR (400 MHz, $CDCl_3$) δ 9.72 (d, *J* = 1.3 Hz, 1 H), 4.39 - 4.35 (m, 2 H), 3.23 - 1.14 (m, 1 H), 2.41 - 2.36 (m, 1 H), 2.28 - 2.16 (m, 2 H), 2.00 - 1.81 (m, 6 H), 1.62 - 1.55 (m, 1 H) ppm. ^{13}C NMR (100 MHz, $CDCl_3$) δ 202.7, 176.6, 70.1, 51.6, 49.4, 38.3, 37.9, 33.4, 25.9, 21.4 ppm. The enantiomeric excess was determined by HPLC on Chiralpak AD-H column, hexane/isopropanol = 85/15; flow rate = 1.0 mL/min; UV detection at 220 nm; t_R = 9.6 min (minor), t_R = 10.8 min (major). **HRMS** calculated $[M+Na]^+$ for $C_{10}H_{14}NaO_3$ = 205.0835, found: 205.0833.

Procedure for gram-scale asymmetric hydroformylation of 1d

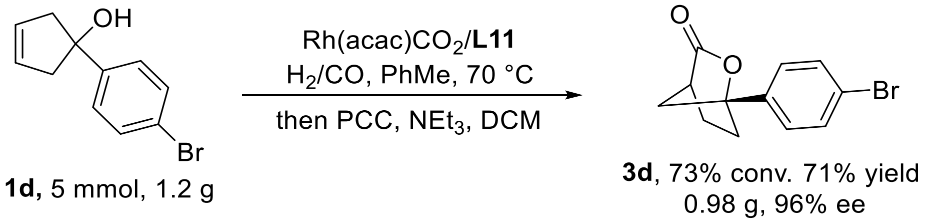

1d, 5 mmol, 1.2 g

3d, 73% conv. 71% yield
0.98 g, 96% ee

In a glovebox filled with argon, to a 20 ml vial equipped with a magnetic bar was added (*S*,*R*)-DM-YanPhos (0.02 mmol) and $Rh(acac)(CO)_2$ (0.01 mmol in 6 mL toluene). After stirring for 10 minutes, the mixture was charged to **1d** (5 mmol). The vial was transferred into an autoclave and taken out of the glovebox. The argon gas was replacement with hydrogen gas for three times, and then hydrogen (3 bar) and carbon monoxide (3 bar) were charged in sequence. The reaction mixture was stirred at 70 °C (oil bath) for 72 h. The reaction was cooled to room temperature and the pressure was carefully released in a well-ventilated hood. The solution was transferred into a solution of pyridinium chlorochromate (PCC) (10 mmol) and triethylamine (2.5 mmol) in 5 mL dichloromethane, the reaction mixture was stirred at 25 °C (oil bath) overnight. The solution was concentrated and the product was isolated by column chromatography (TLC, petroleum ether/ethyl acetate = 10/1). **3d** was isolated with 0.98 g (71% yield, 96% ee).

Procedure for the synthesis of compound 6

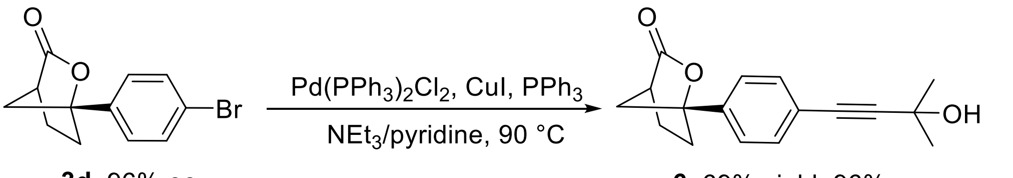

3d, 96% ee　　**6**, 69% yield, 96% ee

To a flame dryed tube **3d** (0.2 mmol), PPh_3 (2.6 mg), CuI (1 mg) and $Pd(PPh_3)_2Cl_2$ (3 mg) were added under nitrogen atmosphere, 2-methyl-but-3-yn-2-ol (0.3 mmol), pyridine (0.5 mL) and NEt_3 (1mL) were transferred into the tube through syringes continuously. And the mixture was stirred at 90 ℃ for 3 hours. After the reaction cooled to room temperature, 1 M HCl was added dropwise to quench the reaction, followed by extraction with dichloromethane three times, dried with anhydrous sodium sulfate. The organic solvent was removed under reduced pressure, and the product was isolated by column chromatography (TLC, petroleum ether/ethyl acetate = 3/1). **6** was isolated with 37.0 mg (69% yield, 96% ee). ^{1}H NMR (400 MHz, $CDCl_3$) δ 7.37 - 7.28 (m, 4 H), 3.01 - 3.00 (m, 1 H), 2.39 (brs, 1 H), 2.21 - 2.17 (m, 1 H), 2.11 - 2.04 (m, 3 H), 2.02 - 1.99 (m, 1 H), 1.89 - 1.83 (m, 1 H), 1.54 (s, 6 H) ppm. ^{13}C NMR (100 MHz, $CDCl_3$) δ 177.4, 136.7, 131.7, 125.3, 123.0, 94.6, 92.0, 81.4, 65.5, 44.6, 43.9, 34.6, 31.4, 24.6 ppm. The enantiomeric excess was determined by HPLC on Chiralcel OD-H column, hexane/isopropanol = 92/8; flow rate = 1.0 mL/min; UV detection at 220 nm; t_R = 30.7

min (minor), t_R = 32.1 min (major). **HRMS** calculated $[M+Na]^+$ for $C_{17}H_{19}O_3$ = 271.1329, found: 271.1330.

Procedure for the synthesis of compound 7

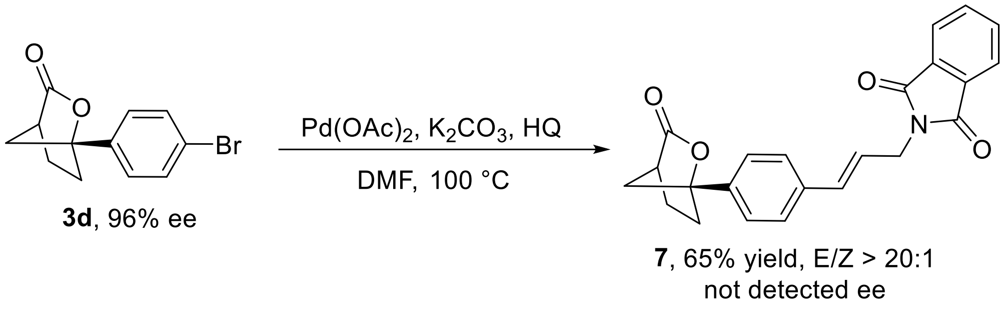

To a flame dryed tube **3d** (0.2 mmol), 3-phthalimido-1-propene (0.24 mmol) $Pd(OAc)_2$ (1.4 mg), K_2CO_3 (33.2 mg) and 1,4-dihydroxybenzol (2.5 mg) were added under nitrogen atmosphere. DMF (1mL) was transferred into the tube through syringe. And the mixture was stirred at 100 °C for 4 hours. After the reaction cooled to room temperature, 10 mL dichloromethane was added to the mixture, and followed by elution with water (5 mL) for three times. Organic phase was dried with anhydrous sodium sulfate. The organic solvent was removed under reduced pressure, and the product was isolated by column chromatography (TLC, petroleum ether/ethyl acetate = 3/1). **7** was isolated with 48.6 mg (65% yield). ^{1}H NMR (400 MHz, $CDCl_3$) δ 7.89 - 7.83 (m, 2 H), 7.75 - 7.70 (m, 2 H), 7.37 (s, 4 H), 6.68 - 6.63 (m, 1 H), 6.32 - 6.25 (m, 1 H), 4.46 - 4.44 (m, 2 H), 3.07 - 3.06 (m, 1 H), 2.29 - 2.25 (m, 1 H), 2.18 - 2.11 (m, 3 H), 2.08 - 2.05 (m, 1 H), 1.98 - 1.90 (m, 1 H) ppm. ^{13}C NMR (100 MHz, $CDCl_3$) δ 177.5, 167.9, 136.5, 136.3, 134.0, 132.9, 132.1, 126.6, 125.7, 123.6, 123.3, 92.2, 44.6, 44.0, 39.5, 34.5, 24.7 ppm. **HRMS** calculated $[M+Na]^+$ for $C_{23}H_{20}NO_4$ = 374.1387, found: 374.1391.

Procedure for the synthesis of compound 8

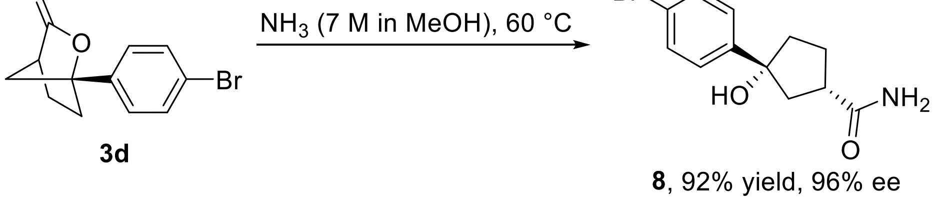

Compound **8** was prepared according to the literature.[5] The optical pure **3d** (0.2 mmol) was combined with ammonia (7.0 M in methanol) (3.0 mL) in a 10 mL sealed tube to

give a clear solution. The reaction mixture was stirred at 60 °C for 12 hours and then concentrated in vacuo followed by column chromatography (TLC, petroleum ether/ethyl acetate = 1/1) to give the desired product **8** as a white solid (51.8 mg, 92% yield, 96% ee). ^{1}H NMR (400 MHz, CD_3OD) δ 7.48 - 7.42 (m, 4 H), 3.09 - 3.02 (m, 1 H), 2.28 - 2.20 (m, 2 H), 2.18 - 2.02 (m, 4 H) ppm. ^{13}C NMR (100 MHz, DMSO-d_6) δ 182.7, 146.4, 131.9, 128.3, 121.3, 83.1, 46.1, 44.6, 42.9, 30.2 ppm. The enantiomeric excess was determined by HPLC on Chiralcel OD-H column, hexane/isopropanol = 80/20; flow rate = 1.0 mL/min; UV detection at 220 nm; t_R = 9.5 min (minor), t_R = 10.4 min (major). **HRMS** calculated $[M+Na]^+$ for $C_{12}H_{14}BrNNaO_2$ = 306.0100, found: 306.0096.

Procedure for the synthesis of compound 9

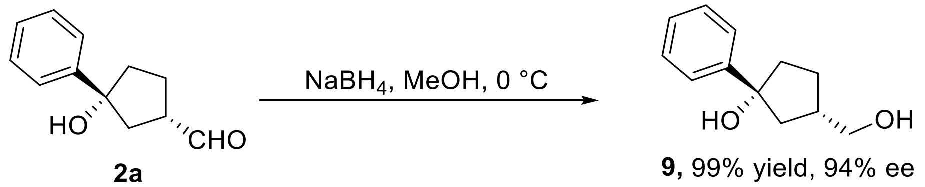

2a → **9**, 99% yield, 94% ee

After the AHF reaction of **1a** under the optimal reaction condition, the system was transformed into a solution of $NaBH_4$ (20 mg) in 1 mL MeOH at 0 °C, and the mixture was stirred for 1 hour at 0 °C. Water was added to quench the reaction followed extraction with ethyl acetate (3 mL) for three times, and the organic phase was dried with Na_2SO_4. The solution was concentrated and the product was isolated by column chromatography (TLC, petroleum ether/ethyl acetate = 5/1). **9** was isolated with 38.5 mg (99% yield, 94% ee). White solid. $[\alpha]^{25}_{D}$ = +12.8 (*c* = 1.0, CH_3OH). ^{1}H NMR (400 MHz, $CDCl_3$) δ 7.50 - 7.47 (m, 2 H), 7.35 - 7.30 (m, 2 H), 7.27 - 7.22 (m, 1 H), 3.66 (qd, *J* = 10.2, 3.9 Hz, 2 H), 3.52 - 3.44 (m, 1 H), 2.57 - 2.47 (m, 1 H), 2.25 (dd, *J* = 14.1, 11.2 Hz, 1 H), 2.11 - 1.87 (m, 5 H) ppm. ^{13}C NMR (100 MHz, $CDCl_3$) δ 145.8, 128.1, 126.8, 125.2, 82.3, 65.7, 44.8, 42.0, 39.1, 26.2 ppm. The enantiomeric excess was determined by GC on *β*-dex225 120 °C isotherm 10 min, 0.5 °C/min to 140 °C, t_R = 32.9 min (major), t_R = 33.7 min (minor). **HRMS** calculated $[M+Na]^+$ for $C_{12}H_{16}NaO_2$ = 215.1043, found: 215.1042.

Procedure for the synthesis of compound 10

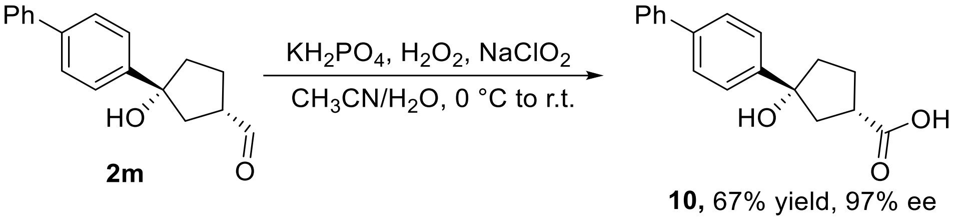

Compound **10** was prepared according to the literature.[6] The aldehyde **2m** (0.2 mmol), CH_3CN (0.5 mL), H_2O (0.5 mL), NaH_2PO_4(4 equiv, 0.8 mmol), and 30% H_2O_2(4 equiv, 0.8 mmol) were combined in a round-bottom flask in this order. The reaction flask was cooled to 0 °C and $NaClO_2$ (4 equiv, 0.8 mmol) was added dropwise as a solution in H_2O (0.5 mL). The solution was stirred vigorously for 3 h, while allowing it to warm up to room temperature. After reaction is complete, sodium sulfite is added for quenching, followed by 1 M HCl (2 mL), and the aqueous mixture is extracted with dichloromethane (3 mL × 5). The solution was concentrated and the product was isolated by column chromatography (TLC, petroleum ether/ethyl acetate/AcOH = 300/100/1). **10** was isolated with 40.1 mg (67% yield, >20:1 dr). Light yellow solid. $[\alpha]^{25}_{D}$ = +36.2 (*c* = 1.0, CH_3OH). ^1H NMR (400 MHz, DMSO-d_6) δ 7.66 - 7.63 (m, 2 H), 7.61 - 7.58 (m, 2 H), 7.56 - 7.53 (m, 2 H), 7.47 - 7.43 (m, 2 H), 7.37 - 7.32 (m, 1 H), 3.03 - 2.95 (m, 1 H), 2.26 - 2.09 (m, 3 H), 2.02 - 1.96 (m, 1 H), 1.95 - 1.90 (m, 1 H) ppm. ^{13}C NMR (100 MHz, DMSO-d_6) δ 176.9, 147.2, 140.2, 138.2, 129.0, 127.3, 126.6, 126.2, 125.9, 81.0, 44.7, 42.5, 42.0, 27.7 ppm. The ee value was detected after a methylation reaction with (Trimethylsilyl)diazomethane.[7] To a solution of **10** in MeOH, $TMSCHN_2$ (2.0 M in hexanes) was slowly added, and the resulting mixture was stirred for another 15 min and quenched by 1-2 drops of acetic acid. The solvent was evaporated in vacuo and the residue was purified by flash column chromatography affording target esters. The enantiomeric excess of was determined by HPLC on Chiralpak IC-H column, hexane/isopropanol = 90/10; flow rate = 1.0 mL/min; UV detection at 220 nm; t_R = 19.7 min (major), t_R = 20.8 min (minor).

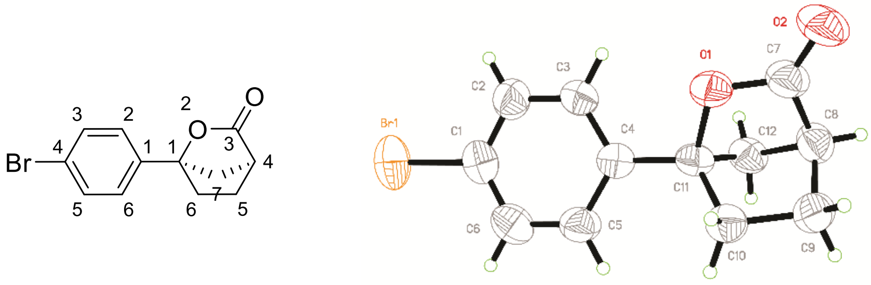

Supplementary Figure 2. X-ray crystallography of 3d

The structure and absolute configuration of **3d** was determined by X-ray. The crystal was prepared from the solution of **3d** in dichloromethane by slow evaporation of the solvent at room temperature. CCDC 2034549 (**3d**) contain the crystallographic data in this paper. These data can be obtained free of charge from The Cambridge Crystallographic Data Centre via www.ccdc.cam.ac.uk/data_request/cif.

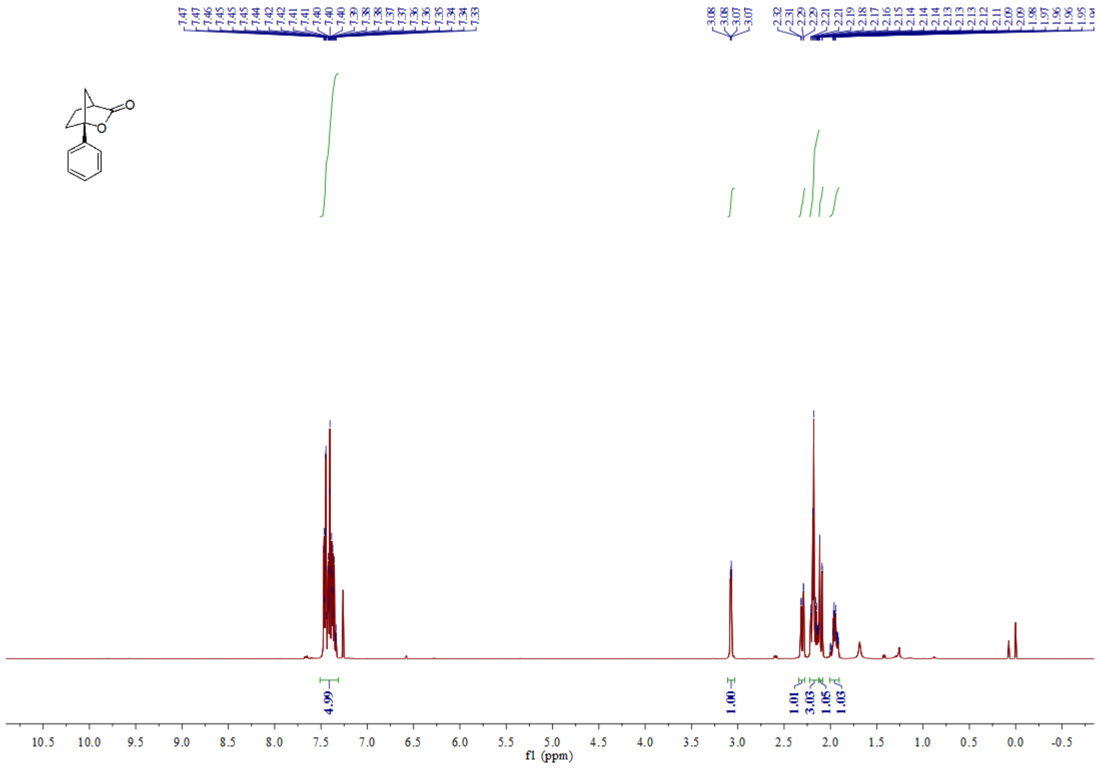

Supplementary Figure 3. ^{1}H NMR (400 MHz, $CDCl_3$) spectra for compound 3a

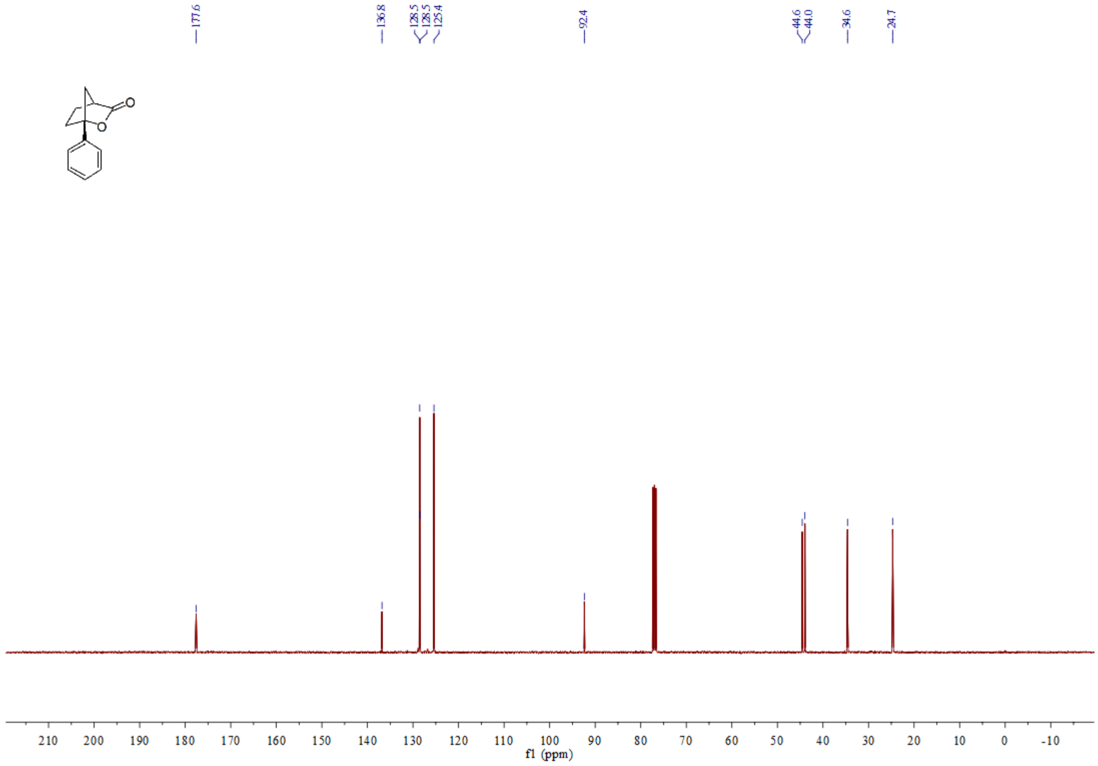

Supplementary Figure 4. ^{13}C NMR (100 MHz, $CDCl_3$) spectra for compound 3a

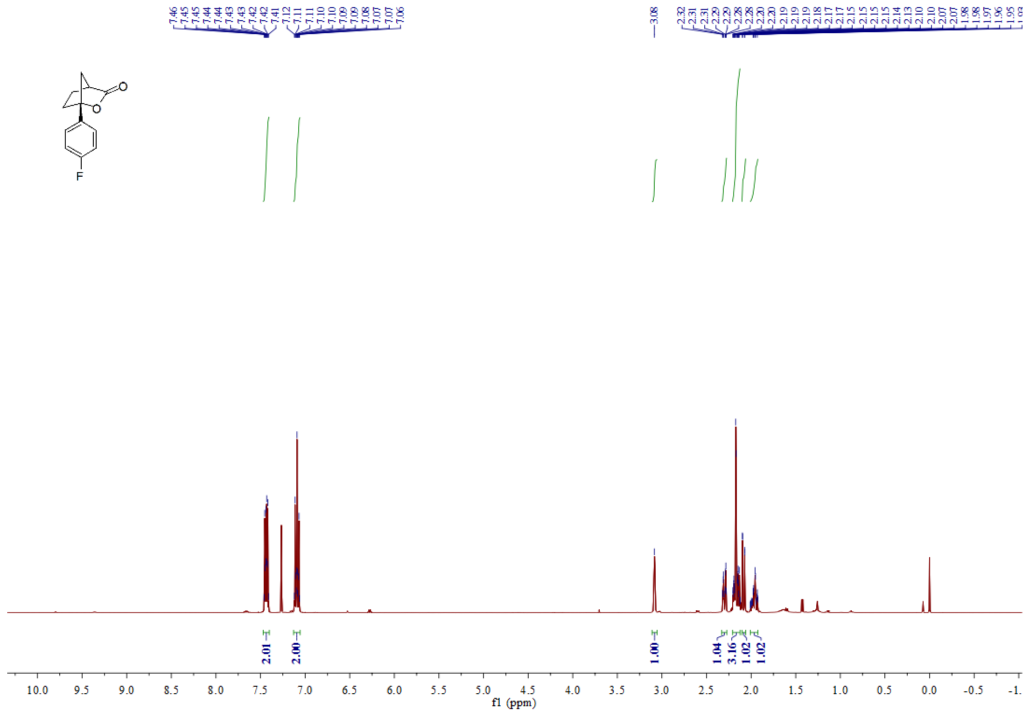

Supplementary Figure 5. ^{1}H NMR (400 MHz, $CDCl_3$) spectra for compound 3b

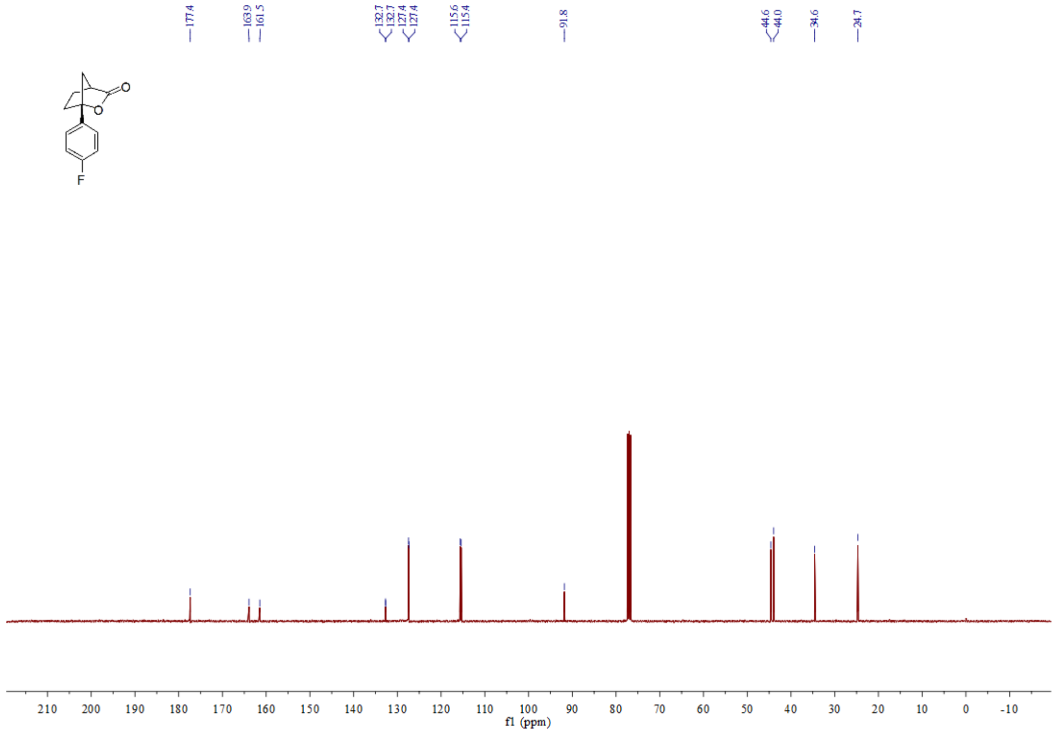

Supplementary Figure 6. ^{13}C NMR (100 MHz, $CDCl_3$) spectra for compound 3b

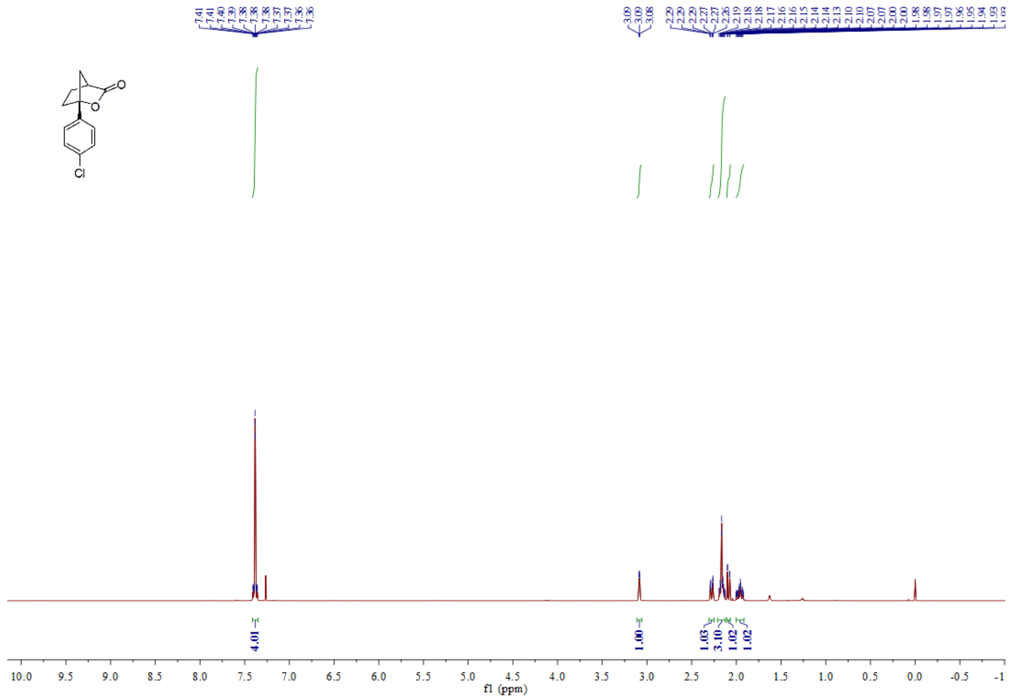

Supplementary Figure 7. ¹H NMR (400 MHz, CDCl3) spectra for compound 3c

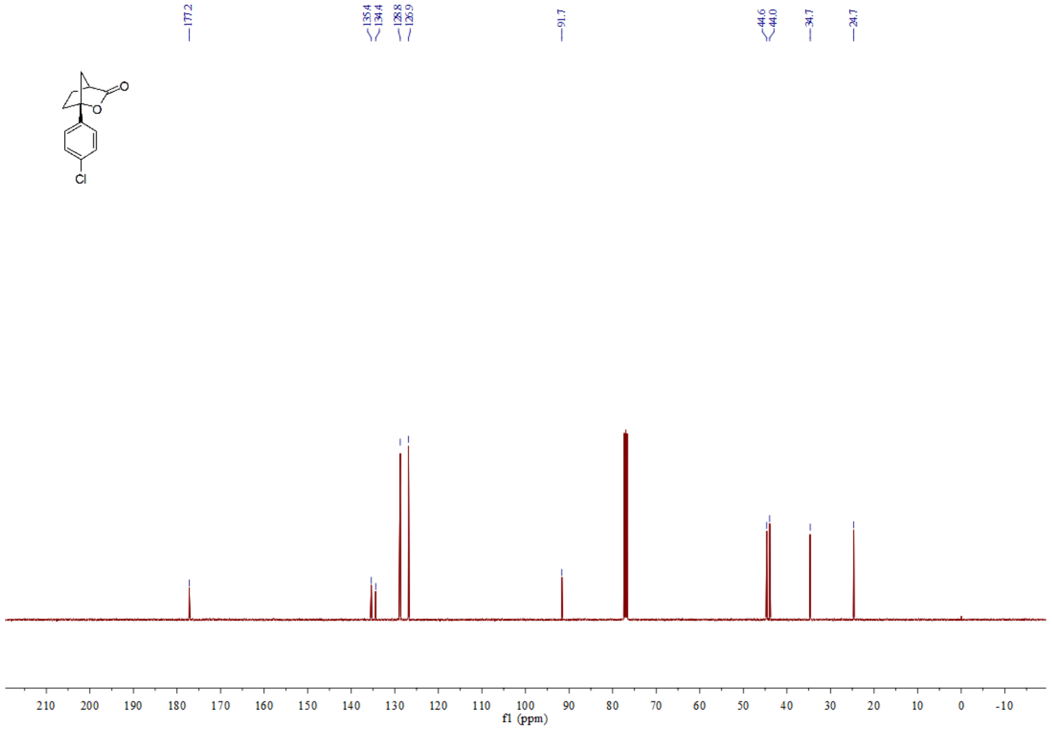

Supplementary Figure 8. ¹³C NMR (100 MHz, CDCl3) spectra for compound 3c

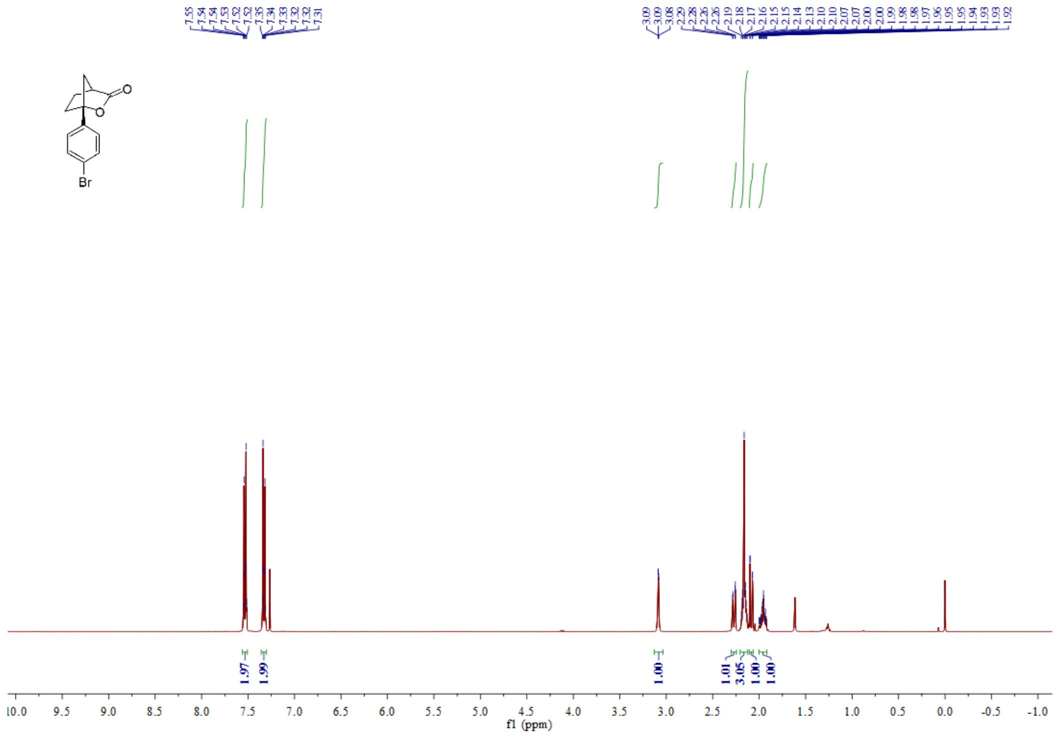

Supplementary Figure 9. ^{1}H NMR (400 MHz, CDCl3) spectra for compound 3d

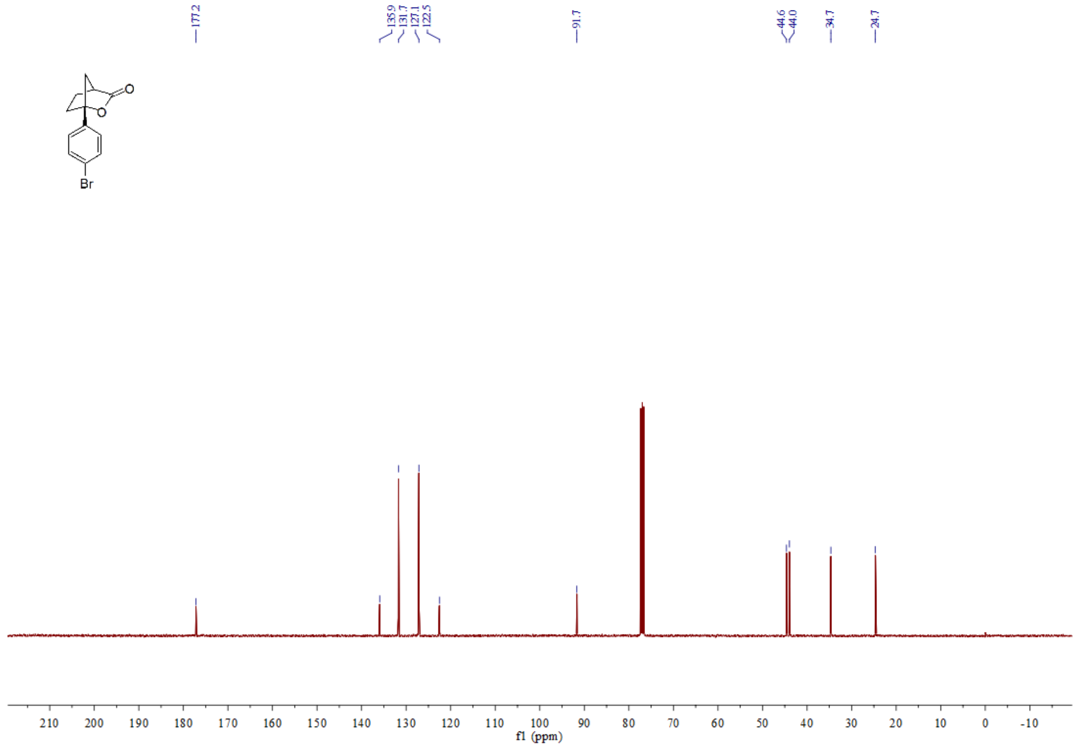

Supplementary Figure 10. ^{13}C NMR (100 MHz, CDCl3) spectra for compound 3d

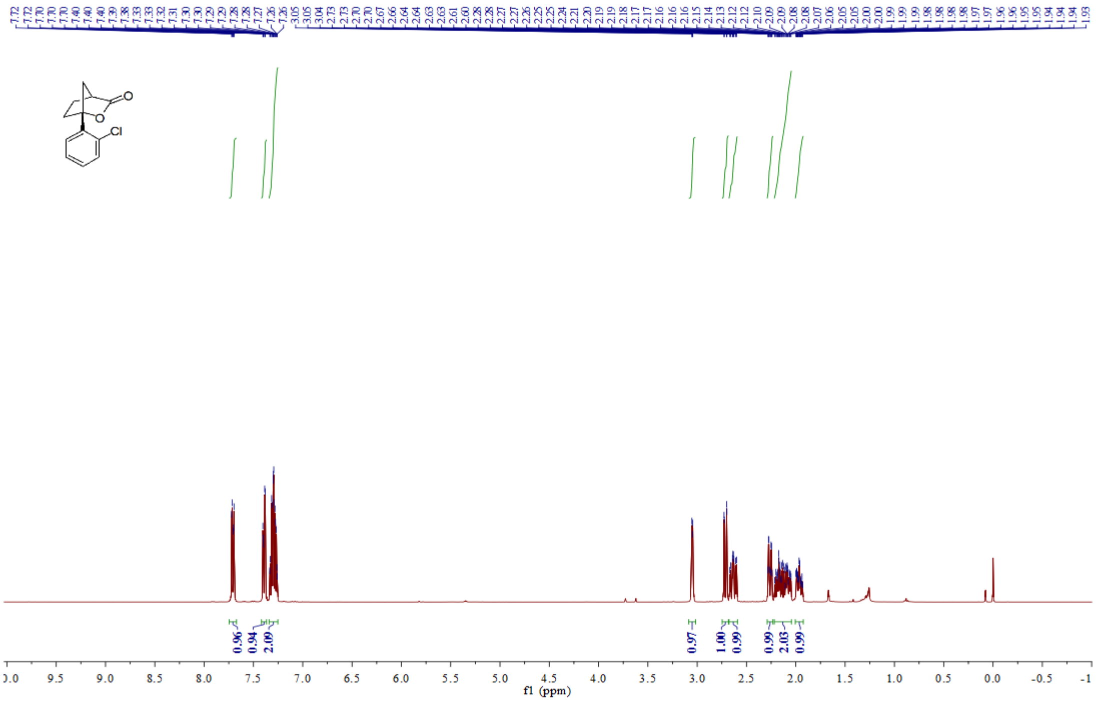

Supplementary Figure 11. ^{1}H NMR (400 MHz, $CDCl_3$) spectra for compound 3e

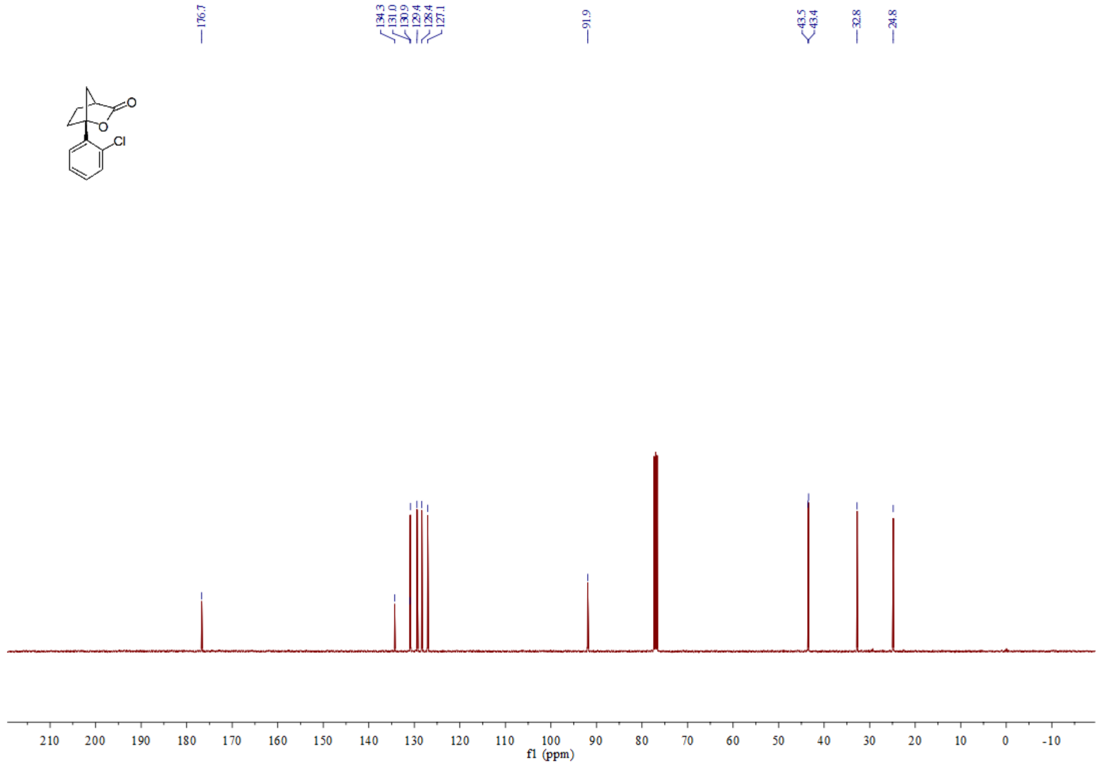

Supplementary Figure 12. ^{13}C NMR (100 MHz, $CDCl_3$) spectra for compound 3e

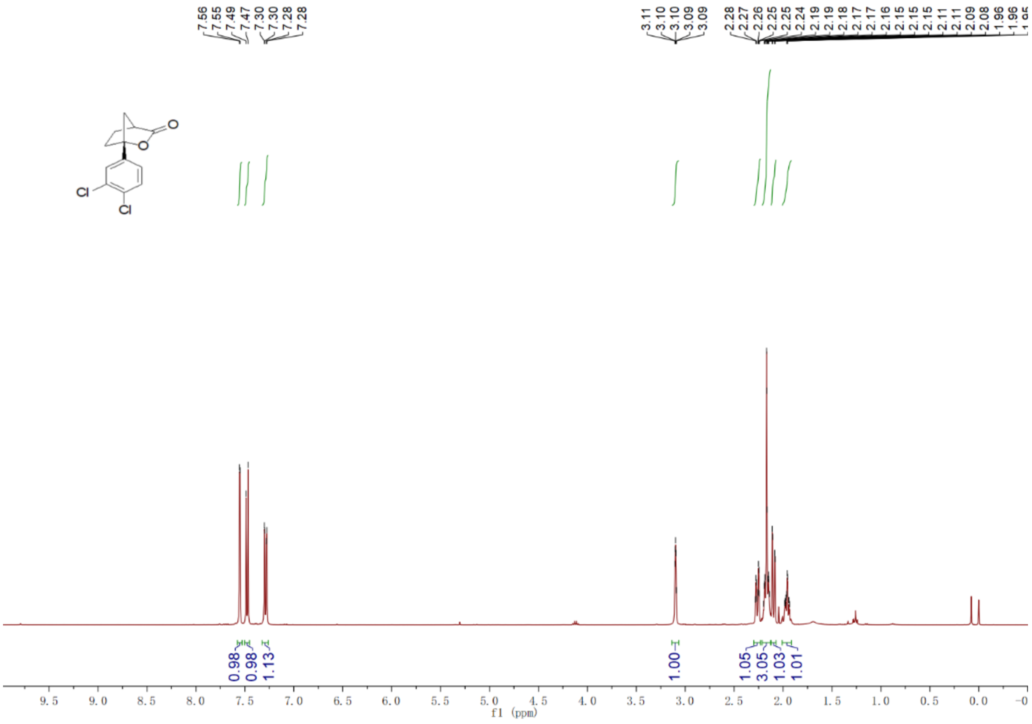

Supplementary Figure 13. ^{1}H NMR (400 MHz, CDCl3) spectra for compound 3f

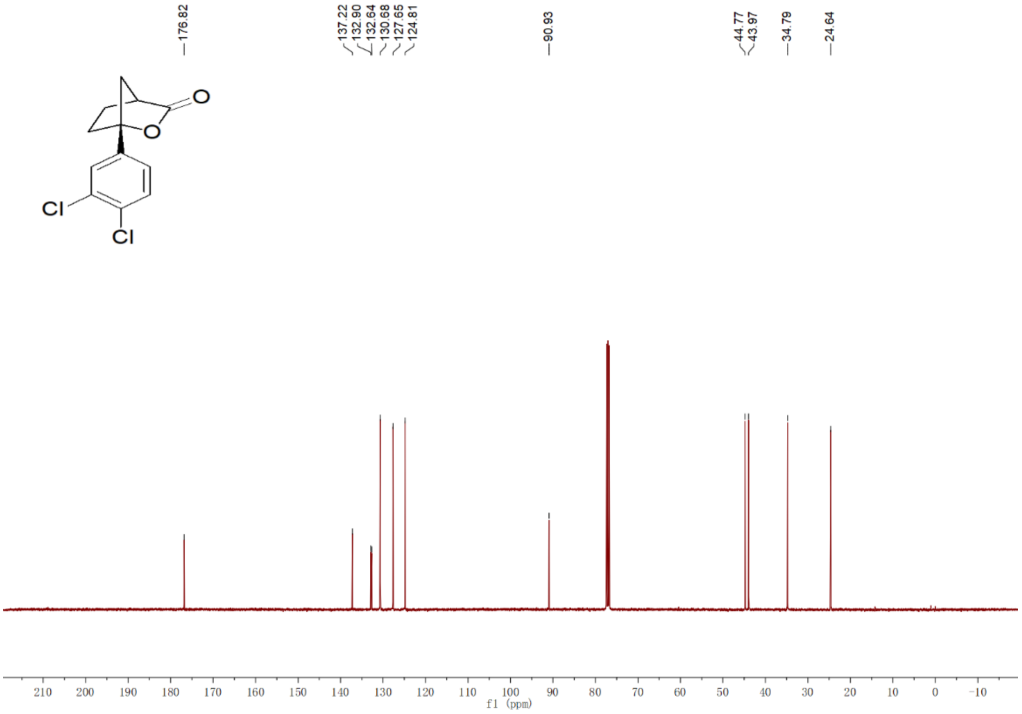

Supplementary Figure 14. ^{13}C NMR (100 MHz, CDCl3) spectra for compound 3f

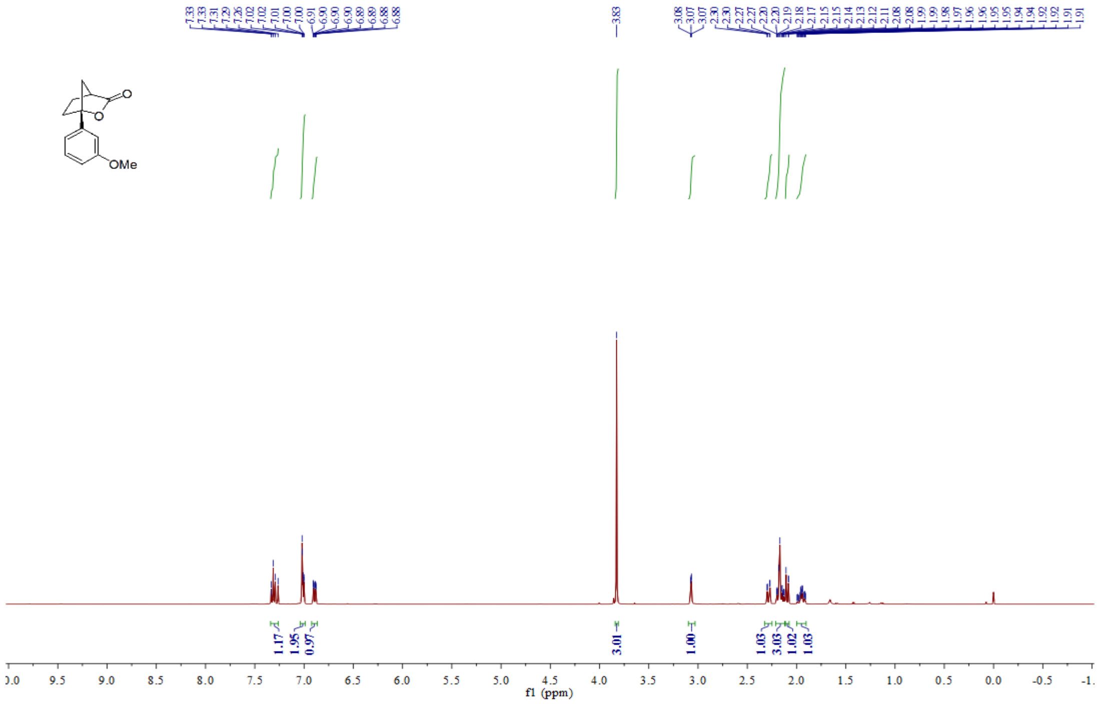

Supplementary Figure 15. ^{1}H NMR (400 MHz, CDCl$_3$) spectra for compound 3g

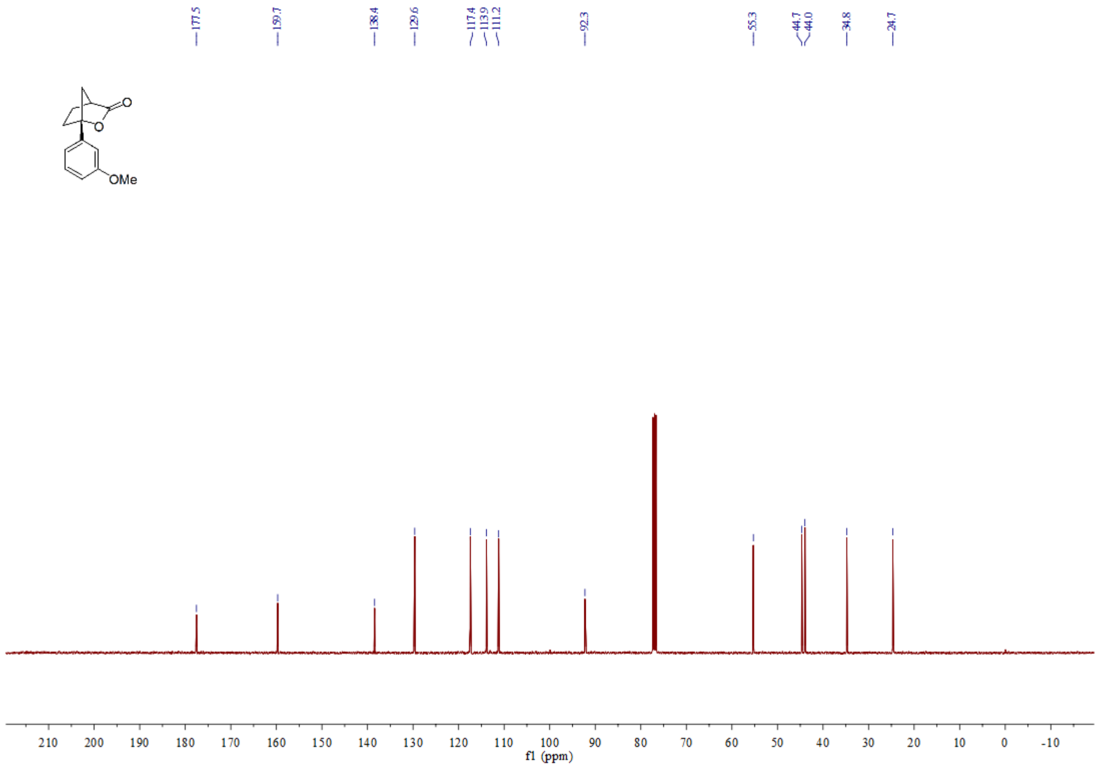

Supplementary Figure 16. ^{13}C NMR (100 MHz, CDCl$_3$) spectra for compound 3g

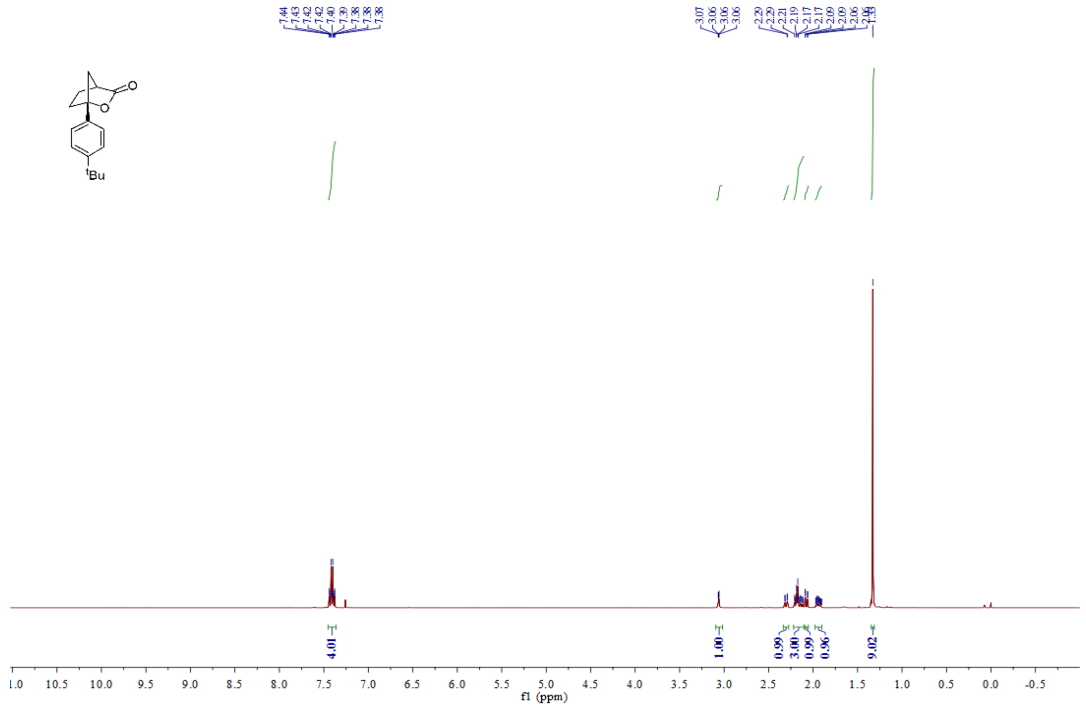

Supplementary Figure 17. ^{1}H NMR (400 MHz, $CDCl_3$) spectra for compound 3h

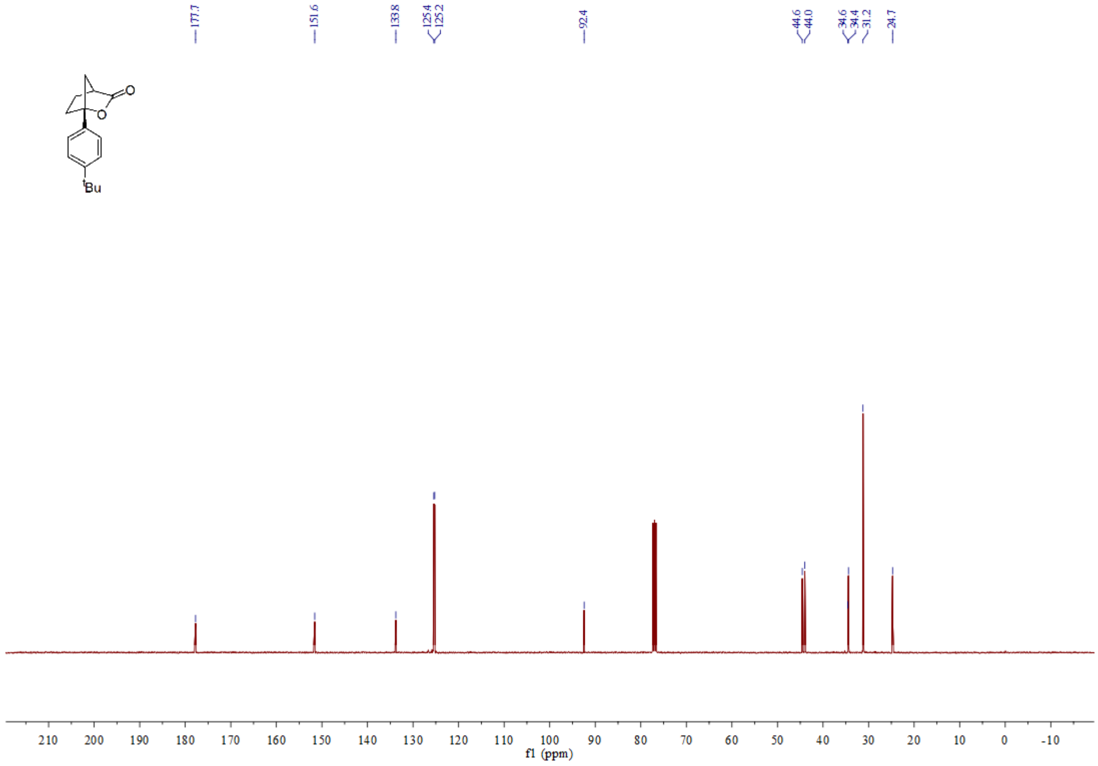

Supplementary Figure 18. ^{13}C NMR (100 MHz, $CDCl_3$) spectra for compound 3h

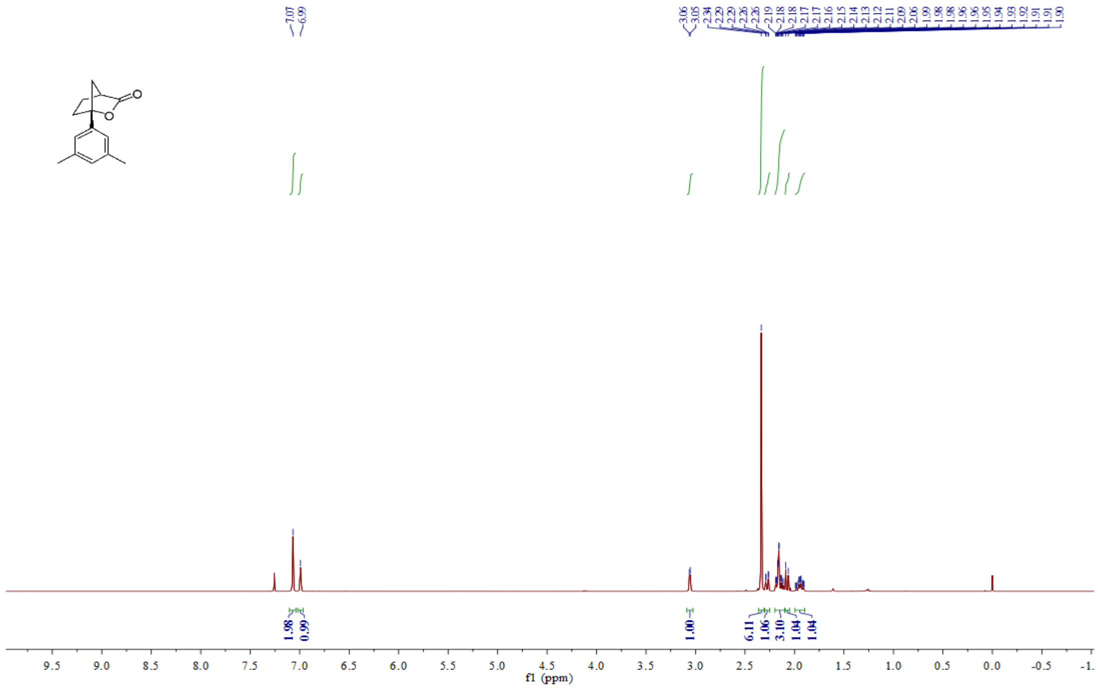

Supplementary Figure 19. ^{1}H NMR (400 MHz, $CDCl_3$) spectra for compound 3i

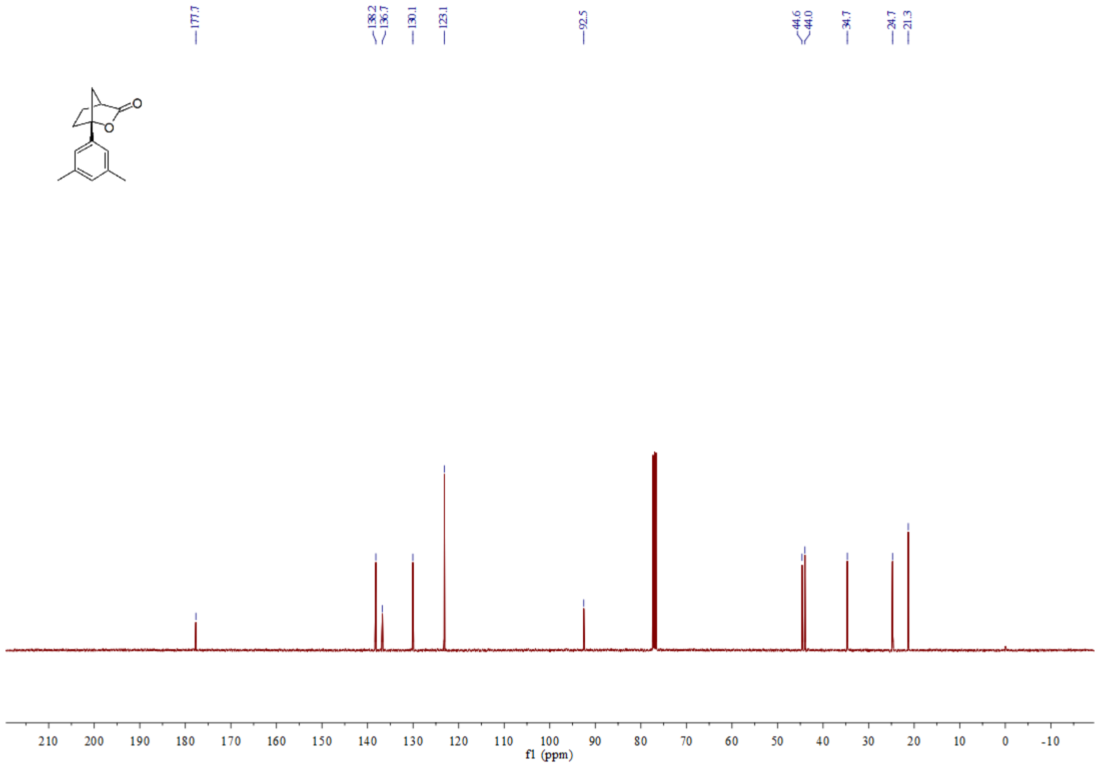

Supplementary Figure 20. ^{13}C NMR (100 MHz, $CDCl_3$) spectra for compound 3i

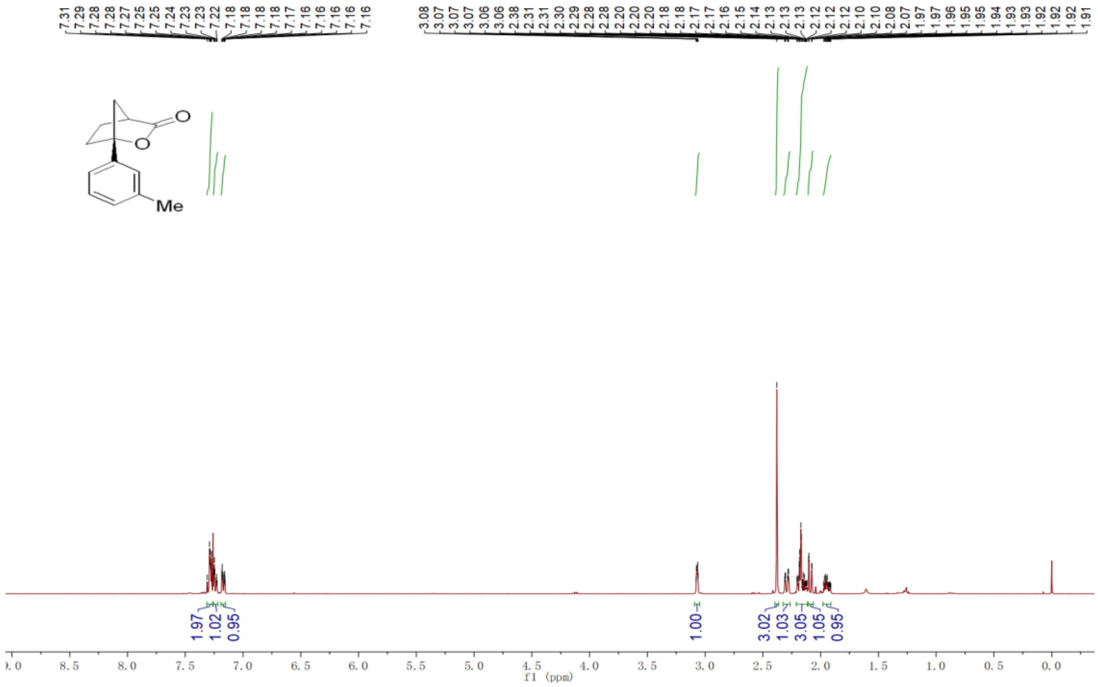

Supplementary Figure 21. ^{1}H NMR (400 MHz, $CDCl_3$) spectra for compound 3j

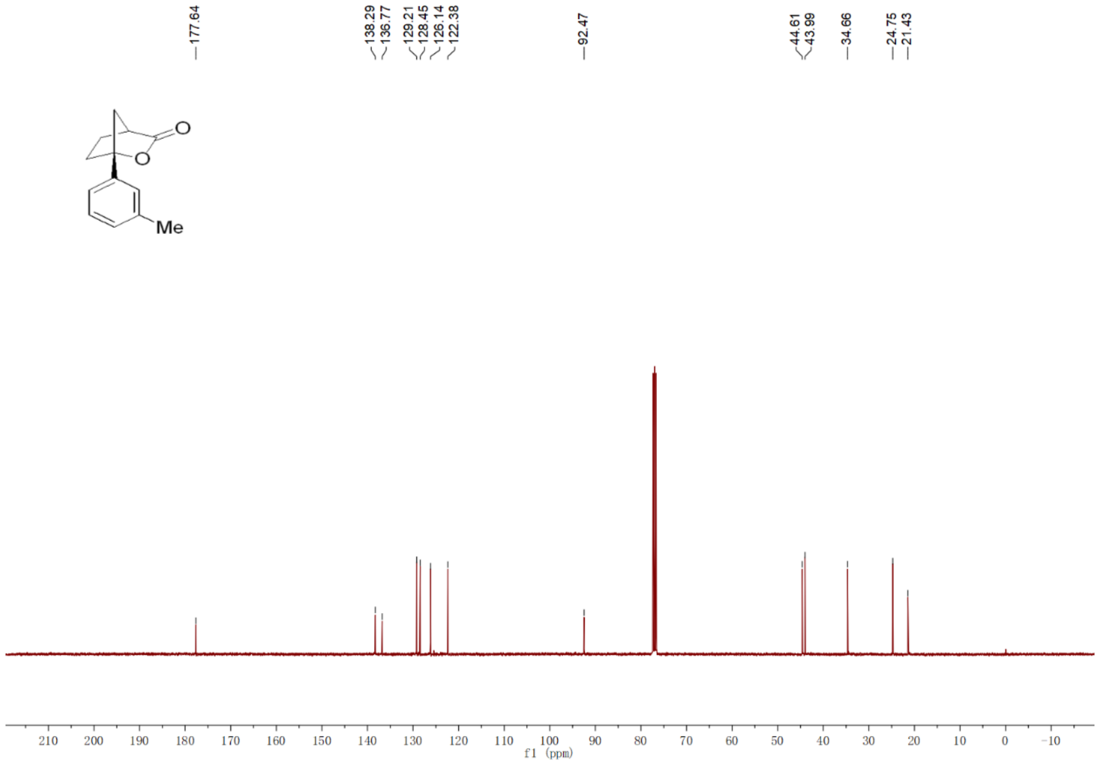

Supplementary Figure 22. ^{13}C NMR (100 MHz, $CDCl_3$) spectra for compound 3j

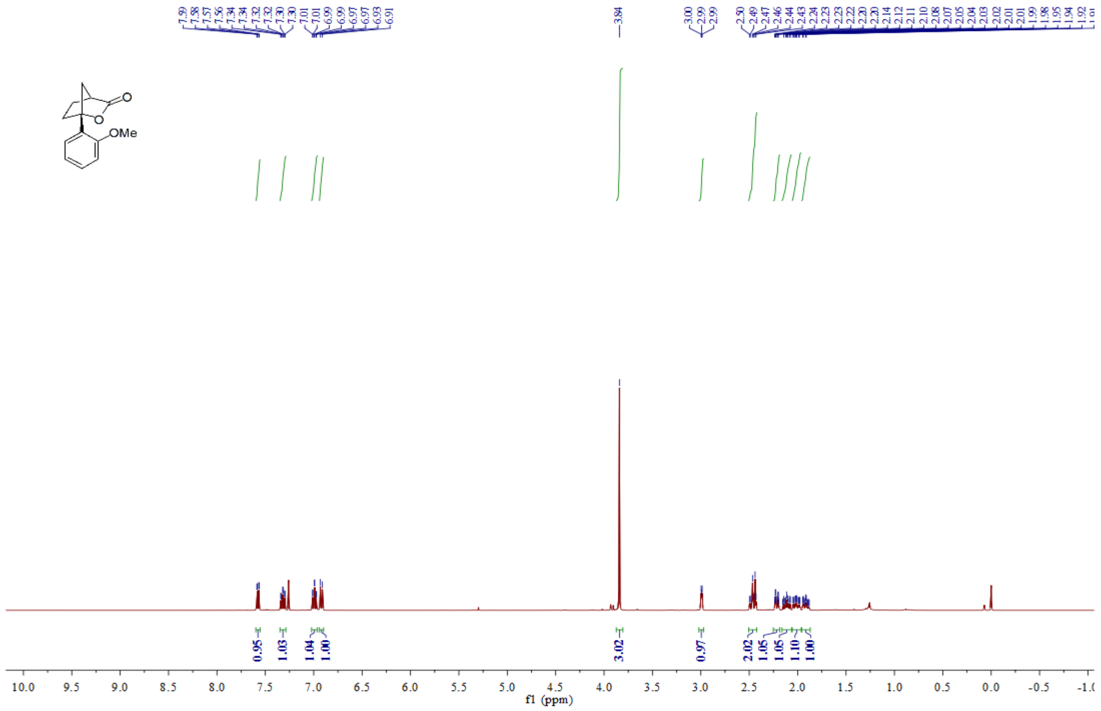

Supplementary Figure 23. ^{1}H NMR (400 MHz, CDCl3) spectra for compound 3k

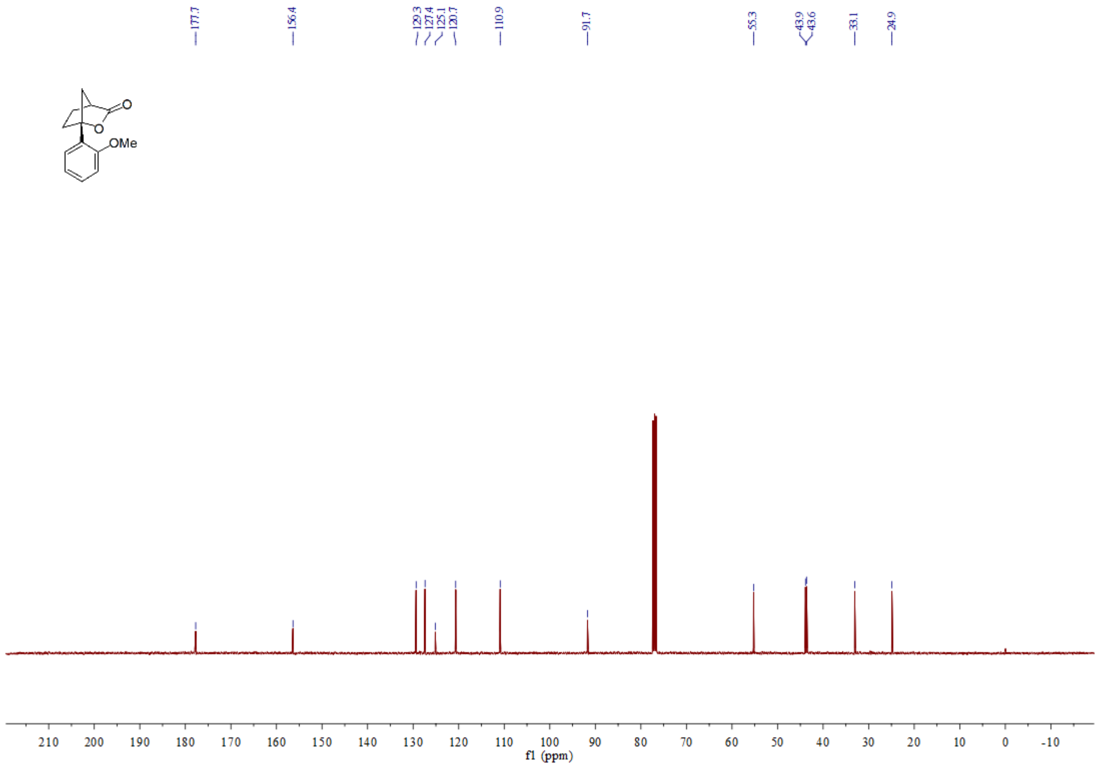

Supplementary Figure 24. ^{13}C NMR (100 MHz, CDCl3) spectra for compound 3k

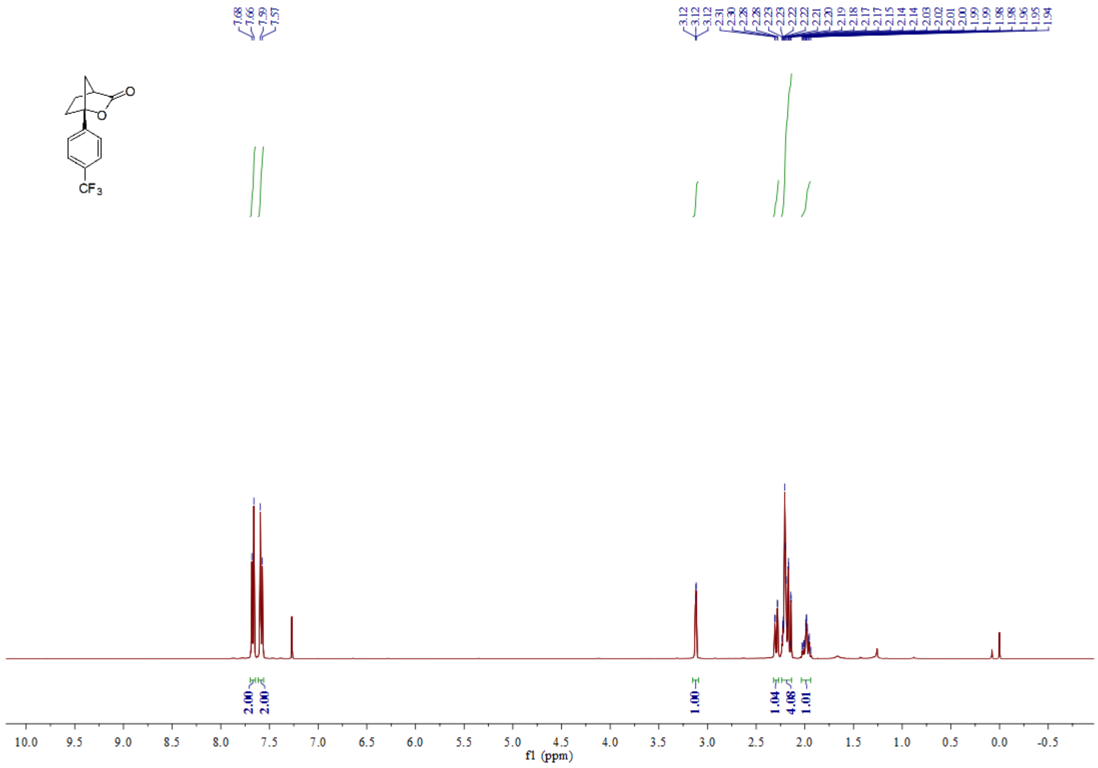

Supplementary Figure 25. ^{1}H NMR (400 MHz, $CDCl_3$) spectra for compound 3l

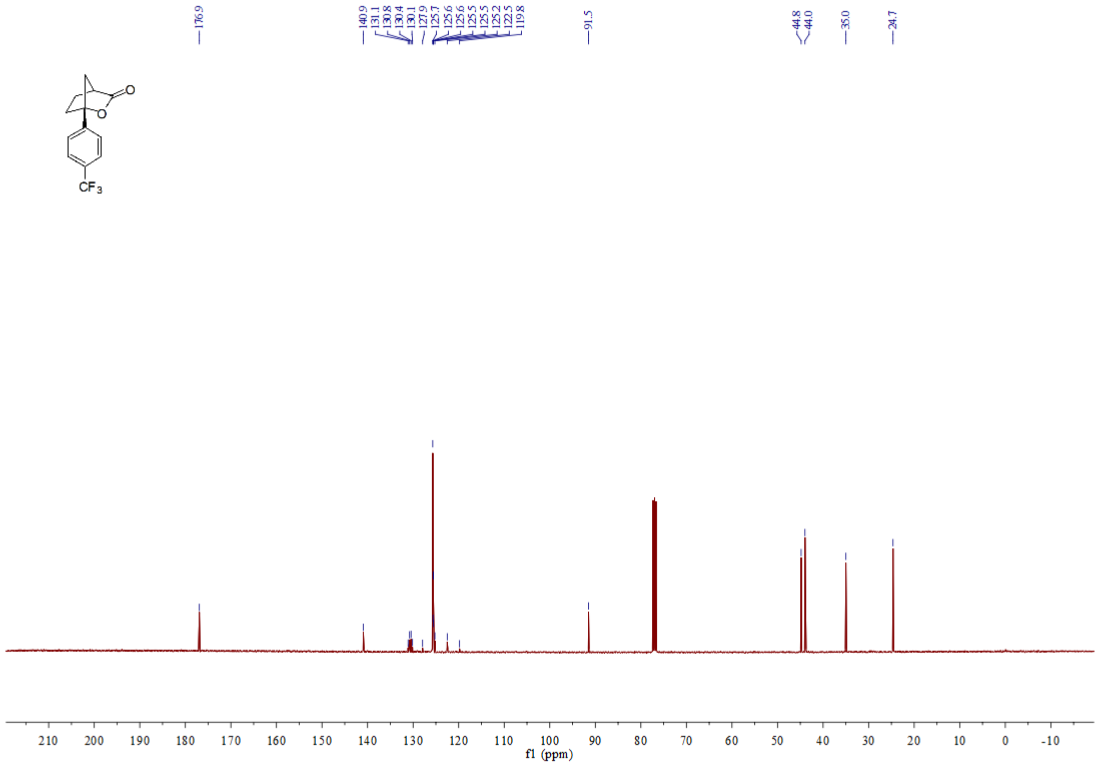

Supplementary Figure 26. ^{13}C NMR (100 MHz, $CDCl_3$) spectra for compound 3l

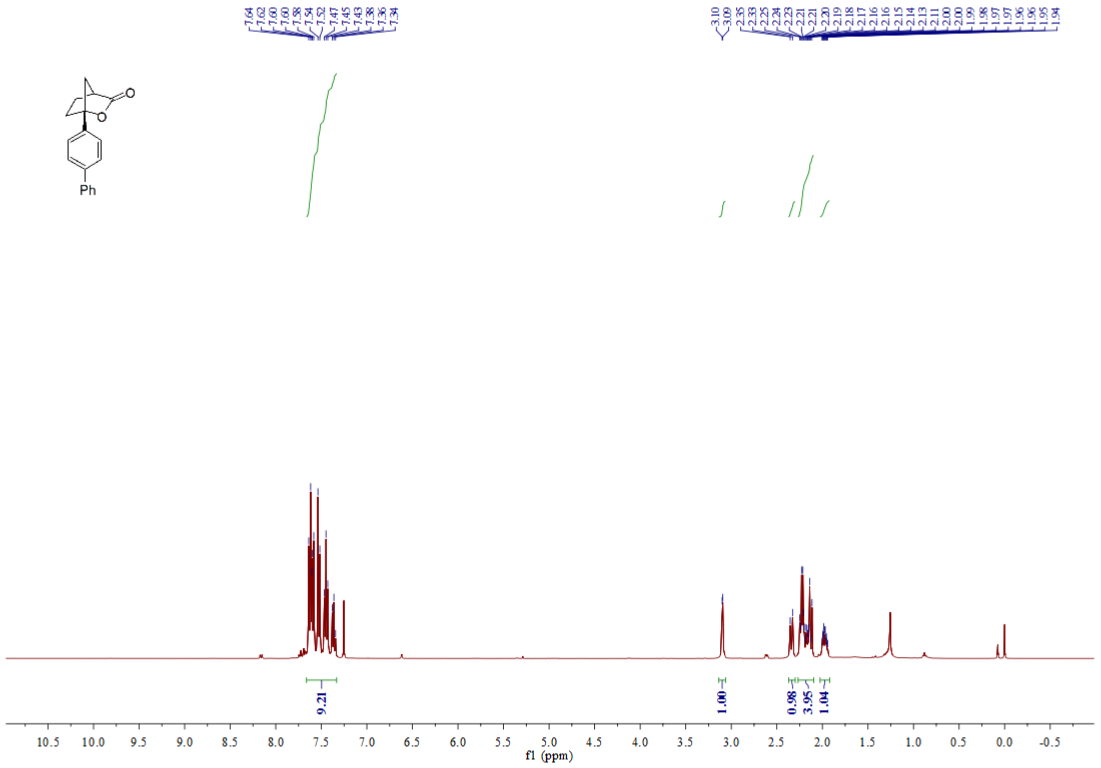

Supplementary Figure 27. ^{1}H NMR (400 MHz, $CDCl_3$) spectra for compound 3m

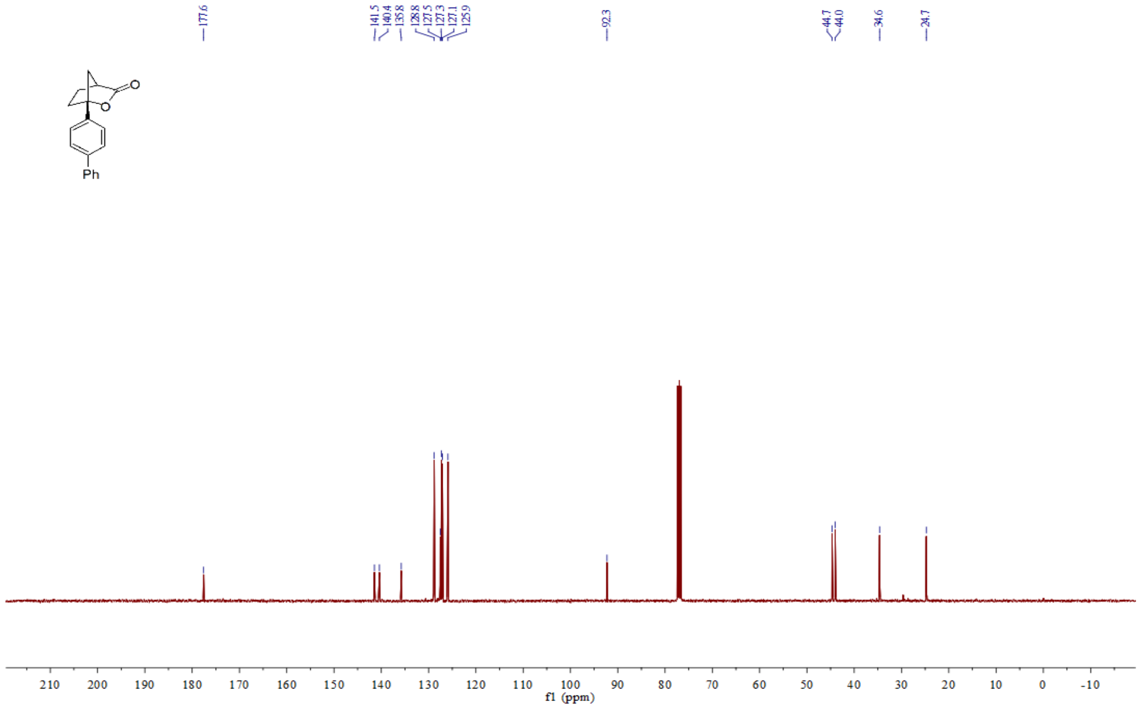

Supplementary Figure 28. ^{13}C NMR (100 MHz, $CDCl_3$) spectra for compound 3m

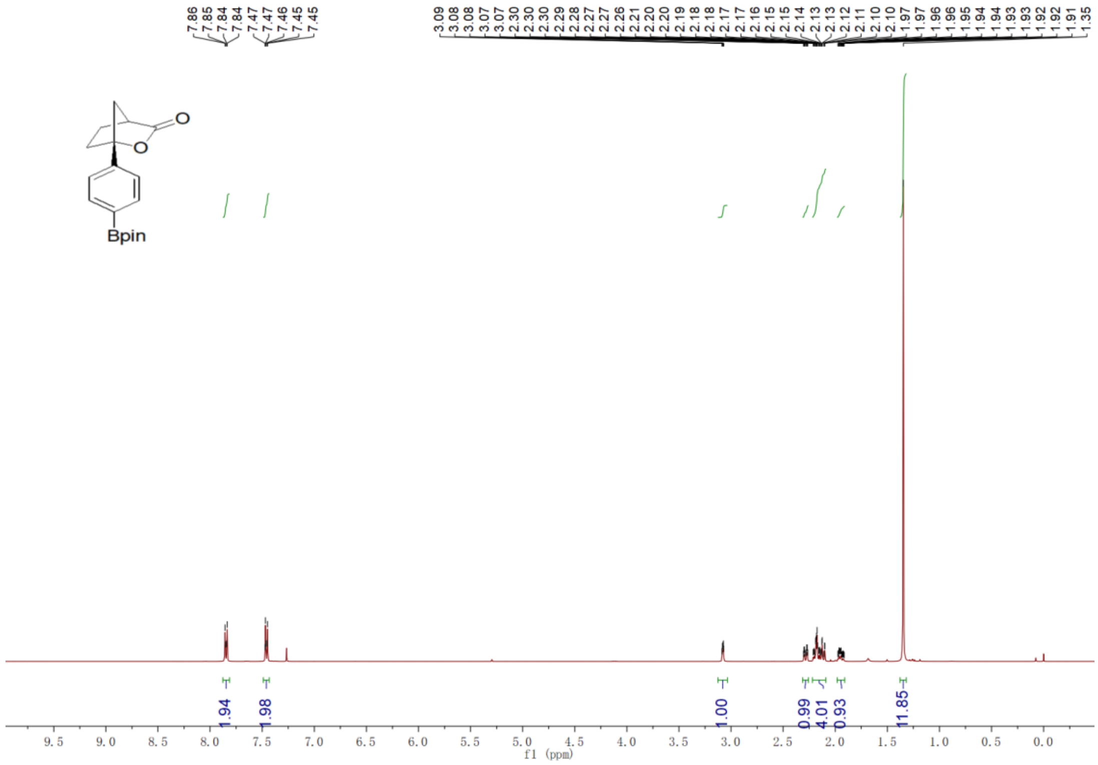

Supplementary Figure 29. ^{1}H NMR (400 MHz, $CDCl_3$) spectra for compound 3n

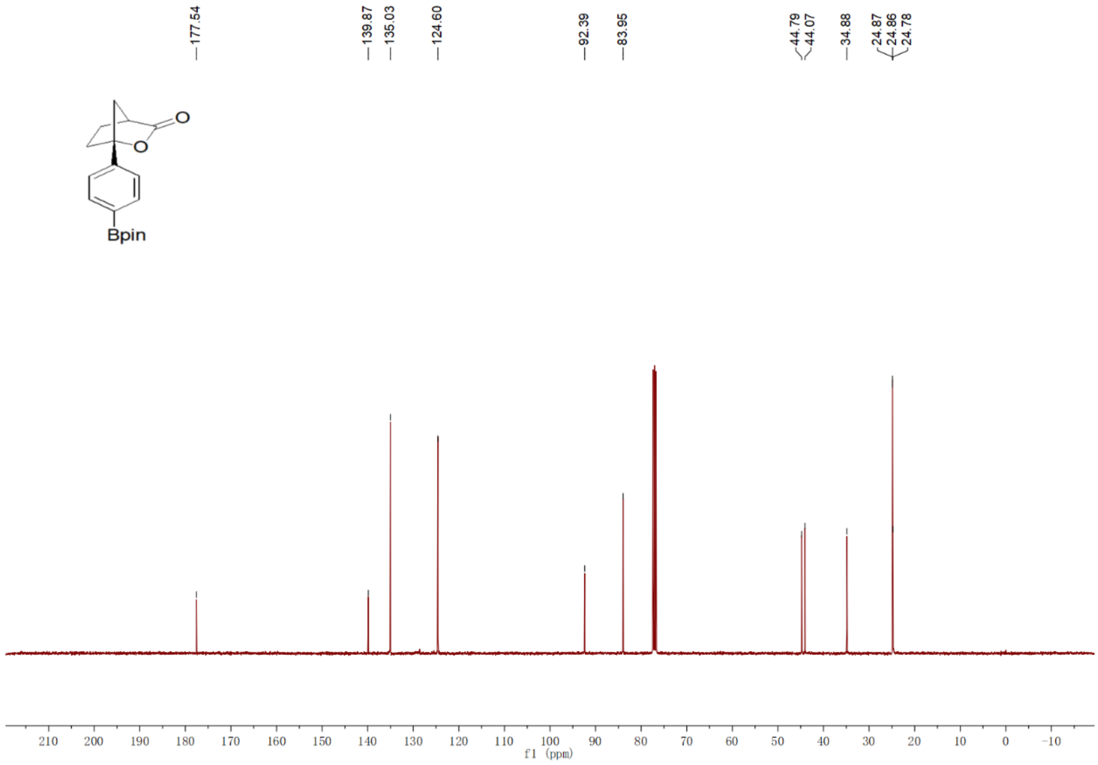

Supplementary Figure 30. ^{13}C NMR (100 MHz, $CDCl_3$) spectra for compound 3n

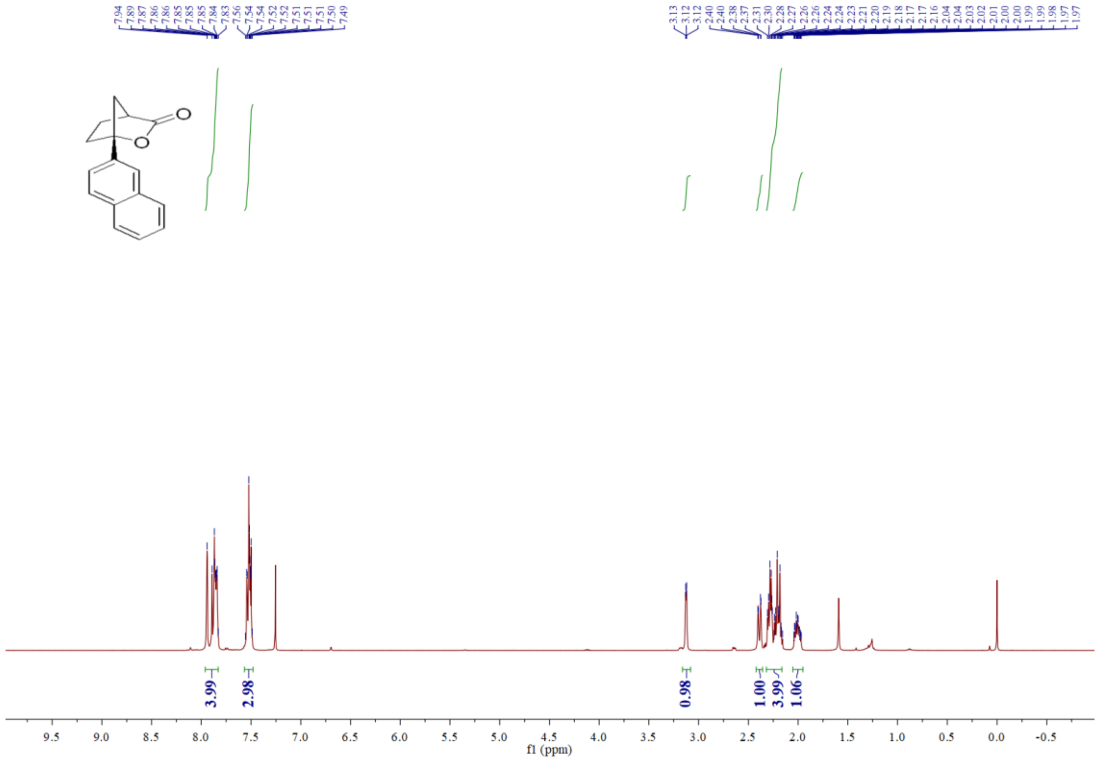

Supplementary Figure 31. ^{1}H NMR (400 MHz, $CDCl_3$) spectra for compound 3o

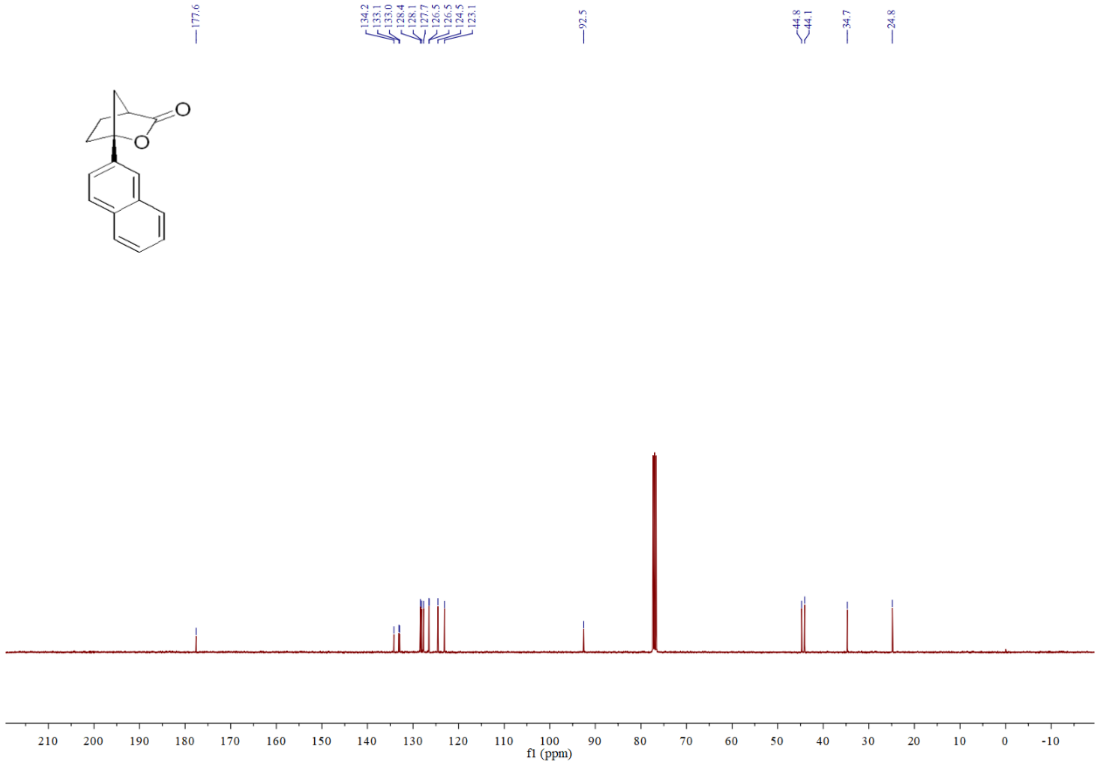

Supplementary Figure 32. ^{13}C NMR (100 MHz, $CDCl_3$) spectra for compound 3o

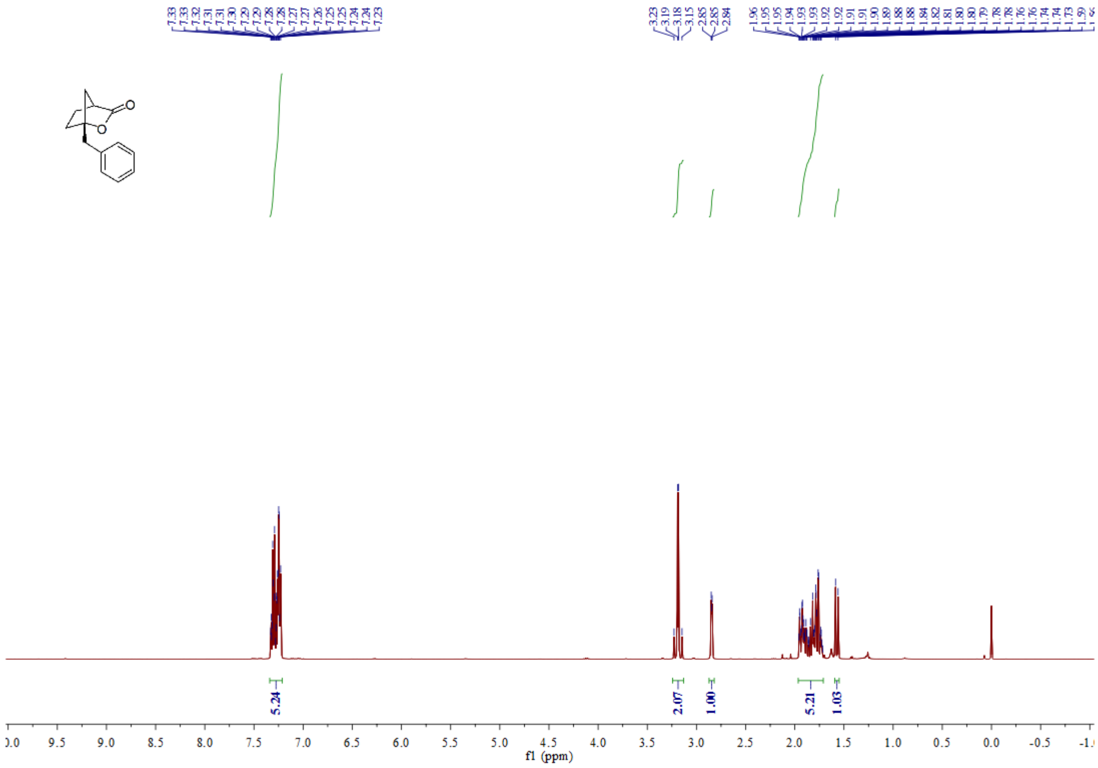

Supplementary Figure 33. **^{1}H NMR (400 MHz, $CDCl_3$) spectra for compound 3p**

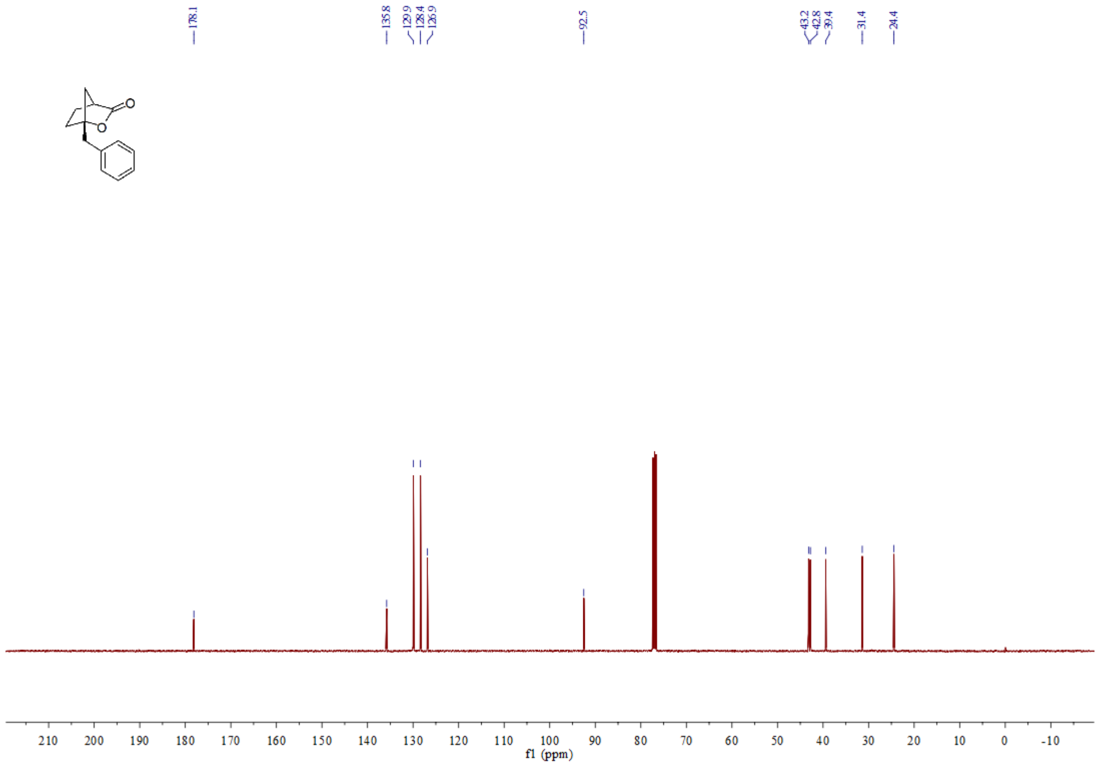

Supplementary Figure 34. **^{13}C NMR (100 MHz, $CDCl_3$) spectra for compound 3p**

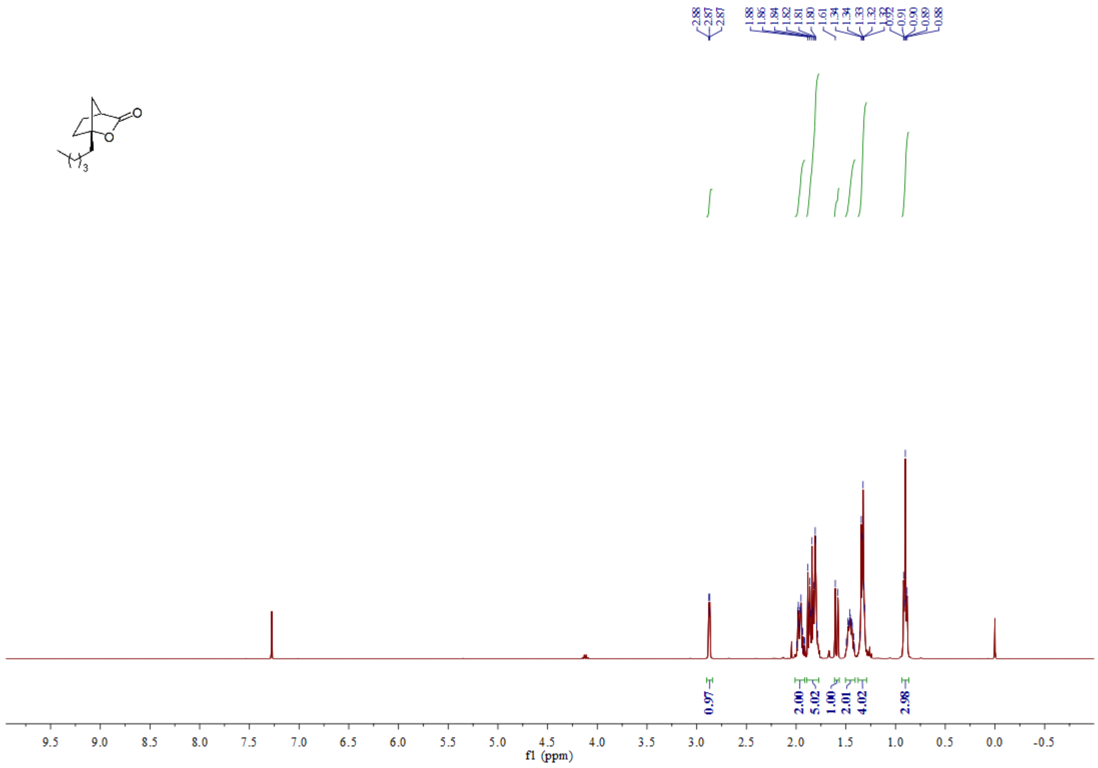

Supplementary Figure 35. ^{1}H NMR (400 MHz, $CDCl_3$) spectra for compound 3q

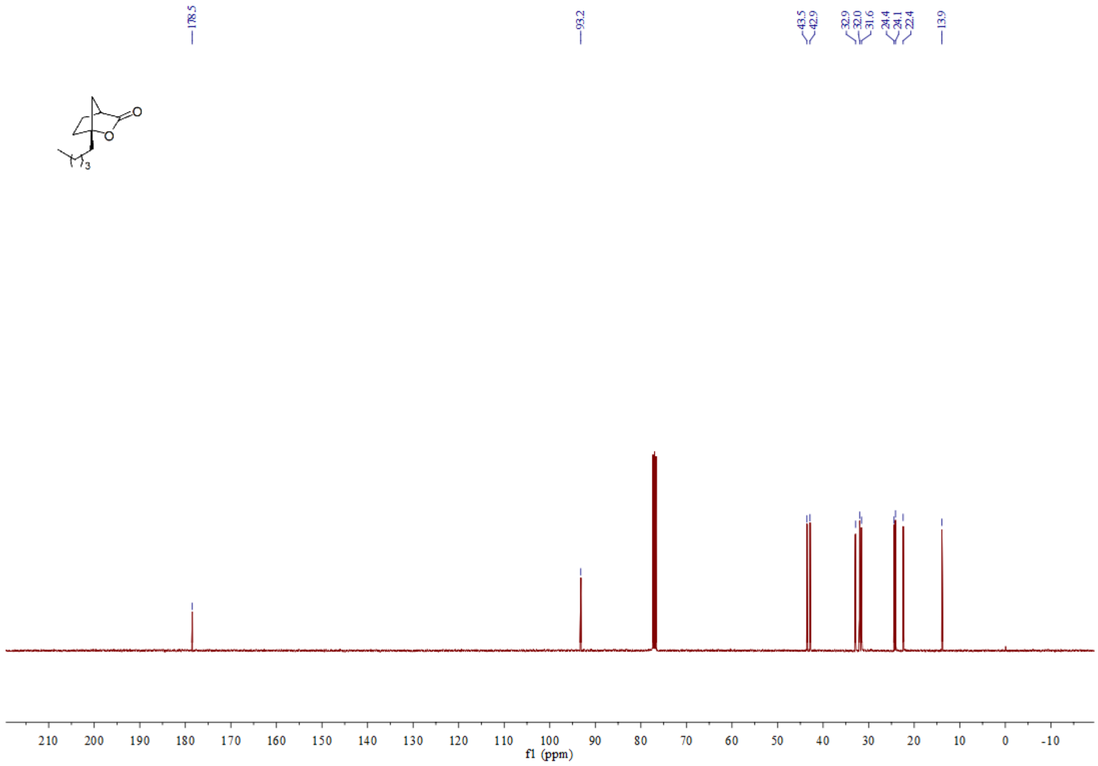

Supplementary Figure 36. ^{13}C NMR (100 MHz, $CDCl_3$) spectra for compound 3q

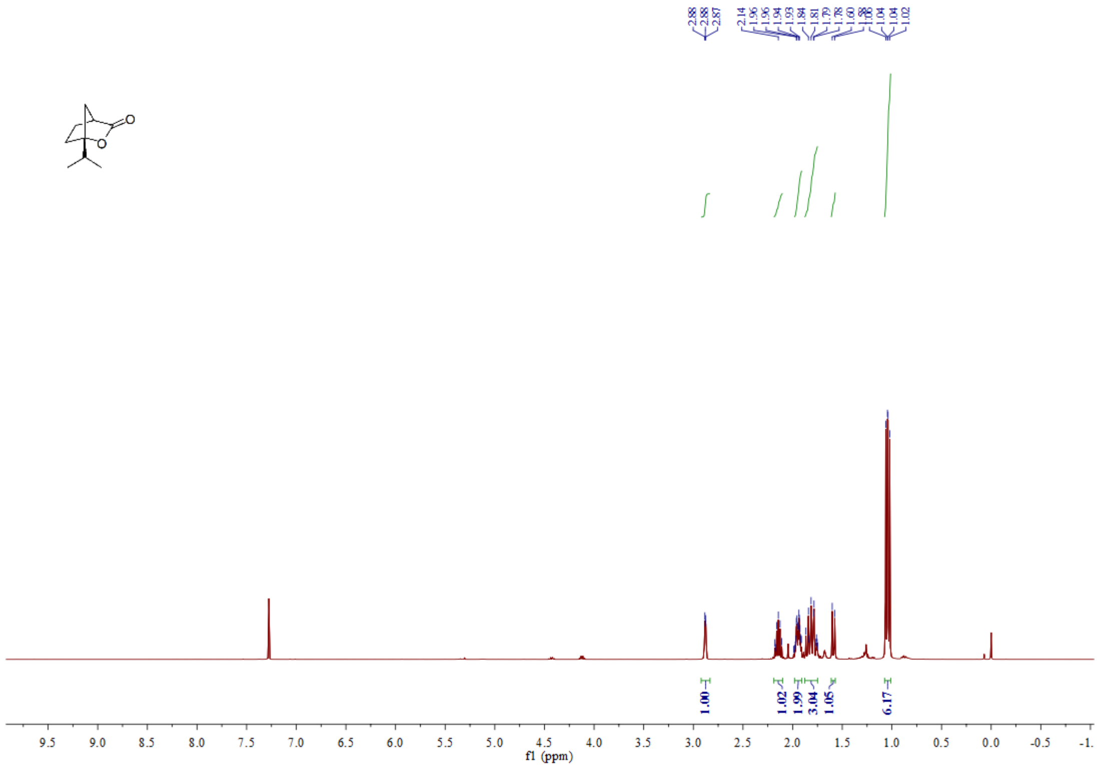

Supplementary Figure 37. ^{1}H NMR (400 MHz, CDCl$_3$) spectra for compound 3r

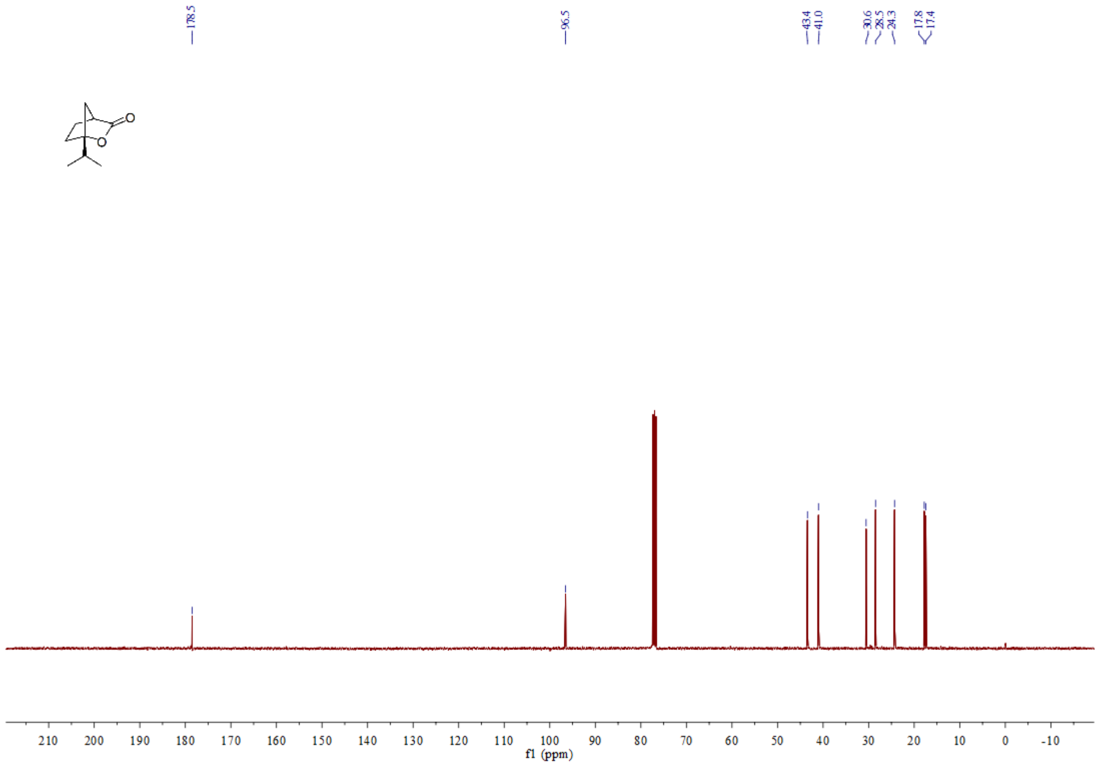

Supplementary Figure 38. ^{13}C NMR (100 MHz, CDCl$_3$) spectra for compound 3r

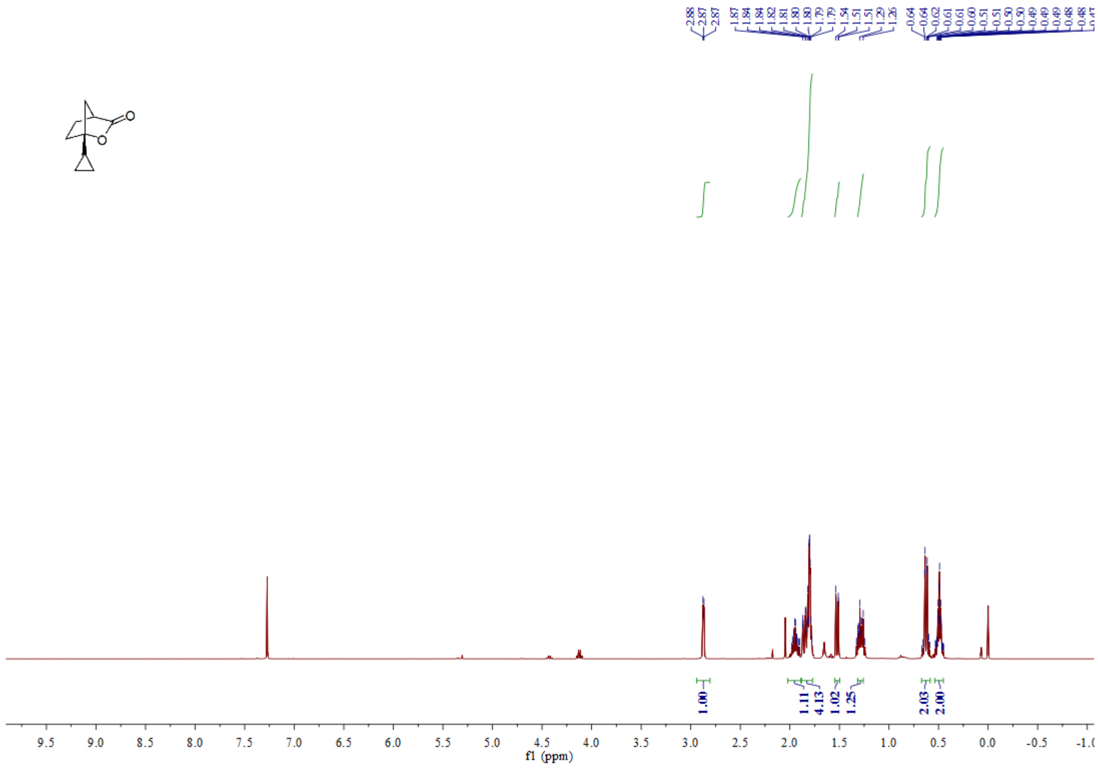

Supplementary Figure 39. ^{1}H NMR (400 MHz, $CDCl_3$) spectra for compound 3s

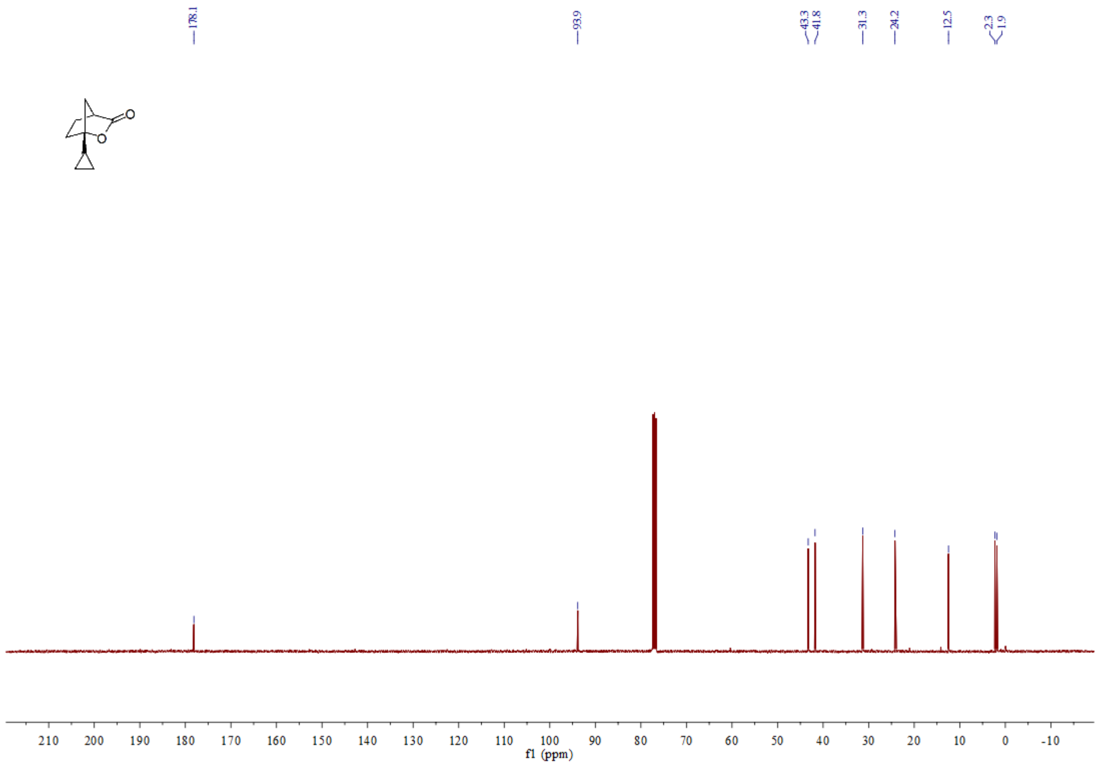

Supplementary Figure 40. ^{13}C NMR (100 MHz, $CDCl_3$) spectra for compound 3s

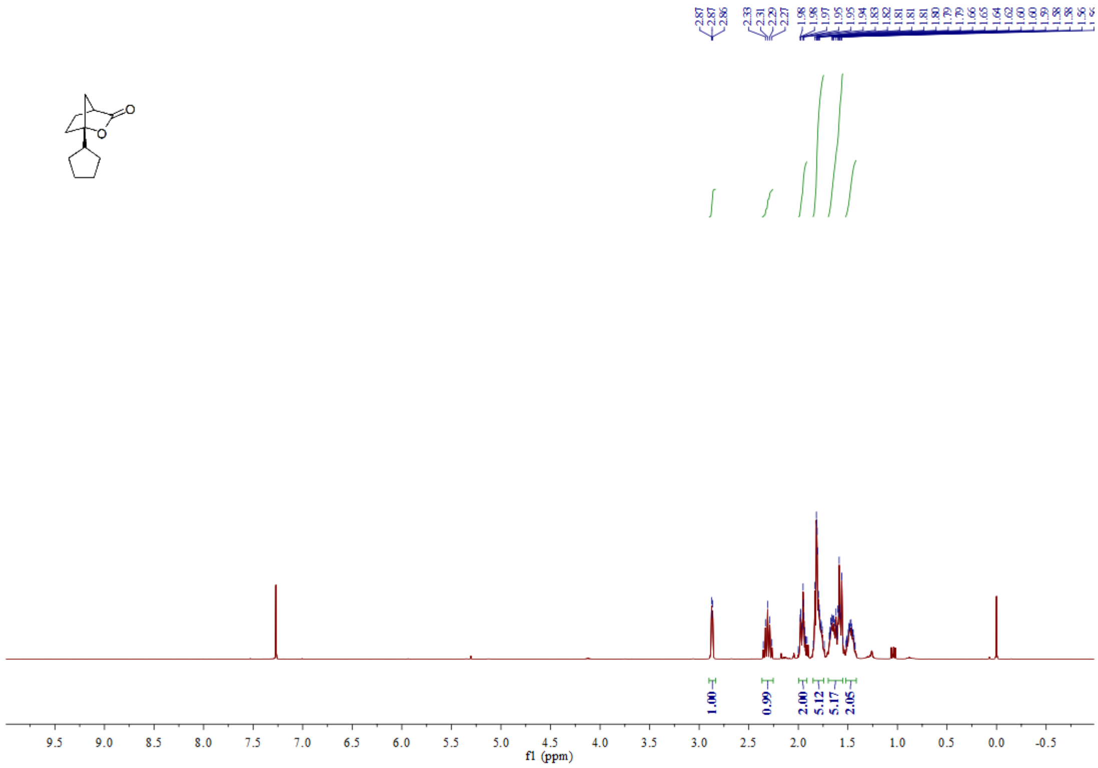

Supplementary Figure 41. ^{1}**H NMR (400 MHz, CDCl**$_3$**) spectra for compound 3t**

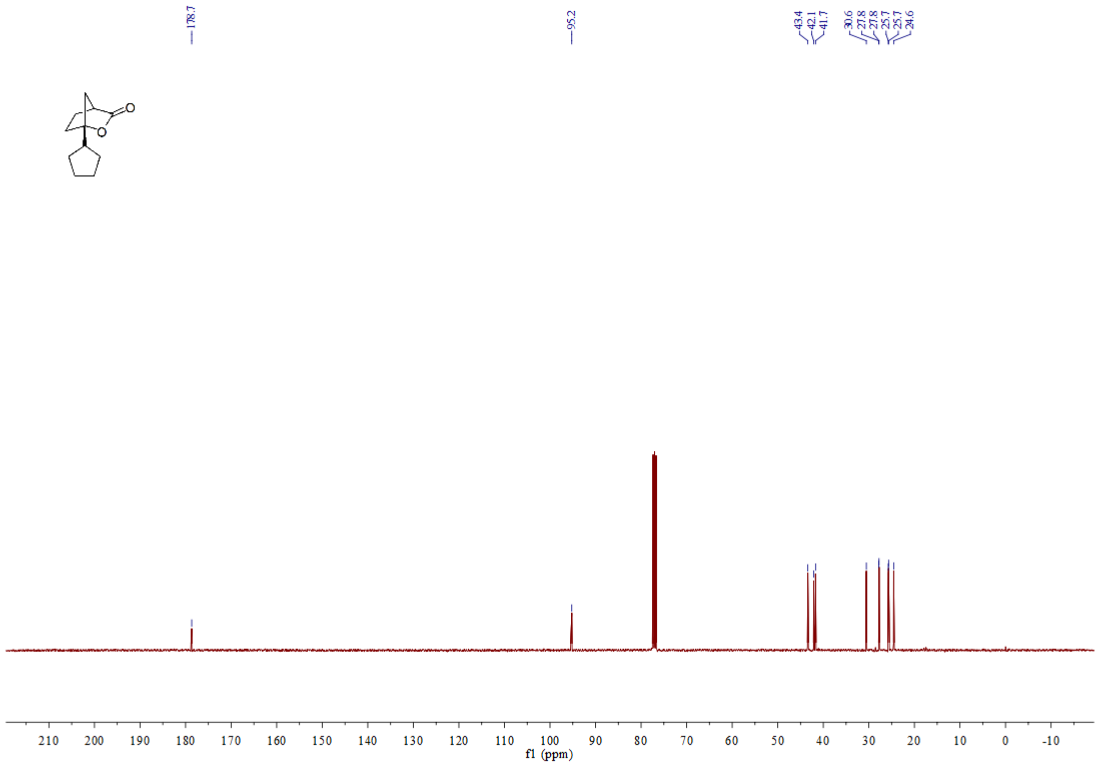

Supplementary Figure 42. ^{13}**C NMR (100 MHz, CDCl**$_3$**) spectra for compound 3t**

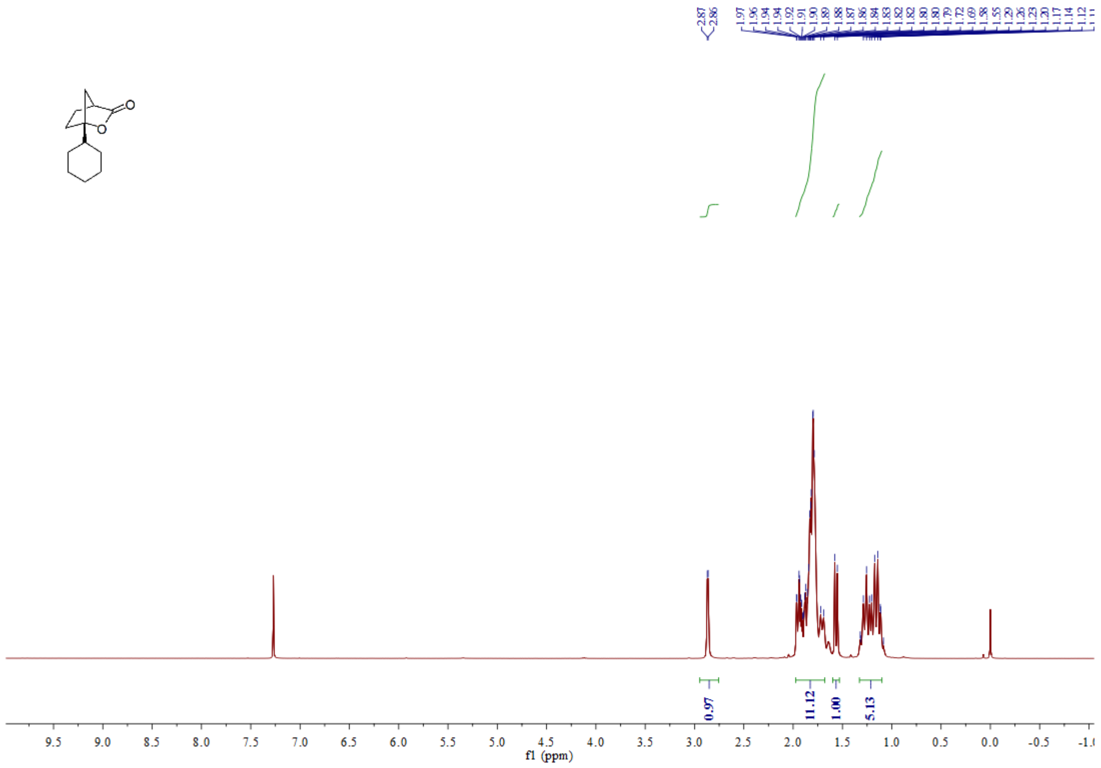

Supplementary Figure 43. ¹H NMR (400 MHz, CDCl₃) spectra for compound 3u

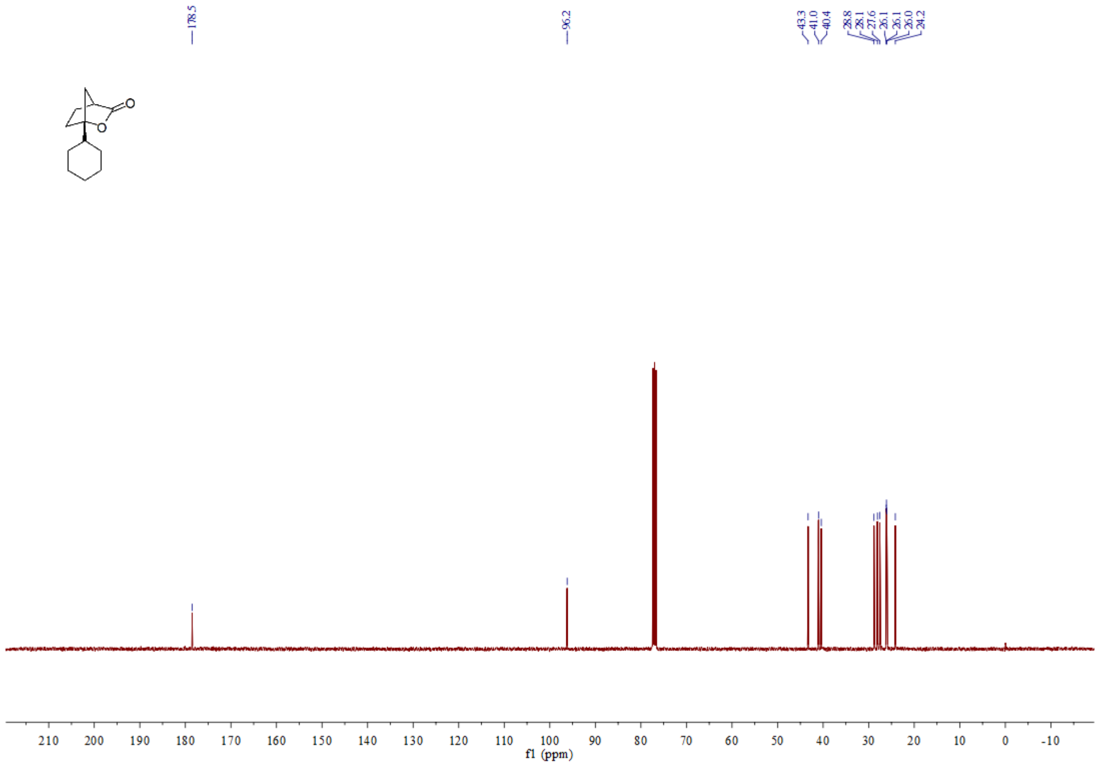

Supplementary Figure 44. ¹³C NMR (100 MHz, CDCl₃) spectra for compound 3u

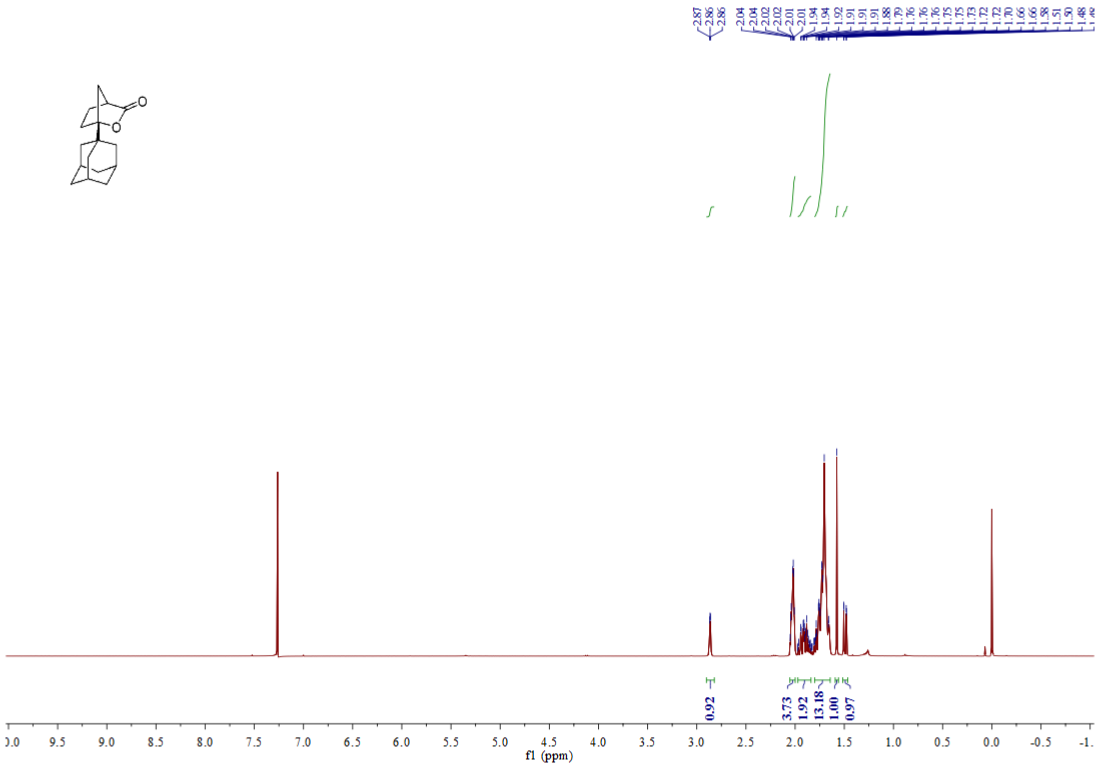

Supplementary Figure 45. ^{1}H NMR (400 MHz, $CDCl_3$) spectra for compound 3v

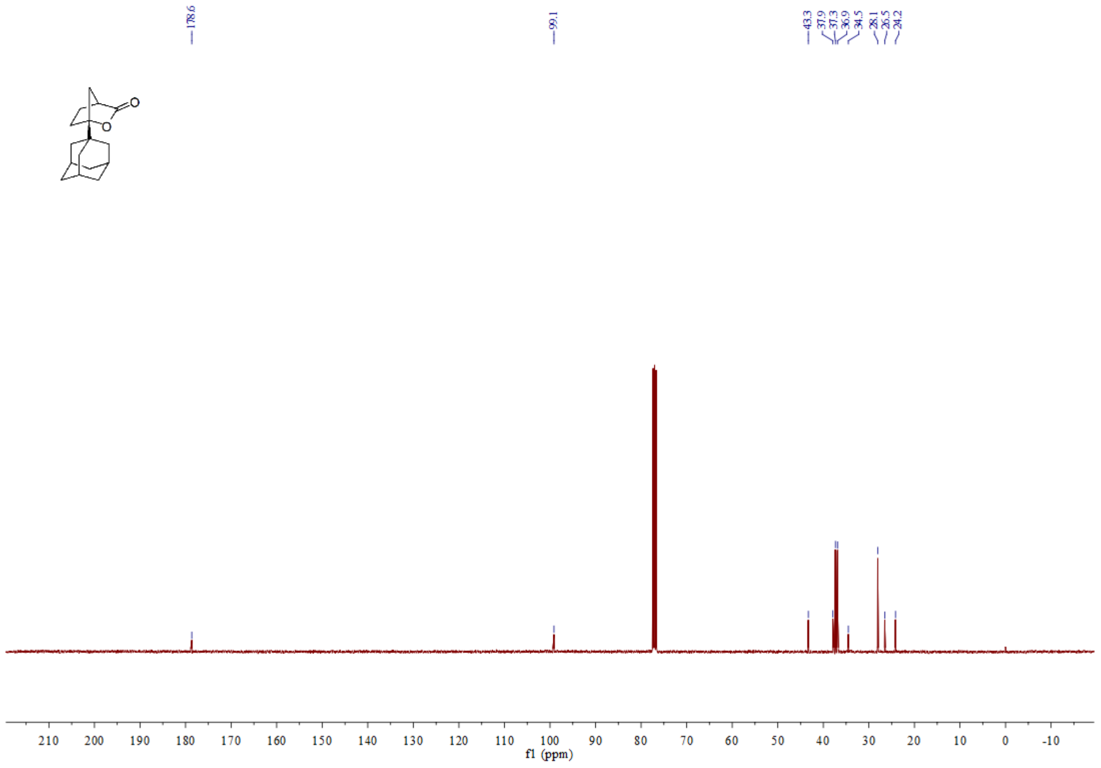

Supplementary Figure 46. ^{13}C NMR (100 MHz, $CDCl_3$) spectra for compound 3v

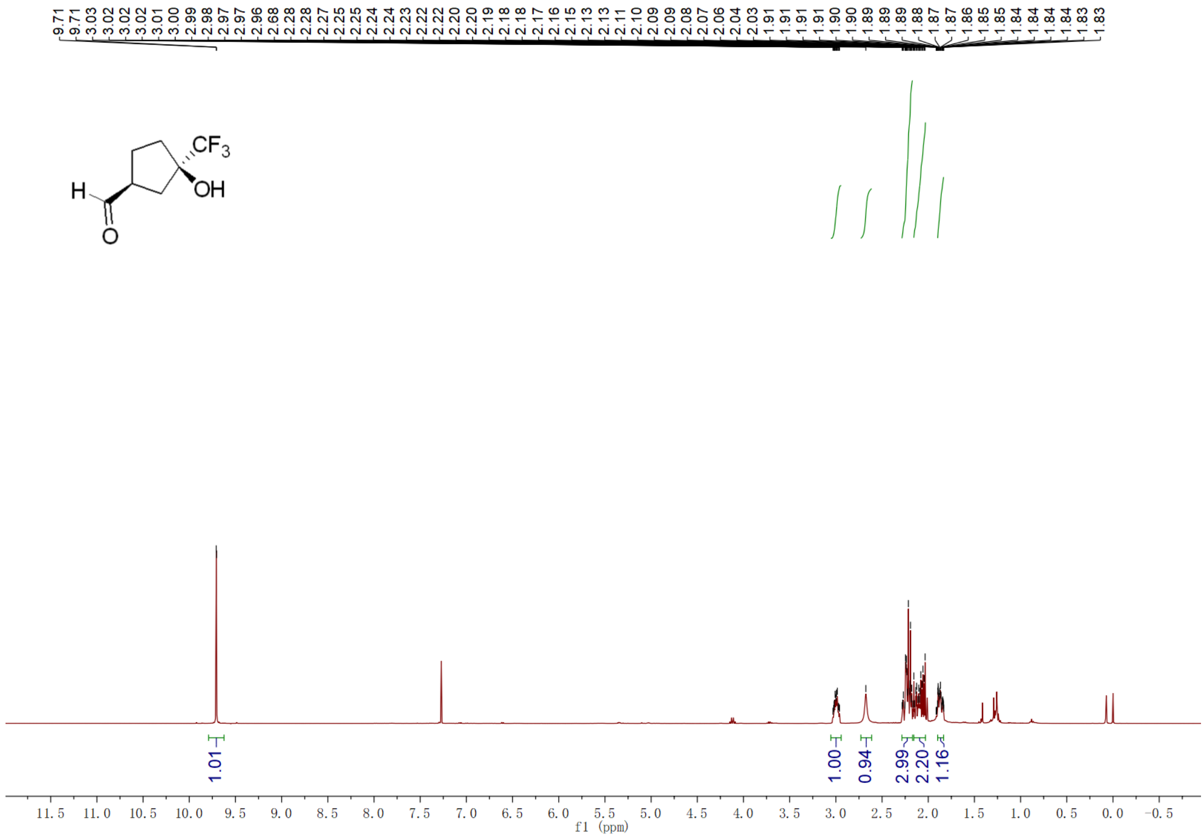

Supplementary Figure 47. ^{1}H NMR (400 MHz, $CDCl_3$) spectra for compound 2w

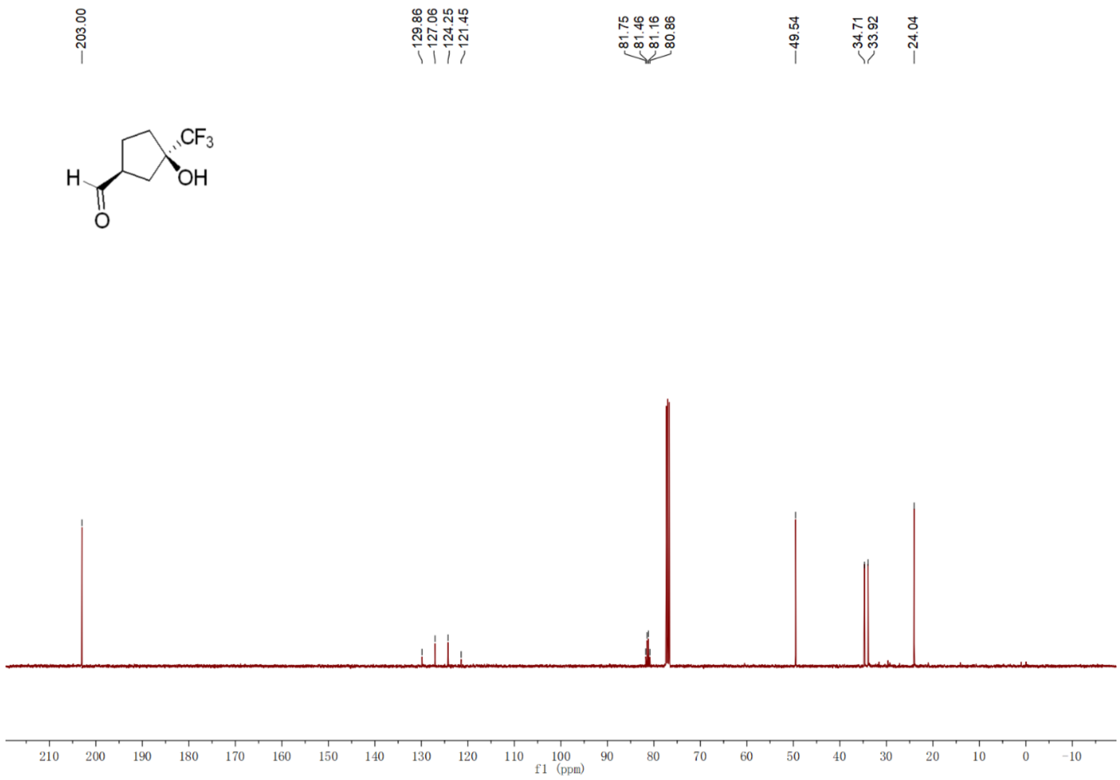

Supplementary Figure 48. ^{13}C NMR (100 MHz, $CDCl_3$) spectra for compound 2w

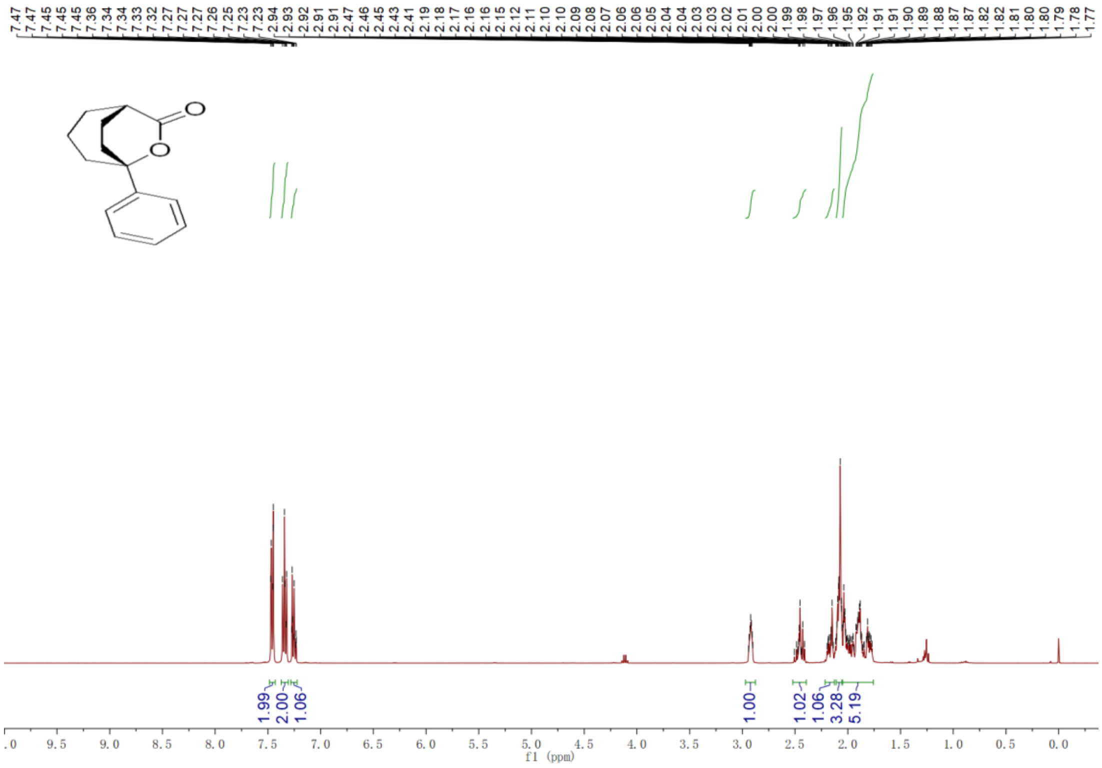

Supplementary Figure 49. ^{1}H NMR (400 MHz, $CDCl_3$) spectra for compound 3x

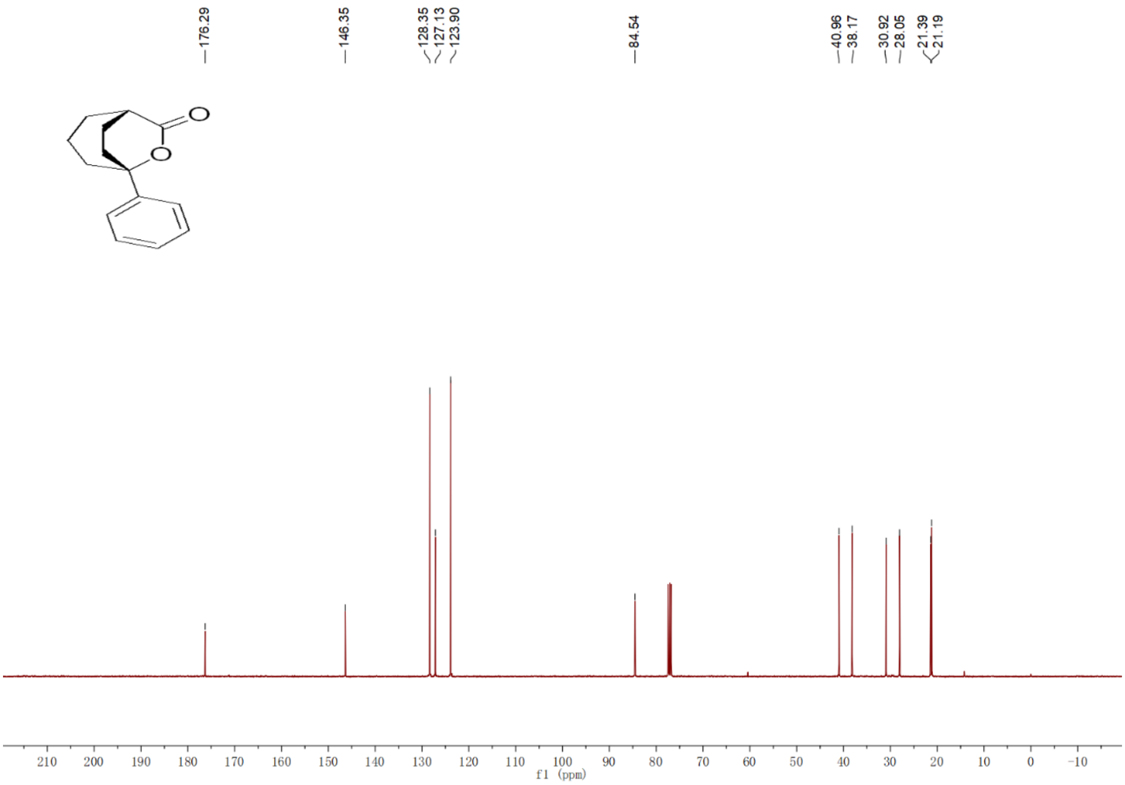

Supplementary Figure 50. ^{13}C NMR (100 MHz, $CDCl_3$) spectra for compound 3x

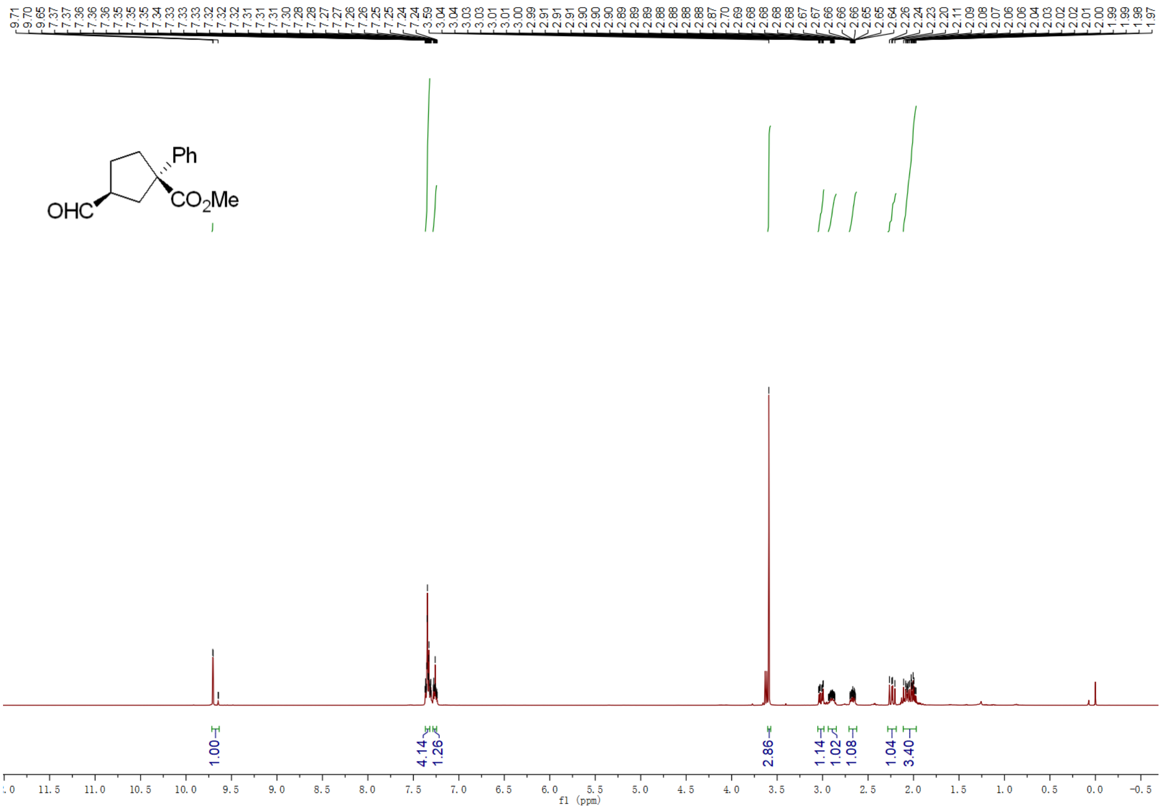

Supplementary Figure 51. ^{1}H NMR (400 MHz, CDCl3) spectra for compound 5a

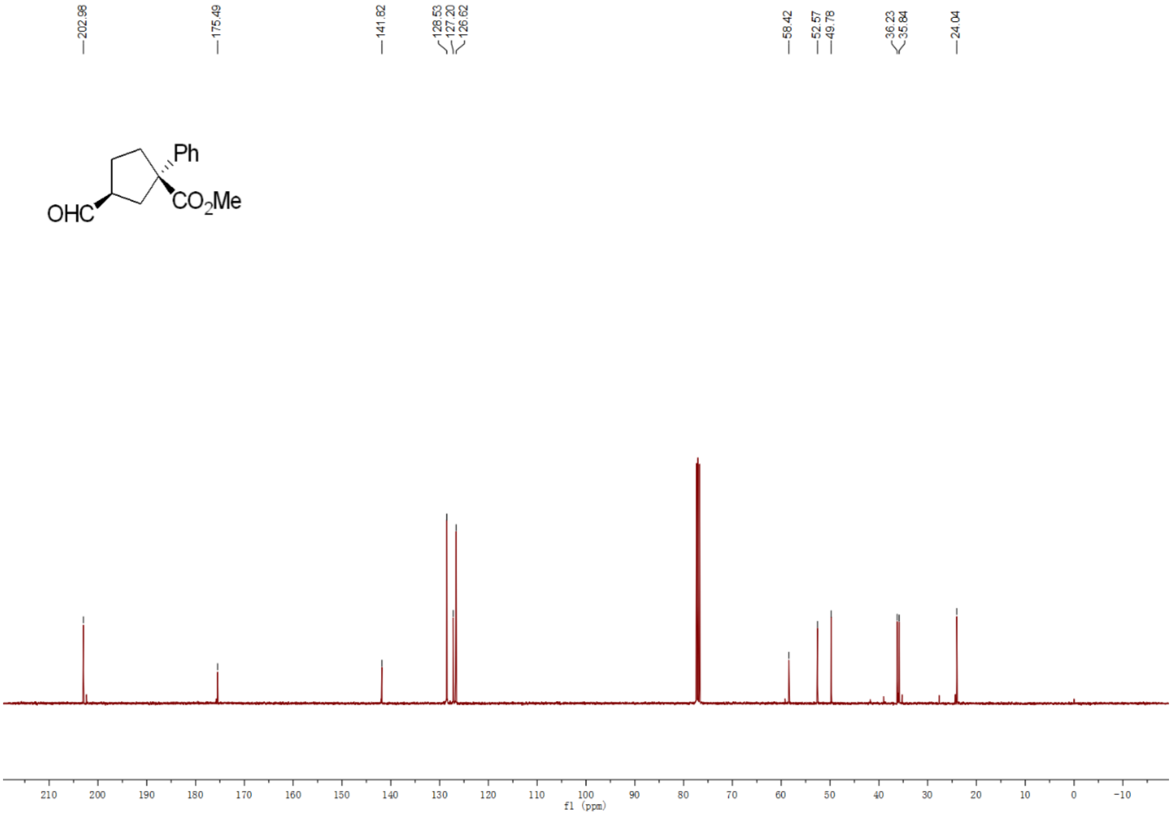

Supplementary Figure 52. ^{13}C NMR (100 MHz, CDCl3) spectra for compound 5a

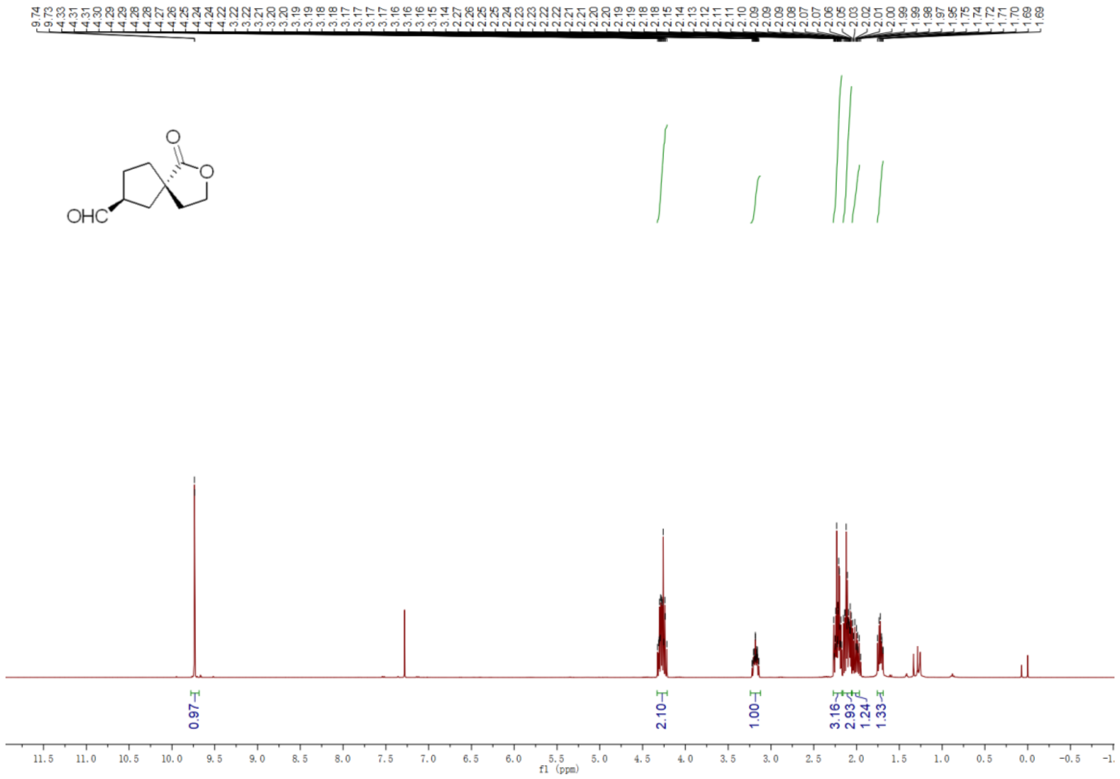

Supplementary Figure 53. ¹H NMR (400 MHz, CDCl₃) spectra for compound 5b

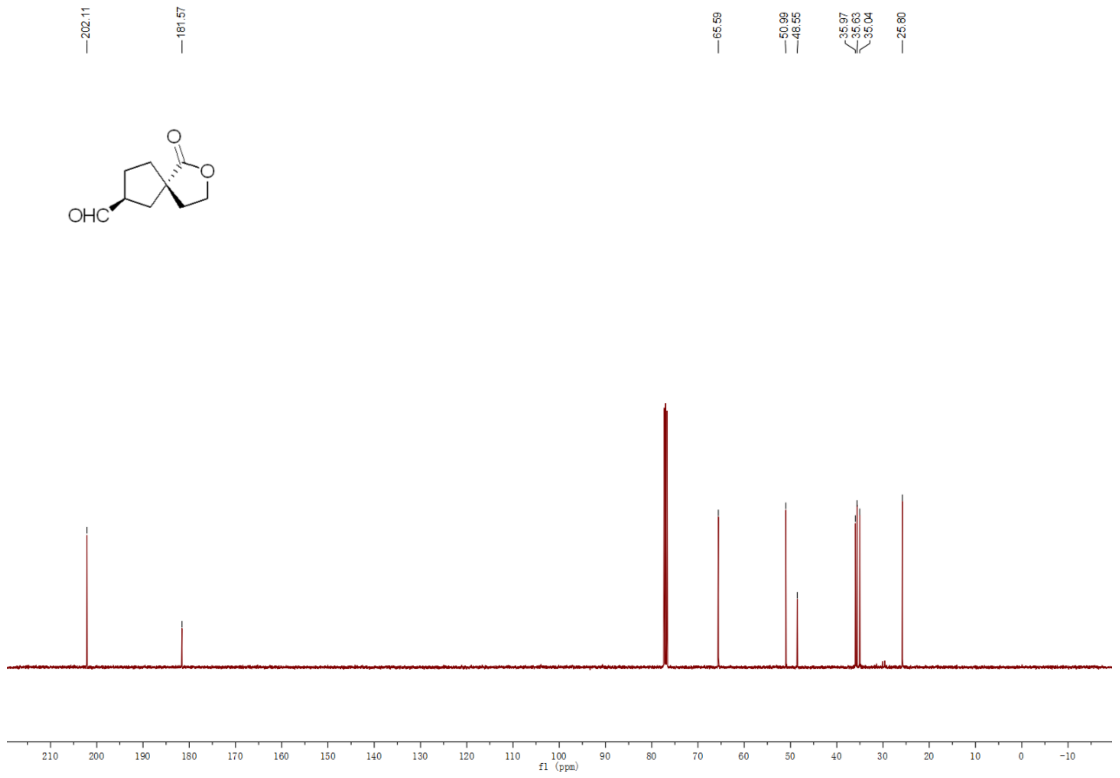

Supplementary Figure 54. ¹³C NMR (100 MHz, CDCl₃) spectra for compound 5b

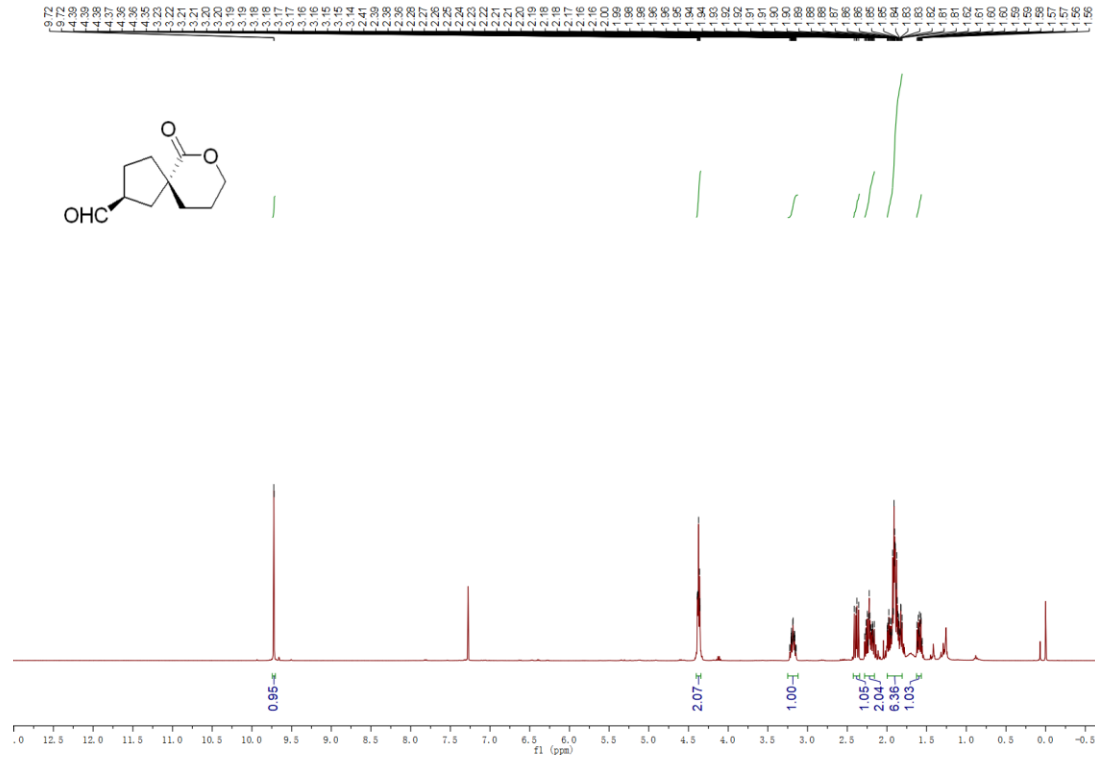

Supplementary Figure 55. ^{1}H NMR (400 MHz, $CDCl_3$) spectra for compound 5c

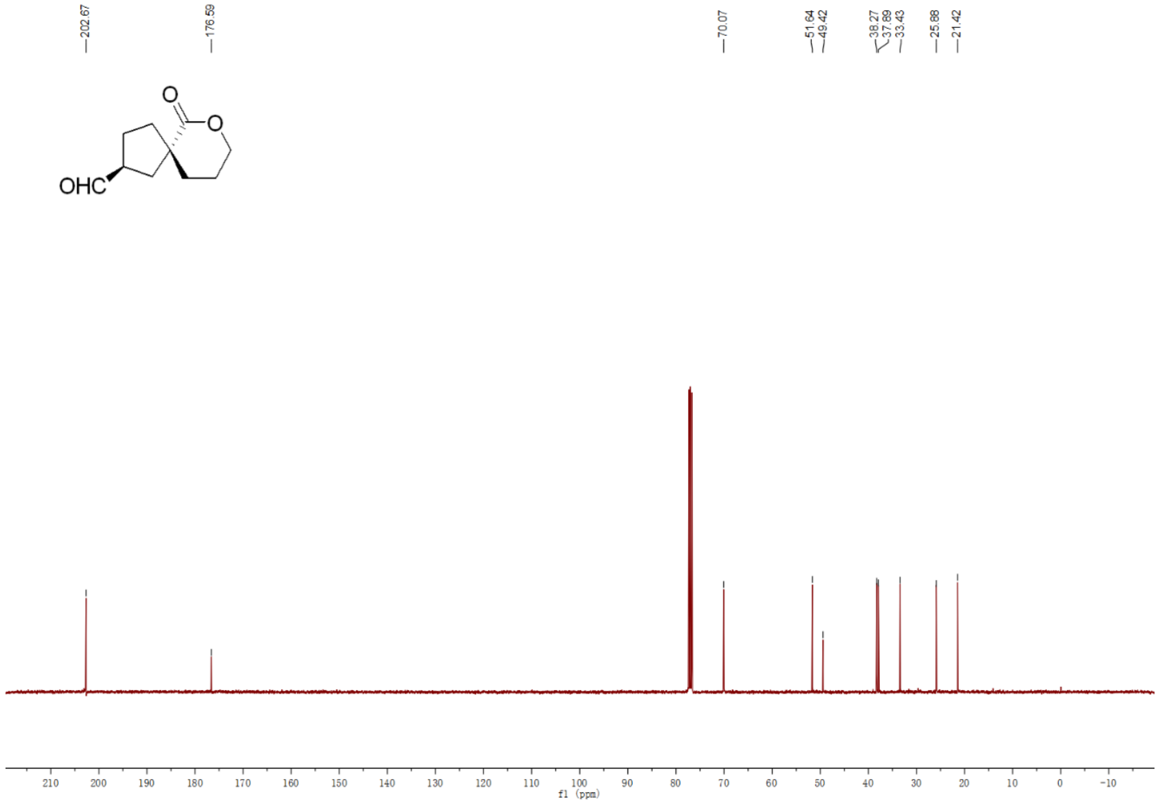

Supplementary Figure 56. ^{13}C NMR (100 MHz, $CDCl_3$) spectra for compound 5c

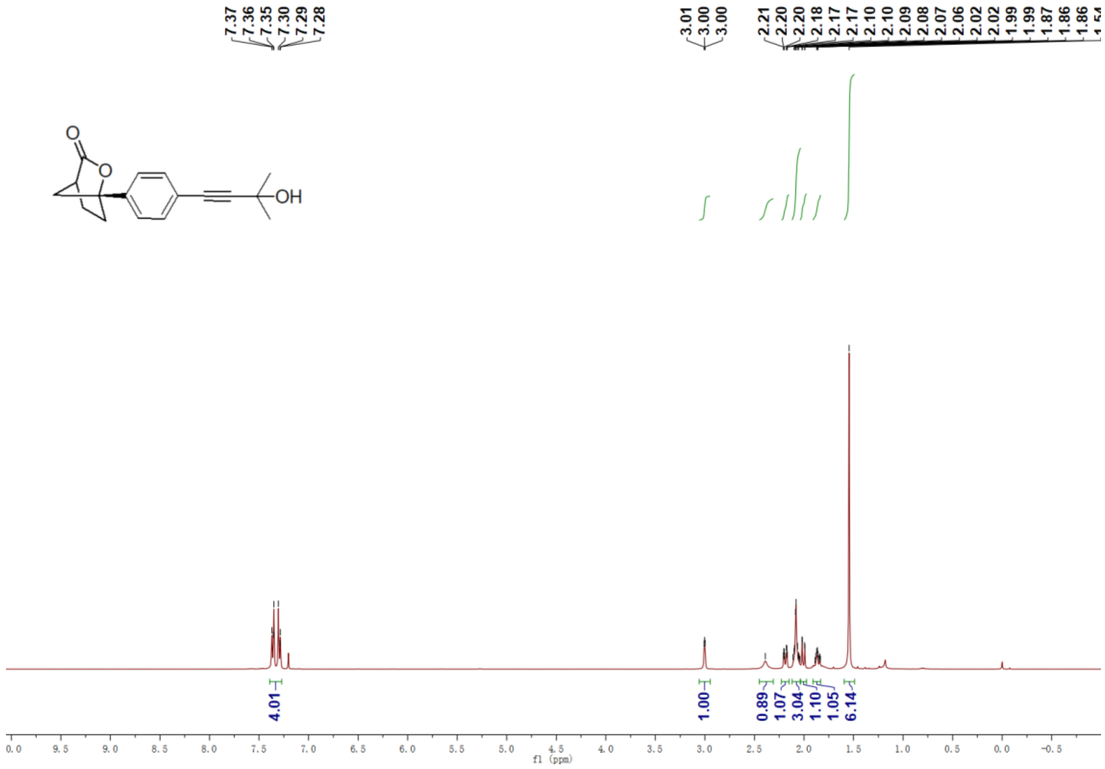

Supplementary Figure 57. ^{1}**H NMR (400 MHz, CDCl**$_3$**) spectra for compound 6**

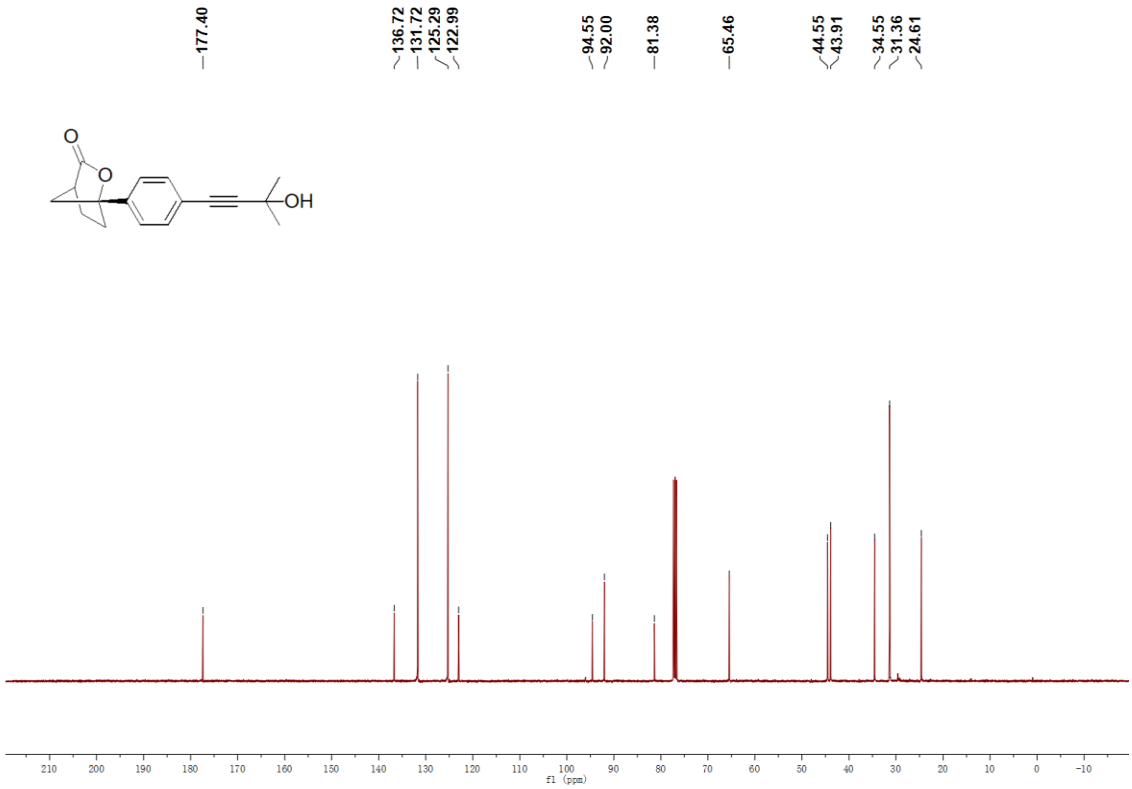

Supplementary Figure 58. ^{13}**C NMR (100 MHz, CDCl**$_3$**) spectra for compound 6**

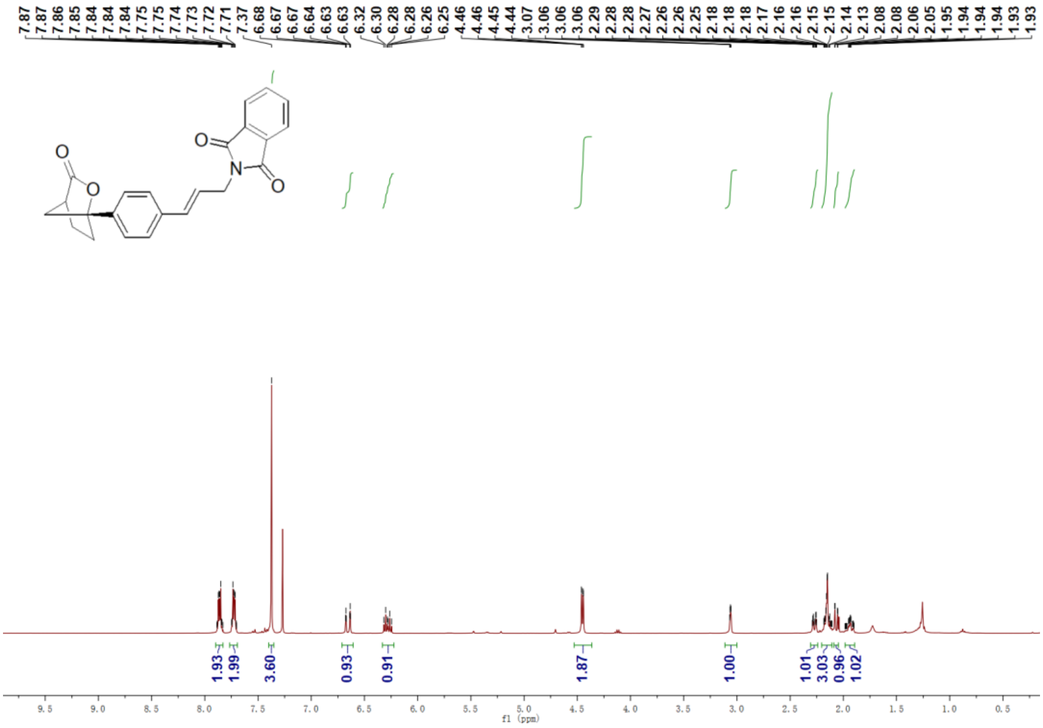

Supplementary Figure 59. ^{1}**H NMR (400 MHz, CDCl**$_3$**) spectra for compound 7**

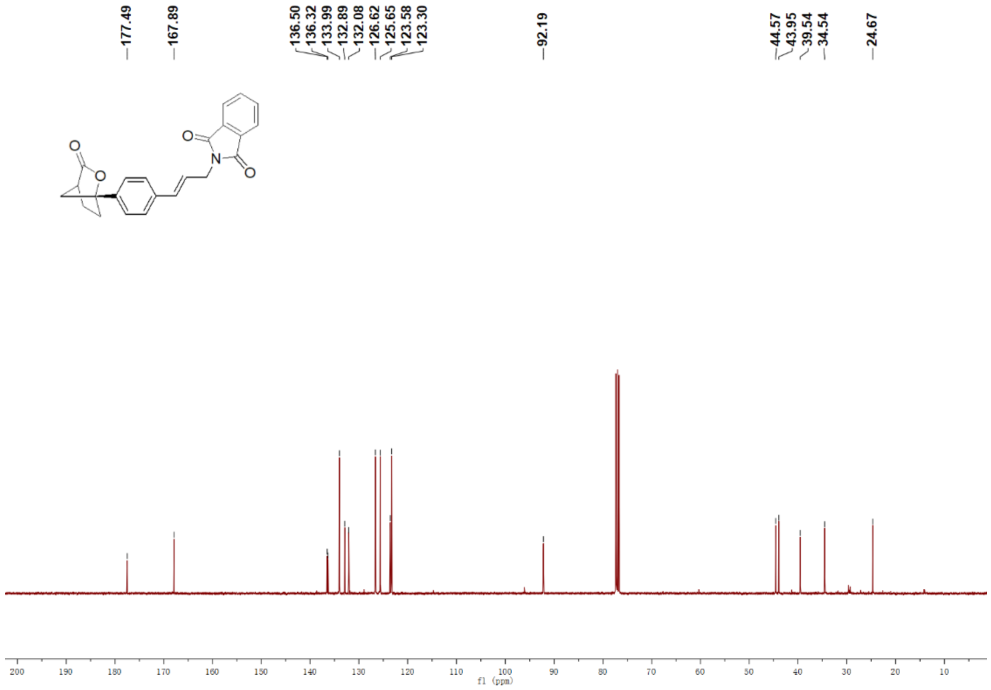

Supplementary Figure 60. ^{13}**C NMR (100 MHz, CDCl**$_3$**) spectra for compound 7**

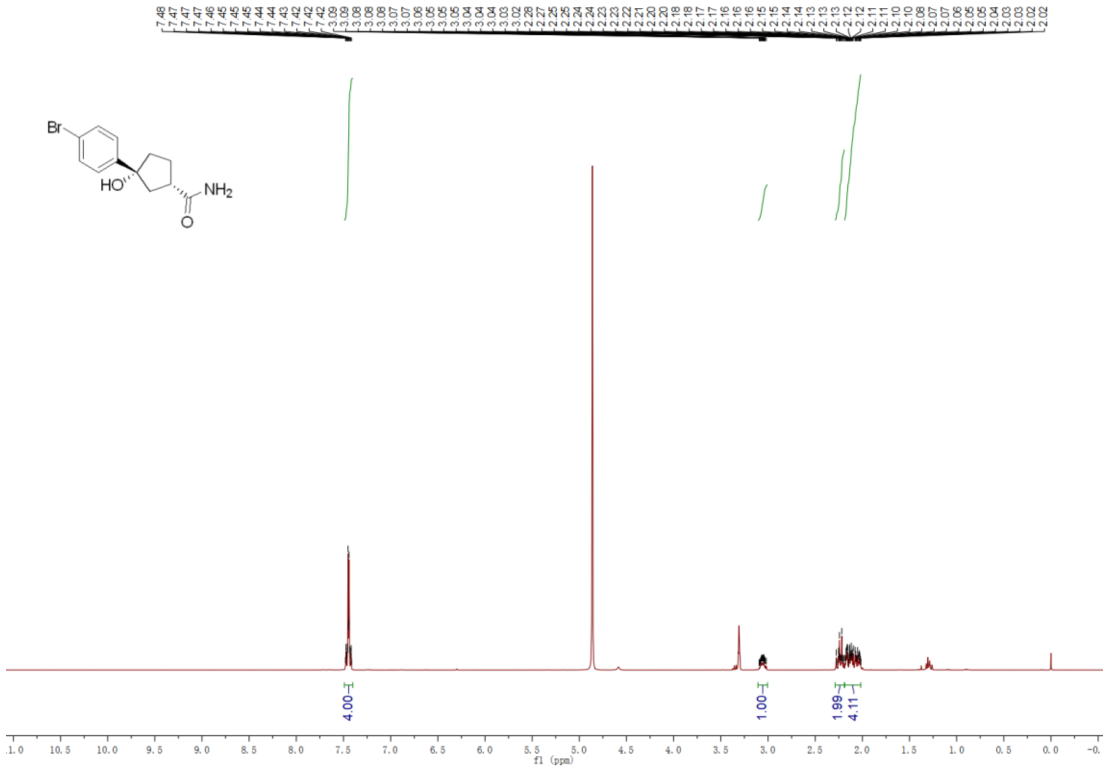

Supplementary Figure 61. ^{1}H NMR (400 MHz, CD_3OD) spectra for compound 8

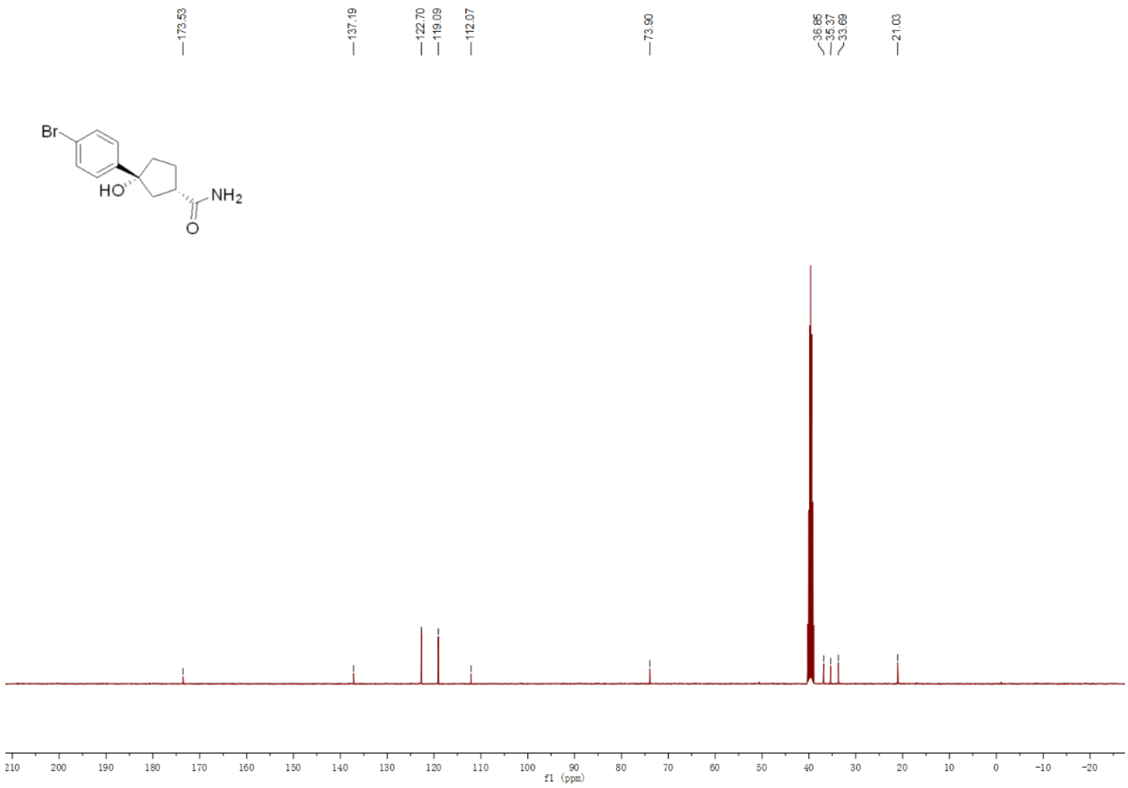

Supplementary Figure 62. ^{13}C NMR (100 MHz, DMSO-d_6) spectra for compound 8

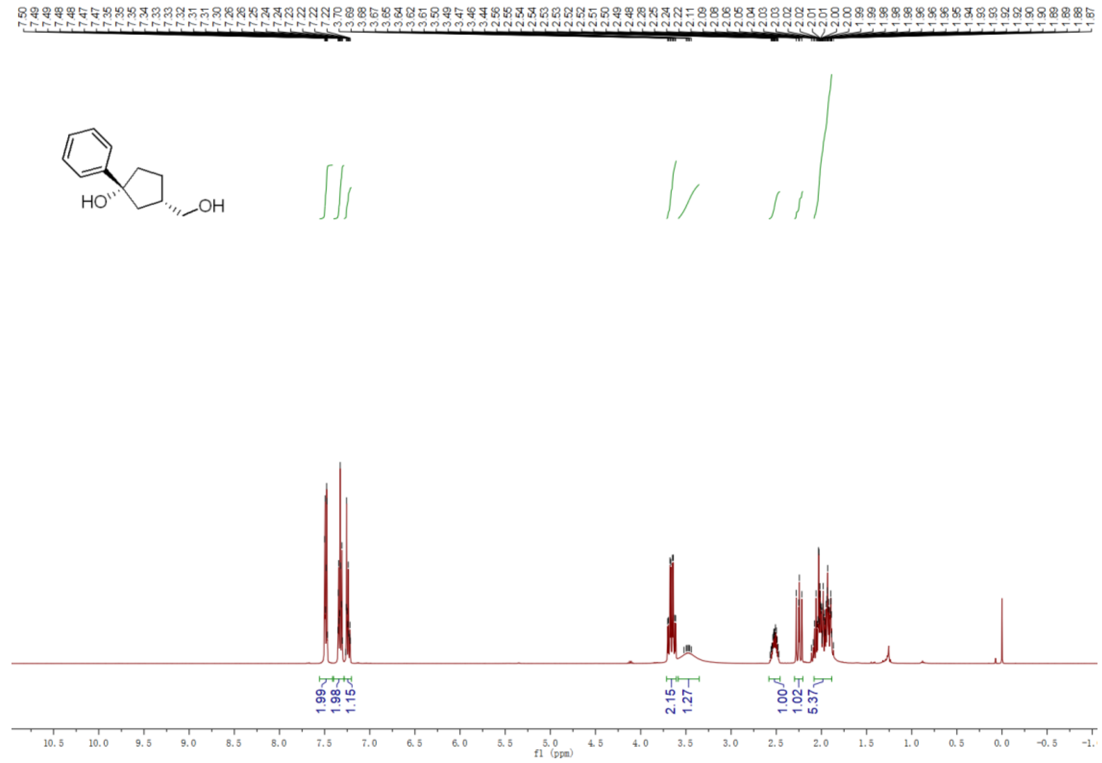

Supplementary Figure 63. ^{1}H NMR (400 MHz, CDCl$_3$) spectra for compound 9

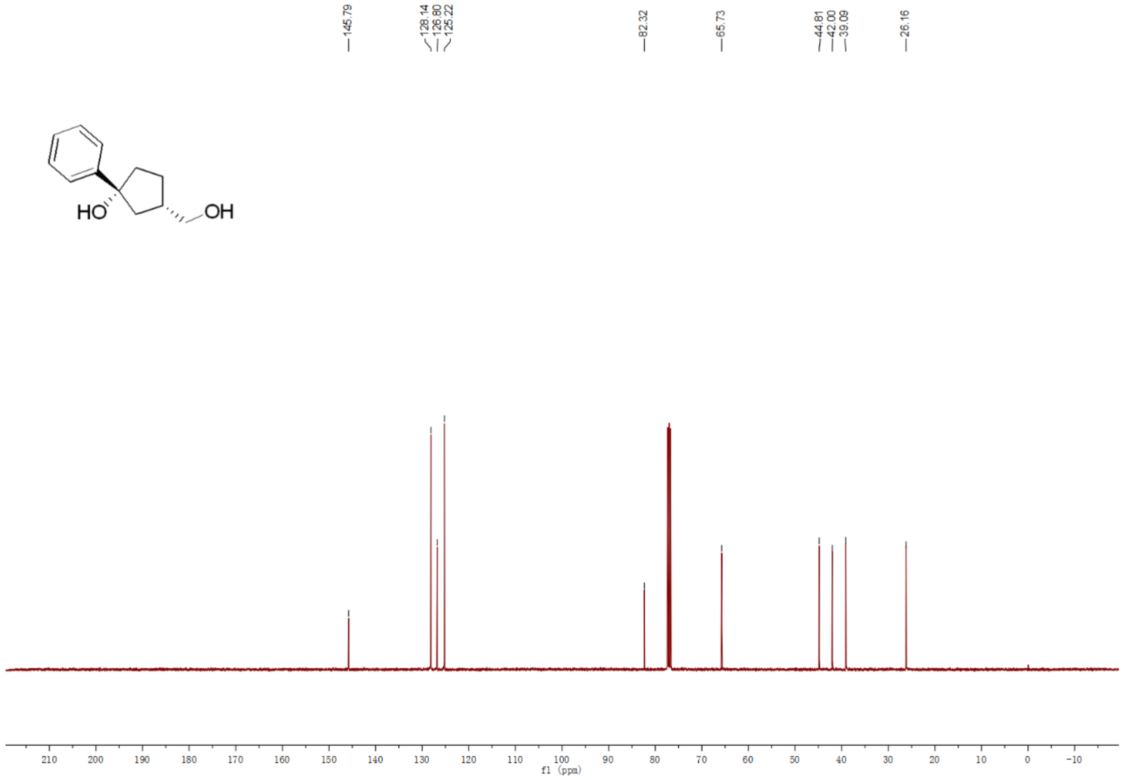

Supplementary Figure 64. ^{13}C NMR (100 MHz, CDCl$_3$) spectra for compound 9

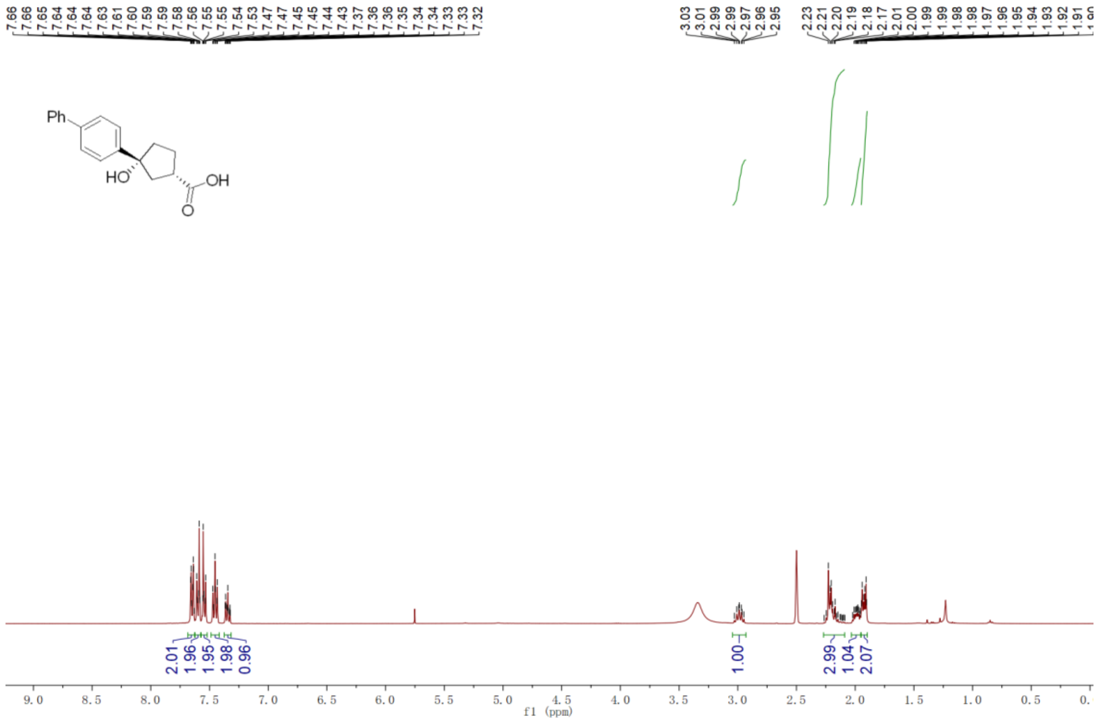

Supplementary Figure 65. ^{1}H NMR (400 MHz, DMSO-d_6) spectra for compound 10

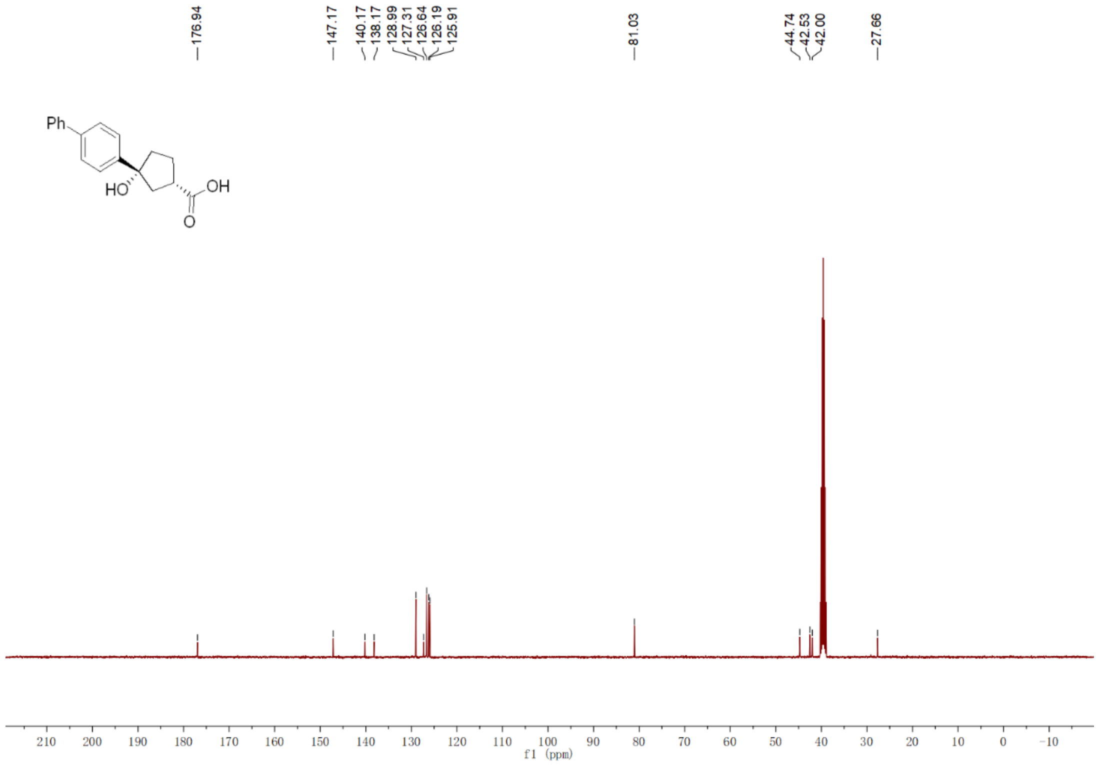

Supplementary Figure 66. ^{13}C NMR (100 MHz, DMSO-d_6) spectra for compound 10

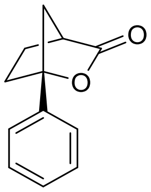

3a

Data File D:\DATA\LSL\LSL-5-22\LSL-5-22 2019-11-23 10-26-57\031-0301.D
Sample Name: LSL-biao-rac

===

Acq. Operator : Seq. Line : 3
Acq. Instrument : Instrument 1 Location : Vial 31
Injection Date : 11/23/2019 10:51:29 AM Inj : 1
Inj Volume : 5.000 µl
Acq. Method : D:\DATA\LSL\LSL-5-22\LSL-5-22 2019-11-23 10-26-57\VWD-OD(1-2)-97-3-1ML-5UL-220NM-60MIN.M
Last changed : 11/23/2019 10:05:59 AM
Analysis Method : D:\METHOD\LSL\VWD-OD(1-2)-97-3-1ML-5UL-220NM-80MIN.M
Last changed : 11/23/2019 2:59:55 PM
(modified after loading)
Additional Info : Peak(s) manually integrated

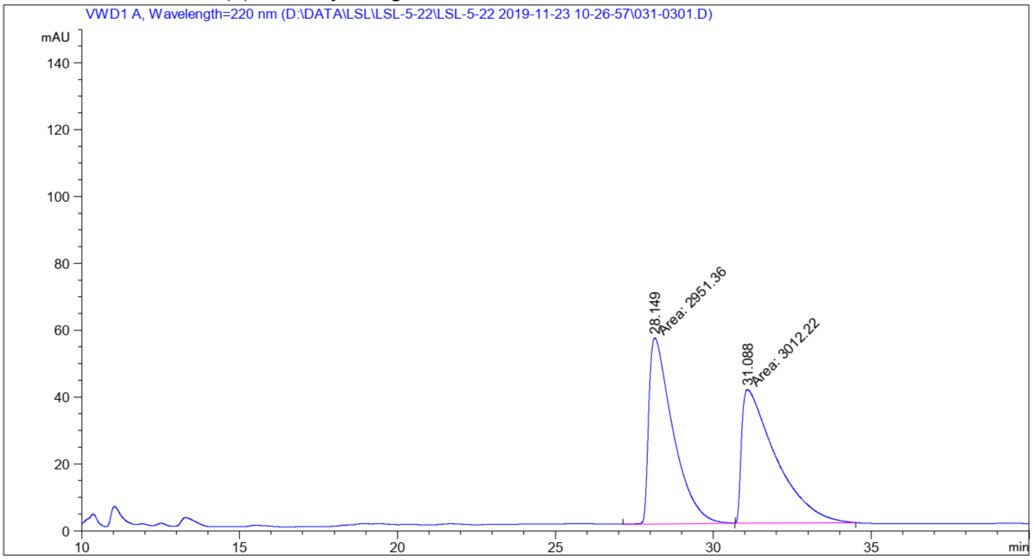

===

Area Percent Report

===

Sorted By : Signal
Multiplier : 1.0000
Dilution : 1.0000
Use Multiplier & Dilution Factor with ISTDs

Signal 1: VWD1 A, Wavelength=220 nm

Peak #	RetTime [min]	Type	Width [min]	Area [mAU*s]	Height [mAU]	Area %
1	28.149	MM	0.8842	2951.35938	55.62992	49.4897
2	31.088	MM	1.2566	3012.21777	39.95097	50.5103
Totals :				5963.57715	95.58088	

```
Data File D:\DATA\LSL\LSL-5-22\LSL-5-22 2019-11-23 10-26-57\041-0401.D
Sample Name: LSL-biao-c

    =====================================================================
    Acq. Operator   :                               Seq. Line :   4
    Acq. Instrument : Instrument 1                   Location : Vial 41
    Injection Date  : 11/23/2019 11:52:20 AM              Inj :   1
                                                   Inj Volume : 5.000 µl
    Acq. Method     : D:\DATA\LSL\LSL-5-22\LSL-5-22 2019-11-23 10-26-57\VWD-OD(1-2)-97-3-1ML-5UL-
                      220NM-60MIN.M
    Last changed    : 11/23/2019 10:05:59 AM
    Analysis Method : D:\METHOD\LSL\VWD-OD(1-2)-97-3-1ML-5UL-220NM-80MIN.M
    Last changed    : 11/23/2019 3:03:24 PM
                      (modified after loading)
    Additional Info : Peak(s) manually integrated
```

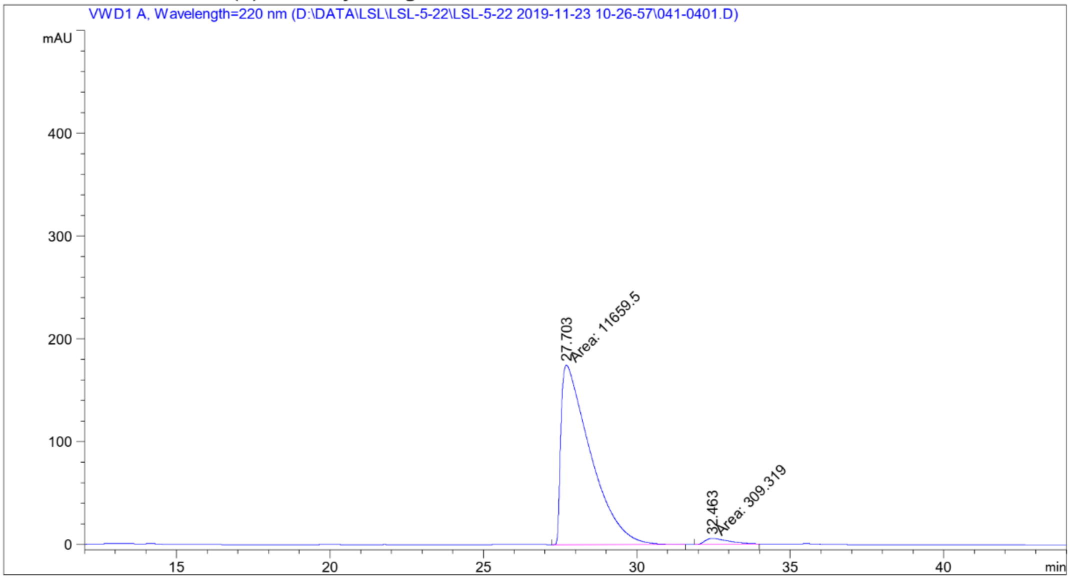


```
    =====================================================================
                             Area Percent Report
    =====================================================================

    Sorted By             :      Signal
    Multiplier            :      1.0000
    Dilution              :      1.0000
    Use Multiplier & Dilution Factor with ISTDs


    Signal 1: VWD1 A, Wavelength=220 nm

    Peak RetTime Type  Width     Area      Height     Area
      #   [min]        [min]   [mAU*s]     [mAU]        %
    ----|-------|----|-------|----------|----------|--------|
       1  27.703 MM    1.1131 1.16595e4  174.58202 97.4156
       2  32.463 MM    0.9040  309.31918    5.70269   2.5844

    Totals :                  1.19688e4  180.28471
```


Supplementary Figure 67. HPLC spectra for compound 3a

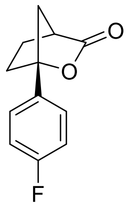

3b

Data File D:\DATA\LWD\LWD-6-95\LWD-6-95 2019-11-21 09-11-41\041-0901.D
Sample Name: LSL-5-p-F

===

```
Acq. Operator   :                               Seq. Line :   9
Acq. Instrument : Instrument 1                   Location : Vial 41
Injection Date  : 11/21/2019 1:26:29 PM               Inj :   1
                                               Inj Volume : 5.000 µl
Acq. Method     : D:\DATA\LWD\LWD-6-95\LWD-6-95 2019-11-21 09-11-41\VWD-OJ(1-6)-95-5-1.0ML-
                  5UL-210NM-60MIN.M
Last changed    : 11/21/2019 9:46:13 AM
Analysis Method : D:\METHOD\LSL\VWD-OJ(1-6)-95-5-1.0ML-5UL-220NM-60MIN.M
Last changed    : 11/21/2019 7:56:36 PM
                  (modified after loading)
Additional Info : Peak(s) manually integrated
```

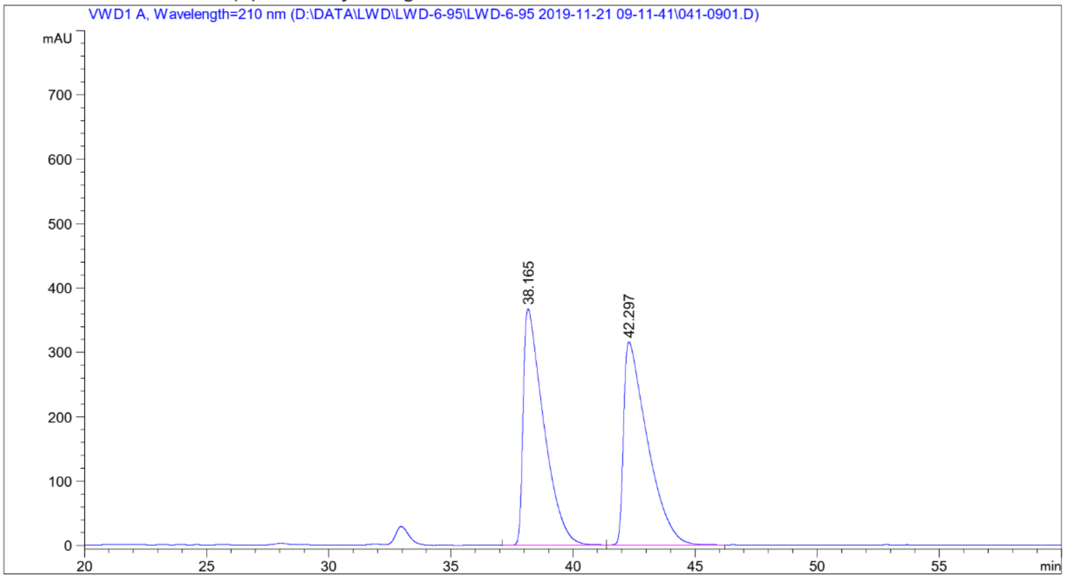

===

Area Percent Report

===

```
Sorted By             :      Signal
Multiplier            :      1.0000
Dilution              :      1.0000
Use Multiplier & Dilution Factor with ISTDs
```

Signal 1: VWD1 A, Wavelength=210 nm

Peak #	RetTime [min]	Type	Width [min]	Area [mAU*s]	Height [mAU]	Area %
1	38.165	BB	0.8551	2.13633e4	367.02646	50.0763
2	42.297	BB	0.9631	2.12982e4	315.28107	49.9237

Totals : 4.26615e4 682.30753

Data File D:\DATA\LWD\LWD-6-95\LWD-6-95 2019-11-21 09-11-41\042-1001.D
Sample Name: LSL-5-p-F chiral

===

Acq. Operator   :   Seq. Line : 10
Acq. Instrument : Instrument 1   Location : Vial 42
Injection Date : 11/21/2019 2:27:20 PM   Inj :   1
                                       Inj Volume : 5.000 µl
Acq. Method   : D:\DATA\LWD\LWD-6-95\LWD-6-95 2019-11-21 09-11-41\VWD-OJ(1-6)-95-5-1.0ML-5UL-210NM-60MIN.M
Last changed   : 11/21/2019 9:46:13 AM
Analysis Method : D:\METHOD\LSL\VWD-OJ(1-6)-95-5-1.0ML-5UL-220NM-60MIN.M
Last changed   : 11/21/2019 7:59:19 PM
                  (modified after loading)
Additional Info : Peak(s) manually integrated

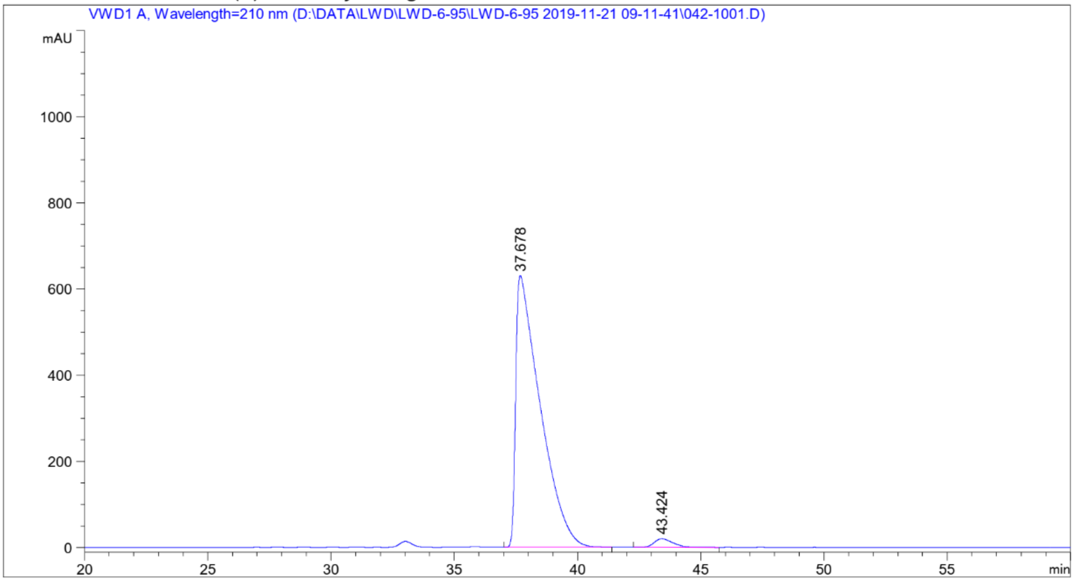

===

Area Percent Report

===

Sorted By : Signal
Multiplier : 1.0000
Dilution : 1.0000
Use Multiplier & Dilution Factor with ISTDs

Signal 1: VWD1 A, Wavelength=210 nm

Peak #	RetTime [min]	Type	Width [min]	Area [mAU*s]	Height [mAU]	Area %
1	37.678	BB	0.9608	4.34579e4	630.77051	97.5999
2	43.424	BB	0.8086	1068.68408	19.89797	2.4001

Totals : 4.45266e4 650.66847

Instrument 1 11/21/2019 7:59:23 PM   Page 1 of 2

Supplementary Figure 68. HPLC spectra for compound 3b

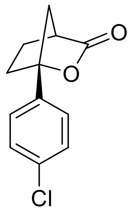

3c

Data File D:\DATA\LWD\LWD-6-95\LWD-6-95 2019-11-21 09-11-41\043-1201.D
Sample Name: LSL-5-p-Cl

===

Acq. Operator : Seq. Line : 12
Acq. Instrument : Instrument 1 Location : Vial 43
Injection Date : 11/21/2019 3:39:17 PM Inj : 1
Inj Volume : 5.000 µl
Acq. Method : D:\DATA\LWD\LWD-6-95\LWD-6-95 2019-11-21 09-11-41\VWD-OJ(1-6)-95-5-1.0ML-5UL-220NM-60MIN.M
Last changed : 11/21/2019 9:48:25 AM
Analysis Method : D:\METHOD\LSL\VWD-OJ(1-6)-95-5-1.0ML-5UL-220NM-60MIN.M
Last changed : 11/21/2019 8:11:46 PM
(modified after loading)
Additional Info : Peak(s) manually integrated

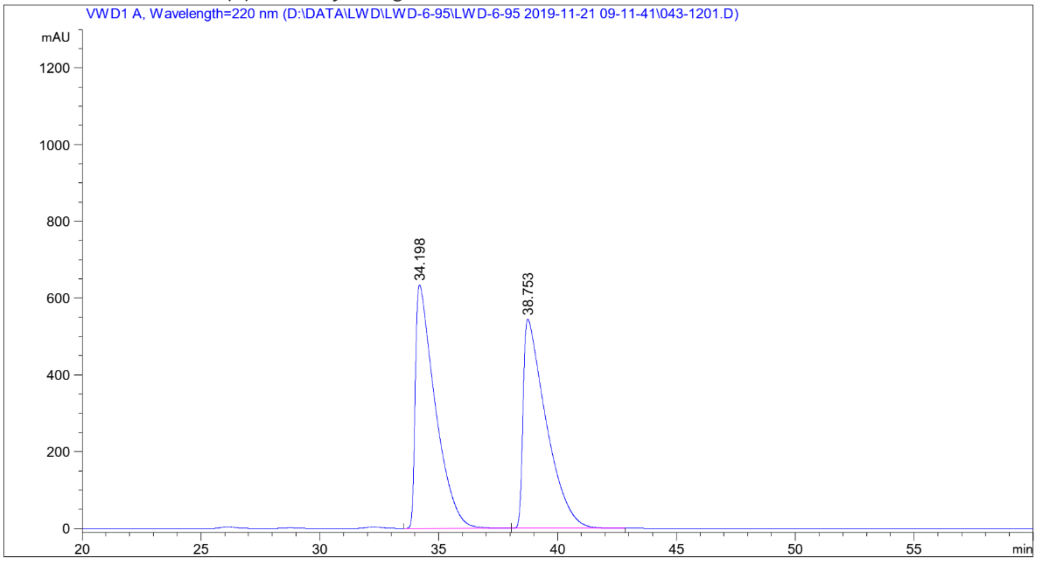

===

Area Percent Report

===

Sorted By : Signal
Multiplier : 1.0000
Dilution : 1.0000
Use Multiplier & Dilution Factor with ISTDs

Signal 1: VWD1 A, Wavelength=220 nm

Peak #	RetTime [min]	Type	Width [min]	Area [mAU*s]	Height [mAU]	Area %
1	34.198	BB	0.8309	3.67073e4	634.56793	50.1522
2	38.753	BB	0.9631	3.64846e4	544.91119	49.8478

Totals : 7.31919e4 1179.47913

Data File D:\DATA\LWD\LWD-6-95\LWD-6-95 2019-11-21 09-11-41\044-1301.D
Sample Name: LSL-5-p-Cl chiral

```
=====================================================================
Acq. Operator   :                                 Seq. Line :  13
Acq. Instrument : Instrument 1                     Location : Vial 44
Injection Date  : 11/21/2019 4:40:08 PM                 Inj :   1
                                                 Inj Volume : 5.000 µl
Acq. Method     : D:\DATA\LWD\LWD-6-95\LWD-6-95 2019-11-21 09-11-41\VWD-OJ(1-6)-95-5-1.0ML-
                  5UL-220NM-60MIN.M
Last changed    : 11/21/2019 9:48:25 AM
Analysis Method : D:\METHOD\LSL\VWD-OJ(1-6)-95-5-1.0ML-5UL-220NM-60MIN.M
Last changed    : 11/21/2019 8:13:25 PM
                  (modified after loading)
Additional Info : Peak(s) manually integrated
```

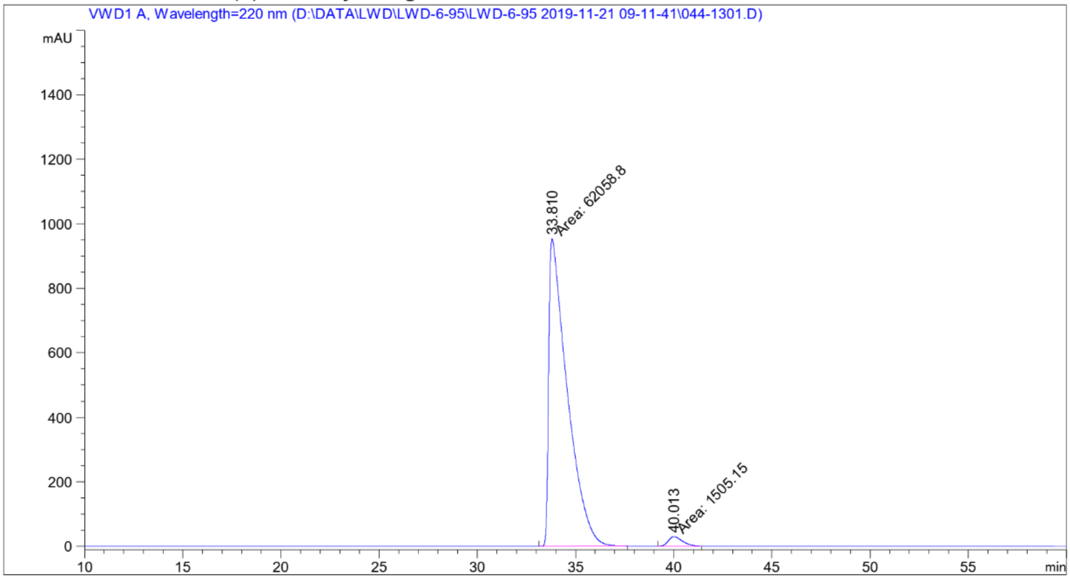


```
=====================================================================
                         Area Percent Report
=====================================================================

Sorted By             :      Signal
Multiplier            :      1.0000
Dilution              :      1.0000
Use Multiplier & Dilution Factor with ISTDs


Signal 1: VWD1 A, Wavelength=220 nm

Peak RetTime Type  Width     Area      Height     Area
  #   [min]        [min]   [mAU*s]     [mAU]        %
----|-------|----|-------|----------|----------|--------|
   1  33.810 MM    1.0858 6.20588e4  952.59686  97.6321
   2  40.013 MM    0.8466 1505.15149   29.63015   2.3679

Totals :                  6.35640e4  982.22701
```

Instrument 1 11/21/2019 8:13:32 PM   Page   1 of 2

Supplementary Figure 69. HPLC spectra for compound 3c

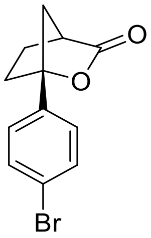

3d

Data File D:\DATA\LWD\LWD-6-95\LWD-6-95 2019-11-21 09-11-41\045-1401.D
Sample Name: LSL-5-p-Br

===
Acq. Operator   :   Seq. Line : 14
Acq. Instrument : Instrument 1   Location : Vial 45
Injection Date : 11/21/2019 5:41:01 PM   Inj :   1
                                               Inj Volume : 5.000 µl
Acq. Method   : D:\DATA\LWD\LWD-6-95\LWD-6-95 2019-11-21 09-11-41\VWD-OJ(1-6)-95-5-1.0ML-5UL-220NM-60MIN.M
Last changed   : 11/21/2019 9:48:25 AM
Analysis Method : D:\METHOD\LSL\VWD-OJ(1-6)-95-5-1.0ML-5UL-220NM-60MIN.M
Last changed   : 11/21/2019 8:14:40 PM
                  (modified after loading)
Additional Info : Peak(s) manually integrated

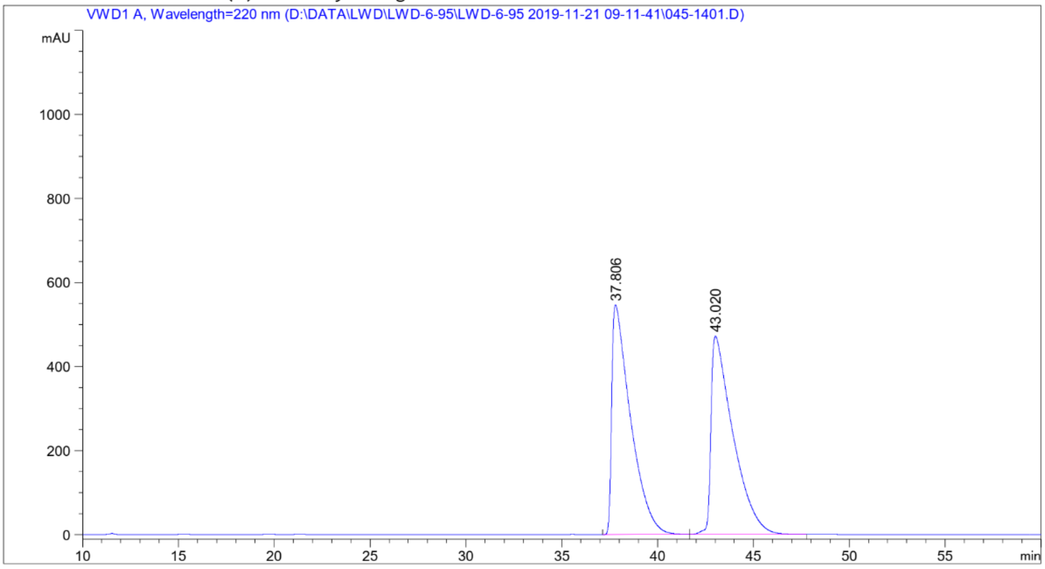

===
Area Percent Report
===

Sorted By : Signal
Multiplier : 1.0000
Dilution : 1.0000
Use Multiplier & Dilution Factor with ISTDs

Signal 1: VWD1 A, Wavelength=220 nm

Peak #	RetTime [min]	Type	Width [min]	Area [mAU*s]	Height [mAU]	Area %
1	37.806	BB	0.9786	3.71921e4	546.05408	49.8010
2	43.020	BB	1.1531	3.74894e4	471.27432	50.1990
Totals :				7.46815e4	1017.32840	

```
Data File D:\DATA\LWD\LWD-6-95\LWD-6-95 2019-11-21 09-11-41\046-1501.D
Sample Name: LSL-5-p-Br chiral

    ====================================================================
    Acq. Operator   :                                 Seq. Line :  15
    Acq. Instrument : Instrument 1                     Location : Vial 46
    Injection Date  : 11/21/2019 6:41:53 PM                 Inj :   1
                                                     Inj Volume : 5.000 µl
    Acq. Method     : D:\DATA\LWD\LWD-6-95\LWD-6-95 2019-11-21 09-11-41\VWD-OJ(1-6)-95-5-1.0ML-
                      5UL-220NM-60MIN.M
    Last changed    : 11/21/2019 9:48:25 AM
    Analysis Method : D:\METHOD\LSL\VWD-OJ(1-6)-95-5-1.0ML-5UL-220NM-60MIN.M
    Last changed    : 11/21/2019 8:16:10 PM
                      (modified after loading)
    Additional Info : Peak(s) manually integrated
```

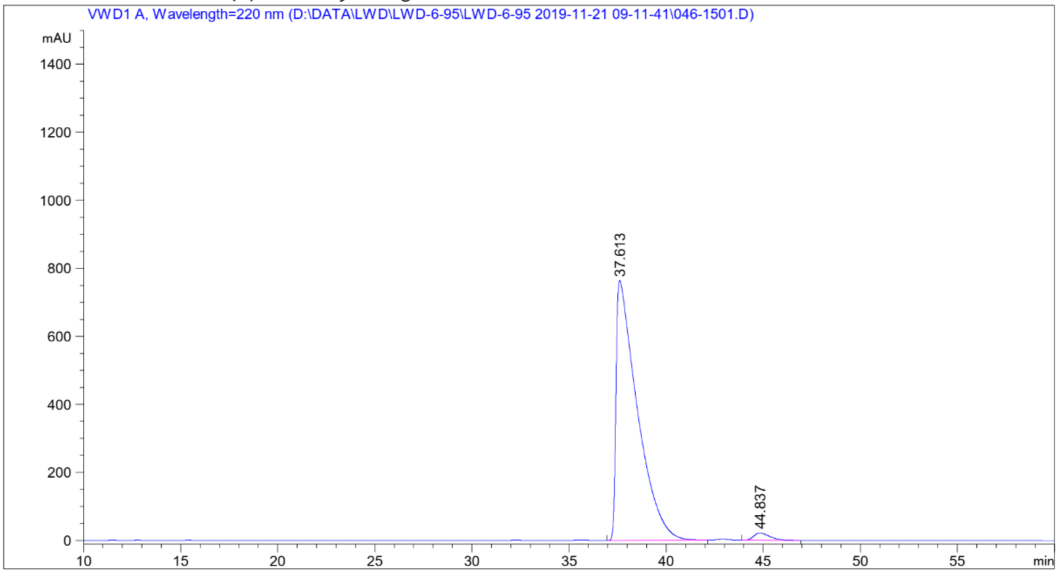


```
    ====================================================================
                             Area Percent Report
    ====================================================================

    Sorted By             :      Signal
    Multiplier            :      1.0000
    Dilution              :      1.0000
    Use Multiplier & Dilution Factor with ISTDs


    Signal 1: VWD1 A, Wavelength=220 nm

    Peak RetTime Type  Width     Area      Height     Area
      #   [min]        [min]   [mAU*s]     [mAU]        %
    ----|-------|----|-------|----------|----------|--------|
       1  37.613 BB    1.1008 5.78921e4  763.37708  97.9355
       2  44.837 BB    0.8629 1220.38513  21.34982   2.0645

    Totals :                  5.91124e4  784.72689
```


Supplementary Figure 70. HPLC spectra for compound 3d

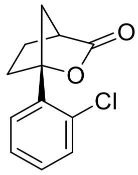

3e

Data File D:\DATA\LWD\LWD-LRH-6-45\LWD-LRH-6-45 2019-12-06 20-16-40\044-0801.D
Sample Name: LSL-5-31-6

```
=====================================================================
Acq. Operator   :                                 Seq. Line :    8
Acq. Instrument : Instrument 2                     Location : Vial 44
Injection Date  : 12/6/2019 10:46:04 PM                  Inj :    1
                                                 Inj Volume : 5.000 µl
Acq. Method     : D:\DATA\LWD\LWD-LRH-6-45\LWD-LRH-6-45 2019-12-06 20-16-40\DAD-OD(1-2)-97-3-
                  1ML-5UL-ALL-60MIN.M
Last changed    : 5/30/2018 8:39:48 AM
Analysis Method : D:\METHOD\LYH\DAD-OD(1-2)-85-15-1ML-2UL-ALL-40MIN.M
Last changed    : 12/7/2019 9:56:42 AM
                  (modified after loading)
Additional Info : Peak(s) manually integrated
```

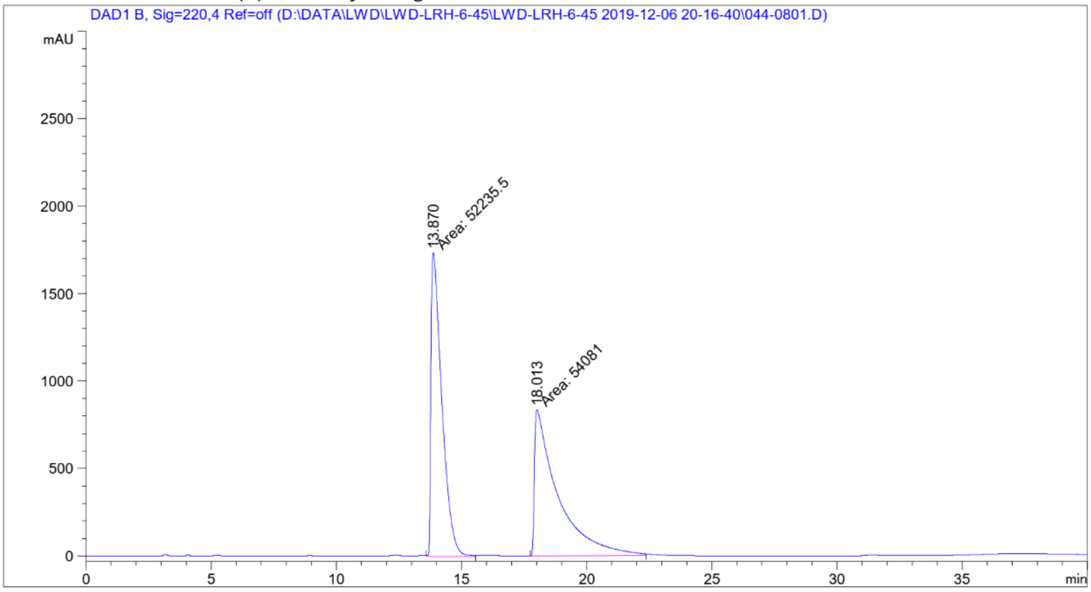


```
=====================================================================
                         Area Percent Report
=====================================================================

Sorted By             :      Signal
Multiplier            :      1.0000
Dilution              :      1.0000
Use Multiplier & Dilution Factor with ISTDs


Signal 1: DAD1 B, Sig=220,4 Ref=off

Peak RetTime Type  Width     Area      Height     Area
  #   [min]        [min]   [mAU*s]     [mAU]        %
----|-------|----|-------|----------|----------|--------|
   1  13.870 MM    0.5017 5.22355e4  1735.31006  49.1321
   2  18.013 MM    1.0793 5.40810e4   835.13312  50.8679

Totals :                  1.06317e5  2570.44318
```

Data File D:\DATA\LWD\LWD-LRH-6-45\LWD-LRH-6-45 2019-12-06 20-16-40\045-0901.D
Sample Name: LSL-o-Cl-chiral

===

Acq. Operator : Seq. Line : 9
Acq. Instrument : Instrument 2 Location : Vial 45
Injection Date : 12/6/2019 11:47:06 PM Inj : 1
Inj Volume : 5.000 µl
Acq. Method : D:\DATA\LWD\LWD-LRH-6-45\LWD-LRH-6-45 2019-12-06 20-16-40\DAD-OD(1-2)-97-3-1ML-5UL-ALL-60MIN.M
Last changed : 5/30/2018 8:39:48 AM
Analysis Method : D:\METHOD\LYH\DAD-OD(1-2)-85-15-1ML-2UL-ALL-40MIN.M
Last changed : 12/7/2019 9:56:42 AM
(modified after loading)
Additional Info : Peak(s) manually integrated

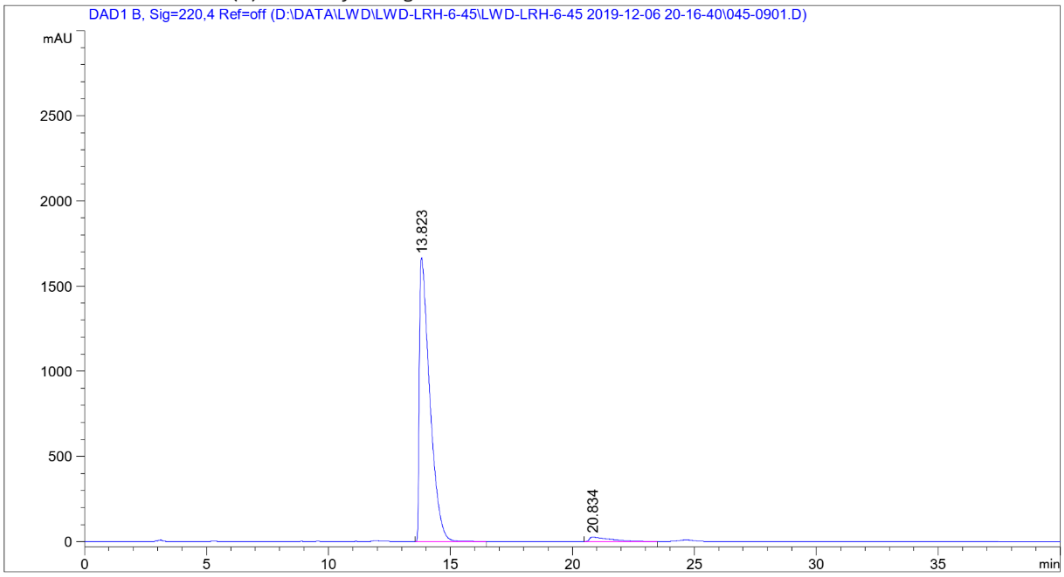

===

Area Percent Report

===

Sorted By : Signal
Multiplier : 1.0000
Dilution : 1.0000
Use Multiplier & Dilution Factor with ISTDs

Signal 1: DAD1 B, Sig=220,4 Ref=off

Peak #	RetTime [min]	Type	Width [min]	Area [mAU*s]	Height [mAU]	Area %
1	13.823	BB	0.4382	4.94277e4	1665.50256	97.0508
2	20.834	BB	0.7708	1502.01514	25.29302	2.9492

Totals : 5.09297e4 1690.79558

Supplementary Figure 71. HPLC spectra for compound 3e

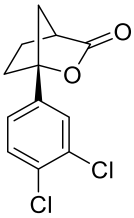

3f

Data File D:\DATA\GUAN YUQING\LK-A\LK-4-CL-TS-22 2020-07-02 13-53-00\051-1001.D
Sample Name: LSL-3,4-CL

===
Acq. Operator : Seq. Line : 10
Acq. Instrument : Instrument 2 Location : Vial 51
Injection Date : 7/2/2020 11:03:28 PM Inj : 1
Inj Volume : 5.000 µl
Acq. Method : D:\DATA\GUAN YUQING\LK-A\LK-4-CL-TS-22 2020-07-02 13-53-00\DAD-OJ(1-2)-95-5-1ML-5UL-ALL-60MIN.M
Last changed : 7/2/2020 5:53:54 PM
Analysis Method : D:\METHOD\LSL\DAD-OJ(1-2)-95-5-1ML-5UL-ALL-60MIN.M
Last changed : 7/3/2020 8:56:58 AM
(modified after loading)
Additional Info : Peak(s) manually integrated

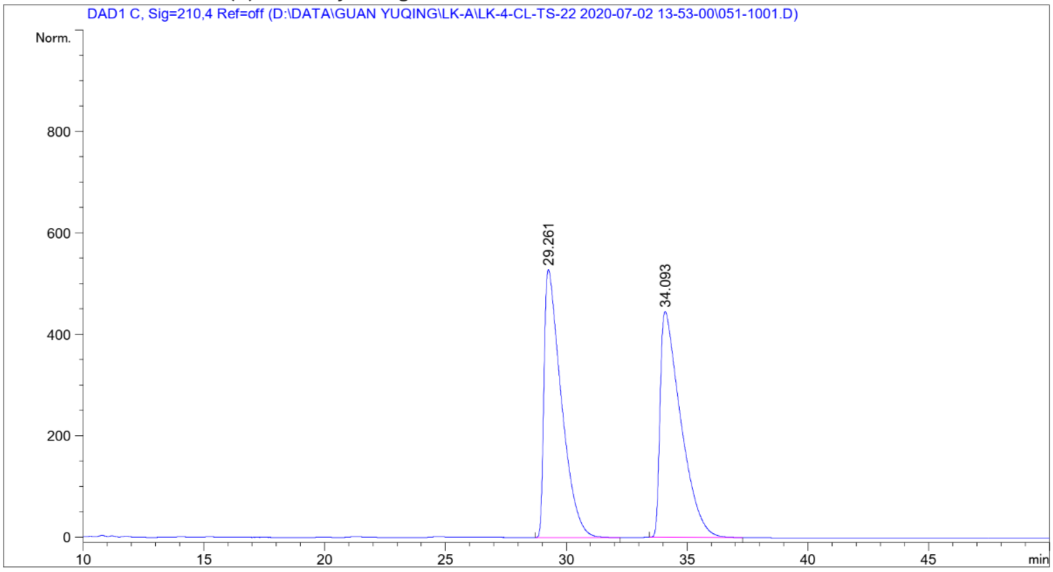

===
Area Percent Report
===

Sorted By : Signal
Multiplier : 1.0000
Dilution : 1.0000
Use Multiplier & Dilution Factor with ISTDs

Signal 1: DAD1 C, Sig=210,4 Ref=off

Peak #	RetTime [min]	Type	Width [min]	Area [mAU*s]	Height [mAU]	Area %
1	29.261	BB	0.7147	2.62919e4	528.20856	49.8509
2	34.093	BB	0.8272	2.64492e4	445.15707	50.1491

Totals : 5.27410e4 973.36563

Data File D:\DATA\GUAN YUQING\LK-A\LK-4-CL-TS-22 2020-07-02 13-53-00\052-1101.D
Sample Name: LSL-3,4-CL-C

```
=====================================================================
Acq. Operator   :                                 Seq. Line :  11
Acq. Instrument : Instrument 2                     Location : Vial 52
Injection Date  : 7/3/2020 12:04:31 AM                  Inj :   1
                                                 Inj Volume : 5.000 µl
Acq. Method     : D:\DATA\GUAN YUQING\LK-A\LK-4-CL-TS-22 2020-07-02 13-53-00\DAD-OJ(1-2)-95-5
                  -1ML-5UL-ALL-60MIN.M
Last changed    : 7/2/2020 5:53:54 PM
Analysis Method : D:\METHOD\LSL\DAD-OJ(1-2)-95-5-1ML-5UL-ALL-60MIN.M
Last changed    : 7/3/2020 8:58:28 AM
                  (modified after loading)
Additional Info : Peak(s) manually integrated
```

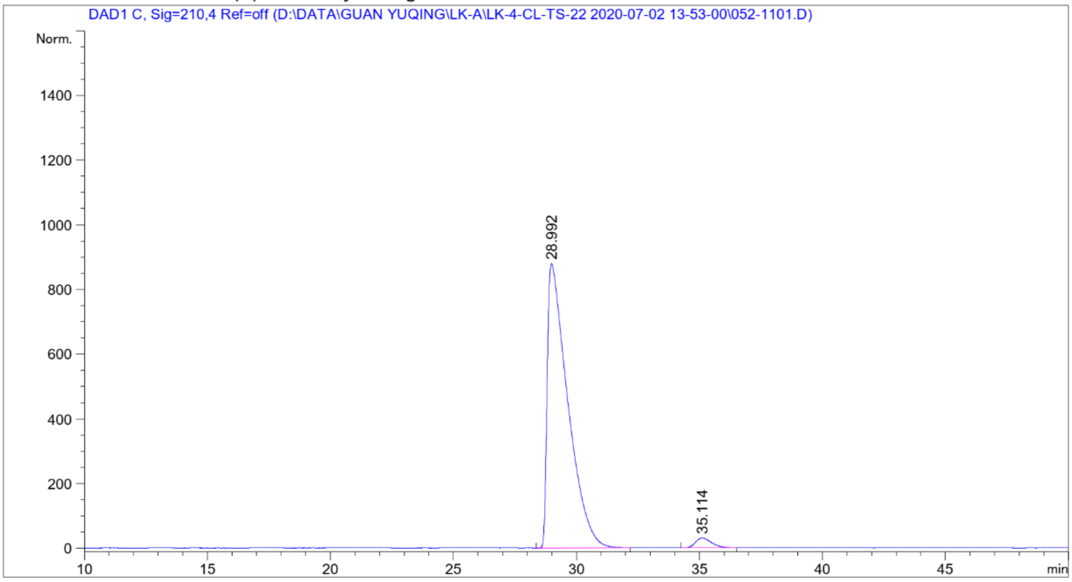


```
=====================================================================
                         Area Percent Report
=====================================================================

Sorted By             :      Signal
Multiplier            :      1.0000
Dilution              :      1.0000
Use Multiplier & Dilution Factor with ISTDs


Signal 1: DAD1 C, Sig=210,4 Ref=off

Peak RetTime Type  Width     Area      Height     Area
  #   [min]        [min]   [mAU*s]     [mAU]        %
----|-------|----|-------|----------|----------|--------|
   1  28.992 BB    0.7913 5.07207e4  878.89783 97.3079
   2  35.114 BB    0.6791 1403.23340   30.48154   2.6921

Totals :                  5.21240e4  909.37937
```

Instrument 2 7/3/2020 8:58:32 AM   Page   1 of 2

Supplementary Figure 72. HPLC spectra for compound 3f

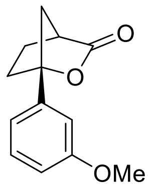

3g

```
Data File D:\DATA\LSL\LSL-5-22\LSL-5-22 2019-11-23 10-26-57\032-1101.D
Sample Name: lsl-m-MeO-rac

=====================================================================
Acq. Operator   :                                 Seq. Line :  11
Acq. Instrument : Instrument 1                     Location : Vial 32
Injection Date  : 11/23/2019 5:43:28 PM                 Inj :   1
                                                Inj Volume : 5.000 µl
Acq. Method     : D:\DATA\LSL\LSL-5-22\LSL-5-22 2019-11-23 10-26-57\VWD-OD(1-2)-97-3-1ML-5UL-
                  220NM-80MIN.M
Last changed    : 11/23/2019 2:57:28 PM
Analysis Method : D:\METHOD\LGY\VWD-OD(1-2)--85-15ML-5UL-210NM-35MIN.M
Last changed    : 11/23/2019 8:56:14 PM
                  (modified after loading)
Additional Info : Peak(s) manually integrated
```

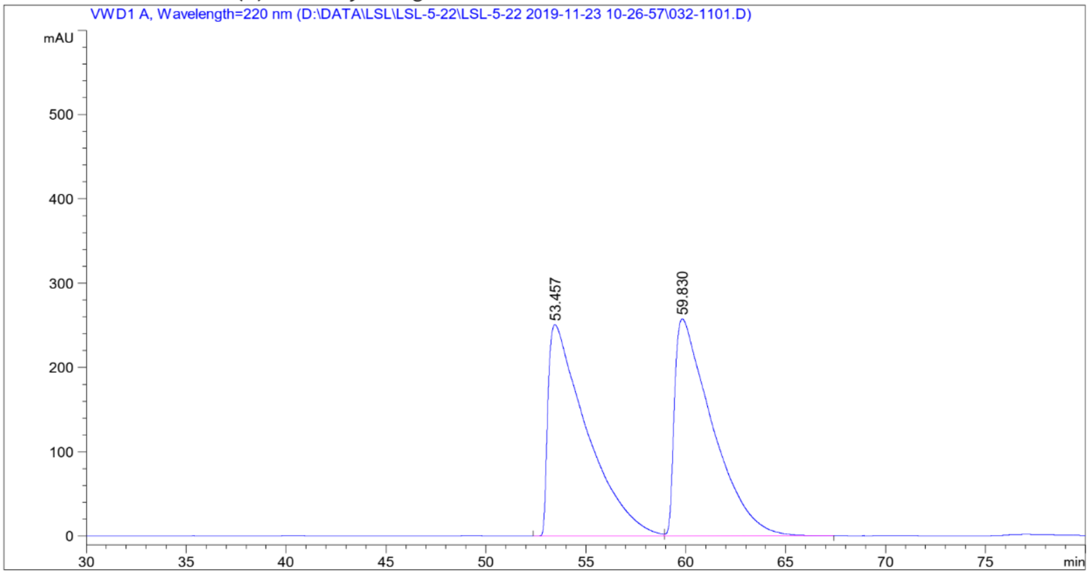


```
=====================================================================
                         Area Percent Report
=====================================================================

Sorted By             :      Signal
Multiplier            :      1.0000
Dilution              :      1.0000
Use Multiplier & Dilution Factor with ISTDs


Signal 1: VWD1 A, Wavelength=220 nm

Peak RetTime Type  Width     Area      Height     Area
  #   [min]        [min]   [mAU*s]     [mAU]        %
----|-------|----|-------|----------|----------|--------|
   1  53.457 BV    1.8282 3.36645e4  250.45319  49.9591
   2  59.830 VB    1.7901 3.37196e4  257.27267  50.0409

Totals :                  6.73842e4  507.72586
```

```
Data File D:\DATA\LSL\LSL-5-22\LSL-5-22 2019-11-23 10-26-57\042-1201.D
Sample Name: lsl-m-MeO-C

    =====================================================================
    Acq. Operator   :                                Seq. Line :  12
    Acq. Instrument : Instrument 1                    Location : Vial 42
    Injection Date  : 11/23/2019 7:04:18 PM                Inj :   1
                                                    Inj Volume : 5.000 µl
    Acq. Method     : D:\DATA\LSL\LSL-5-22\LSL-5-22 2019-11-23 10-26-57\VWD-OD(1-2)-97-3-1ML-5UL-
                      220NM-80MIN.M
    Last changed    : 11/23/2019 2:57:28 PM
    Analysis Method : D:\METHOD\LGY\VWD-OD(1-2)--85-15ML-5UL-210NM-35MIN.M
    Last changed    : 11/23/2019 8:56:14 PM
                      (modified after loading)
    Additional Info : Peak(s) manually integrated
```

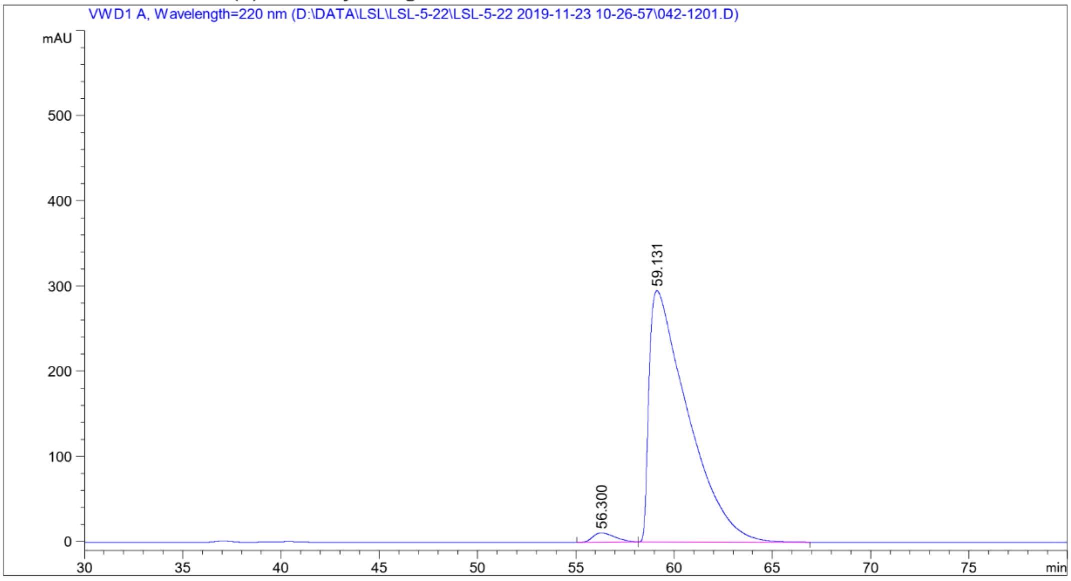


```
    =====================================================================
                             Area Percent Report
    =====================================================================

    Sorted By             :      Signal
    Multiplier            :      1.0000
    Dilution              :      1.0000
    Use Multiplier & Dilution Factor with ISTDs


    Signal 1: VWD1 A, Wavelength=220 nm

    Peak RetTime Type  Width      Area      Height     Area
      #   [min]        [min]    [mAU*s]     [mAU]        %
    ----|-------|----|-------|----------|----------|--------|
       1  56.300 BB    1.1675  852.91675   11.03760   2.0675
       2  59.131 BB    1.9241 4.04004e4   294.94019  97.9325

    Totals :                  4.12533e4   305.97779
```


Supplementary Figure 73. HPLC spectra for compound 3g

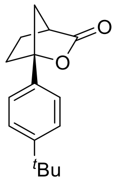

3h

Data File D:\DATA\LSL\LSL-5-22\LSL-5-22 2019-11-23 10-26-57\033-1501.D
Sample Name: lsl-p-tbu-rac-2

```
=====================================================================
Acq. Operator   :                               Seq. Line :  15
Acq. Instrument : Instrument 1                   Location : Vial 33
Injection Date  : 11/23/2019 10:26:57 PM               Inj :   1
                                              Inj Volume : 5.000 µl
Acq. Method     : D:\DATA\LSL\LSL-5-22\LSL-5-22 2019-11-23 10-26-57\VWD-OD(1-2)-97-3-1ML-5UL-
                  220NM-60MIN.M
Last changed    : 11/23/2019 10:05:59 AM
Analysis Method : D:\METHOD\LWD\VWD-OD(1-2)-85-15-0.15ML-3UL-210NM-80MIN.M
Last changed    : 11/24/2019 9:42:28 PM
                  (modified after loading)
Additional Info : Peak(s) manually integrated
```

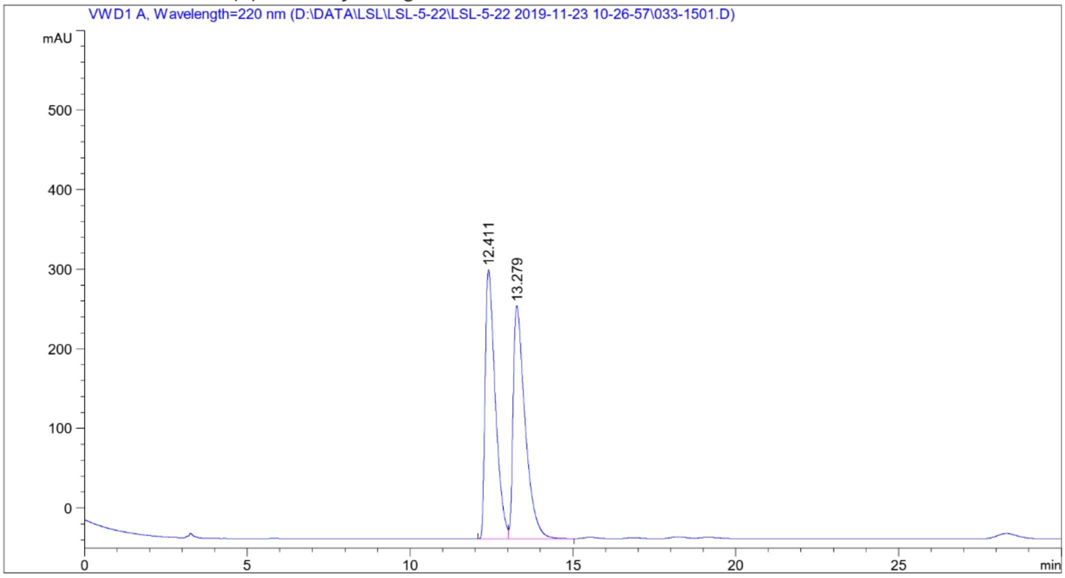


```
=====================================================================
                          Area Percent Report
=====================================================================

Sorted By             :      Signal
Multiplier            :      1.0000
Dilution              :      1.0000
Use Multiplier & Dilution Factor with ISTDs


Signal 1: VWD1 A, Wavelength=220 nm

Peak RetTime Type  Width     Area      Height     Area
  #   [min]        [min]   [mAU*s]     [mAU]        %
----|-------|----|-------|----------|----------|--------|
   1  12.411 BV    0.3292 7332.41260  337.89899  49.5439
   2  13.279 VB    0.3810 7467.42383  292.37897  50.4561

Totals :                  1.47998e4   630.27795
```

```
Data File D:\DATA\LSL\LSL-5-22\LSL-5-22 2019-11-23 10-26-57\043-1001.D
Sample Name: LSL-p-tBu-c

    =====================================================================
    Acq. Operator   :                                 Seq. Line :  10
    Acq. Instrument : Instrument 1                     Location : Vial 43
    Injection Date  : 11/23/2019 4:42:38 PM                 Inj :   1
                                                     Inj Volume : 5.000 µl
    Acq. Method     : D:\DATA\LSL\LSL-5-22\LSL-5-22 2019-11-23 10-26-57\VWD-OD(1-2)-97-3-1ML-5UL-
                      220NM-60MIN.M
    Last changed    : 11/23/2019 10:05:59 AM
    Analysis Method : D:\METHOD\LGY\VWD-OD(1-2)--85-15ML-5UL-210NM-35MIN.M
    Last changed    : 11/23/2019 8:59:50 PM
                      (modified after loading)
    Additional Info : Peak(s) manually integrated
```

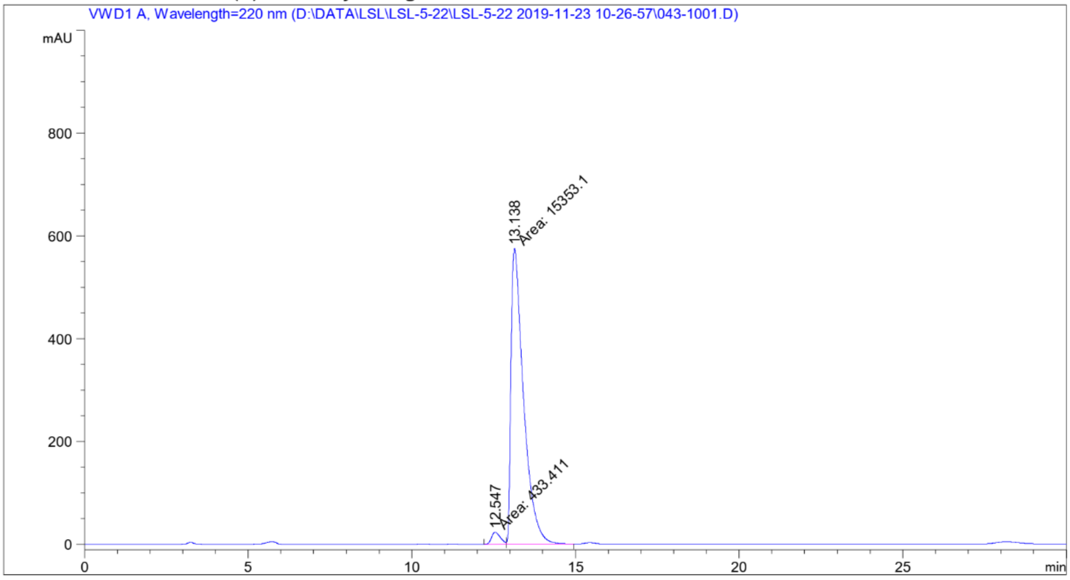


```
    =====================================================================
                         Area Percent Report
    =====================================================================

    Sorted By             :      Signal
    Multiplier            :      1.0000
    Dilution              :      1.0000
    Use Multiplier & Dilution Factor with ISTDs


    Signal 1: VWD1 A, Wavelength=220 nm

    Peak RetTime Type  Width     Area      Height     Area
      #   [min]        [min]   [mAU*s]     [mAU]        %
    ----|-------|----|-------|----------|----------|--------|
       1  12.547 MF    0.3098  433.41061   23.31311   2.7454
       2  13.138 FM    0.4450 1.53531e4  575.05884  97.2546

    Totals :                  1.57865e4  598.37195
```


Supplementary Figure 74. HPLC spectra for compound 3h

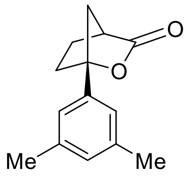

3i

Data File D:\DATA\LG\201912\P-O-BPE 2019-12-13 10-45-12\053-0701.D
Sample Name: LSL-3,5-ME-RAC

```
=====================================================================
Acq. Operator   :                                 Seq. Line :    7
Acq. Instrument : Instrument 1                     Location : Vial 53
Injection Date  : 12/13/2019 12:45:28 PM                Inj :    1
                                                Inj Volume : 5.000 µl
Acq. Method     : D:\DATA\LG\201912\P-O-BPE 2019-12-13 10-45-12\VWD-AD(1-2)-97-3-1ML-5UL-
                  210NM-60MIN.M
Last changed    : 9/29/2019 6:56:04 PM
Analysis Method : D:\METHOD\LWD\VWD-IA(1-2)-95-5-1ML-2UL-210NM-60MIN.M
Last changed    : 12/13/2019 4:10:56 PM
                  (modified after loading)
Additional Info : Peak(s) manually integrated
```

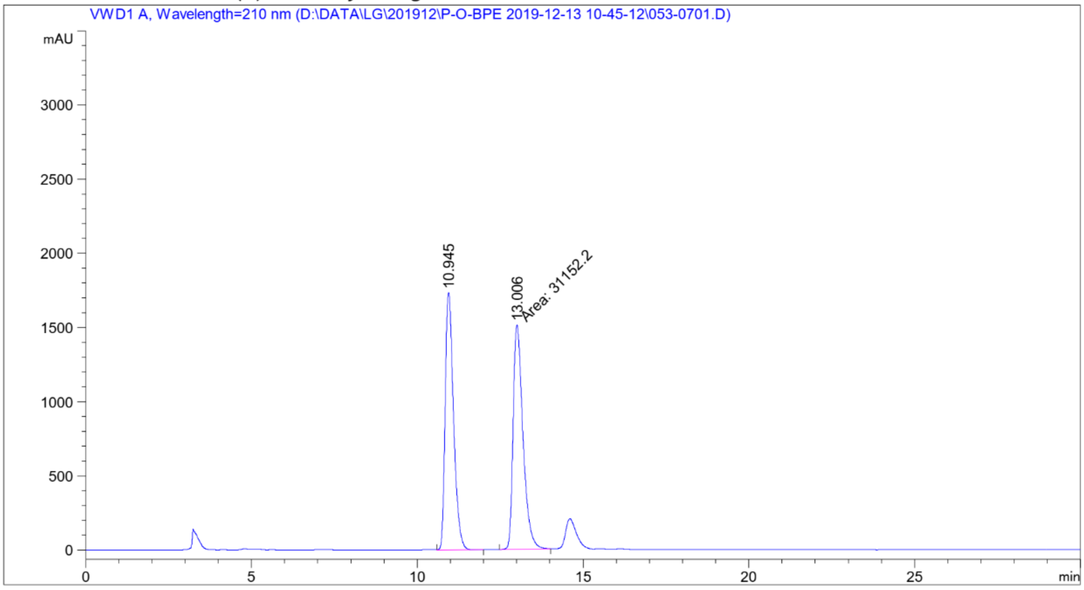


```
=====================================================================
                         Area Percent Report
=====================================================================

Sorted By             :      Signal
Multiplier            :      1.0000
Dilution              :      1.0000
Use Multiplier & Dilution Factor with ISTDs


Signal 1: VWD1 A, Wavelength=210 nm
```

Peak #	RetTime [min]	Type	Width [min]	Area [mAU*s]	Height [mAU]	Area %
1	10.945	VB	0.2695	3.03767e4	1732.83240	49.3698
2	13.006	MM	0.3437	3.11522e4	1510.60535	50.6302

Totals : 6.15289e4 3243.43774

Data File D:\DATA\LG\201912\P-O-BPE 2019-12-13 10-45-12\054-0801.D
Sample Name: LSL-3,5-ME-C

===

Acq. Operator   :   Seq. Line :   8
Acq. Instrument : Instrument 1   Location : Vial 54
Injection Date : 12/13/2019 1:46:20 PM   Inj :   1
                                               Inj Volume : 5.000 µl
Acq. Method   : D:\DATA\LG\201912\P-O-BPE 2019-12-13 10-45-12\VWD-AD(1-2)-97-3-1ML-5UL-210NM-60MIN.M
Last changed   : 9/29/2019 6:56:04 PM
Analysis Method : D:\METHOD\LWD\VWD-IA(1-2)-95-5-1ML-2UL-210NM-60MIN.M
Last changed   : 12/13/2019 4:13:04 PM
                  (modified after loading)
Additional Info : Peak(s) manually integrated

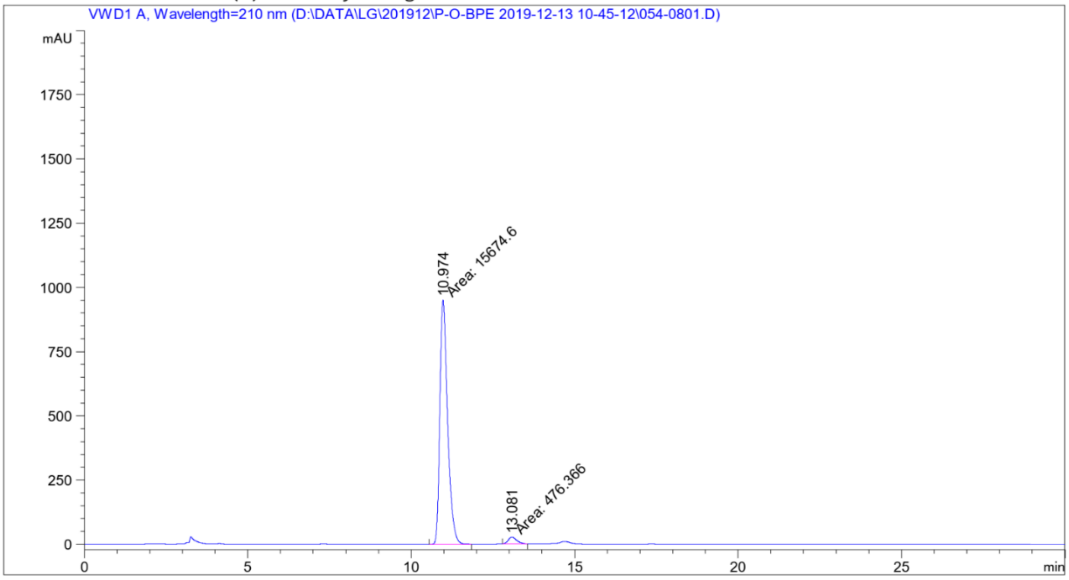

===

Area Percent Report

===

Sorted By : Signal
Multiplier : 1.0000
Dilution : 1.0000
Use Multiplier & Dilution Factor with ISTDs

Signal 1: VWD1 A, Wavelength=210 nm

Peak #	RetTime [min]	Type	Width [min]	Area [mAU*s]	Height [mAU]	Area %
1	10.974	MM	0.2750	1.56746e4	950.09454	97.0505
2	13.081	MM	0.3022	476.36563	26.26962	2.9495
Totals :				1.61510e4	976.36416	

Supplementary Figure 75. HPLC spectra for compound 3i

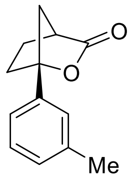

3j

Data File D:\DATA\LSL\LSL-5-1230\LSL-5-1230 2019-12-30 21-57-33\043-0501.D
Sample Name: LSL-5-m-Me-rac

```
=====================================================================
Acq. Operator   :                               Seq. Line :    5
Acq. Instrument : Instrument 2                   Location : Vial 43
Injection Date  : 12/31/2019 12:22:42 AM              Inj :    1
                                               Inj Volume : 5.000 µl
Acq. Method     : D:\DATA\LSL\LSL-5-1230\LSL-5-1230 2019-12-30 21-57-33\DAD-OD(1-2)-95-5-1ML-
                  5UL-ALL-60MIN.M
Last changed    : 6/21/2018 2:29:29 PM
Analysis Method : D:\METHOD\LSL\DAD-OD(1-2)-85-15-1ML-5UL-ALL-40MIN.M
Last changed    : 12/31/2019 9:51:04 AM
                  (modified after loading)
Additional Info : Peak(s) manually integrated
```

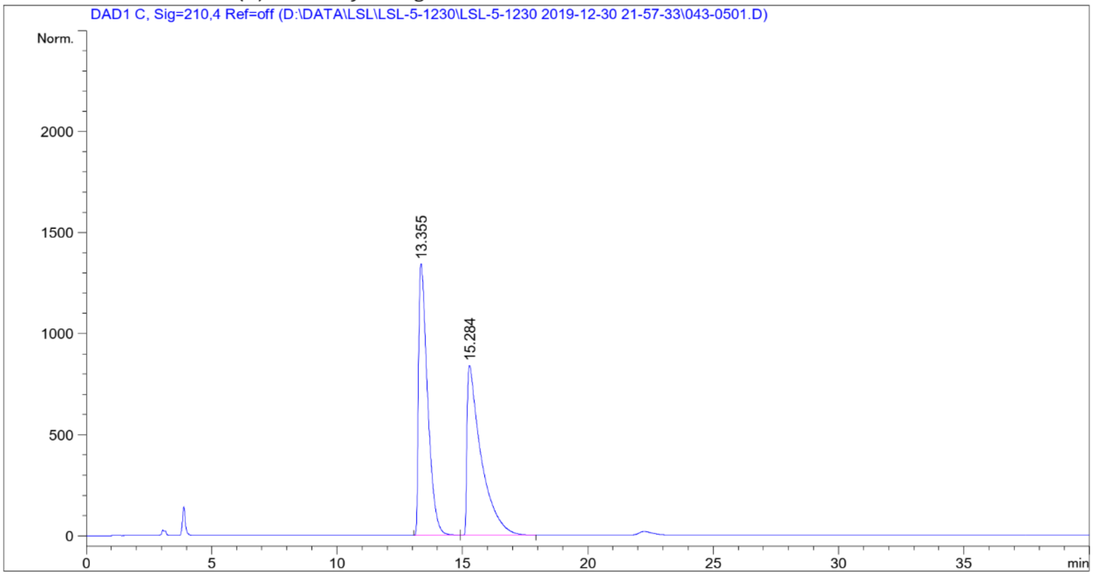


```
=====================================================================
                         Area Percent Report
=====================================================================

Sorted By             :      Signal
Multiplier            :      1.0000
Dilution              :      1.0000
Use Multiplier & Dilution Factor with ISTDs


Signal 1: DAD1 C, Sig=210,4 Ref=off
```

Peak #	RetTime [min]	Type	Width [min]	Area [mAU*s]	Height [mAU]	Area %
1	13.355	BB	0.3755	3.35992e4	1344.66296	50.6850
2	15.284	BB	0.5365	3.26911e4	839.24042	49.3150

Totals : 6.62903e4 2183.90338

Data File D:\DATA\LSL\LSL-5-1230\LSL-5-1230-2 2019-12-31 10-47-02\044-0301.D
Sample Name: LSL-5-m-Me-c

===

Acq. Operator   :   Seq. Line :   3
Acq. Instrument : Instrument 2   Location : Vial 44
Injection Date : 12/31/2019 11:10:56 AM   Inj :   1
                                               Inj Volume : 5.000 µl
Acq. Method   : D:\DATA\LSL\LSL-5-1230\LSL-5-1230-2 2019-12-31 10-47-02\DAD-OD(1-2)-95-5-1ML-5UL-ALL-60MIN.M
Last changed   : 6/21/2018 2:29:29 PM
Analysis Method : D:\METHOD\LSL\DAD-OD(1-2)-85-15-1ML-5UL-ALL-40MIN.M
Last changed   : 12/31/2019 9:51:04 AM
                  (modified after loading)
Additional Info : Peak(s) manually integrated

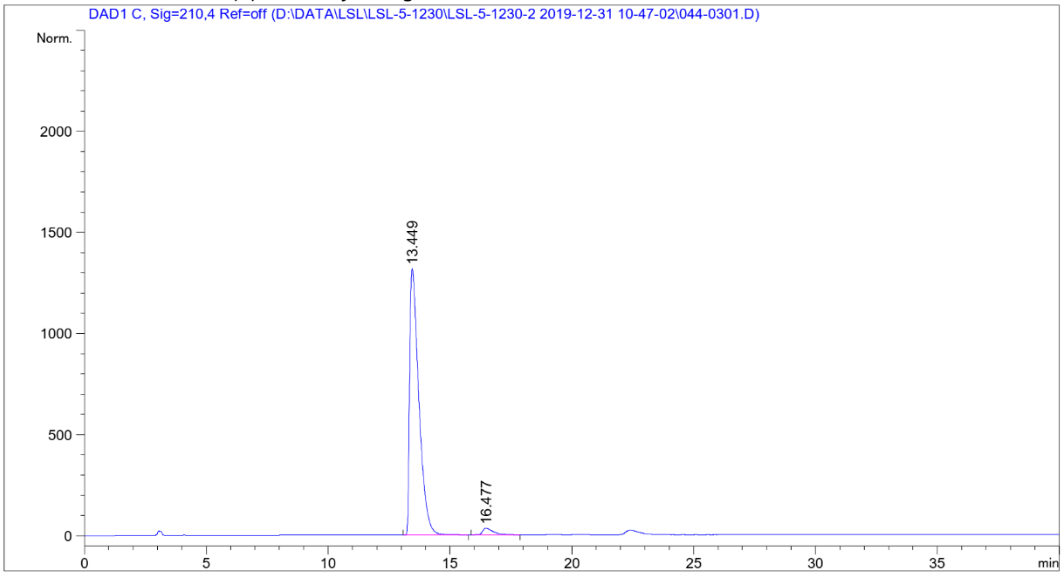

===

Area Percent Report

===

Sorted By : Signal
Multiplier : 1.0000
Dilution : 1.0000
Use Multiplier & Dilution Factor with ISTDs

Signal 1: DAD1 C, Sig=210,4 Ref=off

Peak #	RetTime [min]	Type	Width [min]	Area [mAU*s]	Height [mAU]	Area %
1	13.449	BB	0.3981	3.45537e4	1317.16370	97.1924
2	16.477	BB	0.4600	998.16687	32.17122	2.8076

Totals : 3.55518e4 1349.33491

Supplementary Figure 76. HPLC spectra for compound 3j

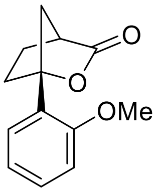

3k

```
Data File D:\DATA\LSL\LSL-5-1230\LSL-5-1230 2019-12-30 21-57-33\041-0301.D
Sample Name: LSL-5-O-MeO-RAC

    =====================================================================
    Acq. Operator    :                                   Seq. Line :    3
    Acq. Instrument  : Instrument 2                       Location : Vial 41
    Injection Date   : 12/30/2019 10:20:40 PM                  Inj :    1
                                                        Inj Volume : 5.000 µl
    Acq. Method      : D:\DATA\LSL\LSL-5-1230\LSL-5-1230 2019-12-30 21-57-33\DAD-OD(1-2)-95-5-1ML-
                       5UL-ALL-60MIN.M
    Last changed     : 6/21/2018 2:29:29 PM
    Analysis Method  : D:\METHOD\LSL\DAD-OD(1-2)-85-15-1ML-5UL-ALL-40MIN.M
    Last changed     : 12/31/2019 9:49:31 AM
                       (modified after loading)
    Additional Info  : Peak(s) manually integrated
```

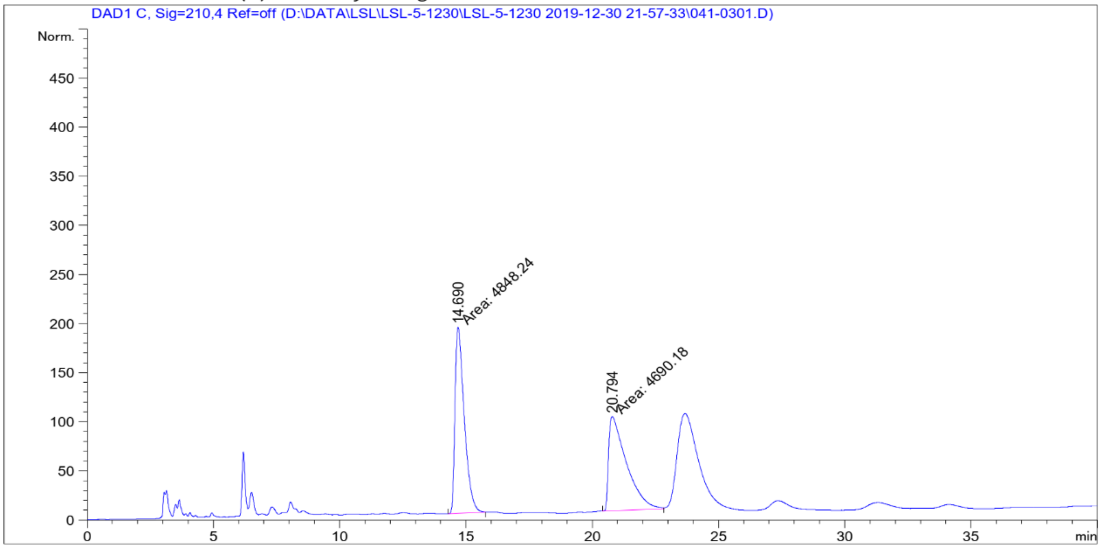


```
    =====================================================================
                             Area Percent Report
    =====================================================================

    Sorted By             :      Signal
    Multiplier            :      1.0000
    Dilution              :      1.0000
    Use Multiplier & Dilution Factor with ISTDs


    Signal 1: DAD1 C, Sig=210,4 Ref=off

    Peak RetTime Type  Width     Area      Height     Area
      #   [min]        [min]   [mAU*s]     [mAU]        %
    ----|-------|----|-------|----------|----------|--------|
       1  14.690 MM    0.4265 4848.23779  189.45085  50.8286
       2  20.794 MM    0.8141 4690.17529   96.02020  49.1714

    Totals :                  9538.41309  285.47105
```

Data File D:\DATA\LSL\LSL-5-1230\LSL-5-1230 2019-12-30 21-57-33\042-0401.D
Sample Name: LSL-5-O-MeO-C

===

Acq. Operator : Seq. Line : 4
Acq. Instrument : Instrument 2 Location : Vial 42
Injection Date : 12/30/2019 11:21:41 PM Inj : 1
Inj Volume : 5.000 µl
Acq. Method : D:\DATA\LSL\LSL-5-1230\LSL-5-1230 2019-12-30 21-57-33\DAD-OD(1-2)-95-5-1ML-5UL-ALL-60MIN.M
Last changed : 6/21/2018 2:29:29 PM
Analysis Method : D:\METHOD\LSL\DAD-OD(1-2)-85-15-1ML-5UL-ALL-40MIN.M
Last changed : 12/31/2019 9:51:04 AM
(modified after loading)
Additional Info : Peak(s) manually integrated

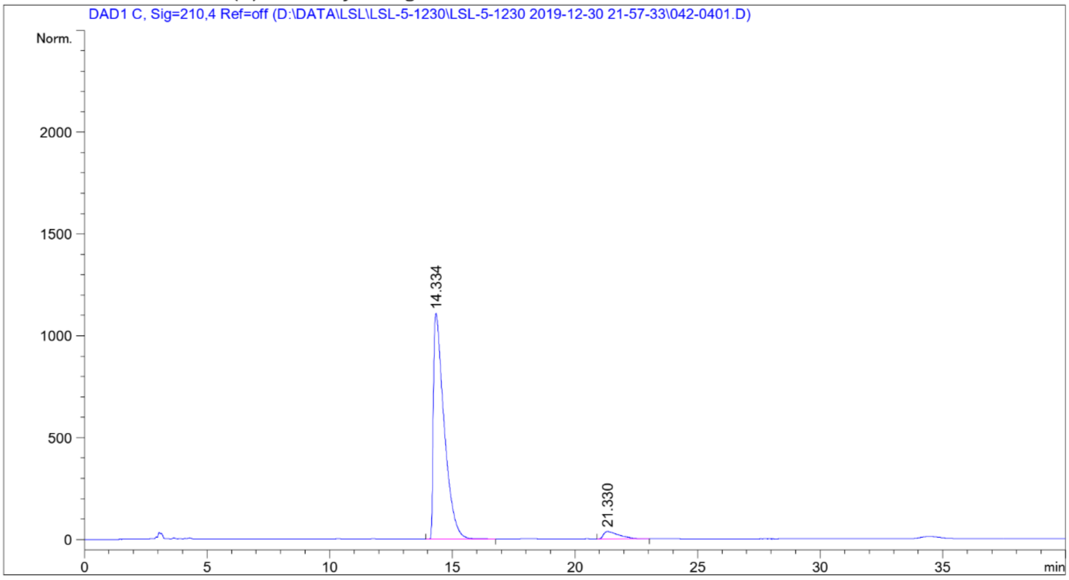

===

Area Percent Report

===

Sorted By : Signal
Multiplier : 1.0000
Dilution : 1.0000
Use Multiplier & Dilution Factor with ISTDs

Signal 1: DAD1 C, Sig=210,4 Ref=off

Peak #	RetTime [min]	Type	Width [min]	Area [mAU*s]	Height [mAU]	Area %
1	14.334	BB	0.4513	3.37430e4	1107.90454	95.3913
2	21.330	BB	0.6029	1630.22961	36.51231	4.6087

Totals : 3.53732e4 1144.41685

Instrument 2 12/31/2019 9:51:09 AM Page 1 of 2

Supplementary Figure 77. HPLC spectra for compound 3k

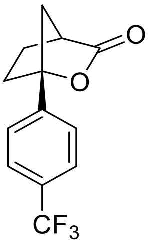

3l

```
Data File D:\DATA\LSL\LSL-5-22\LSL-5-22 2019-11-23 10-26-57\034-2201.D
Sample Name: LSL-p-CF3-rac

    =====================================================================
    Acq. Operator   :                                    Seq. Line :  22
    Acq. Instrument : Instrument 1                        Location : Vial 34
    Injection Date  : 11/24/2019 3:53:25 AM                    Inj :   1
                                                        Inj Volume : 5.000 µl
    Acq. Method     : D:\DATA\LSL\LSL-5-22\LSL-5-22 2019-11-23 10-26-57\VWD-OD(1-2)-98-2-0.5ML-
                      5UL-220NM-60MIN.M
    Last changed    : 11/23/2019 10:15:54 AM
    Analysis Method : D:\METHOD\LWD\VWD-OD(1-2)-85-15-0.15ML-3UL-210NM-80MIN.M
    Last changed    : 11/24/2019 9:32:00 PM
                      (modified after loading)
    Additional Info : Peak(s) manually integrated
```

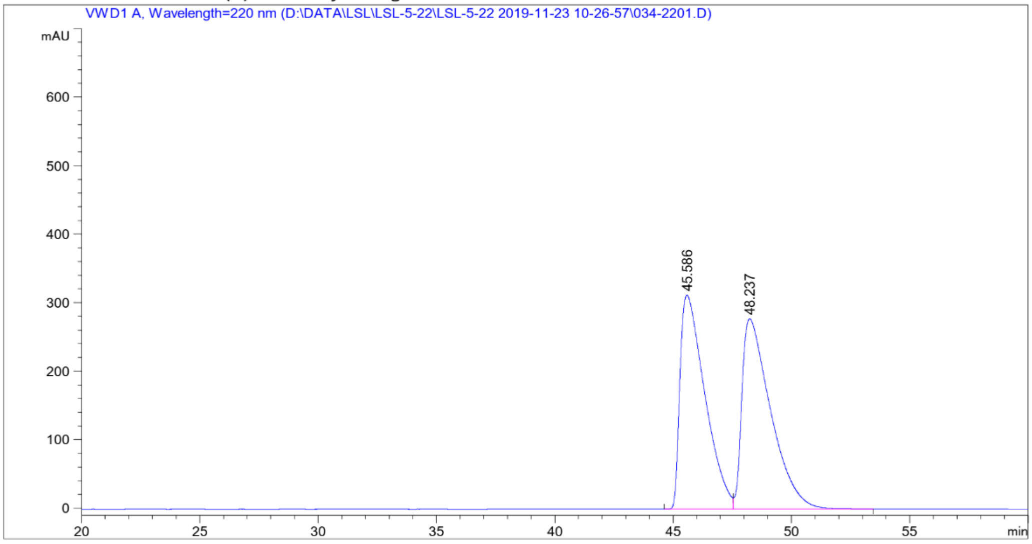


```
    =====================================================================
                             Area Percent Report
    =====================================================================

    Sorted By             :      Signal
    Multiplier            :      1.0000
    Dilution              :      1.0000
    Use Multiplier & Dilution Factor with ISTDs


    Signal 1: VWD1 A, Wavelength=220 nm

    Peak RetTime Type  Width     Area      Height     Area
      #   [min]        [min]   [mAU*s]     [mAU]        %
    ----|-------|----|-------|----------|----------|--------|
       1  45.586 BV    1.0888 2.27916e4  312.17868  49.3219
       2  48.237 VB    1.2302 2.34183e4  277.59415  50.6781

    Totals :                  4.62100e4  589.77283
```

Data File D:\DATA\LSL\LSL-5-22\LSL-5-22 2019-11-23 10-26-57\044-2301.D
Sample Name: LSL-p-CF3-c

===

Acq. Operator   :   Seq. Line : 23
Acq. Instrument : Instrument 1   Location : Vial 44
Injection Date : 11/24/2019 4:54:14 AM   Inj :   1
                                                   Inj Volume : 5.000 µl
Acq. Method   : D:\DATA\LSL\LSL-5-22\LSL-5-22 2019-11-23 10-26-57\VWD-OD(1-2)-98-2-0.5ML-5UL-220NM-60MIN.M
Last changed   : 11/23/2019 10:15:54 AM
Analysis Method : D:\METHOD\LWD\VWD-OD(1-2)-85-15-0.15ML-3UL-210NM-80MIN.M
Last changed   : 11/24/2019 9:34:17 PM
                  (modified after loading)
Additional Info : Peak(s) manually integrated

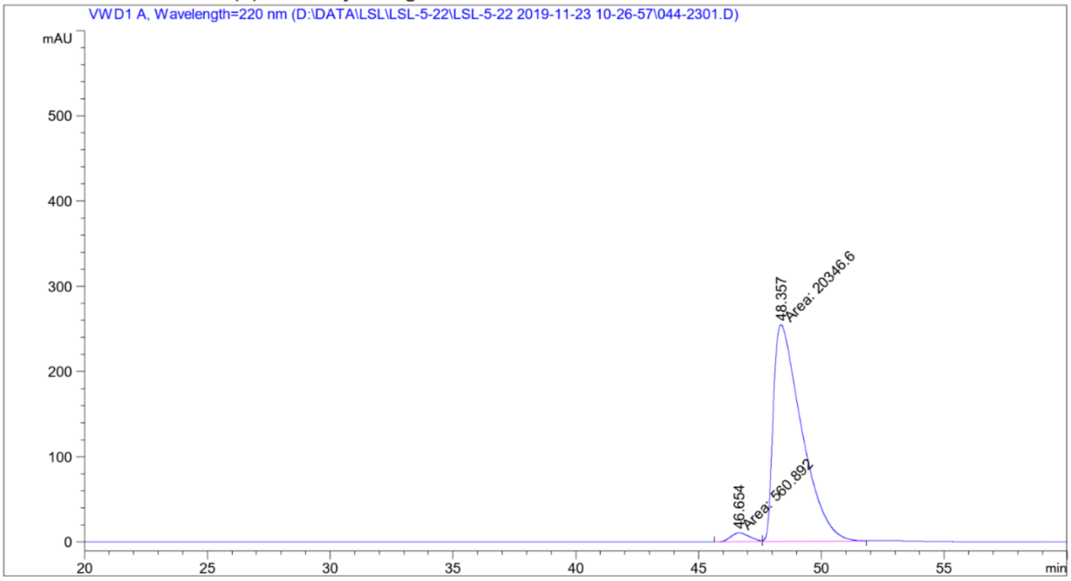

===

Area Percent Report

===

Sorted By : Signal
Multiplier : 1.0000
Dilution : 1.0000
Use Multiplier & Dilution Factor with ISTDs

Signal 1: VWD1 A, Wavelength=220 nm

Peak #	RetTime [min]	Type	Width [min]	Area [mAU*s]	Height [mAU]	Area %
1	46.654	MF	0.9033	560.89240	10.34876	2.6827
2	48.357	FM	1.3342	2.03466e4	254.16226	97.3173

Totals : 2.09075e4 264.51102

Supplementary Figure 78. HPLC spectra for compound 3l

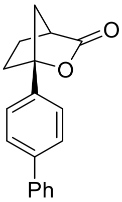

3m

Data File D:\DATA\LSL\LSL-5-22\LSL-5-22 2019-11-23 10-26-57\035-1901.D
Sample Name: LSL-Ph-rac

===

Acq. Operator : Seq. Line : 19
Acq. Instrument : Instrument 1 Location : Vial 35
Injection Date : 11/24/2019 1:40:37 AM Inj : 1
Inj Volume : 5.000 µl
Acq. Method : D:\DATA\LSL\LSL-5-22\LSL-5-22 2019-11-23 10-26-57\VWD-OD(1-2)-95-5-1ML-5UL-254NM-60MIN.M
Last changed : 11/23/2019 10:42:24 PM
Analysis Method : D:\METHOD\LWD\VWD-OD(1-2)-85-15-0.15ML-3UL-210NM-80MIN.M
Last changed : 11/24/2019 9:35:42 PM
(modified after loading)
Additional Info : Peak(s) manually integrated

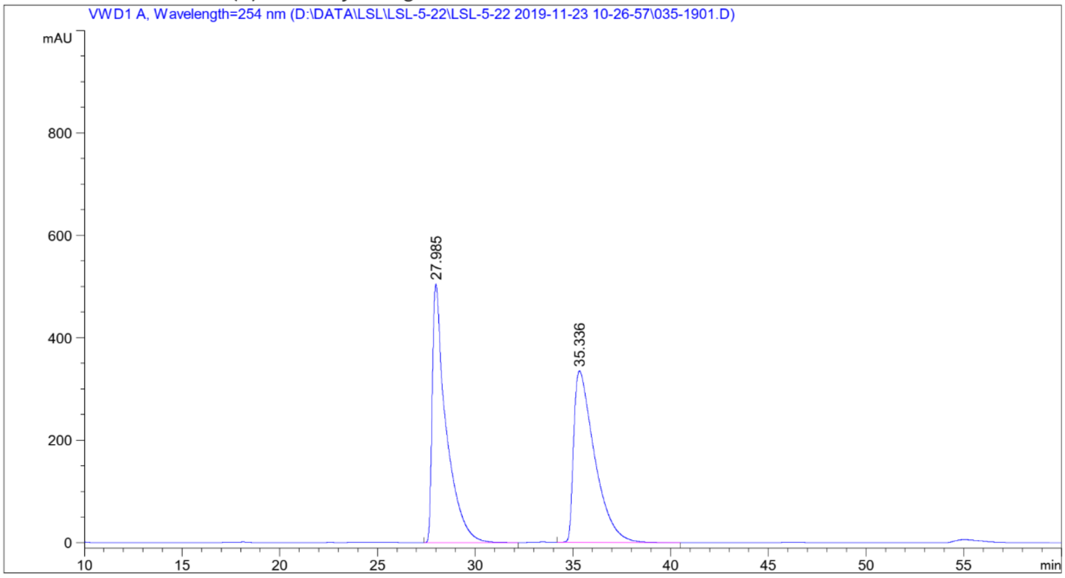

===

Area Percent Report

===

Sorted By : Signal
Multiplier : 1.0000
Dilution : 1.0000
Use Multiplier & Dilution Factor with ISTDs

Signal 1: VWD1 A, Wavelength=254 nm

Peak #	RetTime [min]	Type	Width [min]	Area [mAU*s]	Height [mAU]	Area %
1	27.985	BB	0.6954	2.48468e4	504.66043	50.0156
2	35.336	BB	1.1013	2.48313e4	334.40659	49.9844

Totals : 4.96782e4 839.06702

Data File D:\DATA\LSL\LSL-5-22\LSL-5-22 2019-11-23 10-26-57\045-2001.D
Sample Name: lsl-ph

===

Acq. Operator : Seq. Line : 20
Acq. Instrument : Instrument 1 Location : Vial 45
Injection Date : 11/24/2019 2:41:29 AM Inj : 1
Inj Volume : 5.000 µl
Acq. Method : D:\DATA\LSL\LSL-5-22\LSL-5-22 2019-11-23 10-26-57\VWD-OD(1-2)-95-5-1ML-5UL-254NM-60MIN.M
Last changed : 11/23/2019 10:42:24 PM
Analysis Method : D:\METHOD\LWD\VWD-OD(1-2)-85-15-0.15ML-3UL-210NM-80MIN.M
Last changed : 11/24/2019 9:36:39 PM
(modified after loading)

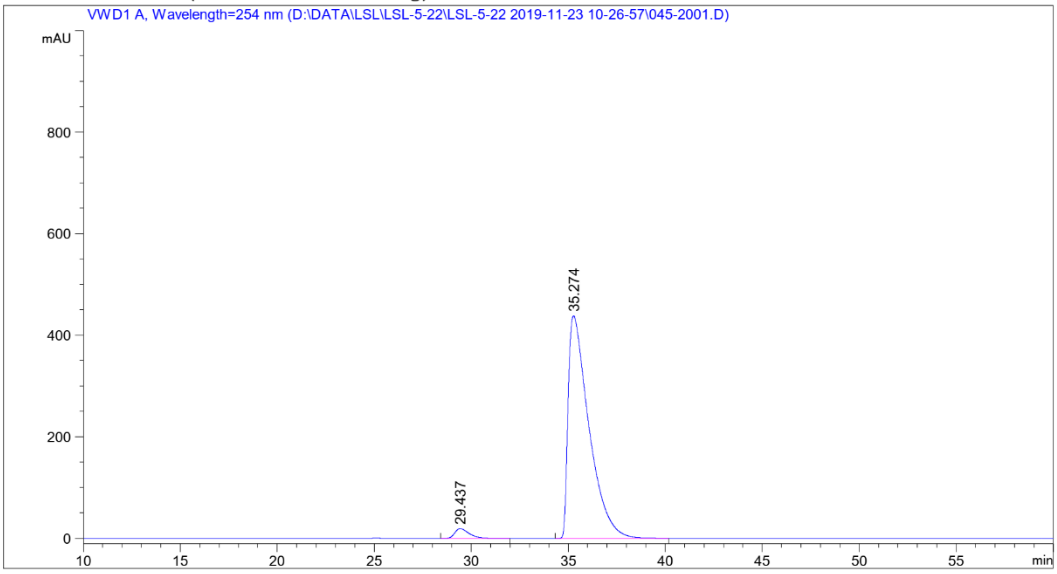

===

Area Percent Report

===

Sorted By : Signal
Multiplier : 1.0000
Dilution : 1.0000
Use Multiplier & Dilution Factor with ISTDs

Signal 1: VWD1 A, Wavelength=254 nm

Peak #	RetTime [min]	Type	Width [min]	Area [mAU*s]	Height [mAU]	Area %
1	29.437	BB	0.7824	990.90814	19.22360	2.9487
2	35.274	BB	1.1241	3.26135e4	438.30768	97.0513

Totals : 3.36044e4 457.53128

===

Instrument 1 11/24/2019 9:36:46 PM Page 1 of 1

Supplementary Figure 79. HPLC spectra for compound 3m

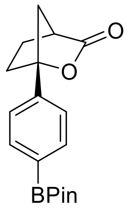

3n

Data File D:\DATA\GUAN YUQING\LK-A\LK-4-F-TS-EE 2020-07-02 16-15-03\083-0701.D
Sample Name: LSL-P-BPIN

===
Acq. Operator   :   Seq. Line :   7
Acq. Instrument : Instrument 1   Location : Vial 83
Injection Date : 7/2/2020 7:51:28 PM   Inj :   1
                                                Inj Volume : 5.000 µl
Acq. Method   : D:\DATA\GUAN YUQING\LK-A\LK-4-F-TS-EE 2020-07-02 16-15-03\VWD-OD(1-6)-99-1-0.5ML-5UL-220NM-90MIN.M
Last changed   : 7/2/2020 9:09:12 PM
                  (modified after loading)
Analysis Method : D:\METHOD\GUAN YUQING\VWD-OD(1-6)-96-4-0.5ML-5UL-210NM-10MIN.M
Last changed   : 7/2/2020 10:17:33 PM
                  (modified after loading)
Additional Info : Peak(s) manually integrated

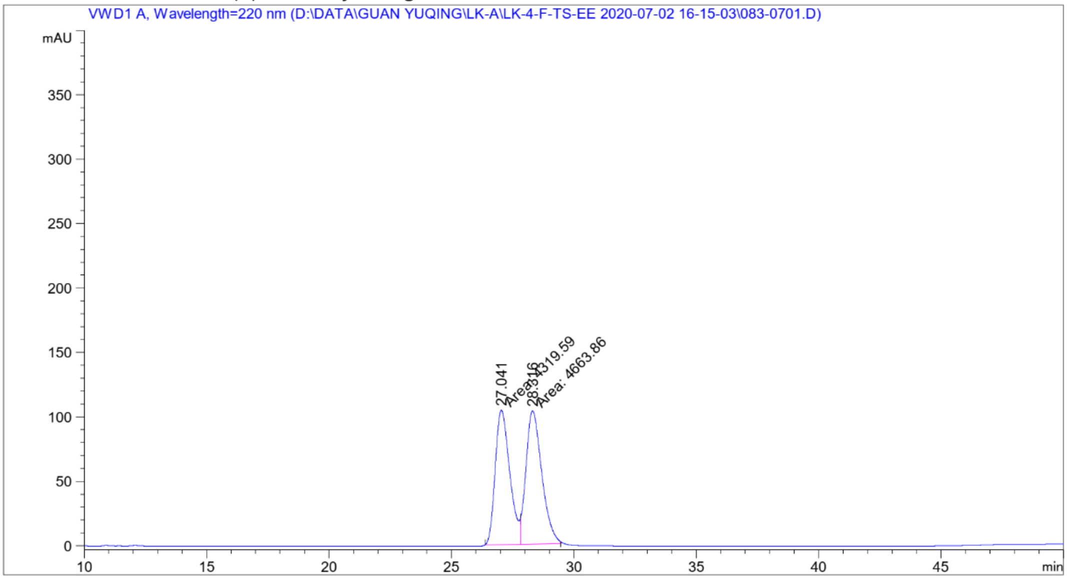

===
Area Percent Report
===

Sorted By   :   Signal
Multiplier   :   1.0000
Dilution   :   1.0000
Use Multiplier & Dilution Factor with ISTDs

Signal 1: VWD1 A, Wavelength=220 nm

Peak #	RetTime [min]	Type	Width [min]	Area [mAU*s]	Height [mAU]	Area %
1	27.041	MF	0.6890	4319.59326	104.49349	48.0839
2	28.316	FM	0.7514	4663.86426	103.44584	51.9161
Totals :				8983.45752	207.93933	

Instrument 1 7/2/2020 10:17:36 PM   Page   1 of 2

Data File D:\DATA\GUAN YUQING\LK-A\LK-4-F-TS-EE 2020-07-02 16-15-03\084-0801.D
Sample Name: LSL-P-BIN-C

===

Acq. Operator : Seq. Line : 8
Acq. Instrument : Instrument 1 Location : Vial 84
Injection Date : 7/2/2020 9:10:05 PM Inj : 1
Inj Volume : 5.000 µl
Acq. Method : D:\DATA\GUAN YUQING\LK-A\LK-4-F-TS-EE 2020-07-02 16-15-03\VWD-OD(1-6)-99-1-0.5ML-5UL-220NM-90MIN.M
Last changed : 7/2/2020 9:09:12 PM
(modified after loading)
Analysis Method : D:\METHOD\GUAN YUQING\VWD-OD(1-6)-96-4-0.5ML-5UL-210NM-10MIN.M
Last changed : 7/2/2020 10:19:20 PM
(modified after loading)
Additional Info : Peak(s) manually integrated

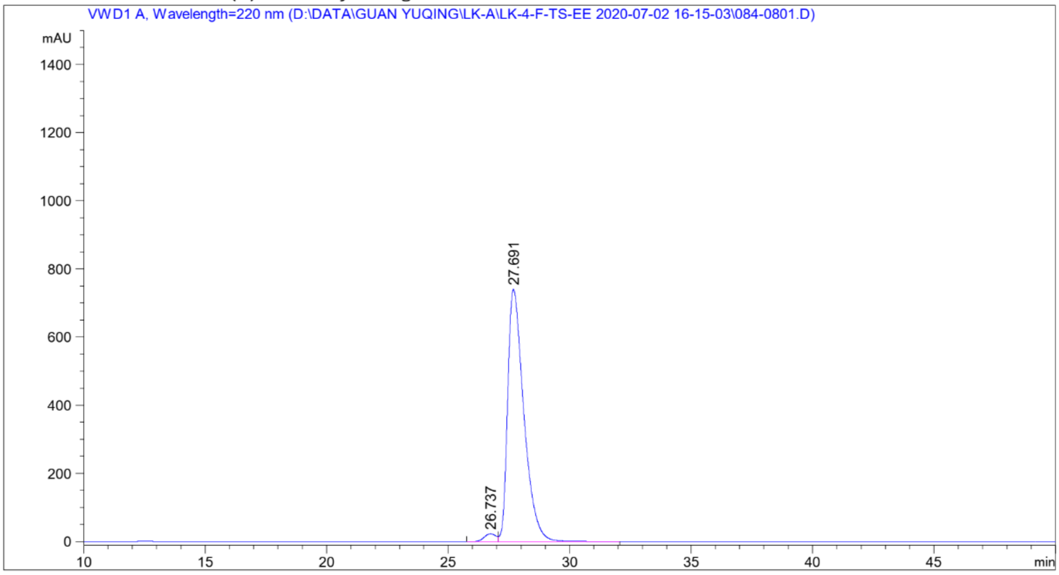

===

Area Percent Report

===

Sorted By : Signal
Multiplier : 1.0000
Dilution : 1.0000
Use Multiplier & Dilution Factor with ISTDs

Signal 1: VWD1 A, Wavelength=220 nm

Peak #	RetTime [min]	Type	Width [min]	Area [mAU*s]	Height [mAU]	Area %
1	26.737	BV	0.5153	754.36218	22.78495	2.1597
2	27.691	VB	0.6965	3.41753e4	740.39209	97.8403

Totals : 3.49297e4 763.17704

Supplementary Figure 80. HPLC spectra for compound 3n

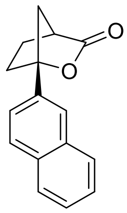

3o

Data File D:\DATA\LSL\LSL-5-17\LSL-5-17-2 2019-12-02 16-26-58\051-1001.D
Sample Name: LSL-NAI

===

Acq. Operator : Seq. Line : 10
Acq. Instrument : Instrument 1 Location : Vial 51
Injection Date : 12/2/2019 9:56:45 PM Inj : 1
Inj Volume : 3.000 µl
Acq. Method : D:\DATA\LSL\LSL-5-17\LSL-5-17-2 2019-12-02 16-26-58\VWD-AD(1-2)-95-5-0.5ML-3UL-220NM-60MIN.M
Last changed : 7/23/2018 8:16:17 AM
Analysis Method : D:\METHOD\LSL\VWD-AD(1-2)-99-1-1ML-5UL-210NM-60MIN.M
Last changed : 12/3/2019 12:36:39 PM
(modified after loading)
Additional Info : Peak(s) manually integrated

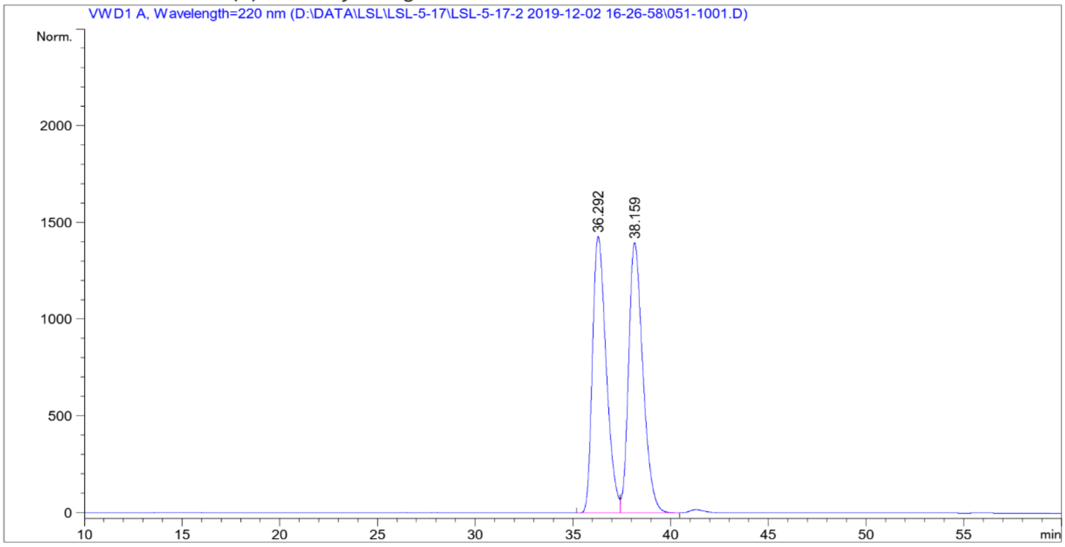

===
Area Percent Report
===

Sorted By : Signal
Multiplier : 1.0000
Dilution : 1.0000
Use Multiplier & Dilution Factor with ISTDs

Signal 1: VWD1 A, Wavelength=220 nm

Peak #	RetTime [min]	Type	Width [min]	Area [mAU*s]	Height [mAU]	Area %
1	36.292	BV	0.7483	6.98919e4	1429.88916	49.5978
2	38.159	VB	0.7731	7.10255e4	1397.04480	50.4022

Totals : 1.40917e5 2826.93396

Data File D:\DATA\LSL\LSL-5-17-3\LSL-5-17-NAI 2019-12-03 10-21-01\052-0401.D
Sample Name: LSL-2-NAI

===

Acq. Operator : Seq. Line : 4
Acq. Instrument : Instrument 1 Location : Vial 52
Injection Date : 12/3/2019 11:30:37 AM Inj : 1
Inj Volume : 3.000 µl
Acq. Method : D:\DATA\LSL\LSL-5-17-3\LSL-5-17-NAI 2019-12-03 10-21-01\VWD-AD(1-2)-95-5-0.5ML-3UL-220NM-60MIN.M
Last changed : 12/3/2019 11:30:46 AM
(modified after loading)
Analysis Method : D:\METHOD\LSL\VWD-AD(1-2)-99-1-1ML-5UL-210NM-60MIN.M
Last changed : 12/3/2019 12:33:51 PM
(modified after loading)
Additional Info : Peak(s) manually integrated

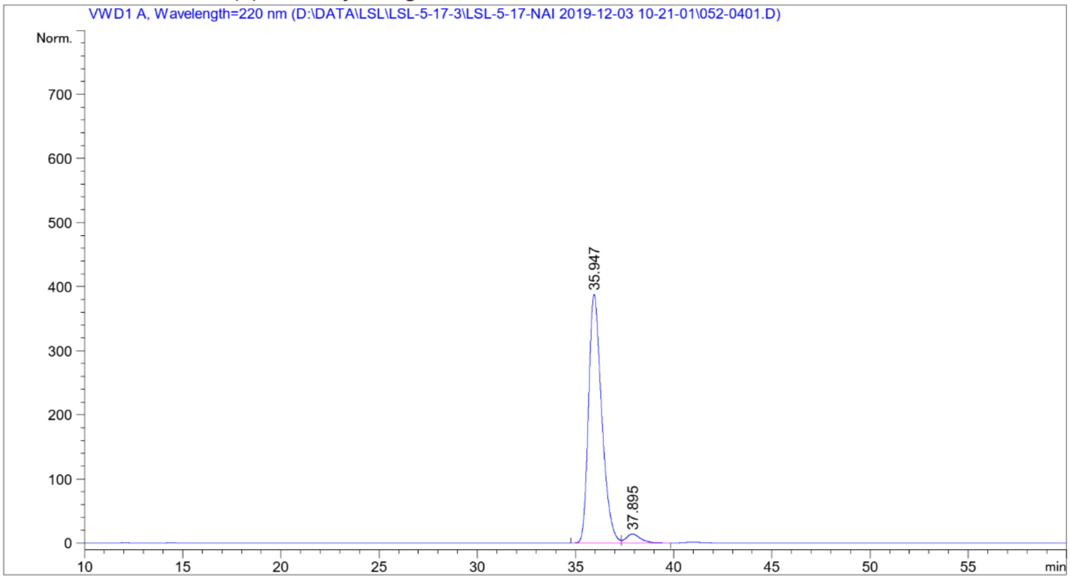

===

Area Percent Report

===

Sorted By : Signal
Multiplier : 1.0000
Dilution : 1.0000
Use Multiplier & Dilution Factor with ISTDs

Signal 1: VWD1 A, Wavelength=220 nm

Peak #	RetTime [min]	Type	Width [min]	Area [mAU*s]	Height [mAU]	Area %
1	35.947	BV	0.7070	1.80730e4	387.59146	96.2816
2	37.895	VB	0.7451	697.98010	13.94120	3.7184

Totals : 1.87710e4 401.53266

Instrument 1 12/3/2019 12:33:56 PM Page 1 of 2

Supplementary Figure 81. HPLC spectra for compound 3o

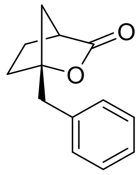

3p

Data File D:\DATA\LSL\LSL-5-22\LSL-5-22 2019-11-23 10-26-57\036-1601.D
Sample Name: LSL-Bn-rac

===

Acq. Operator : Seq. Line : 16
Acq. Instrument : Instrument 1 Location : Vial 36
Injection Date : 11/23/2019 11:27:50 PM Inj : 1
Inj Volume : 5.000 µl
Acq. Method : D:\DATA\LSL\LSL-5-22\LSL-5-22 2019-11-23 10-26-57\VWD-OD(1-2)-97-3-1ML-5UL-210NM-60MIN.M
Last changed : 11/23/2019 10:08:19 AM
Analysis Method : D:\METHOD\LWD\VWD-OD(1-2)-85-15-0.15ML-3UL-210NM-80MIN.M
Last changed : 11/24/2019 9:37:55 PM
(modified after loading)
Additional Info : Peak(s) manually integrated

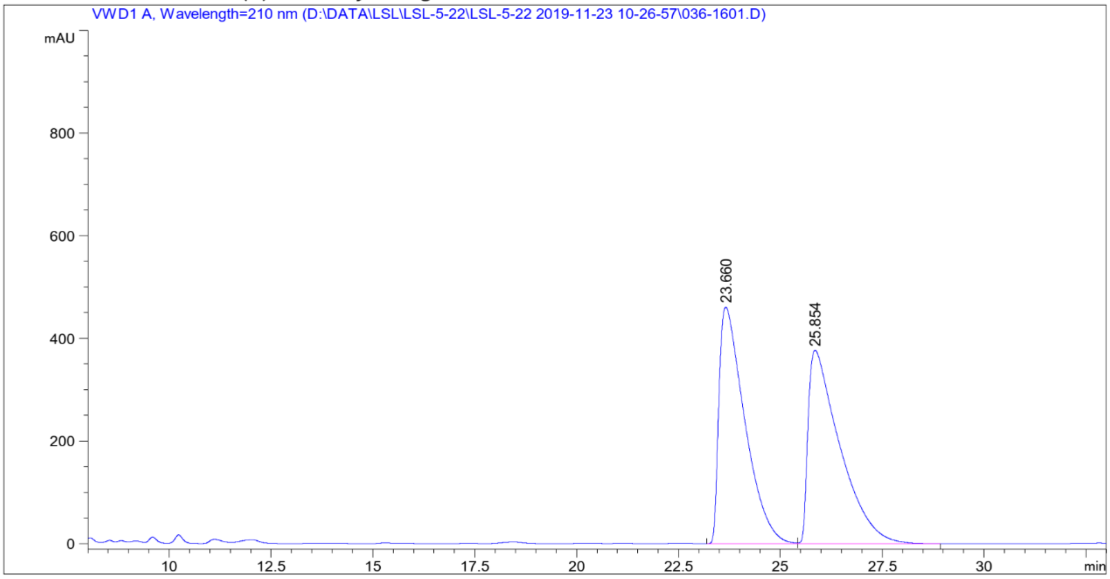

===

Area Percent Report

===

Sorted By : Signal
Multiplier : 1.0000
Dilution : 1.0000
Use Multiplier & Dilution Factor with ISTDs

Signal 1: VWD1 A, Wavelength=210 nm

Peak #	RetTime [min]	Type	Width [min]	Area [mAU*s]	Height [mAU]	Area %
1	23.660	BV	0.6605	2.02645e4	461.26483	49.8596
2	25.854	VB	0.7752	2.03786e4	376.66876	50.1404

Totals : 4.06431e4 837.93359

Data File D:\DATA\LSL\LSL-5-22\LSL-5-22 2019-11-23 10-26-57\046-1701.D
Sample Name: LSL-Bn-c

===

Acq. Operator : Seq. Line : 17
Acq. Instrument : Instrument 1 Location : Vial 46
Injection Date : 11/24/2019 12:28:43 AM Inj : 1
Inj Volume : 5.000 µl
Acq. Method : D:\DATA\LSL\LSL-5-22\LSL-5-22 2019-11-23 10-26-57\VWD-OD(1-2)-97-3-1ML-5UL-210NM-60MIN.M
Last changed : 11/23/2019 10:08:19 AM
Analysis Method : D:\METHOD\LWD\VWD-OD(1-2)-85-15-0.15ML-3UL-210NM-80MIN.M
Last changed : 11/24/2019 9:39:58 PM
(modified after loading)
Additional Info : Peak(s) manually integrated

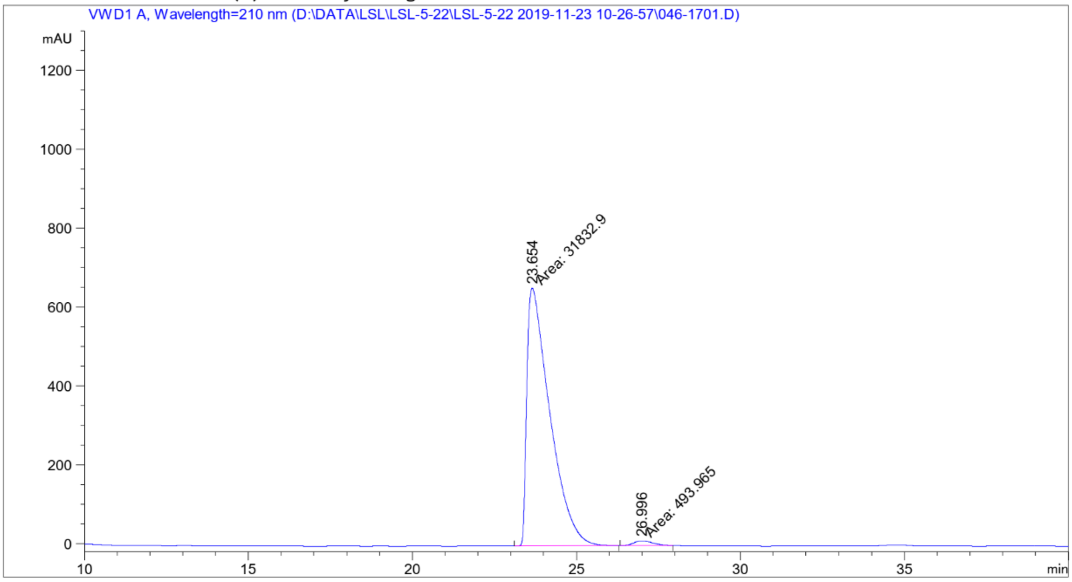

===

Area Percent Report

===

Sorted By : Signal
Multiplier : 1.0000
Dilution : 1.0000
Use Multiplier & Dilution Factor with ISTDs

Signal 1: VWD1 A, Wavelength=210 nm

Peak #	RetTime [min]	Type	Width [min]	Area [mAU*s]	Height [mAU]	Area %
1	23.654	MM	0.8117	3.18329e4	653.62006	98.4720
2	26.996	MM	0.6791	493.96524	12.12325	1.5280

Totals : 3.23268e4 665.74331

Supplementary Figure 82. HPLC spectra for compound 3p

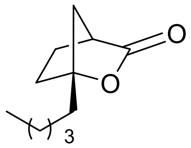

3q

2019-12-11 19:32:46 Page 1 / 1

SHIMADZU LabSolutions

Analysis Report

<Sample Information>

Sample Name : lsl-hex-rac
Sample ID :
Data Filename : lsl-hex-rac.gcd
Method Filename : bdex225-220-130~160-230-75min.gcm
Batch Filename : lsl-hex.gcb
Vial # : 1 Sample Type : Unknown
Injection Volume : 1 uL
Date Acquired : 2019-12-11 17:11:24 Acquired by : System Administrator
Date Processed : 2019-12-11 18:26:29 Processed by : System Administrator

<Chromatogram>

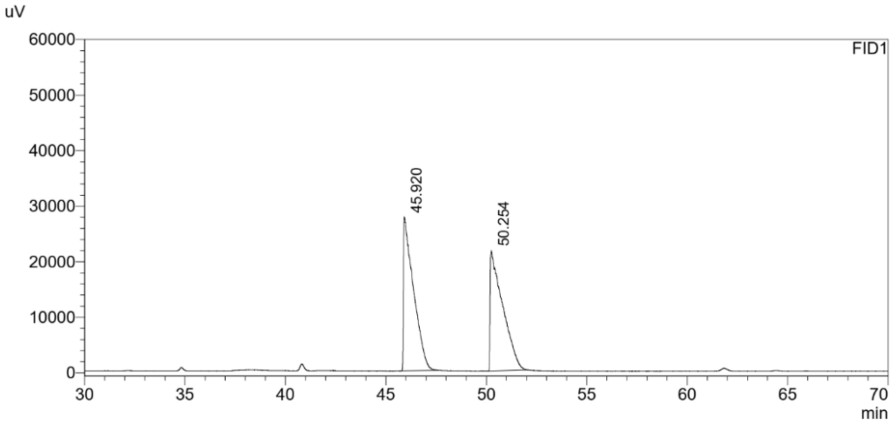

<Peak Table>

FID1

Peak#	Ret. Time	Area	Height	Conc.	Unit	Mark	Name
1	45.920	961823	27648	50.504		M	
2	50.254	942635	21679	49.496		M	
Total		1904457	49327				

D:\DATA FILE\lsl\data\lsl-hex\lsl-hex-rac.gcd

SHIMADZU LabSolutions **Analysis Report**

<Sample Information>

Sample Name	: lsl-hex-c		
Sample ID	:		
Data Filename	: lsl-hex-c.gcd		
Method Filename	: bdex225-220-130~160-230-75min.gcm		
Batch Filename	: lsl-hex.gcb		
Vial #	: 2	Sample Type	: Unknown
Injection Volume	: 1 uL		
Date Acquired	: 2019-12-11 18:31:21	Acquired by	: System Administrator
Date Processed	: 2019-12-11 19:46:24	Processed by	: System Administrator

<Chromatogram>

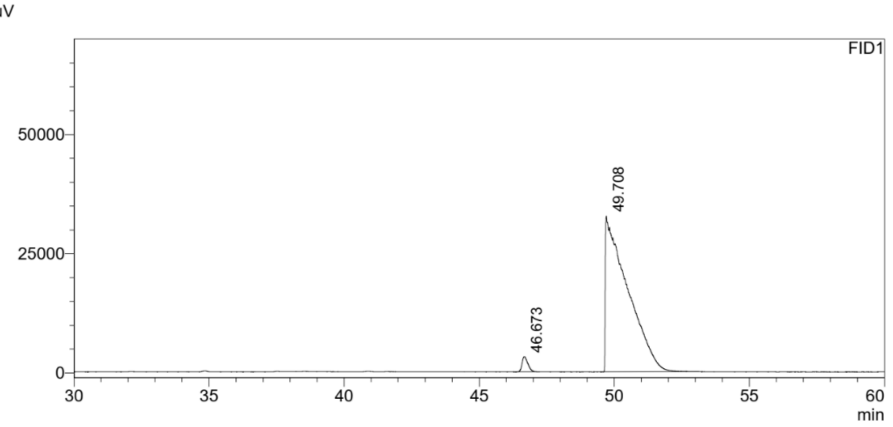

<Peak Table>

FID1

Peak#	Ret. Time	Area	Height	Conc.	Unit	Mark	Name
1	46.673	47912	3194	2.495		M	
2	49.708	1872043	32644	97.505		M	
Total		1919955	35838				

D:\DATA FILE\lsl\data\lsl-hex\lsl-hex-c.gcd

Supplementary Figure 83. GC spectra for compound 3q

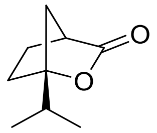

3r

2019-12-23 19:57:22 Page 1 / 1

SHIMADZU LabSolutions Analysis Report

<Sample Information>

Sample Name	: lsl-5-yibing-rac		
Sample ID	:		
Data Filename	: lsl-5-yibing-rac.gcd		
Method Filename	: bdex225-220-130~160-230-75min.gcm		
Batch Filename	: lsl-5-yibing,huanbing,huanwu.gcb		
Vial #	: 1	Sample Type	: Unknown
Injection Volume	: 1 uL		
Date Acquired	: 2019-12-23 16:59:42	Acquired by	: System Administrator
Date Processed	: 2019-12-23 18:14:48	Processed by	: System Administrator

<Chromatogram>

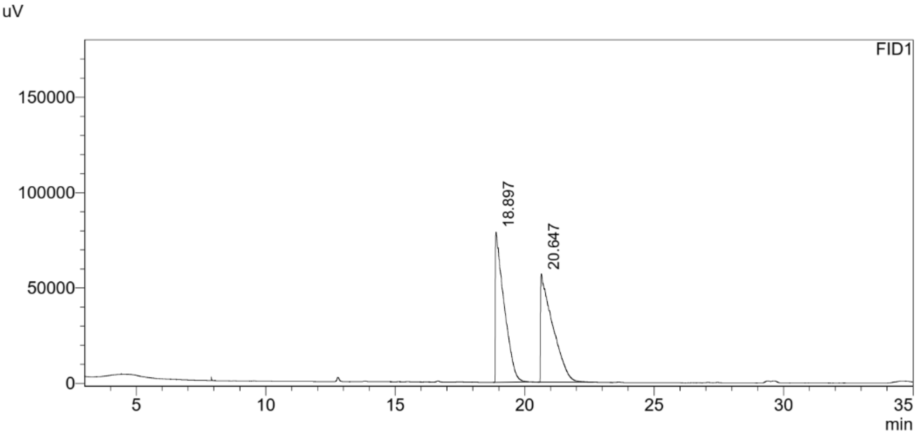

<Peak Table>

FID1

Peak#	Ret. Time	Area	Height	Conc.	Unit	Mark	Name
1	18.897	1845650	78748	50.196		M	
2	20.647	1831213	56663	49.804		M	
Total		3676863	135411				

D:\DATA FILE\lsl\data\lsl-yibingji, huangbingji,huanwuji\lsl-5-yibing-rac.gcd

SHIMADZU LabSolutions **Analysis Report**

<Sample Information>

Sample Name	: lsl-5-yibing-c		
Sample ID	:		
Data Filename	: lsl-5-yibing-c.gcd		
Method Filename	: bdex225-220-130~160-230-75min.gcm		
Batch Filename	: lsl-5-yibing,huanbing,huanwu.gcb		
Vial #	: 2	Sample Type	: Unknown
Injection Volume	: 1 uL		
Date Acquired	: 2019-12-23 18:19:42	Acquired by	: System Administrator
Date Processed	: 2019-12-23 19:34:47	Processed by	: System Administrator

<Chromatogram>

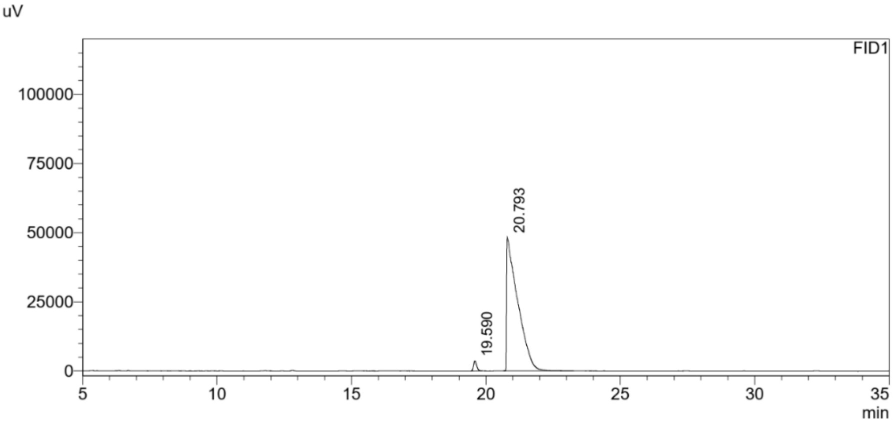

<Peak Table>

FID1

Peak#	Ret. Time	Area	Height	Conc.	Unit	Mark	Name
1	19.590	30730	3710	2.163		V	
2	20.793	1389921	48394	97.837		M	
Total		1420651	52104				

D:\DATA FILE\lsl\data\lsl-yibingji, huangbingji,huanwuji\lsl-5-yibing-c.gcd

Supplementary Figure 84. GC spectra for compound 3r

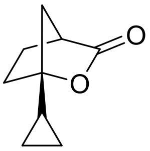

3s

SHIMADZU LabSolutions

Analysis Report

<Sample Information>

Sample Name	: lsl-5-huanbing-rac		
Sample ID	:		
Data Filename	: lsl-5-huanbing-rac.gcd		
Method Filename	: bdex225-220-130~160-230-75min.gcm		
Batch Filename	: lsl-5-yibing,huanbing,huanwu.gcb		
Vial #	: 3	Sample Type	: Unknown
Injection Volume	: 1 uL		
Date Acquired	: 2019-12-23 19:39:42	Acquired by	: System Administrator
Date Processed	: 2019-12-23 20:54:46	Processed by	: System Administrator

<Chromatogram>

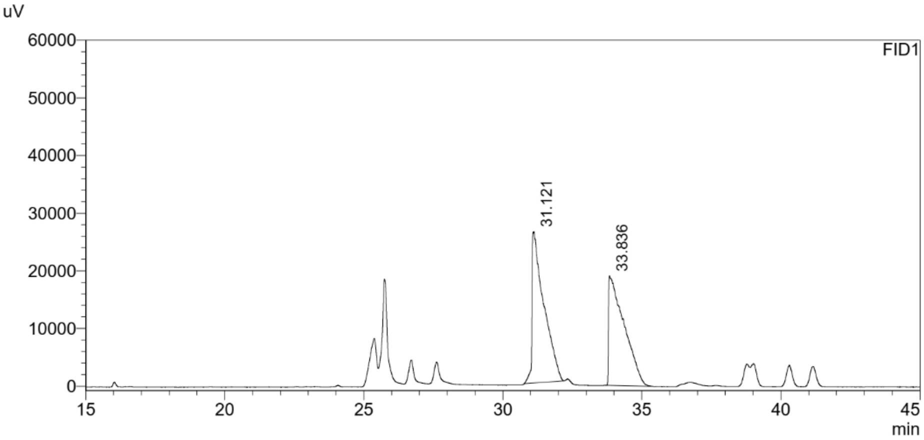

<Peak Table>

FID1

Peak#	Ret. Time	Area	Height	Conc.	Unit	Mark	Name
1	31.121	786153	26299	51.427		M	
2	33.836	742520	19018	48.573		M	
Total		1528673	45317				

D:\DATA FILE\lsl\data\lsl-yibingji, huangbingji,huanwuji\lsl-5-huanbing-rac.gcd

SHIMADZU LabSolutions **Analysis Report**

<Sample Information>

Sample Name	: lsl-5-huanbing-c		
Sample ID	:		
Data Filename	: lsl-5-huanbing-c.gcd		
Method Filename	: bdex225-220-130~160-230-75min.gcm		
Batch Filename	: lsl-5-yibing,huanbing,huanwu.gcb		
Vial #	: 4	Sample Type	: Unknown
Injection Volume	: 1 uL		
Date Acquired	: 2019-12-23 20:59:42	Acquired by	: System Administrator
Date Processed	: 2019-12-23 22:14:46	Processed by	: System Administrator

<Chromatogram>

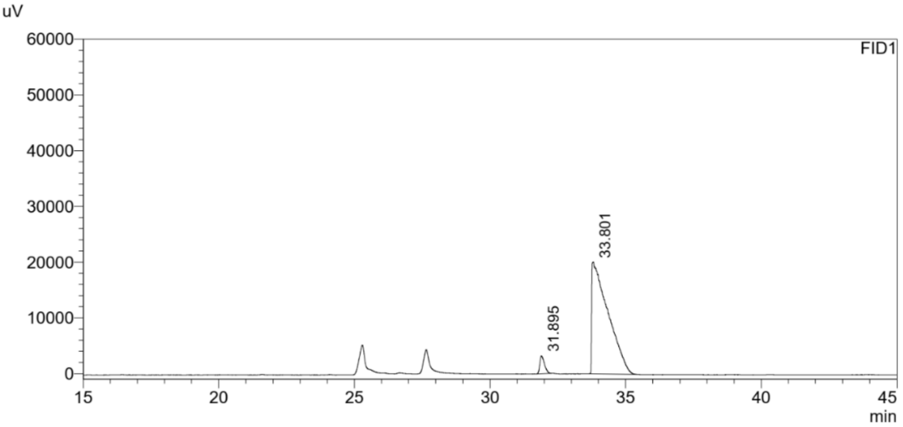

<Peak Table>

FID1

Peak#	Ret. Time	Area	Height	Conc.	Unit	Mark	Name
1	31.895	37836	3214	4.299		M	
2	33.801	842285	20047	95.701		M	
Total		880120	23261				

D:\DATA FILE\lsl\data\lsl-yibingji, huangbingji,huanwuji\lsl-5-huanbing-c.gcd

Supplementary Figure 85. GC spectra for compound 3s

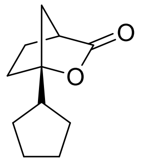

3t

SHIMADZU LabSolutions

Analysis Report

<Sample Information>

Sample Name	: lsl-5-huanwu-rac		
Sample ID	:		
Data Filename	: lsl-5-huanwu-rac.gcd		
Method Filename	: bdex225-220-130~160-230-75min.gcm		
Batch Filename	: lsl-5-huanwu-2.gcb		
Vial #	: 1	Sample Type	: Unknown
Injection Volume	: 1 uL		
Date Acquired	: 2019-12-26 15:15:30	Acquired by	: System Administrator
Date Processed	: 2019-12-26 16:30:32	Processed by	: System Administrator

<Chromatogram>

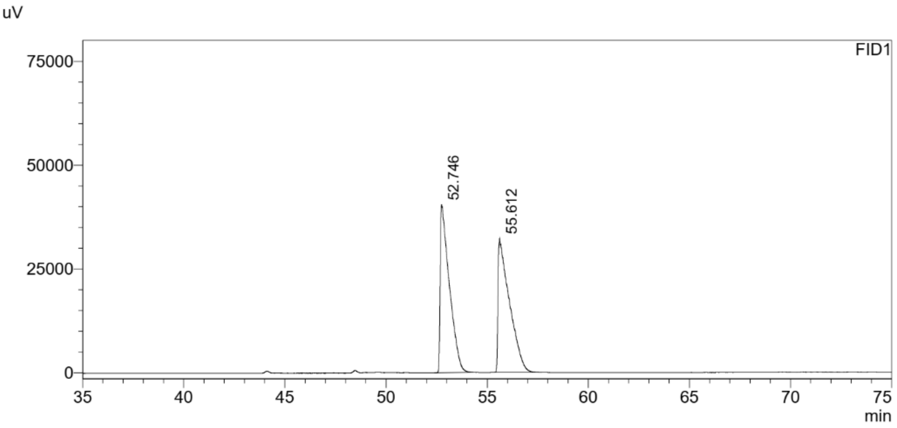

<Peak Table>

FID1

Peak#	Ret. Time	Area	Height	Conc.	Unit	Mark	Name
1	52.746	1274370	40415	50.127		M	
2	55.612	1267914	32305	49.873		M	
Total		2542284	72720				

D:\DATA FILE\lsl\data\lsl-yibingji, huangbingji,huanwuji\lsl-5-huanwu-rac.gcd

SHIMADZU LabSolutions **Analysis Report**

<Sample Information>

Sample Name	: lsl-5-huanwu-c		
Sample ID	:		
Data Filename	: lsl-5-huanwu-c-2.gcd		
Method Filename	: bdex225-220-130~160-230-75min.gcm		
Batch Filename	: lsl-5-huanwu-2.gcb		
Vial #	: 2	Sample Type	: Unknown
Injection Volume	: 1 uL		
Date Acquired	: 2019-12-26 16:35:27	Acquired by	: System Administrator
Date Processed	: 2019-12-26 17:50:31	Processed by	: System Administrator

<Chromatogram>

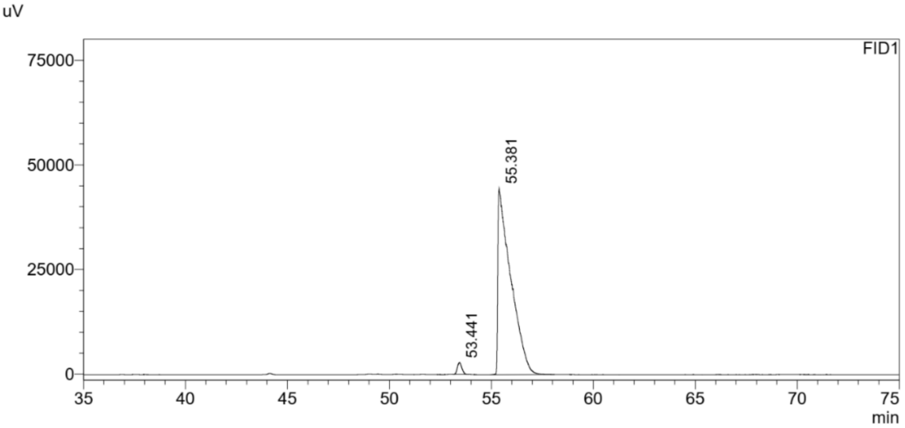

<Peak Table>

FID1

Peak#	Ret. Time	Area	Height	Conc.	Unit	Mark	Name
1	53.441	43285	2841	2.153		M	
2	55.381	1966961	44582	97.847		M	
Total		2010247	47423				

D:\DATA FILE\lsl\data\lsl-yibingji, huangbingji,huanwuji\lsl-5-huanwu-c-2.gcd

Supplementary Figure 86. GC spectra for compound 3t

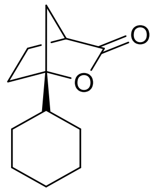

3u

2019-12-7 15:21:47 Page 1 / 1

Analysis Report

SHIMADZU LabSolutions

<Sample Information>

Sample Name : lsl-huanji-rac
Sample ID :
Data Filename : lsl-huanji-rac.gcd
Method Filename : bdex225-220-130~160-230-90min.gcm
Batch Filename : lsl-huanji-c.gcb
Vial # : 1 Sample Type : Unknown
Injection Volume : 1 uL
Date Acquired : 2019-12-7 10:42:02 Acquired by : System Administrator
Date Processed : 2019-12-7 12:12:07 Processed by : System Administrator

<Chromatogram>

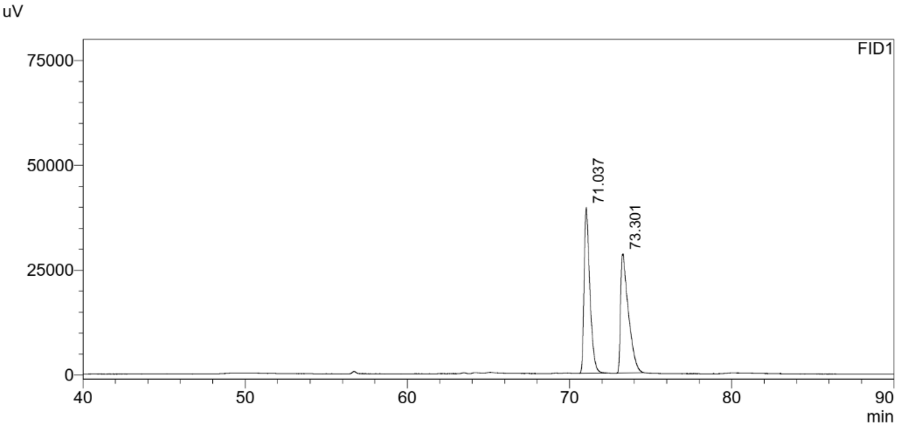

<Peak Table>

FID1

Peak#	Ret. Time	Area	Height	Conc.	Unit	Mark	Name
1	71.037	979042	39513	50.601			
2	73.301	955802	28443	49.399		M	
Total		1934843	67956				

D:\DATA FILE\lsl\data\lsl-huanji-rac.gcd

SHIMADZU LabSolutions

Analysis Report

<Sample Information>

Sample Name	: lsl-huanji-c		
Sample ID	:		
Data Filename	: lsl-huanji-c.gcd		
Method Filename	: bdex225-220-130~160-230-90min.gcm		
Batch Filename	: lsl-huanji-c.gcb		
Vial #	: 2	Sample Type	: Unknown
Injection Volume	: 1 uL		
Date Acquired	: 2019-12-7 12:17:01	Acquired by	: System Administrator
Date Processed	: 2019-12-7 13:47:05	Processed by	: System Administrator

<Chromatogram>

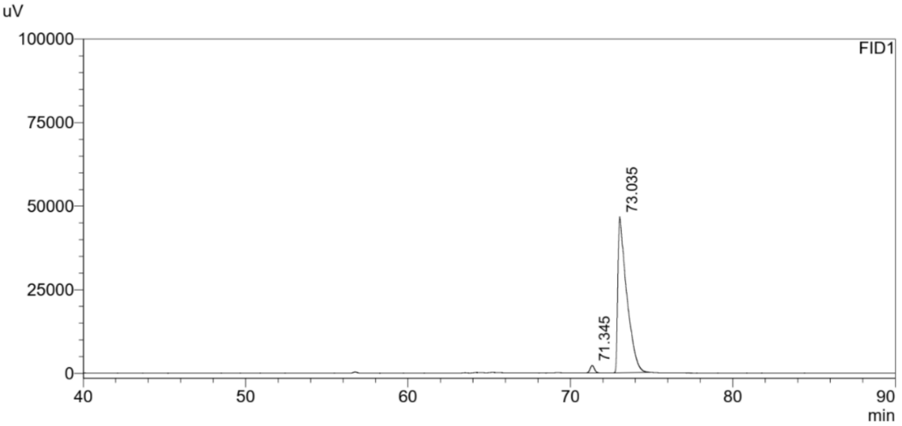

<Peak Table>

FID1

Peak#	Ret. Time	Area	Height	Conc.	Unit	Mark	Name
1	71.345	39481	2217	2.170			
2	73.035	1780071	46704	97.830			
Total		1819552	48921				

Supplementary Figure 87. GC spectra for compound 3u

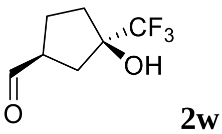

2w

SHIMADZU LabSolutions

Analysis Report

<Sample Information>

Sample Name	: lsl-5-76-r		
Sample ID	:		
Data Filename	: lsl-5-76-r.gcd		
Method Filename	: bdex225-220-120~140-230-55min.gcm		
Batch Filename	: lsl-5-76-r.gcb		
Vial #	: 4	Sample Type	: Unknown
Injection Volume	: 1 uL		
Date Acquired	: 2020-6-20 10:04:49	Acquired by	: System Administrator
Date Processed	: 2020-6-20 11:19:53	Processed by	: System Administrator

<Chromatogram>

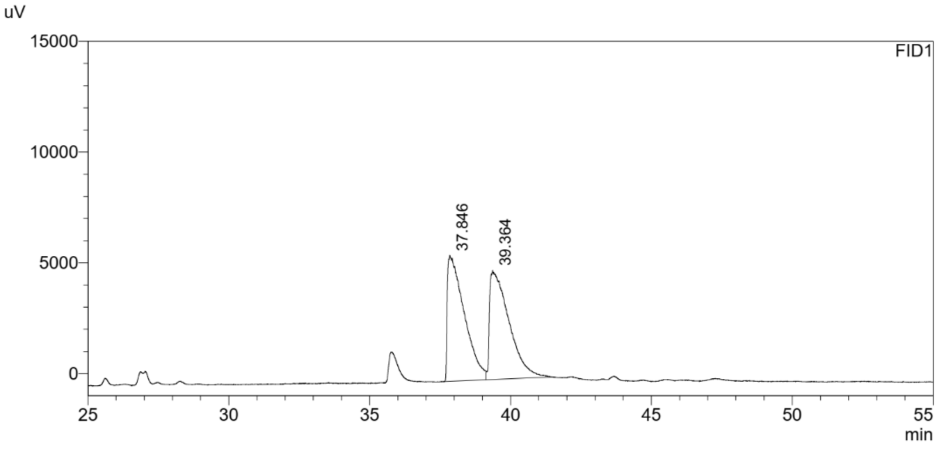

<Peak Table>

FID1

Peak#	Ret. Time	Area	Height	Conc.	Unit	Mark	Name
1	37.846	236917	5675	50.198		M	
2	39.364	235047	4916	49.802		V M	
Total		471964	10591				

D:\DATA FILE\lsl\data\lsl-5-66\lsl-5-76-r.gcd

SHIMADZU LabSolutions **Analysis Report**

<Sample Information>

Sample Name	: lsl-5-76-c-2		
Sample ID	:		
Data Filename	: lsl-5-76-c-2.gcd		
Method Filename	: bdex225-220-120~140-230-55min.gcm		
Batch Filename	: lsl-5-76-c-2.gcb		
Vial #	: 5	Sample Type	: Unknown
Injection Volume	: 1 uL		
Date Acquired	: 2020-6-19 19:55:13	Acquired by	: System Administrator
Date Processed	: 2020-6-19 21:10:17	Processed by	: System Administrator

<Chromatogram>

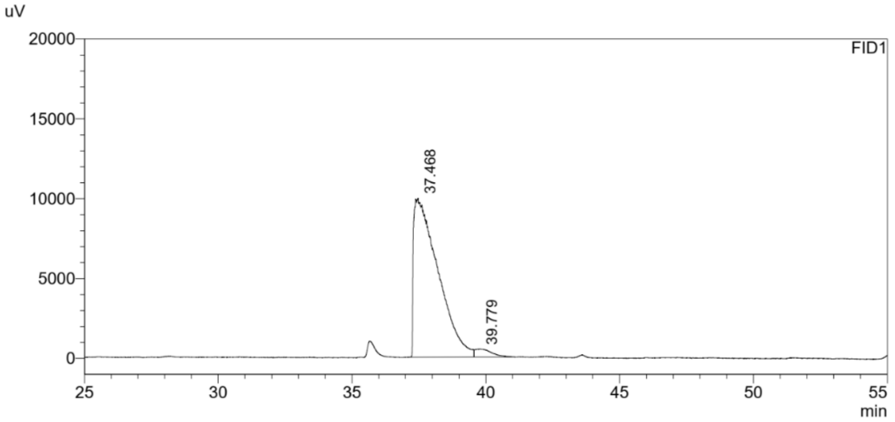

<Peak Table>

FID1

Peak#	Ret. Time	Area	Height	Conc.	Unit	Mark	Name
1	37.468	630454	9962	96.578		M	
2	39.779	22341	510	3.422		V M	
Total		652795	10472				

D:\DATA FILE\lsl\data\lsl-5-66\lsl-5-76-c-2.gcd

Supplementary Figure 88. GC spectra for compound 2w

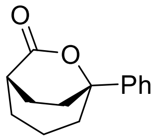

3x

Data File D:\DATA\LSL\LSL-5-81\LSL-5-81-2-C 2020-07-04 11-24-27\082-0301.D
Sample Name: LSL-qi-rac

===

Acq. Operator : Seq. Line : 3
Acq. Instrument : Instrument 1 Location : Vial 82
Injection Date : 7/4/2020 11:50:44 AM Inj : 1
Inj Volume : 5.000 µl
Acq. Method : D:\DATA\LSL\LSL-5-81\LSL-5-81-2-C 2020-07-04 11-24-27\VWD-AD(1-2)-97-3-1ML-5UL-210NM-60MIN.M
Last changed : 9/29/2019 6:56:04 PM
Analysis Method : D:\METHOD\GUAN YUQING\VWD-OD(1-6)-97-3-0.5ML-5UL-210NM-160MIN.M
Last changed : 7/4/2020 2:14:22 PM
(modified after loading)
Additional Info : Peak(s) manually integrated

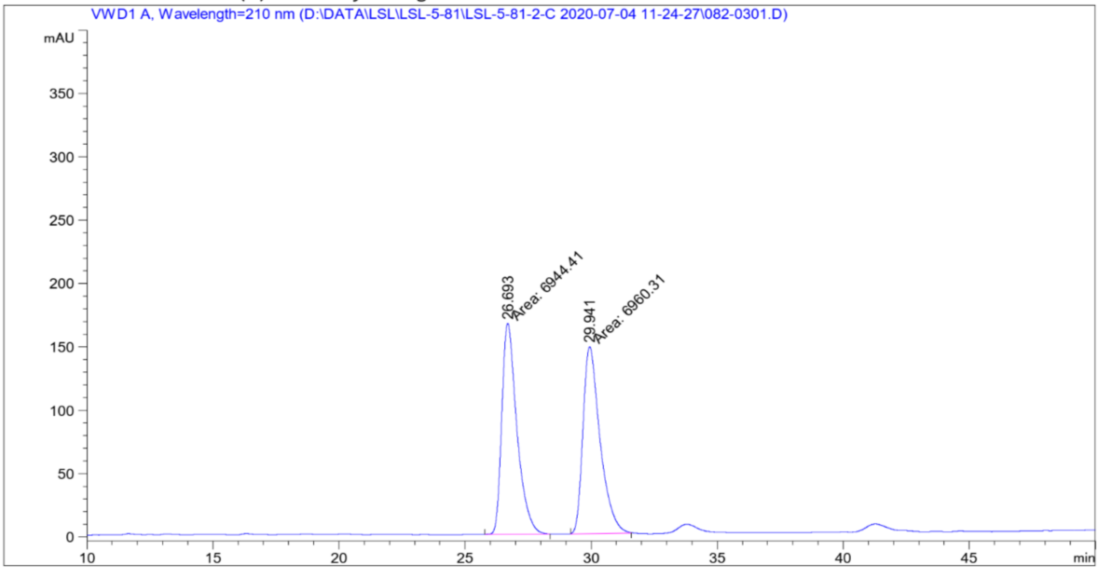

===

Area Percent Report

===

Sorted By : Signal
Multiplier : 1.0000
Dilution : 1.0000
Use Multiplier & Dilution Factor with ISTDs

Signal 1: VWD1 A, Wavelength=210 nm

Peak #	RetTime [min]	Type	Width [min]	Area [mAU*s]	Height [mAU]	Area %
1	26.693	MM	0.6948	6944.40967	166.57359	49.9428
2	29.941	MM	0.7860	6960.31445	147.59740	50.0572

Totals : 1.39047e4 314.17099

Data File D:\DATA\LSL\LSL-5-81\LSL-5-81-2-C 2020-07-04 11-24-27\083-0401.D
Sample Name: LSL-qi-c

===

Acq. Operator : Seq. Line : 4
Acq. Instrument : Instrument 1 Location : Vial 83
Injection Date : 7/4/2020 12:51:36 PM Inj : 1
Inj Volume : 5.000 µl
Acq. Method : D:\DATA\LSL\LSL-5-81\LSL-5-81-2-C 2020-07-04 11-24-27\VWD-AD(1-2)-97-3-1ML-5UL-210NM-60MIN.M
Last changed : 9/29/2019 6:56:04 PM
Analysis Method : D:\METHOD\GUAN YUQING\VWD-OD(1-6)-97-3-0.5ML-5UL-210NM-160MIN.M
Last changed : 7/4/2020 2:15:52 PM
(modified after loading)
Additional Info : Peak(s) manually integrated

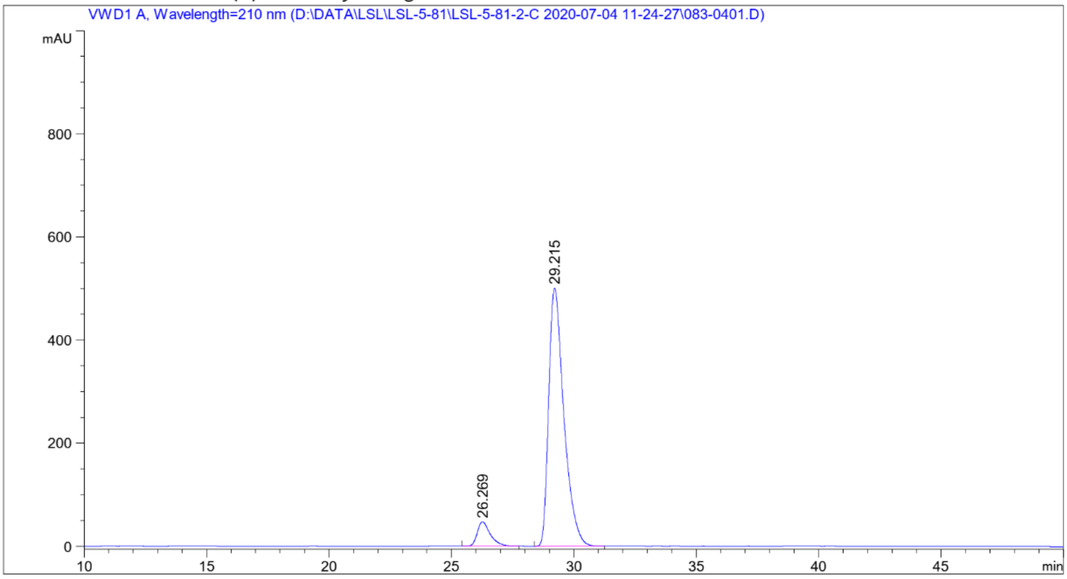

===

Area Percent Report

===

Sorted By : Signal
Multiplier : 1.0000
Dilution : 1.0000
Use Multiplier & Dilution Factor with ISTDs

Signal 1: VWD1 A, Wavelength=210 nm

Peak #	RetTime [min]	Type	Width [min]	Area [mAU*s]	Height [mAU]	Area %
1	26.269	BB	0.5472	1732.44653	47.31734	7.4513
2	29.215	BB	0.6488	2.15179e4	500.26712	92.5487
Totals :				2.32504e4	547.58446	

Instrument 1 7/4/2020 2:15:56 PM Page 1 of 2

Supplementary Figure 89. HPLC spectra for compound 3x

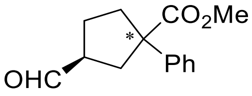

5a

```
Data File D:\DATA\LSL\LSL-135\LSL-5-135-AD 2020-08-27 17-53-28\003-1001.D
Sample Name: LSL-OMe-As-r

    =====================================================================
    Acq. Operator   :                                 Seq. Line :  10
    Acq. Instrument : Instrument 1                     Location : Vial 3
    Injection Date  : 8/27/2020 9:15:16 PM                  Inj :   1
                                                     Inj Volume : 3.000 µl
    Acq. Method     : D:\DATA\LSL\LSL-135\LSL-5-135-AD 2020-08-27 17-53-28\VWD-AS(1-6)-99-1-1ML-
                      3UL-220NM-60MIN.M
    Last changed    : 5/29/2018 8:52:56 AM
    Analysis Method : D:\DATA\GUAN YUQING\LJ-2-161\LJ-2-161 2020-08-26 14-15-29\VWD-AD(1-2)-80-20
                      --1ML-5UL-254NM-20MIN.M
    Last changed    : 8/29/2020 9:12:58 PM
                      (modified after loading)
    Additional Info : Peak(s) manually integrated
```

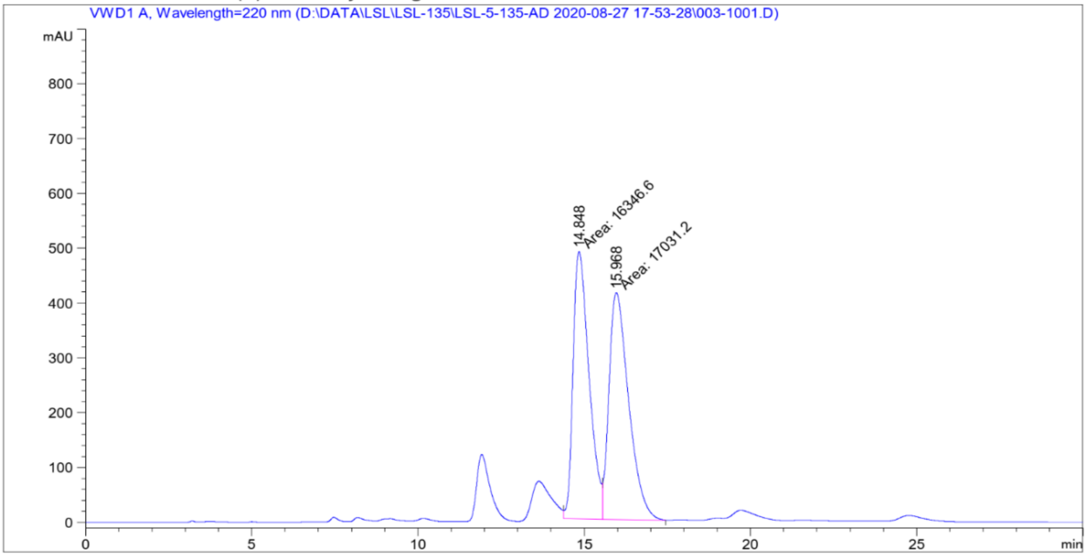


```
    =====================================================================
                            Area Percent Report
    =====================================================================

    Sorted By             :      Signal
    Multiplier            :      1.0000
    Dilution              :      1.0000
    Use Multiplier & Dilution Factor with ISTDs


    Signal 1: VWD1 A, Wavelength=220 nm

    Peak RetTime Type  Width     Area      Height     Area
      #   [min]        [min]   [mAU*s]     [mAU]        %
    ----|-------|----|-------|----------|----------|--------|
       1  14.848 MF     0.5588 1.63466e4  487.55164  48.9744
       2  15.968 FM     0.6857 1.70312e4  413.97235  51.0256

    Totals :                   3.33778e4  901.52399
```

Data File D:\DATA\LSL\LSL-135\LSL-135-2 2020-08-29 17-27-32\002-0301.D
Sample Name: LSL-OME-C

===

Acq. Operator : Seq. Line : 3
Acq. Instrument : Instrument 1 Location : Vial 2
Injection Date : 8/29/2020 5:53:19 PM Inj : 1
Inj Volume : 3.000 µl
Acq. Method : D:\DATA\LSL\LSL-135\LSL-135-2 2020-08-29 17-27-32\VWD-AS(1-6)-99-1-1ML-3UL-220NM-60MIN.M
Last changed : 5/29/2018 8:52:56 AM
Analysis Method : D:\DATA\LSL\LSL-135\LSL-135-2 2020-08-29 17-27-32\002-0301.D\DA.M (VWD-AS(1-6)-99-1-1ML-3UL-220NM-60MIN.M, From Data File)
Last changed : 8/29/2020 9:09:57 PM
(modified after loading)
Additional Info : Peak(s) manually integrated

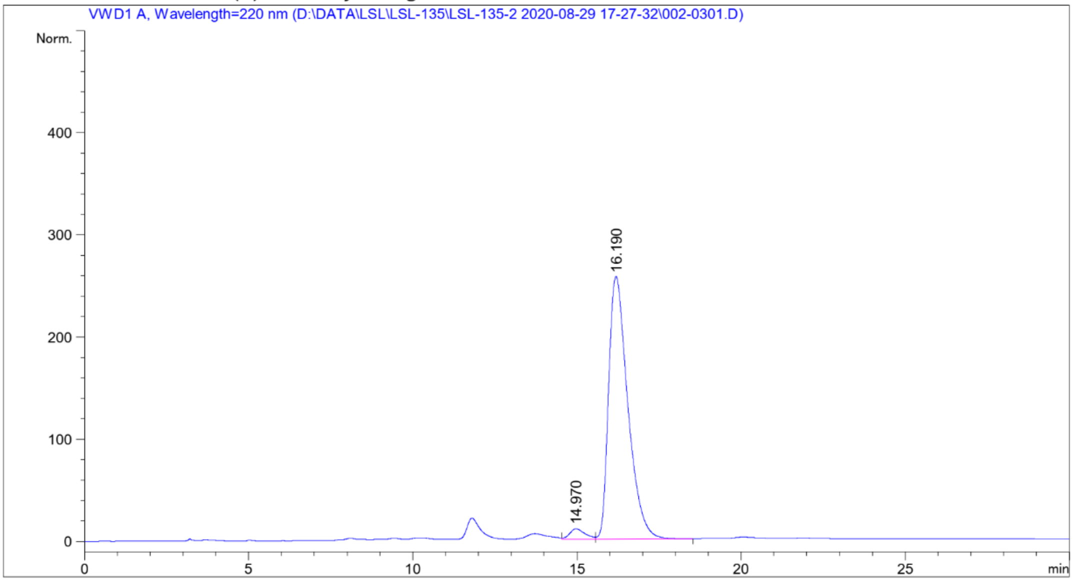

===
Area Percent Report
===

Sorted By : Signal
Multiplier : 1.0000
Dilution : 1.0000
Use Multiplier & Dilution Factor with ISTDs

Signal 1: VWD1 A, Wavelength=220 nm

Peak #	RetTime [min]	Type	Width [min]	Area [mAU*s]	Height [mAU]	Area %
1	14.970	VV	0.4743	327.92484	10.19381	3.0448
2	16.190	VB	0.6168	1.04421e4	257.10480	96.9552

Totals : 1.07700e4 267.29860

Instrument 1 8/29/2020 9:10:02 PM Page 1 of 2

Supplementary Figure 90. HPLC spectra for compound 5a

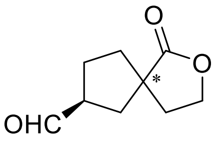

5b

```
Data File D:\DATA\LSL\LSL-5-LUO\LSL-5-31-4 2019-12-13 16-33-54\041-0301.D
Sample Name: LSL-5-31-4

    =====================================================================
    Acq. Operator   :                                  Seq. Line :    3
    Acq. Instrument : Instrument 2                      Location : Vial 41
    Injection Date  : 12/13/2019 4:57:04 PM                  Inj :    1
                                                      Inj Volume : 5.000 µl
    Acq. Method     : D:\DATA\LSL\LSL-5-LUO\LSL-5-31-4 2019-12-13 16-33-54\DAD-OJ(1-6)-85-15-1ML-
                      5UL-ALL-50MIN.M
    Last changed    : 12/6/2019 7:34:13 PM
    Analysis Method : D:\METHOD\LWD\DAD-IC(1-6)-95-5-0.5ML-2UL-ALL-10MIN.M
    Last changed    : 12/14/2019 9:39:07 AM
                      (modified after loading)
    Additional Info : Peak(s) manually integrated
```

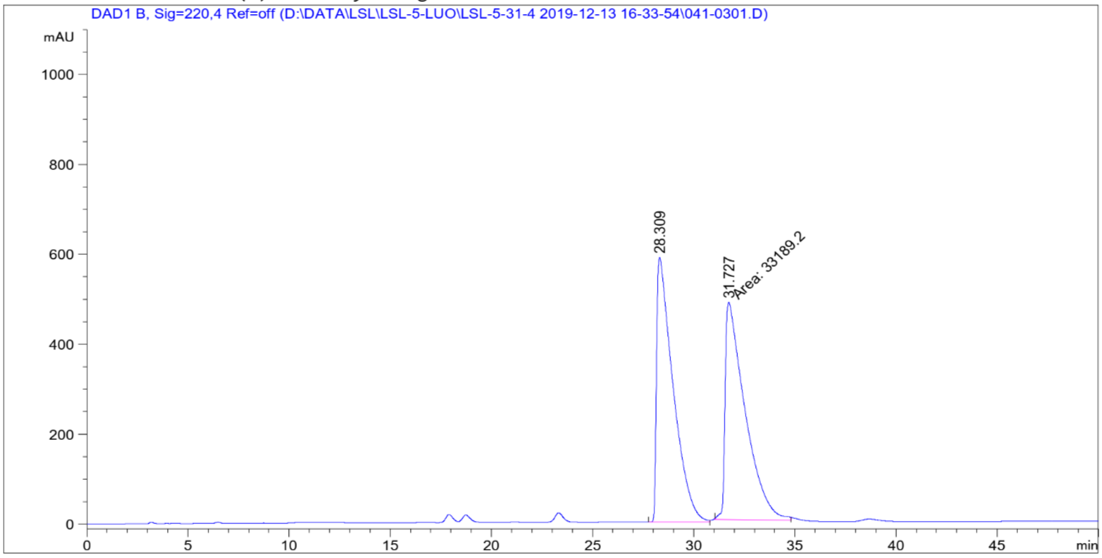


```
    =====================================================================
                             Area Percent Report
    =====================================================================

    Sorted By             :      Signal
    Multiplier            :      1.0000
    Dilution              :      1.0000
    Use Multiplier & Dilution Factor with ISTDs


    Signal 1: DAD1 B, Sig=220,4 Ref=off

    Peak RetTime Type  Width     Area      Height     Area
      #   [min]        [min]   [mAU*s]     [mAU]        %
    ----|-------|----|-------|----------|----------|--------|
       1  28.309 BV    0.7853 3.33528e4  588.40472 50.1229
       2  31.727 MM    1.1444 3.31892e4  483.36938 49.8771

    Totals :                  6.65420e4  1071.77411
```

Data File D:\DATA\LSL\LSL-5-LUO\LSL-5-31-4 2019-12-13 16-33-54\042-0401.D
Sample Name: LSL-5-31-4-C

```
=====================================================================
Acq. Operator   :                               Seq. Line :   4
Acq. Instrument : Instrument 2                   Location : Vial 42
Injection Date  : 12/13/2019 5:48:03 PM               Inj :   1
                                               Inj Volume : 5.000 µl
Acq. Method     : D:\DATA\LSL\LSL-5-LUO\LSL-5-31-4 2019-12-13 16-33-54\DAD-OJ(1-6)-85-15-1ML-
                  5UL-ALL-50MIN.M
Last changed    : 12/6/2019 7:34:13 PM
Analysis Method : D:\METHOD\LWD\DAD-IC(1-6)-95-5-0.5ML-2UL-ALL-10MIN.M
Last changed    : 12/14/2019 9:29:48 AM
                  (modified after loading)
Additional Info : Peak(s) manually integrated
```

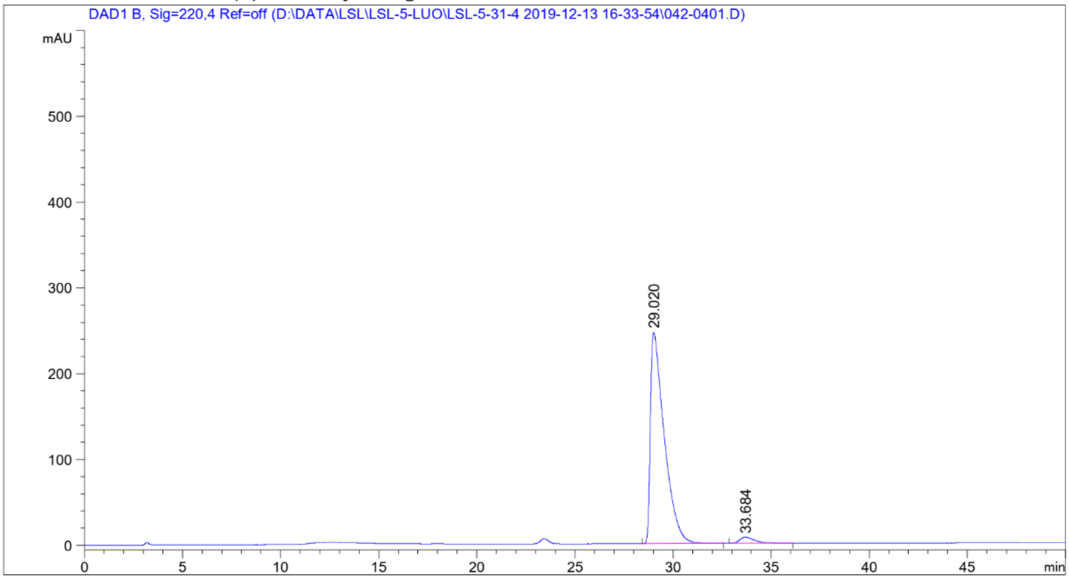


```
=====================================================================
                         Area Percent Report
=====================================================================

Sorted By             :      Signal
Multiplier            :      1.0000
Dilution              :      1.0000
Use Multiplier & Dilution Factor with ISTDs


Signal 1: DAD1 B, Sig=220,4 Ref=off
```

Peak #	RetTime [min]	Type	Width [min]	Area [mAU*s]	Height [mAU]	Area %
1	29.020	BB	0.7098	1.21007e4	246.05632	96.8737
2	33.684	BB	0.7351	390.50784	7.15152	3.1263

Totals : 1.24912e4 253.20784

Supplementary Figure 91. HPLC spectra for compound 5b

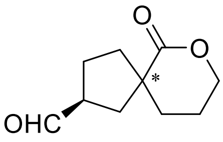

5c

Data File D:\DATA\LSL\LSL-5-131\LSL-5-131 2020-08-19 11-09-46\012-0501.D
Sample Name: LSL-LUO-6

===

Acq. Operator : Seq. Line : 5
Acq. Instrument : Instrument 1 Location : Vial 12
Injection Date : 8/19/2020 12:47:47 PM Inj : 1
Inj Volume : 5.000 µl
Acq. Method : D:\DATA\LSL\LSL-5-131\LSL-5-131 2020-08-19 11-09-46\VWD-AD(1-2)-85-15-1ML-5UL-220NM-40MIN.M
Last changed : 1/3/2020 2:20:54 PM
Analysis Method : D:\METHOD\LYH\VWD-AD(1-2)-95-5-1ML-10UL-210NM-60MIN.M
Last changed : 8/19/2020 2:21:53 PM
(modified after loading)
Additional Info : Peak(s) manually integrated

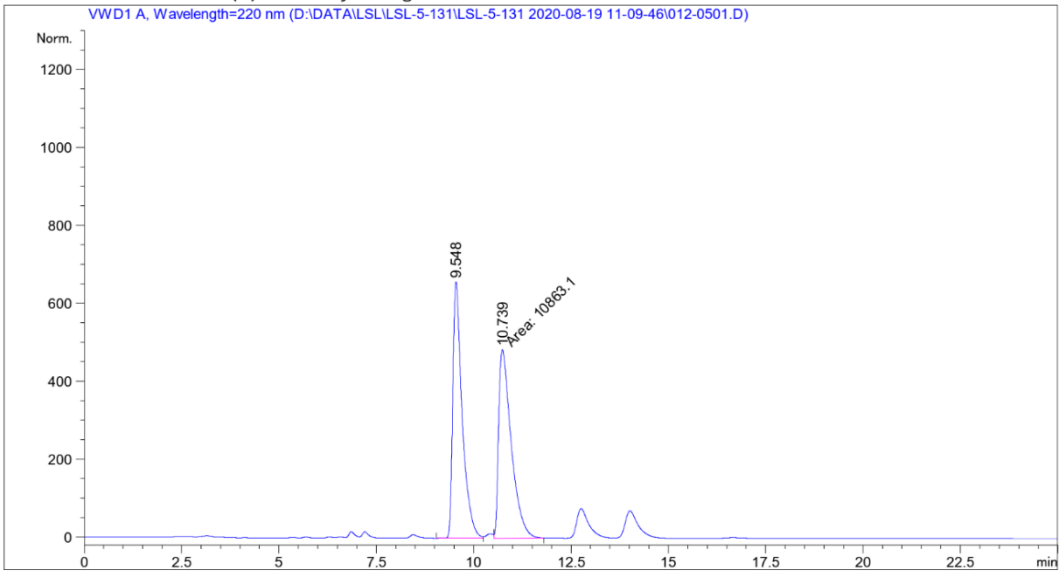

===

Area Percent Report

===

Sorted By : Signal
Multiplier : 1.0000
Dilution : 1.0000
Use Multiplier & Dilution Factor with ISTDs

Signal 1: VWD1 A, Wavelength=220 nm

Peak #	RetTime [min]	Type	Width [min]	Area [mAU*s]	Height [mAU]	Area %
1	9.548	BV	0.2468	1.10308e4	657.16449	50.3830
2	10.739	MM	0.3743	1.08631e4	483.67297	49.6170

Totals : 2.18939e4 1140.83746

```
Data File D:\DATA\LSL\LSL-5-131\LSL-5-131 2020-08-19 11-09-46\013-0601.D
Sample Name: LSL-LUO-6-C

    =====================================================================
    Acq. Operator   :                                 Seq. Line :   6
    Acq. Instrument : Instrument 1                     Location : Vial 13
    Injection Date  : 8/19/2020 1:28:41 PM                  Inj :   1
                                                     Inj Volume : 5.000 µl
    Acq. Method     : D:\DATA\LSL\LSL-5-131\LSL-5-131 2020-08-19 11-09-46\VWD-AD(1-2)-85-15-1ML-
                      5UL-220NM-40MIN.M
    Last changed    : 1/3/2020 2:20:54 PM
    Analysis Method : D:\METHOD\LYH\VWD-AD(1-2)-95-5-1ML-10UL-210NM-60MIN.M
    Last changed    : 8/19/2020 2:24:23 PM
                      (modified after loading)
    Additional Info : Peak(s) manually integrated
```

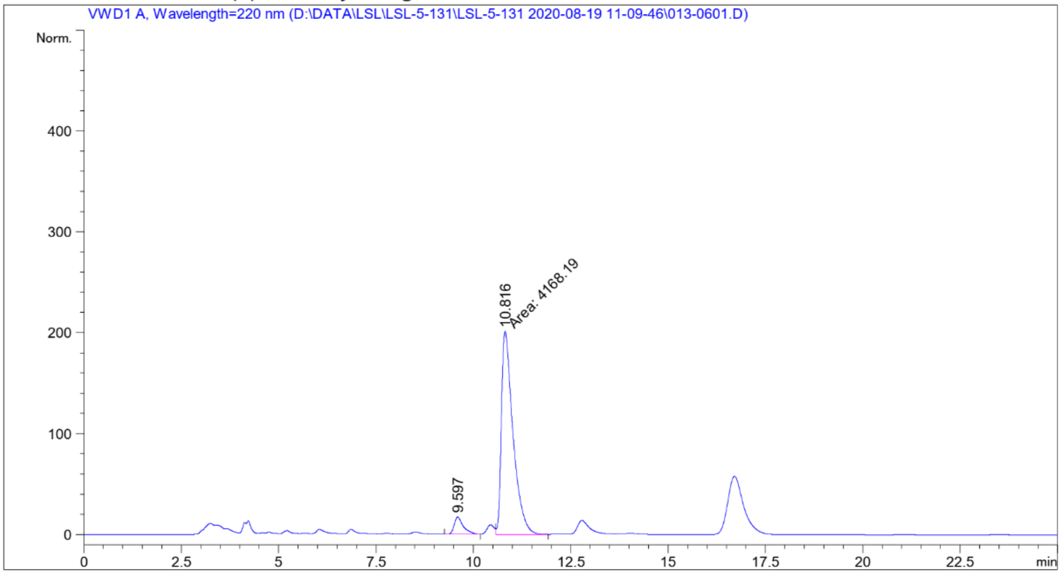


```
    =====================================================================
                             Area Percent Report
    =====================================================================

    Sorted By             :      Signal
    Multiplier            :      1.0000
    Dilution              :      1.0000
    Use Multiplier & Dilution Factor with ISTDs


    Signal 1: VWD1 A, Wavelength=220 nm

    Peak RetTime Type  Width     Area      Height     Area
      #   [min]        [min]   [mAU*s]     [mAU]        %
    ----|-------|----|-------|----------|----------|--------|
       1   9.597 BV    0.2407  280.59915   17.07792   6.3073
       2  10.816 FM    0.3452 4168.18994  201.27332  93.6927

    Totals :                  4448.78909  218.35124
```


Supplementary Figure 92. HPLC spectra for compound 5c

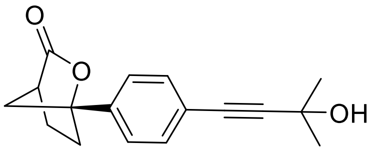

6

Data File D:\DATA\LSL\LSL-6-ALKYNE\LSL-6-ALKYNE-OD-3 2021-06-09 13-37-39\033-0301.D
Sample Name: LSL-AKYNE-RAC

===

Acq. Operator : Seq. Line : 3
Acq. Instrument : Instrument 2 Location : Vial 33
Injection Date : 6/9/2021 2:04:22 PM Inj : 1
Inj Volume : 5.000 µl
Acq. Method : D:\DATA\LSL\LSL-6-ALKYNE\LSL-6-ALKYNE-OD-3 2021-06-09 13-37-39\DAD-OD(1-2)-92-8-1ML-5UL-ALL-100MIN.M
Last changed : 6/9/2021 3:21:29 PM
(modified after loading)
Analysis Method : D:\METHOD\MYC\DAD-OD(1-2)-98-2-0.5ML-5UL-254-10MIN.M
Last changed : 6/9/2021 5:28:28 PM
(modified after loading)
Additional Info : Peak(s) manually integrated

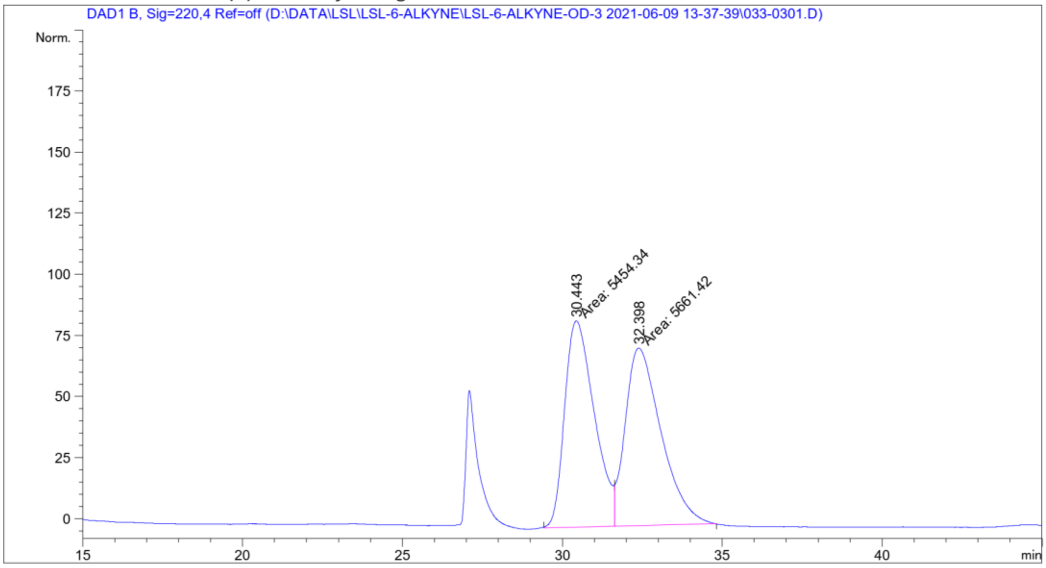

===

Area Percent Report

===

Sorted By : Signal
Multiplier : 1.0000
Dilution : 1.0000
Use Multiplier & Dilution Factor with ISTDs

Signal 1: DAD1 B, Sig=220,4 Ref=off

Peak #	RetTime [min]	Type	Width [min]	Area [mAU*s]	Height [mAU]	Area %
1	30.443	MF	1.0776	5454.34326	84.36189	49.0685
2	32.398	FM	1.2989	5661.42334	72.64340	50.9315

Totals : 1.11158e4 157.00529

Data File D:\DATA\LSL\LSL-6-ALKYNE\LSL-6-ALKYNE-OD-3 2021-06-09 13-37-39\034-0401.D
Sample Name: LSL-ALKYNE-C

===

Acq. Operator : Seq. Line : 4
Acq. Instrument : Instrument 2 Location : Vial 34
Injection Date : 6/9/2021 3:22:31 PM Inj : 1
Inj Volume : 5.000 µl
Acq. Method : D:\DATA\LSL\LSL-6-ALKYNE\LSL-6-ALKYNE-OD-3 2021-06-09 13-37-39\DAD-OD(1-2)-92-8-1ML-5UL-ALL-100MIN.M
Last changed : 6/9/2021 3:21:29 PM
(modified after loading)
Analysis Method : D:\METHOD\MYC\DAD-OD(1-2)-98-2-0.5ML-5UL-254-10MIN.M
Last changed : 6/9/2021 5:30:19 PM
(modified after loading)
Additional Info : Peak(s) manually integrated

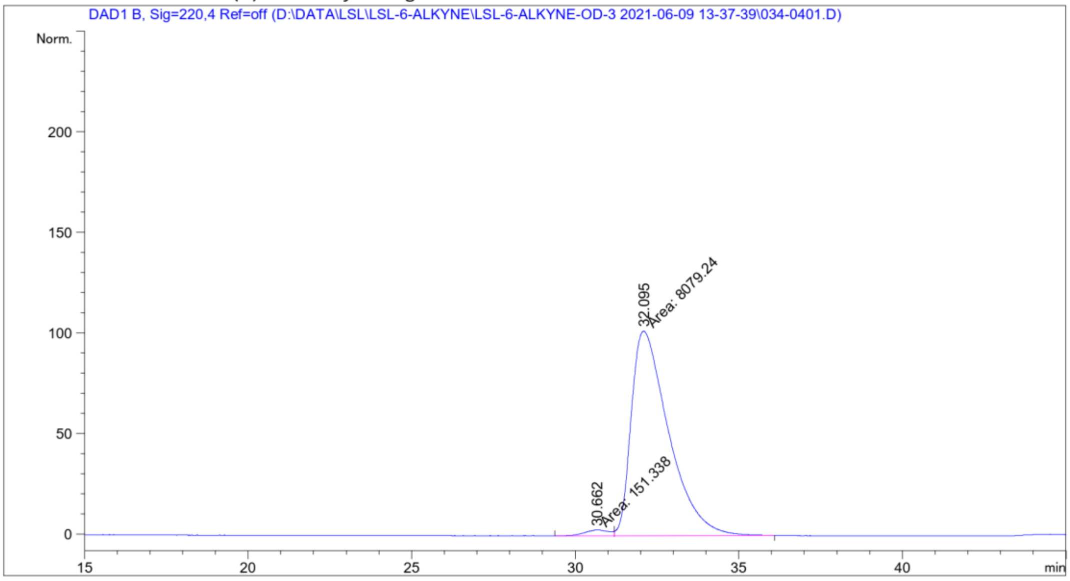

===

Area Percent Report

===

Sorted By : Signal
Multiplier : 1.0000
Dilution : 1.0000
Use Multiplier & Dilution Factor with ISTDs

Signal 1: DAD1 B, Sig=220,4 Ref=off

Peak #	RetTime [min]	Type	Width [min]	Area [mAU*s]	Height [mAU]	Area %
1	30.662	MF	0.8922	151.33838	2.82705	1.8387
2	32.095	FM	1.3257	8079.23975	101.56900	98.1613

Totals : 8230.57813 104.39605

Instrument 2 6/9/2021 5:30:22 PM Page 1 of 2

Supplementary Figure 93. HPLC spectra for compound 6

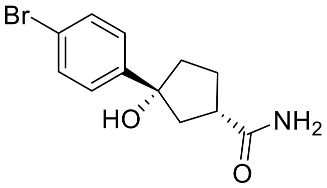

8

Data File D:\DATA\LGY\LGY-5-163\LGY-5-163 2020-08-06 21-49-10\012-2501.D
Sample Name: LSL-5-111-rac-2

===

Acq. Operator : Seq. Line : 25
Acq. Instrument : Instrument 2 Location : Vial 12
Injection Date : 8/7/2020 1:50:14 PM Inj : 1
Inj Volume : 5.000 µl
Acq. Method : D:\DATA\LGY\LGY-5-163\LGY-5-163 2020-08-06 21-49-10\DAD-OD(1-2)-80-20-1ML-5UL-ALL-90MIN.M
Last changed : 8/7/2020 2:23:18 PM
(modified after loading)
Analysis Method : D:\METHOD\LSL\DAD-OD(1-2)-99-1-1ML-5UL-205-30MIN.M
Last changed : 8/7/2020 2:26:02 PM
(modified after loading)
Additional Info : Peak(s) manually integrated

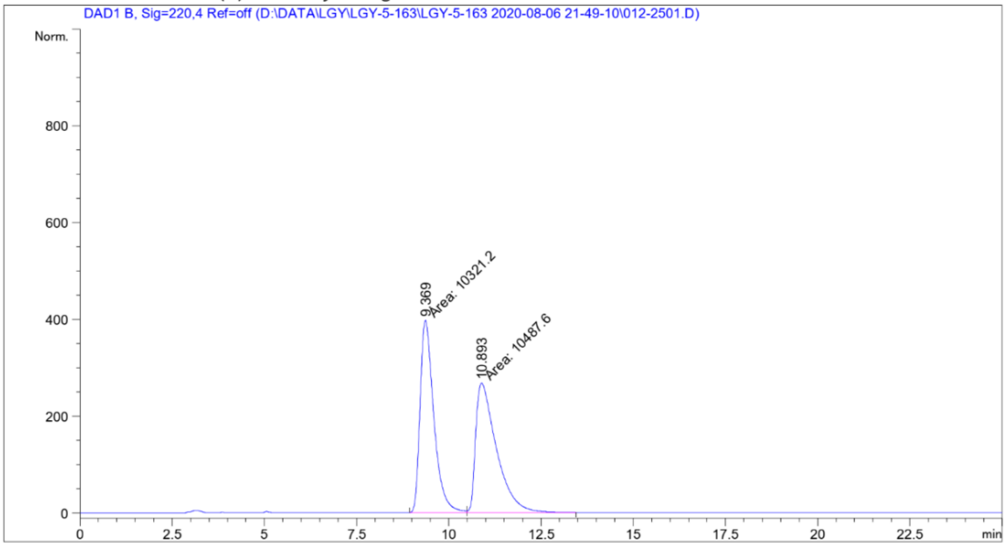

===
Area Percent Report
===

Sorted By : Signal
Multiplier : 1.0000
Dilution : 1.0000
Use Multiplier & Dilution Factor with ISTDs

Signal 1: DAD1 B, Sig=220,4 Ref=off

Peak #	RetTime [min]	Type	Width [min]	Area [mAU*s]	Height [mAU]	Area %
1	9.369	MF	0.4331	1.03212e4	397.15033	49.6003
2	10.893	FM	0.6538	1.04876e4	267.35117	50.3997

Totals : 2.08088e4 664.50150

```
Data File D:\DATA\LSL\LSL-5-111\LSL-5-111-OD 2020-08-04 07-38-15\012-0201.D
Sample Name: LGY-5-148-1

    =====================================================================
    Acq. Operator   :                               Seq. Line :   2
    Acq. Instrument : Instrument 2                   Location : Vial 12
    Injection Date  : 8/4/2020 7:53:39 AM                 Inj :   1
                                                   Inj Volume : 5.000 µl
    Acq. Method     : D:\DATA\LSL\LSL-5-111\LSL-5-111-OD 2020-08-04 07-38-15\DAD-OD(1-2)-80-20-
                      1ML-5UL-ALL-90MIN.M
    Last changed    : 8/4/2020 8:18:35 AM
                      (modified after loading)
    Analysis Method : D:\METHOD\LSL\DAD-OD(1-2)-99-1-1ML-5UL-205-30MIN.M
    Last changed    : 8/4/2020 5:37:04 PM
                      (modified after loading)
    Additional Info : Peak(s) manually integrated
```

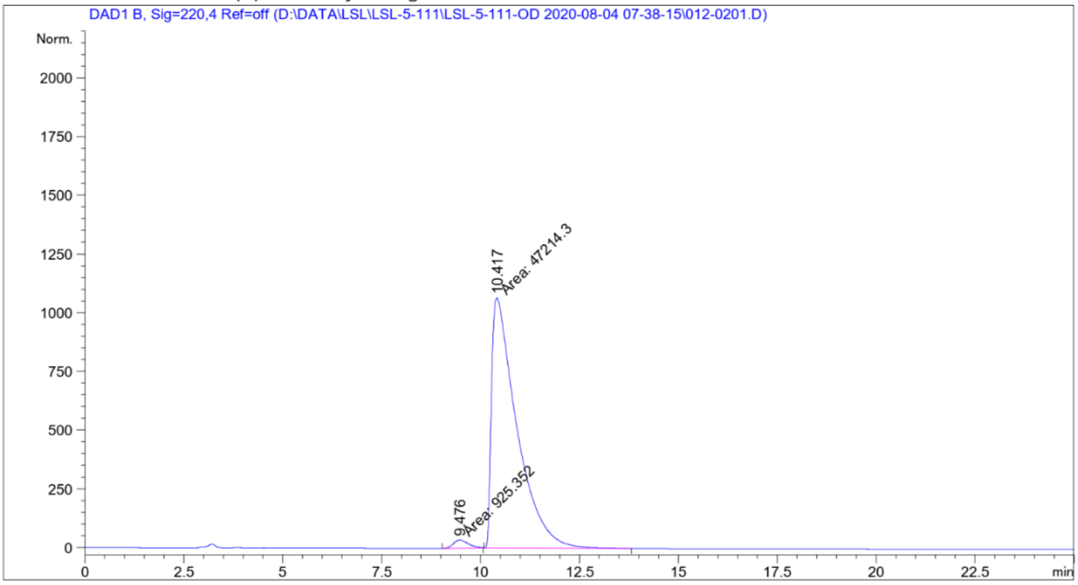


```
    =====================================================================
                             Area Percent Report
    =====================================================================

    Sorted By             :      Signal
    Multiplier            :      1.0000
    Dilution              :      1.0000
    Use Multiplier & Dilution Factor with ISTDs


    Signal 1: DAD1 B, Sig=220,4 Ref=off
```

Peak #	RetTime [min]	Type	Width [min]	Area [mAU*s]	Height [mAU]	Area %
1	9.476	MM	0.4352	925.35150	35.43519	1.9222
2	10.417	MM	0.7389	4.72143e4	1064.97449	98.0778

```
    Totals :                 4.81397e4  1100.40968
```


Supplementary Figure 94. HPLC spectra for compound 8

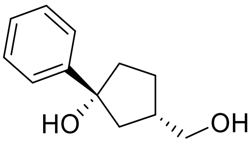

9

SHIMADZU LabSolutions

Analysis Report

<Sample Information>

Sample Name	: lsl-5-90-1-rac		
Sample ID	:		
Data Filename	: lsl-5-90-1-rac.gcd		
Method Filename	: bdex225-220-120~140-230-55min.gcm		
Batch Filename	: lsl-5-105-1.gcb		
Vial #	: 6	Sample Type	: Unknown
Injection Volume	: 1 uL		
Date Acquired	: 2020-7-29 16:32:43	Acquired by	: System Administrator
Date Processed	: 2020-7-29 17:27:47	Processed by	: System Administrator

<Chromatogram>

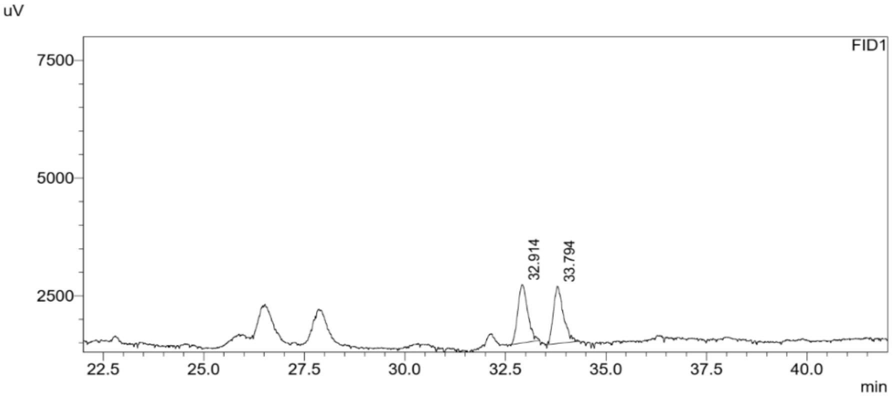

<Peak Table>

FID1

Peak#	Ret. Time	Area	Height	Conc.	Unit	Mark	Name
1	32.914	21128	1240	50.206		M	
2	33.794	20954	1212	49.794		M	
Total		42081	2451				

D:\DATA FILE\lsl\data\lsl-5-105-1\lsl-5-90-1-rac.gcd

SHIMADZU LabSolutions

Analysis Report

<Sample Information>

Sample Name	: lsl-5-105-1-c
Sample ID	:
Data Filename	: lsl-5-105-1-c.gcd
Method Filename	: bdex225-220-120~140-230-55min.gcm
Batch Filename	: lsl-5-105-1-2.gcb
Vial #	: 8
Sample Type	: Unknown
Injection Volume	: 1 uL
Date Acquired	: 2020-7-29 18:33:06
Acquired by	: System Administrator
Date Processed	: 2020-7-29 19:28:11
Processed by	: System Administrator

<Chromatogram>

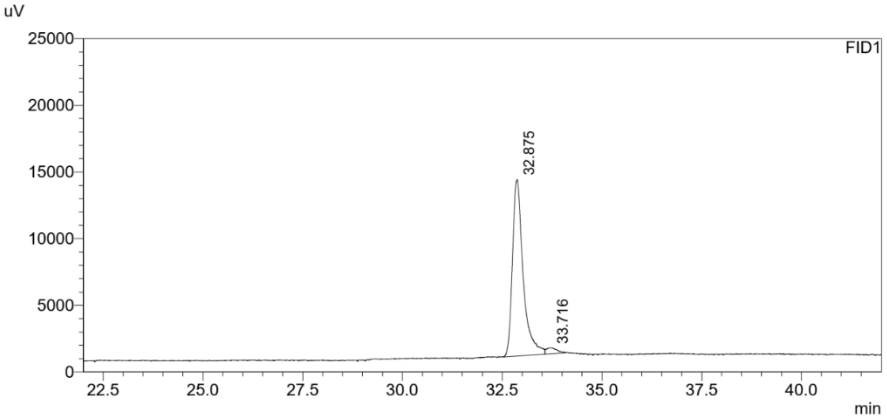

<Peak Table>

FID1

Peak#	Ret. Time	Area	Height	Conc.	Unit	Mark	Name
1	32.875	246082	13225	96.817		M	
2	33.716	8090	435	3.183		V M	
Total		254172	13660				

D:\DATA FILE\lsl\data\lsl-5-105-1\lsl-5-105-1-c.gcd

Supplementary Figure 95. GC spectra for compound 9

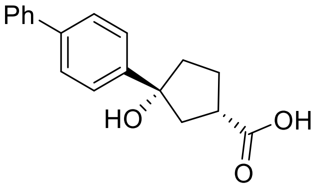

10

Data File D:\DATA\LGY\LCZ-1-31\LSL-5-131-OD-SHUJU 2020-08-25 19-00-23\051-0601.D
Sample Name: LSL-5-131-RAC-IC

===

Acq. Operator : Seq. Line : 6
Acq. Instrument : Instrument 2 Location : Vial 51
Injection Date : 8/25/2020 8:31:54 PM Inj : 1
Inj Volume : 5.000 µl
Acq. Method : D:\DATA\LGY\LCZ-1-31\LSL-5-131-OD-SHUJU 2020-08-25 19-00-23\DAD-IC(1-6)-90-10-1ML-5UL-ALL-40MIN.M
Last changed : 8/25/2020 9:02:46 PM
(modified after loading)
Analysis Method : D:\METHOD\LWD\DAD-OD(1-2)-95-5--0.75ML-5UL-ALL-20MIN.M
Last changed : 8/25/2020 9:40:18 PM
(modified after loading)
Additional Info : Peak(s) manually integrated

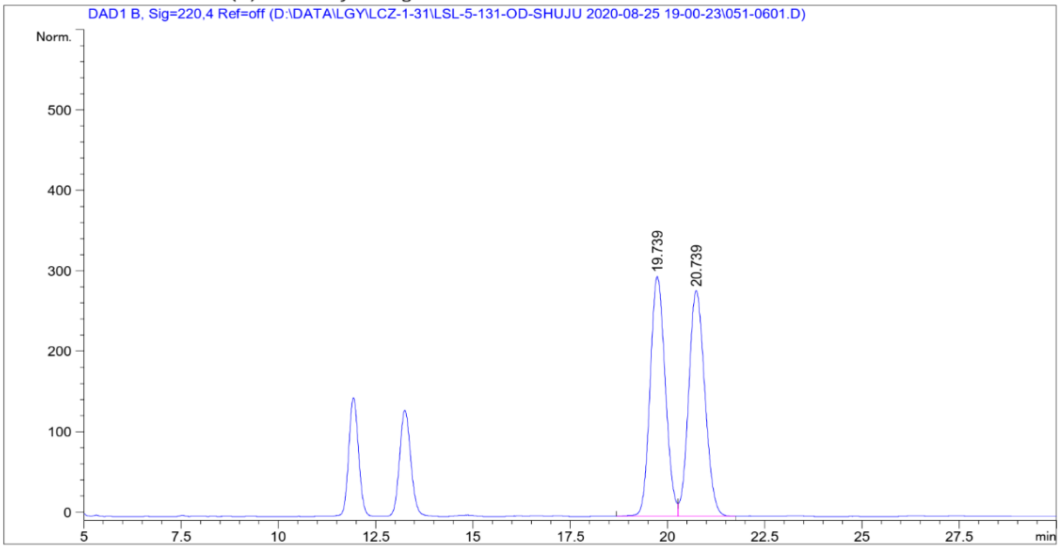

===

Area Percent Report

===

Sorted By : Signal
Multiplier : 1.0000
Dilution : 1.0000
Use Multiplier & Dilution Factor with ISTDs

Signal 1: DAD1 B, Sig=220,4 Ref=off

Peak #	RetTime [min]	Type	Width [min]	Area [mAU*s]	Height [mAU]	Area %
1	19.739	BV	0.4227	8088.63184	298.00751	49.9071
2	20.739	VB	0.4496	8118.74561	280.55249	50.0929

Totals : 1.62074e4 578.56000

Data File D:\DATA\LGY\LCZ-1-31\LSL-5-131-OD-SHUJU 2020-08-25 19-00-23\052-0701.D
Sample Name: LSL-5-131-IC-C

===

Acq. Operator : Seq. Line : 7
Acq. Instrument : Instrument 2 Location : Vial 52
Injection Date : 8/25/2020 9:03:49 PM Inj : 1
Inj Volume : 5.000 µl
Acq. Method : D:\DATA\LGY\LCZ-1-31\LSL-5-131-OD-SHUJU 2020-08-25 19-00-23\DAD-IC(1-6)-90-10-1ML-5UL-ALL-40MIN.M
Last changed : 8/25/2020 9:02:46 PM
(modified after loading)
Analysis Method : D:\METHOD\LWD\DAD-OD(1-2)-95-5--0.75ML-5UL-ALL-20MIN.M
Last changed : 8/25/2020 9:43:21 PM
(modified after loading)
Additional Info : Peak(s) manually integrated

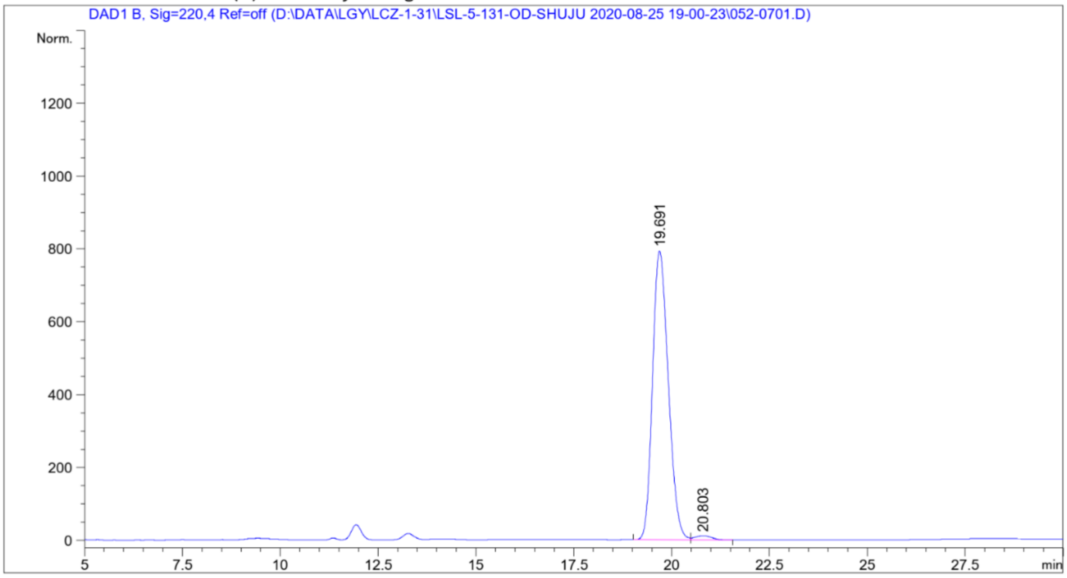

===
Area Percent Report
===

Sorted By : Signal
Multiplier : 1.0000
Dilution : 1.0000
Use Multiplier & Dilution Factor with ISTDs

Signal 1: DAD1 B, Sig=220,4 Ref=off

Peak #	RetTime [min]	Type	Width [min]	Area [mAU*s]	Height [mAU]	Area %
1	19.691	BV	0.4366	2.21842e4	792.51733	98.5689
2	20.803	VB	0.4269	322.09879	11.28784	1.4311

Totals : 2.25063e4 803.80517

Supplementary Figure 96. HPLC spectra for compound 10

Supplementary References

1. Ueda K, Umihara H, Yokoshima S, Fukuyama T. Conversion of Ester Moieties to 4-Bromophenyl Groups via Electrocyclic Reaction of Dibromocyclopropanes. *Org. Lett.* **17**, 3191-3193 (2015).
2. Gui Q, Wang J, Ng S, Dancevic A, Wright T B, Evans P A. Copper-catalyzed desymmetrization of prochiral 4,4-disubstituted cyclopentenes via a site-selective allylic oxidation: a concise total synthesis of untenone A. *Chem. Commun.* **55**, 12368-12371 (2019).
3. Rashid S, Bhat B A, Mehta G. Regenerative γ-Lactone Annulations: A Modular, Iterative Approach to Oligo-tetrahydrofuran Molecular Stairs and Related Frameworks. *Org. Lett.* **17**, 3604-3607 (2015).
4. Kuang Y, Anthony D, Katigbak J, Marrucci F, Humagain S, Diao T. Ni(I)-Catalyzed Reductive Cyclization of 1,6-Dienes: Mechanism-Controlled trans Selectivity. *Chem* **3**, 268-280 (2017).
5. Fort D A, Woltering T J, Nettekoven M, Knust H, Bach T. Conformationally restricted pyrrolidines by intramolecular [2+2] photocycloaddition reactions. *Chem. Commun.* **49**, 2989-2991 (2013).
6. Foarta F, Landis C R. Condensation Oligomers with Sequence Control but without Coupling Reagents and Protecting Groups via Asymmetric Hydroformylation and Hydroacyloxylation. *J. Org. Chem.* **81**, 11250-11255 (2016).
7. Lin J-B, Xu S-M, Xie J-K, Li H-Y, Xu P-F. An organocatalytic Michael-cyclization cascade of 4-oxa-α,β-unsaturated carboxylic acids with aldehydes: facile synthesis of chiral γ-lactols and trisubstituted γ-lactones. *Chem. Commun.* **51**, 3596-3599 (2015).
